# Annulative π-extension of phenothiazines: access to room temperature ultralong organic phosphorescent materials

**DOI:** 10.1039/d6sc04090j

**Published:** 2026-07-06

**Authors:** Chunlin Zhou, Shu Zhao, Lian Gou, Danni Yang, Haichao Liu, Bijin Li

**Affiliations:** a Chongqing Key Laboratory of Natural Product Synthesis and Drug Research, School of Pharmaceutical Sciences, Chongqing University Chongqing 401331 P. R. China bijinli@cqu.edu.cn; b State Key Laboratory of Supramolecular Structure and Materials, College of Chemistry, Jilin University Changchun Jilin 130012 P. R. China hcliu@jlu.edu.cn; c Department of Medical Oncology, The Second Medical Center and National Clinical Research Center for Geriatric Diseases, Chinese PLA General Hospital Beijing 100853 China

## Abstract

Here is reported the first example of annulative π-extension of phenothiazines to develop ultralong-lifetime room-temperature phosphorescent (RTP) materials based on a stepwise adjustment strategy. Through systematic modification of substituents and the degree of conjugation, the energy levels and spin–orbit coupling (SOC) values of the preliminary phosphorescent skeleton were systematically optimized, leading to the development of ultralong-lifetime RTP materials. In this work, we have developed the phenothiazine-class material with the longest-recorded phosphorescence lifetime (over 1.5 seconds) and a 15-second afterglow. Moreover, for the first time, ultralong-lifetime red RTP materials within the phenothiazine family are reported, featuring emission peaks at 644 nm and 660 nm and lifetimes of 273 milliseconds and 230 milliseconds. The novel RTP materials have demonstrated excellent applications in anti-counterfeiting, multilevel encryption, white light-emitting diodes (LEDs), optical security, luminescent display technologies, dual-channel cellular imaging, and photocatalysts. Meanwhile, the design and synthesis strategy *via* π-extension of phenothiazine derivatives represents a highly promising pathway for developing ultralong-lifetime RTP materials.

## Abstract 2

This work presents the first annulative π-extension of phenothiazines enabled by a stepwise structural tuning strategy, affording ultralong-lifetime room-temperature phosphorescent materials.

## Graphical abstract

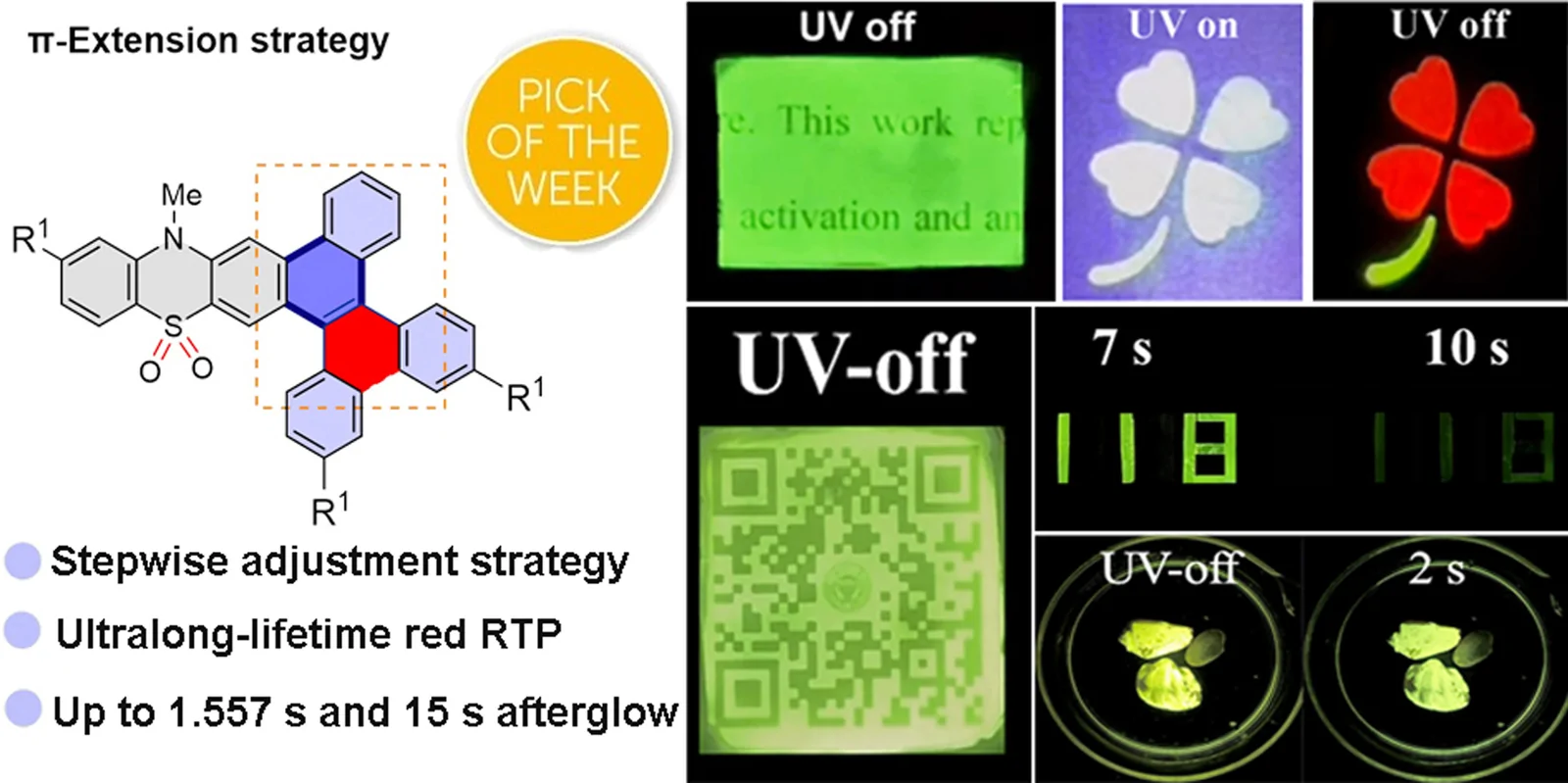

## Introduction

Luminescent materials serve as core functional materials underpinning modern technology, public health, industrial advancement, and cutting-edge scientific research.^1–8^ Breaking through the limitations of traditional lighting technologies, they have comprehensively reshaped human production patterns, lifestyles, medical and healthcare systems, and information interaction mechanisms. As such, they are among the key materials that drive the ongoing evolution of human civilization. Room-temperature ultralong organic phosphorescent (UORTP) materials constitute an emerging family of luminescent materials. Benefiting from their great potential for applications in bioimaging, anti-counterfeiting, emergency indicators, optoelectronic devices, and information storage, they have attracted widespread research interest.^9–16^ This is attributed to their distinctive advantages, including a high signal-to-noise ratio, large Stokes shift, exceptionally long emission lifetimes, and diverse excited-state properties.^1–3,17–26^ Unfortunately, achieving ultralong organic phosphorescence remains challenging, primarily due to inefficient spin–orbit coupling (SOC), which leads to ineffective intersystem crossing (ISC) and pronounced nonradiative decay under ambient conditions.^1–5,27–35^

**Scheme 1:**
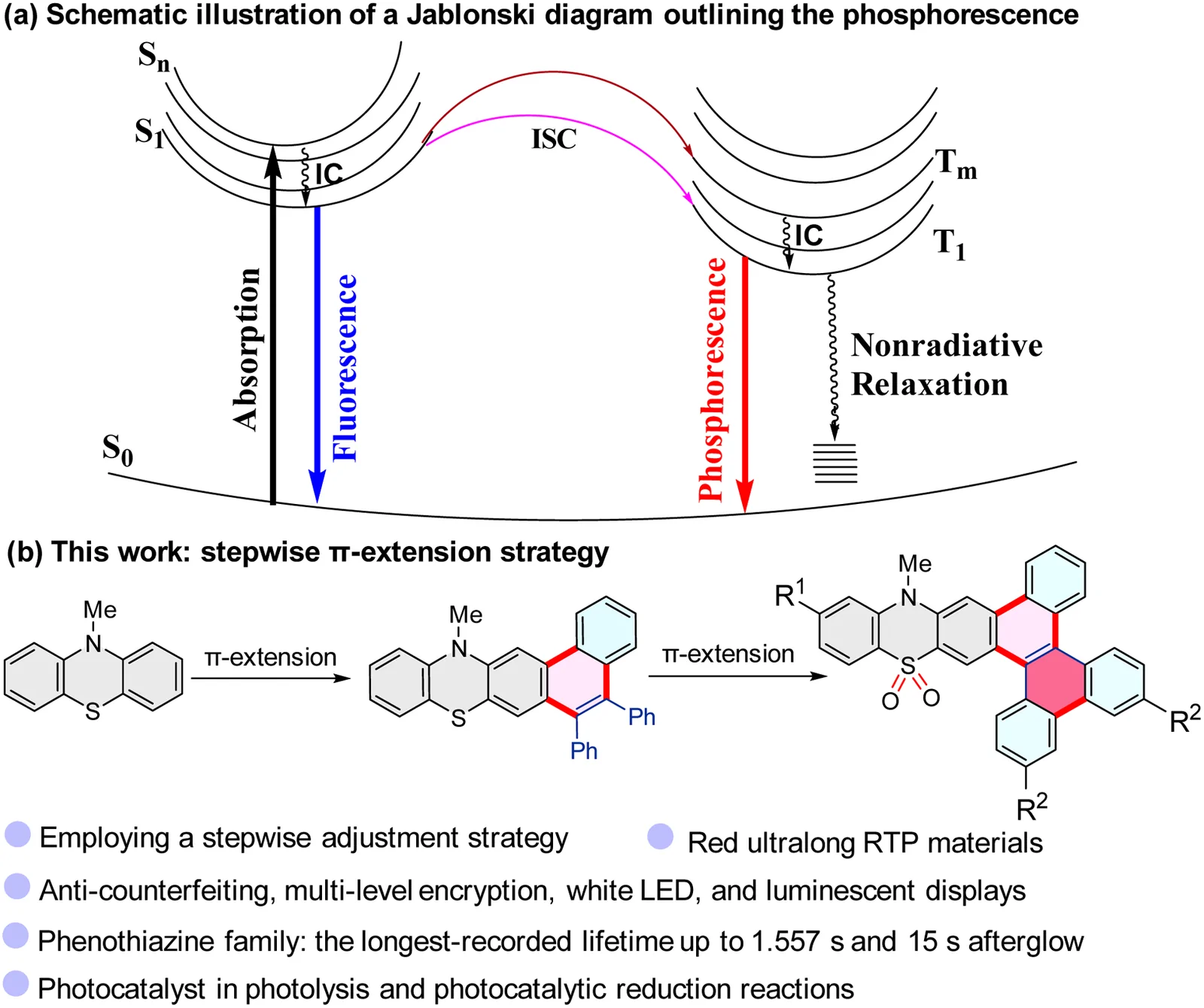
(a) Schematic illustration of a Jablonski diagram outlining the phosphorescence process. (b) Molecular design strategy and π-extension of phenothiazine derivatives.

Phosphorescence emission involves transitions from the first excited triplet (T_1_) to the ground singlet (S_0_) state in stable molecules (Scheme 1). Theoretically, promoting intersystem crossing from singlet to triplet states while suppressing nonradiative deactivation of triplet excitons represents a validated strategy for constructing long-lived RTP systems.^1–7,36–44^ Thus, introducing heteroatoms to enhance SOC, extending the π-conjugated framework to strengthen intermolecular interactions while suppressing nonradiative transitions, and doping polymers to mitigate nonradiative transition rates could serve as highly effective strategies for the development of ultralong organic phosphorescent materials.^1–7,40–49^

Phenothiazine possesses electron-rich sulfur and nitrogen heteroatoms and the characteristic non-planar “butterfly” structure, which favors the n–π* transition, promotes ISC from the excited singlet and triplet states, and could hinder intermolecular π–π stacking and suppress non-radiative transitions to some extent. Therefore, phenothiazine holds great potential for the development of ultralong room-temperature phosphorescent (URTP) materials (Scheme 1). Nevertheless, for 10-methyl-10*H*-phenothiazine, reverse intersystem crossing (RISC) is suppressed at low temperature (77 K). Although short-wavelength yellow-green phosphorescence emission can be detected, its luminescence lifetime remains relatively short. Moreover, almost no phosphorescence signal is observable at room temperature, accompanied by an extremely short triplet lifetime. To address these limitations and fabricate high-performance URTP materials, especially those with long-wavelength red phosphorescence emission, we propose a stepwise π-extension strategy to construct phenothiazine-based derivatives with ultralong phosphorescence properties (Scheme 1).

## Results and discussion

Based on the π-extension strategy, we designed a novel phosphorescent molecule 4a*via* π-extension of the 10-methyl-10*H*-phenothiazine core, as shown in Scheme 1. Subsequently, the synthetic routes of compound 4a were analyzed. In recent years, transition metal-catalyzed annulated π-extension reactions have emerged as one of the most efficient approaches for achieving molecular π-extension.^50–52^ Herein, we strategically designed NBE-CO_2_Me-mediated palladium-catalyzed C–H bond activation and annulation of 3-bromo-10-methyl-10*H*-phenothiazine (1a), 2-bromobenzoic acid (2a), and 1,2-diphenylethyne (3a) as substrates to enable the efficient construction of 4a. After screening several parameters (see SI, Table S1), we obtained a yield of 54% under the standard reaction conditions: 1a (0.1 mmol), 2a (2.0 equiv.), 3a (1.5 equiv.), Pd(OAc)_2_ (10 mol%), PhDavePhos (25 mol%), K_2_CO_3_ (4.5 equiv.), NBE-CO_2_Me (1.5 equiv.), toluene (0.1 M), 130 °C, and 72 h. In addition, a reasonably plausible catalytic cycle is proposed to elaborate the reaction mechanism based on the experimental observations (see SI, Fig. S2).

**Scheme 2:**
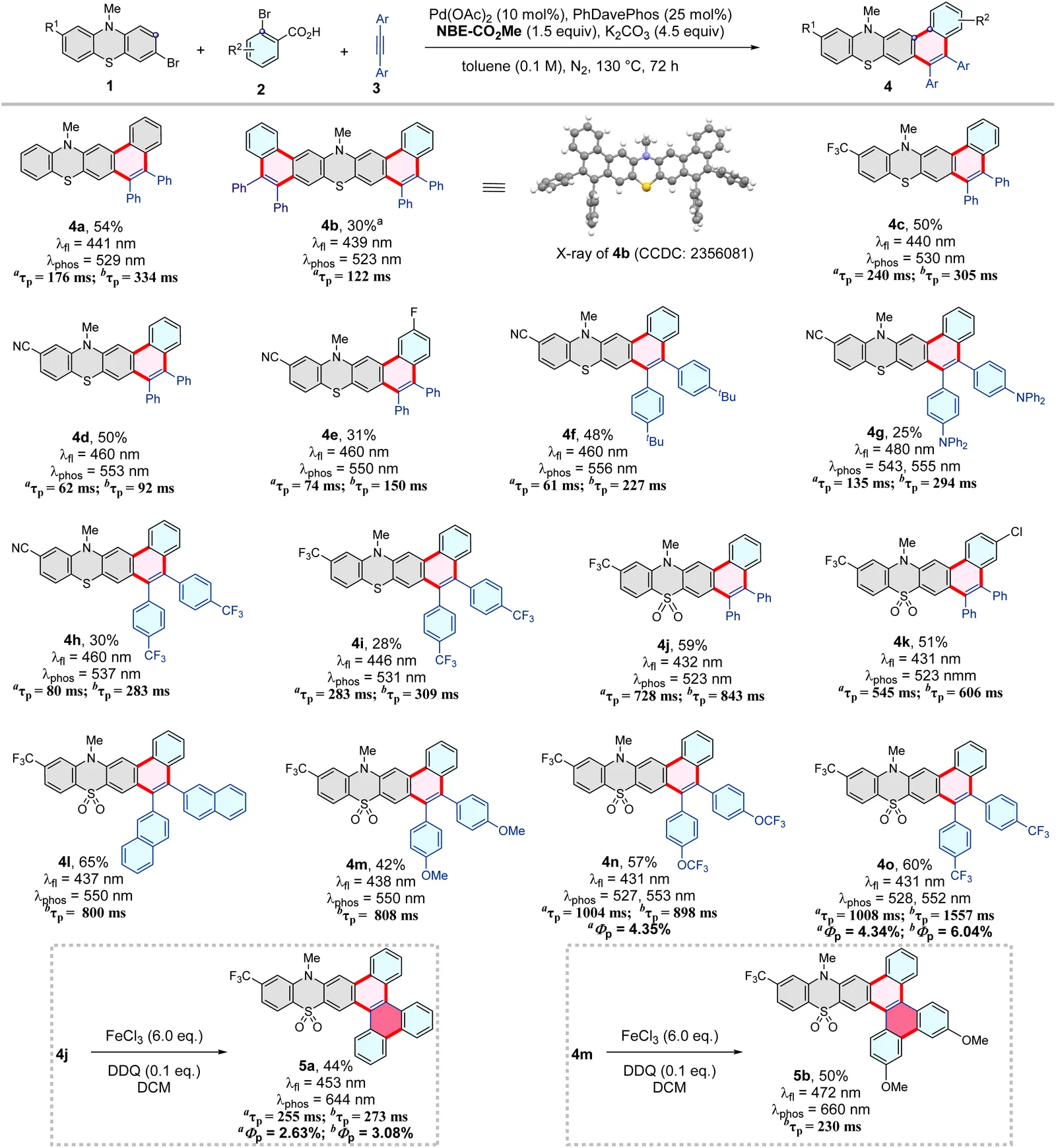
The synthesis of RTP materials. Reaction conditions: 1 (0.1 mmol), 2 (0.2 mmol), 3 (0.15 mmol), Pd(OAc)_2_ (10 mol%), PhDavePhos (25 mol%), NBE-CO_2_Me (1.5 equiv.), K_2_CO_3_ (4.5 equiv.), toluene (0.1 M), N_2_, 130 °C, and 72 h. Isolated yield. ^*a*^0.05 mmol scale. Test conditions for absorption and emission: absorption maximum in PMMA (1.0 wt%) or HEA-AA (0.05 wt%). Emission maximum in the PMMA film (1.0 wt%) or HEA-AA (0.05 wt%). Irradiation conditions: UV 365 nm and 30 s. Excitation slit: 2.5 nm, emission slit: 1.0 nm. *λ*_ex_ = 365 nm and PMT voltage = 700 V. Test parameters for the quantum yield: scan slit: 0.55 nm, fixed/offset slit: 2.5 nm, detector: PMT-900. *λ*_fl_ = fluorescence emission wavelength; *λ*_phos_ = phosphoresence emission wavelength; ^*a*^*τ*_p_ = phosphorescence lifetime in PMMA (1.0 wt%). ^*b*^*τ*_p_ = phosphorescence lifetime in HEA-AA (0.05 wt%).

As expected, 4a exhibits an ultralong room-temperature phosphorescence lifetime in PMMA and HEA-AA films (176 ms and 334 ms, HEA = hydroxyethyl acrylate; AA = acrylic acid), but both are less than 1 second (Scheme 2; Fig. S3 and S20, SI). We further fine-tuned the energy levels and SOC values of the primary phosphorescent molecule 4a by systematically modifying substituents (4c–4o) and conjugation degrees (4b, 5a, and 5b; Scheme 2). Subsequently, we synthesized a series of compounds with different substituents (4c–4o) under the optimized reaction conditions (Scheme 2). Molecules 4b, 5a, and 5b with increased conjugation were also synthesized by an annulated π-extension reaction or an intramolecular oxidative coupling reaction (Scheme 2). The corresponding structure of 4b was confirmed by single-crystal X-ray analysis (Table S7 and Fig. S53). All the compounds were purified by column chromatography and three recrystallizations to ensure purity and rule out the effect of impurities on photophysical properties. Exciting, the poly(methyl methacrylate) (PMMA) and HEA-AA films, based on 4b–5b, exhibited photo-activated organic RTP (Fig. S4–S16 and S21–S35). Hence, this work achieves the first example of NBE-CO_2_Me-mediated palladium-catalyzed C–H bond activation and annulation of bromo-phenothiazines to construct structurally diverse π-extension of phenothiazine-based room-temperature phosphorescent materials.

**Fig. 1:**
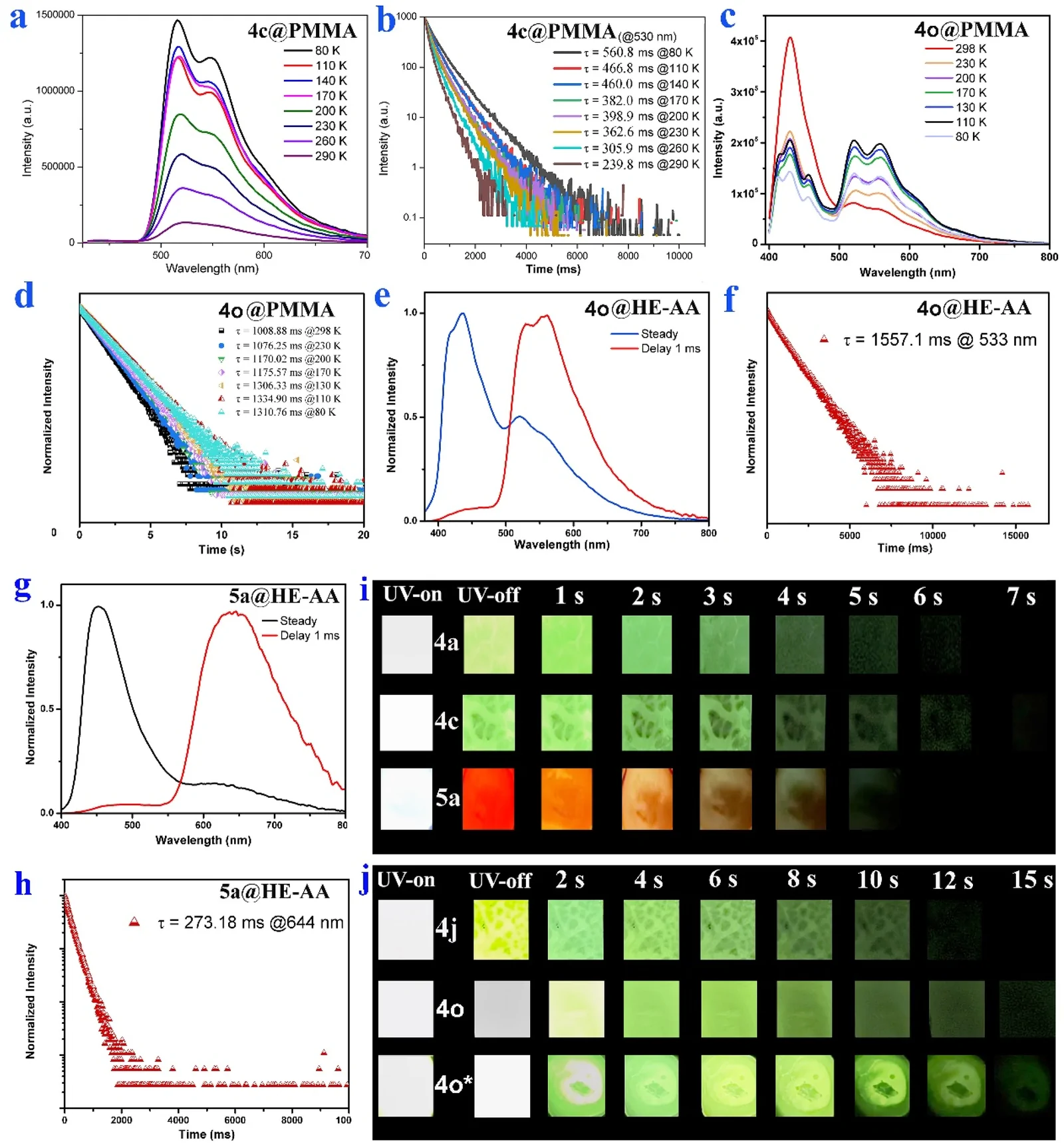
Luminescent properties. (a) Temperature-variable PL spectra of 4c in the PMMA film (1.0 wt%). (b) Temperature-dependent time-resolved phosphorescence decay curves of 4c in the PMMA film (1.0 wt%). (c) Luminescence spectra of 4o (1 wt%) in the PMMA film under 365 nm excitation at different temperatures ranging from 80 to 298 K. (d) Temperature-dependent time-resolved phosphorescence decay curves of 4o in the PMMA film (1.0 wt%). (e) Steady-state FL and delayed PL spectra of 4o@HEA-AA (0.05 wt%). (f) Lifetime decay profiles of phosphorescence at 533 nm of 4o@HEA-AA (0.05 wt%). (g) Steady-state FL and delayed PL spectra of 5a@HEA-AA (0.05 wt%). (h) Lifetime decay profiles of phosphorescence at 644 nm of 5a@HEA-AA (0.05 wt%). (i) Photographs of the long-lived luminescence from 4a, 4c in the PMMA film (1.0 wt%), and 5a@HEA-AA film (0.05 wt%) taken before and after turning off UV-365 nm excitation. (j) Photographs of the long-lived luminescence from 4j, 4o in the PMMA film (1.0 wt%), and 4o@HEA-AA film (0.05 wt%) taken before and after turning off UV-365 nm excitation.

The photophysical properties of 4c and 4o in the PMMA film (1.0 wt%) were further investigated in detail. The UV/Vis absorption spectra of 4c and 4o in the PMMA film (1.0 wt%) indicated absorption peaks at 360 nm and 380 nm (Fig. S38c and S40e). The photoluminescence (PL) spectra of 4c and 4o exhibited fluorescence emission at 440 nm and 431 nm, as well as phosphorescence emission at 530 nm and 528 nm, respectively (Fig. S5a and S15a). The temperature-dependent emission spectra and lifetimes further demonstrated that 4c and 4o in the PMMA film (1.0 wt%) exhibit phosphorescence emission (Fig. 1, S5 and S15). The intensity of emission and lifetimes highly depend on the temperature. For 4c, upon cooling from 290 K to 80 K, the emission intensity increased and the lifetime prolonged from 239.8 ms to 560.8 ms (Fig. 1a, b, S5d and e). For 4o, similar behavior was observed: the emission intensity increased and the lifetime extended from 1008.9 ms to 1310.8 ms when the temperature was lowered from 298 K to 80 K (Fig. 1c, d, S15e, and f). These observations indicate that the corresponding emissions are attributable to phosphorescence. The delayed spectra further confirmed the phosphorescent nature with a 1 ms delay, which exhibited only a single emission peak (Fig. 1e, S5c, and S15b). Other compounds in PMMA films (1.0 wt%) also showed only a single emission peak after a delay of 1 ms (Fig. S3–S16).

**Fig. 2:**
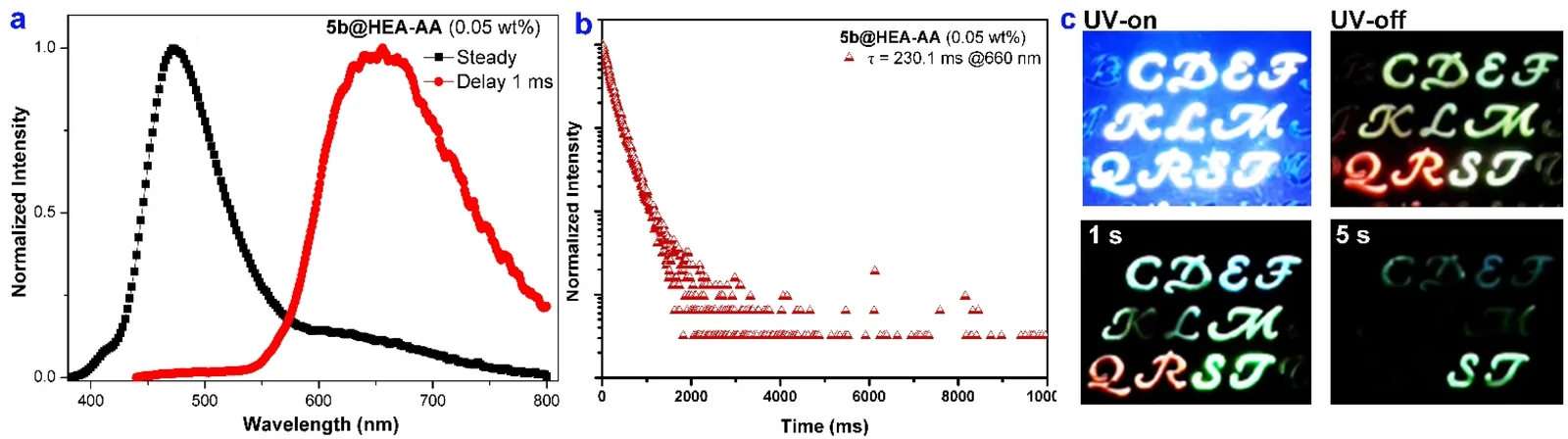
(a) Steady-state FL and delayed PL spectra of 5b@HEA-AA (0.05 wt%). (b) Lifetime decay profiles of phosphorescence at 660 nm of 5b@HEA-AA (0.05 wt%). (c) Preparation of letters using 4d, 4e, 4k, 4o and 5a (HEA-AA, 0.05 wt%), and their changes under UV light on/off conditions.

**Fig. 3:**
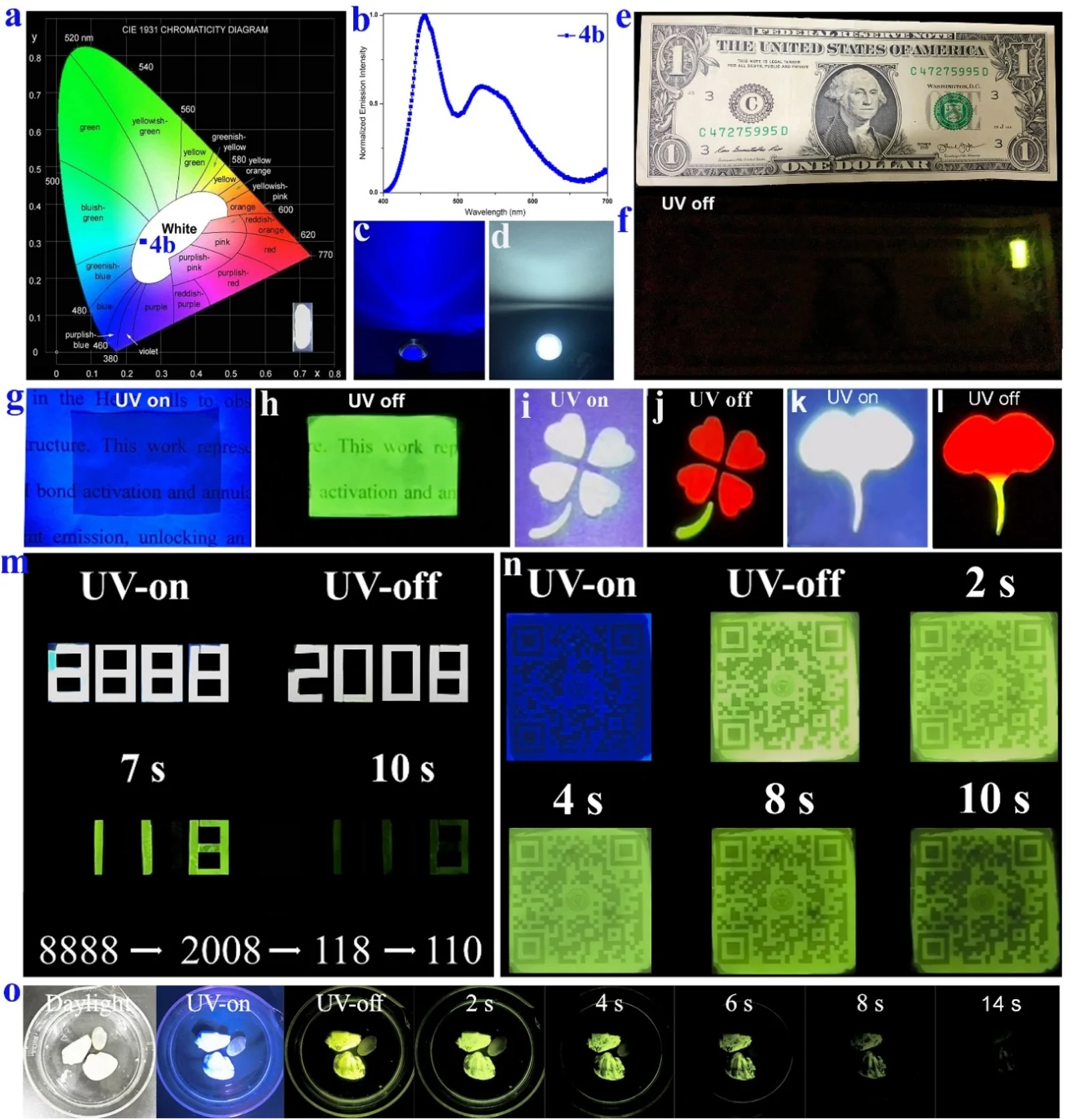
The applications of ultralong organic phosphorescent materials. (a) Emission color coordinates and luminescence images of 4b in the PMMA film (3.0 wt%): CIE 1931 chromaticity diagrams (0.25, 0.30). (b) The corresponding emission spectrum of 4b in the PMMA film (3.0 wt%). (c) Luminescence image of a commercially available UV lamp (365 nm). (d) Luminescence image of UV LED light coated with the 4b PMMA film (3.0 wt%) (turning on). (e) U.S. dollar. (f) Anti-counterfeiting application of the 4c film (1.0 wt%) in U.S. dollar. (g) and (h) Transparency and afterglow intensity testing of the 4c PMMA film (0.1 wt%). (i) and (j) The flower was prepared using 5a and 4c in PMMA films. (k) and (l) The flower was prepared using 5b and 4o in HEA-AA(0.05 wt%) films. (m) Changes in anticounterfeiting information under 365 nm excitation. PMMA films doped with 4b, 4c, 4j, and 4o at a mass fraction of 1.0 wt%, respectively, were used. (n) Information encryption with a QR code using 4o@PMMA (1.0 wt%). The QR code links to the website of Chongqing University. (o) Luminescence images of the underwater process of the shells covered with the 4o@PMMA film (1.0 wt%, *λ*_ex_ = 365 nm).

Impressively, π-conjugated molecules 4a, 4c, 4j, and 4o in PMMA films (1.0 wt%) possessed yellow-green afterglow up to more than six seconds (Fig. 1) after the UV light was turned off. The 4o-doped HEA-AA film (0.05 wt%) exhibits a phosphorescence quantum yield of 6.0% and a lifetime of 1557 ms, which is currently reported as the longest lifetime among phenothiazine-based phosphorescent materials (Fig. 1e, f, j, S33 and S36). Furthermore, compound 4o does not exhibit RTP properties in its crystalline form. The PXRD patterns of 4o in its crystalline form, PMMA film, and HEA-AA film are distinct, a difference that reflects variations in molecular packing (Fig. S46). Specifically, 4o exists in an amorphous state in the PMMA and HEA-AA films. The red ultralong organic phosphorescent materials are scarce, which exhibit significant application potential in the field of bioimaging.^13,53^ Notably, the HEA-AA film doped with 5a (0.05 wt%) exhibits red phosphorescence at 644 nm, featuring an ultralong phosphorescence lifetime of 273 ms (Fig. 1g–i, and S34). Similarly, the film containing 5b (0.05 wt%) emits red phosphorescence at 660 nm, with an ultralong phosphorescence lifetime of 230 ms (Fig. 2a, b and S35). Subsequently, 4d, 4e, 4k, 4o, and 5a with different afterglows (in HEA-AA, 0.05 wt%) were prepared into letters. When the UV light is turned off, we can see different color letter patterns (Fig. 2c). In addition, compound 4b displayed dual emission with blue emission (fluorescent component) at approximately 455 nm and yellow emission (phosphorescence component) at 533 nm in the PMMA film (3.0 wt%) with white-light CIE coordinates of (0.25, 0.30) (Fig. 3a and b). The thermogravimetric analysis measurement reveals that 4b is a highly thermally stable material, with its thermal-decomposition temperature (*T*_d_) at 5% weight loss reaching 410 °C (Fig. S45). We further coated the 4b film (3.0 wt% in PMMA) on a commercially available UV light-emitting diode (LED) to rapidly fabricate a robust organic and low-cost white LED, and bright white light emissions can be observed when the LED is turned on (Fig. 3c and d).

Based on the aforementioned investigations and characterization of the photophysical properties of these π-conjugated molecules, we further summarize their structure–property relationships. Extending the π-conjugated framework at the 2- and 3-positions of 10-methyl-10*H*-phenothiazine furnished molecule 4a, a room-temperature phosphorescent material that emits at 529 nm with a lifetime of 176 ms. This result verifies that expanded π-conjugation converts parent 10-methyl-10*H*-phenothiazine into an efficient room-temperature phosphor. Further extension of the π-conjugated framework at the C7 and C8 positions of the 4a scaffold afforded molecule 4b, a room-temperature phosphor exhibiting phosphorescence emission at 523 nm with a lifetime of 122 ms. This result indicates that π-conjugation expansion at the 7,8-sites of 4a induces neither a red shift of the phosphorescence band nor a prolonged excited-state lifetime. Modification of the substituent at the C8 position of the 4a scaffold afforded compound 4c bearing a CF_3_ group, whose room-temperature phosphorescence lifetime was prolonged to 240 ms. Subsequent oxidation of the sulfur atom to the corresponding sulfone delivered derivative 4j with a drastically extended RTP lifetime of 728 ms. Further expansion of the π-conjugated system of 4j yielded molecule 5a, which exhibited a shortened lifetime of 255 ms alongside a red-shifted emission maximum at 644 nm. Moreover, introducing extra CF_3_ groups to 4j afforded molecule 4o with the maximum RTP lifetime of 1008 ms. Structure–property relationship analyses indicate that enhanced π-conjugation, CF_3_ heteroatom substitution, and sulfone modification substantially alter the room-temperature phosphorescence characteristics of the target compounds.

We further explored their application in optical anti-counterfeiting and luminescent displays based on the excellent phosphorescence properties of the doped films. The anti-counterfeiting 4c film is smeared on the numeral “1” of the U.S. dollar (Fig. 3e). After turning off the 365 nm UV light, we could still see a yellow-green afterglow pattern, which demonstrated that it has great potential in optical anti-counterfeiting (Fig. 3f). When 4c in the PMMA film (0.1 wt%) was placed on a paper with text, the text on the paper could be observed clearly through the film under 365 nm UV light, which revealed the excellent transparency of the film (Fig. 3g). Then, turning off the UV light, the text still could be observed on the paper owing to the strong phosphorescence emitted by the 4c film itself, which indicated its good luminescent display application (Fig. 3h). Moreover, when preparing the beautiful flowers using 5a and 4c in PMMA films or 5b and 4o in HEA-AA(0.05 wt%) films, upon turning off the UV lamp, flowers with red petals and green branches were observed (Fig. 3i–l). Furthermore, the digit “8888” was prepared using 4b, 4c, 4j and 4o. Upon illumination of a 365 nm UV lamp, the pattern displayed the white “8888”, and after the UV lamp was turned off, the white image of “2008” was observed. After 7 s, the yellow-green afterglow of the security code of “118” was observed and after 10 s, only the green afterglow of the distress signal code of “110” was observed (Fig. 3m). In addition, when the 4o-doped PMMA film (1.0 wt%) was placed over a QR code, the QR code exhibited high resolution and remained scannable by cellphones even 10 seconds after the UV light was turned off (Fig. 3n). Applying the 4o PMMA film onto the surface of the shell, an afterglow display lasting over 14 seconds can also be observed in water (Fig. 3o). Even after 300 days, the phosphorescence emission of the shells remains clearly detectable in aqueous environments, demonstrating the phosphorescent material's stable luminescence performance under water. These findings highlight its potential applications in the marking and tracking of underwater shellfish (Fig. 3o).

**Fig. 4:**
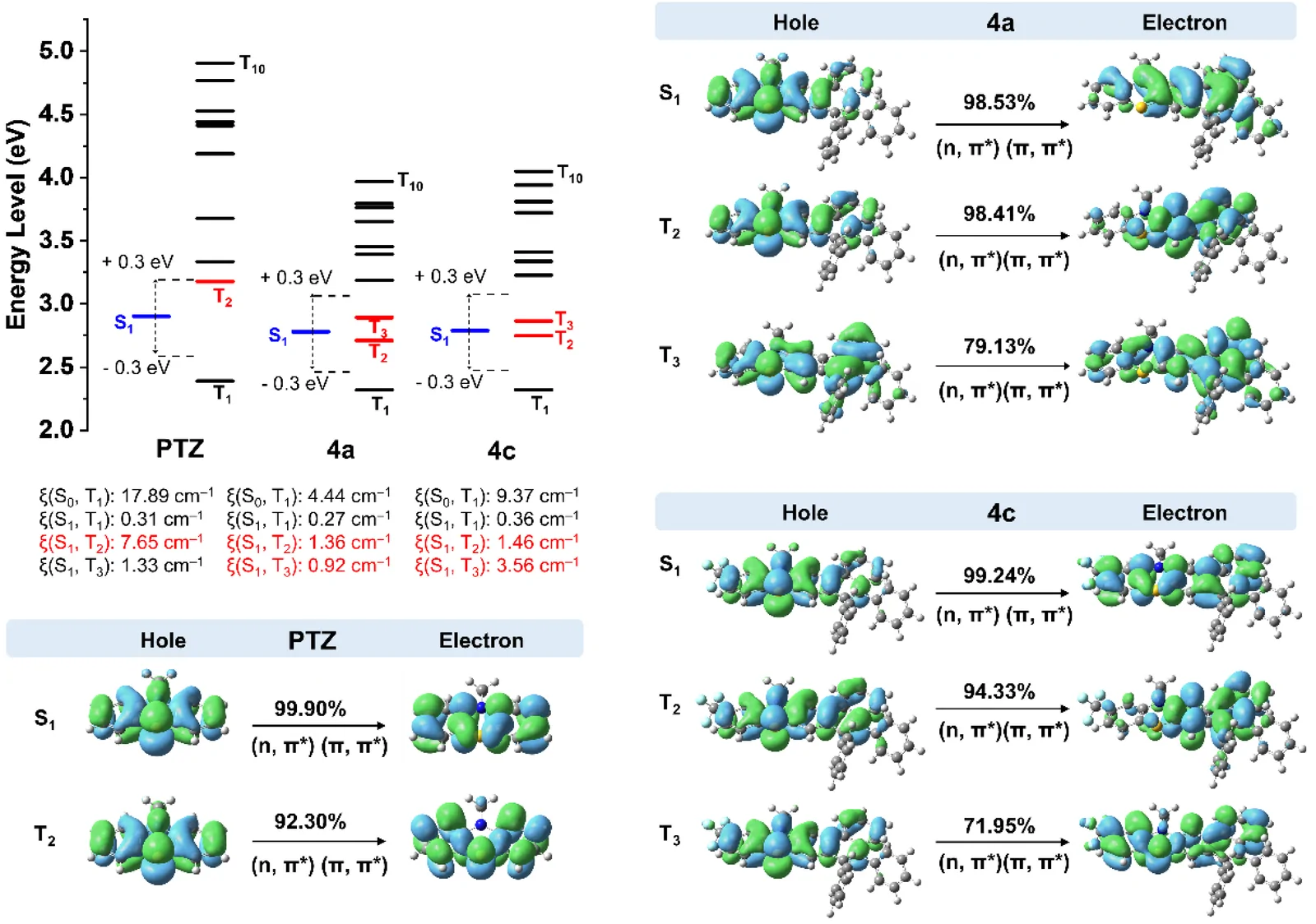
Energy level diagrams, SOC coefficients, and NTOs based on optimized geometries of S_1_ for PTZ, 4a, and 4c.

Subsequently, to further aid in understanding phosphorescence emission, the natural transition orbitals (NTOs), energy levels, and SOC values of the singlet and triplet states for compounds 10-methyl-10*H*-phenothiazine (PTZ), 4a, and 4c were systematically calculated (Fig. 4; Tables S3–S6 and Fig. S47–S50, section 7, SI). The S_1_ state of the reference compound PTZ exhibits coexisting electronic transition characters, ^1^(n,π*) and ^1^(π,π*) (Fig. 4). Notably, the energy gap between the S_1_ and T_2_ states (Δ*E*_S1–T2_ = 0.27 eV) is appropriate. The T_2_ state also exhibits coexisting ^1^(n,π*) and ^1^(π,π*) electronic transition configurations. Although the substantial SOC coefficient (7.65 cm^−1^) between S_1_ and T_2_ facilitates the ISC process, the large energy gap between T_2_ and T_1_ (Δ*E*_T2–T1_ = 0.78 eV) may hinder the IC process, thus limiting the population of T_1_ excitons in PTZ. Furthermore, the exceptionally large SOC coefficient (17.89 cm^−1^) between T_1_ and S_0_ can accelerate nonradiative decay pathways, making it difficult for PTZ to achieve efficient and long-lived RTP emission (Fig. 4, S47 and S50). Similarly, for the π-extended phenothiazine derivatives (4a and 4c), their S_1_ state retains coexisting transition characters (^1^(n,π*) and ^1^(π,π*)) (Fig. 4). However, the energy gaps between the S_1_ state and higher-lying triplet states (T_*n*_, *n* = 2, 3) are smaller compared to those of PTZ. As for the T_*n*_ states (*n* = 2, 3) of 4a and 4c, they exhibit coexisting ^3^(n,π*) and ^3^(π,π*) transition configurations with dominant^3^(π,π*) transition configurations due to extended π-conjugation, which results in moderate SOC coefficients. According to the first-order perturbation theory,^54^ the small energy gap between the excited singlet and triplet states and the moderate SOC coefficients favor the ISC process. Furthermore, for 4a and 4c, the small energy gap between T_2_ and T_1_ and the small SOC coefficient between T_1_ and S_0_ may promote the population of T_1_ excitons and suppress the nonradiative decay pathways, which facilitates the increase in both phosphorescence efficiency and phosphorescence lifetime. For these reasons, PTZ lacks RTP properties in the PMMA film, whereas 4a and 4c exhibit prominent RTP properties. Collectively, the experimental and computational results demonstrate that an increase in molecular conjugation leads to denser energy-level splitting and better-matched excited states, thereby creating more opportunities to achieve high-performance RTP materials.

**Fig. 5:**
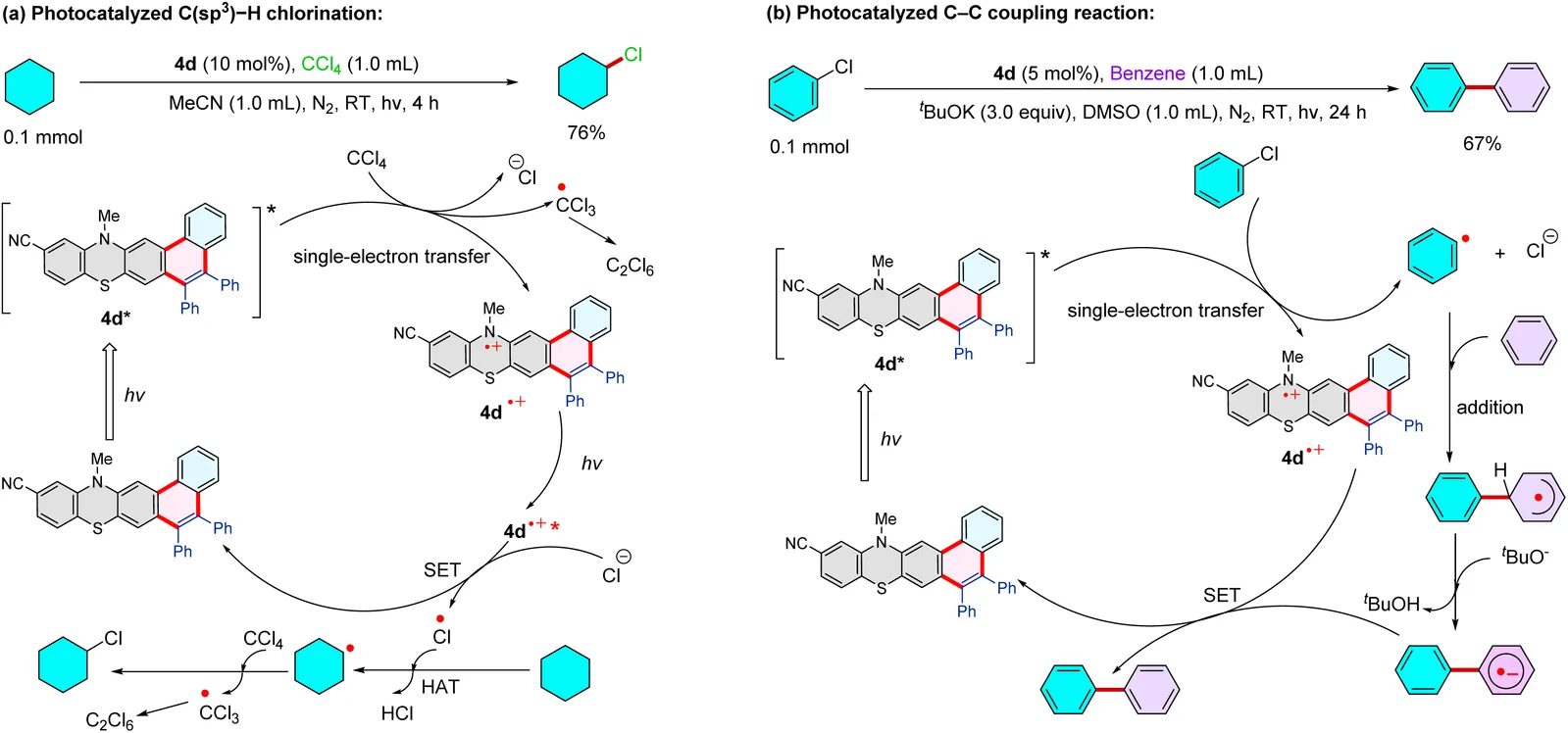
The applications of π-extended phenothiazine derivatives in the field of photocatalysis. (a) Chlorination of unactivated cyclohexane C(sp^3^)–H bonds. (b) The C–C cross-coupling reaction for the generation of biphenyl. Proposed plausible mechanism of photocatalytic C–H functionalization reactions. HAT: hydrogen atom abstraction.

Anti-Kasha dual-emissive materials have attracted research interest due to their tremendous potential for high-precision analytical studies in fundamental life sciences.^55,56^ Furthermore, we explored the applications of the synthesized π-extended phenothiazine derivatives in cell imaging. Compounds 4d and 4g exhibit distinct anti-Kasha dual-emission characteristics (Fig. S43). Subsequently, compound 4g was fabricated into water-dispersible nanoparticles (NPs) with Pluronic F188 as the matrix by the thin-film hydration method. Cytotoxicity assays of 4g NPs showed negligible toxicity toward HeLa cells (Fig. S52, Section 8, SI). Dual-channel imaging of 4g NPs was performed in HeLa cells. Confocal laser scanning microscopy revealed the spatial distribution of green (anti-Kasha emission) and red (Kasha emission) signals upon excitation at 405 nm (Fig. S51). These results demonstrate that compound 4g exhibits considerable potential for application in dual-channel cellular imaging. In addition, the photocatalytic C–H functionalization reactions have attracted significant attention in recent years.^57–64^ We further explored the applications of π-extended phenothiazine derivatives in photocatalysis. Specifically, compound 4d was employed as a photocatalyst for the chlorination of unactivated C(sp^3^)–H bonds in cyclohexane (Fig. 5; Section 9, SI). Moreover, 4d was utilized as a photocatalyst in the C–C cross-coupling reaction for the generation of biphenyl (Fig. 5). The experimental results show that compound 4d, as an effective photocatalyst, can be applied not only to the chlorination of unactivated cyclohexane C(sp^3^)−H bonds but also to the C–C cross-coupling reaction for the generation of biphenyl (Fig. 5).

## Conclusions

In conclusion, we first designed the primary phenothiazine-based phosphorescent molecule, followed by developing NBE-CO_2_Me-mediated palladium-catalyzed C–H bond activation and annulation of bromo-phenothiazines for its rapid and efficient synthesis. We fine-tuned the energy levels and SOC values of the primary phosphorescent molecule by systematically modifying substituents and conjugation degrees. Employing a stepwise adjustment strategy, we successfully screened out ultralong-lifetime RTP materials and red phosphorescent materials. Their applications in anti-counterfeiting, multi-level encryption, white LEDs, optical anti-counterfeiting, luminescent displays, and dual-channel cellular imaging were systematically investigated. Furthermore, the novel π-extension of phenothiazine derivatives was found to serve as an effective photocatalyst in photolysis and photocatalytic reduction reactions. In this work, we have developed the phenothiazine-class material with the longest-recorded phosphorescence lifetime (over 1.5 seconds) and a 15-second afterglow. Moreover, an ultralong-lifetime red RTP material within the phenothiazine family is reported for the first time, with emission peaks at 644 nm and 660 nm and lifetimes of 273 ms and 230 ms, respectively. To the best of our knowledge, this result is a significant breakthrough in RTP and will open up a new avenue for the synthesis and discovery of novel ultralong-lifetime RTP materials.

## Author contributions

C. Z., S. Z., L. G., D. Y. performed the experiments and analyzed the data. S. Z., H. L. performed the corresponding theoretical calculations and revised the manuscript. B. L. designed and directed the project and wrote the manuscript. All authors contributed to discussions.

## Conflicts of interest

There are no conflicts to declare.

## Supplementary Material

SC-017-D6SC04090J-s001

SC-017-D6SC04090J-s002

## Data Availability

The authors confirm that all data supporting the findings of this study are available within the manuscript and its supplementary information (SI) files. If required in alternative formats, they are available from the corresponding author upon reasonable request. Supplementary information: detailed experimental protocols, characterization data (NMR spectra, mass spectra, UV-Vis spectra, and computational data), reaction yields, and characterization parameters for all synthesized compounds. See DOI: https://doi.org/10.1039/d6sc04090j. CCDC 2356081 contains the supplementary crystallographic data for this paper.^65^

## References

[cit1] Zhang T., Ma X., Wu H., Zhu L., Zhao Y., Tian H. (2020). Angew. Chem., Int. Ed..

[cit2] Gan N., Shi H., An Z., Huang W. (2018). Adv. Funct. Mater..

[cit3] Gao H., Ma X. (2021). Aggregate.

[cit4] Du J., Wang X., Sun S., Wu Y., Jiang K., Li S., Lin H. (2024). Adv. Mater..

[cit5] Ding B., Ma X., Tian H. (2023). Acc. Mater. Res..

[cit6] Law A. W. K., Cheung T. S., Zhang J., Leung N. L. C., Kwok R. T. K., Zhao Z., Sung H. H. Y., Williams I. D., Qiu Z., Alam P., Lam J. W. Y., Tang B. Z. (2024). Adv. Mater..

[cit7] Sun T., Zhang J., Zhang J., Yang X., Yang D., Yang T., Li B. (2025). J. Am. Chem. Soc..

[cit8] Tu L., Chen Y., Song X., Jiang W., Xie Y., Li Z. (2024). Angew. Chem., Int. Ed..

[cit9] Liu J., Zhou X., Tang X., Tang Y., Wu J., Song Z., Jiang H., Ma Y., Li B., Lu Y., Li Q. (2025). Adv. Funct. Mater..

[cit10] Guo D., Wang W., Zhang K., Chen J., Wang Y., Wang T., Hou W., Zhang Z., Huang H., Chi Z., Yang Z. (2024). Nat. Commun..

[cit11] He Z., Song J., Li C., Huang Z., Liu W., Ma X. (2025). Adv. Mater..

[cit12] Wang J., Yang Y., Zhang L., Li Z. (2025). Adv. Mater..

[cit13] Yang G., Hao S., Dan Y., Dang L., Zhang H., Zhang Q., Li A., Li M.-D., Yuan W. Z. (2025). Adv. Mater..

[cit14] Ma L., Xu Q., Sun S., Ding B., Huang Z., Ma X., Tian H. (2022). Angew. Chem., Int. Ed..

[cit15] Song J., Ma L., Sun S., Tian H., Ma X. (2022). Angew. Chem., Int. Ed..

[cit16] Yang X., Li N., Wang B., Chen P., Ma S., Deng Y., Lü S., Tang Y. (2024). Angew. Chem., Int. Ed..

[cit17] Wan L., Ling S., Yang L., Li B. (2025). Chem. Sci..

[cit18] Li C., Lou Z., Wu M., Ma F., Chen X., Tan H., Liu Z., Gao F., Qiu Z., Zhao Z., Hu L., Xie G., Li M., Guo Y., Ren Z., Zhang S., Liu Y., Yan S., Li Z., Xu B., Kwok R. T. K., Lam J. W. Y., Tang B. Z. (2025). J. Am. Chem. Soc..

[cit19] Xiao Y., Li J., Song Z., Liao J., Shen M., Yu T., Huang W. (2025). J. Am. Chem. Soc..

[cit20] Cui D., Zhang L., Zhang J., Li W., Chen J., Guo Z., Sun C., Wang Y., Wang W., Li S., Huang W., Zheng C., Chen R. (2024). Angew. Chem., Int. Ed..

[cit21] Zhang J., Zhang S., Sun C., Wang R., Guo Z., Cui D., Tang G., Li D., Yuan J., Lu X., Zheng C., Huang W., Chen R. (2025). Adv. Mater..

[cit22] Partanen I., Hsu C. H., Shi E. H. C., Maisuls I., Eskelinen T., Karttunen A. J., Saarinen J. J., Strassert C. A., Belyaev A., Chou P. T., Koshevoy I. O. (2025). Angew. Chem., Int. Ed..

[cit23] Chen P., Qie H., Yang X., Ma S., Wang Z., Li N., Deng Y., Bian F., Lü S. (2024). Adv. Funct. Mater..

[cit24] Wang G., Chen X., Zeng Y., Li X., Wang X., Zhang K. (2024). J. Am. Chem. Soc..

[cit25] Zhang Y., Su Y., Wu H., Wang Z., Wang C., Zheng Y., Zheng X., Gao L., Zhou Q., Yang Y., Chen X., Yang C., Zhao Y. (2021). J. Am. Chem. Soc..

[cit26] Yang Z., Qian J., Zhao S., Lv Y., Feng Z., Wang S., He H., Zhang S. T., Liu H., Yang B. (2025). Angew. Chem., Int. Ed..

[cit27] Yang H., Wang Y., Yao X., Ma H., Yu J., Li X., Wang X., Liang X., Peng Q., Cai S., An Z., Huang W. (2024). J. Am. Chem. Soc..

[cit28] Hou H., Wang H., He M., Li Q., Wang X., Guo F., Chen Q., Qu L., Yang C. (2024). Angew. Chem., Int. Ed..

[cit29] Si C., Wang T., Gupta A. K., Cordes D. B., Slawin A. M. Z., Siegel J. S., Zysman-Colman E. (2023). Angew. Chem., Int. Ed..

[cit30] Zhou L., Song J., He Z., Liu Y., Jiang P., Li T., Ma X. (2024). Angew. Chem., Int. Ed..

[cit31] Jiang P., Ding B., Yao J., Zhou L., He Z., Huang Z., Yin C., Tian H., Ma X. (2025). Angew. Chem., Int. Ed..

[cit32] Chen C., Chi Z., Chong K. C., Batsanov A. S., Yang Z., Mao Z., Yang Z., Liu B. (2020). Nat. Mater..

[cit33] Ma F., Wu B., Zhang S., Jiang J., Shi J., Ding Z., Zhang Y., Tan H., Alam P., Lam J. W. Y., Xiong Y., Li Z., Tang B. Z., Zhao Z. (2025). J. Am. Chem. Soc..

[cit34] Yang X., Yang D., Li B. (2025). J. Am. Chem. Soc..

[cit35] Li X., Li W., Deng Z., Ou X., Gao F., He S., Li X., Qiu Z., Kwok R. T. K., Sun J., Phillips D. L., Lam J. W. Y., Guo Z., Tang B. Z. (2025). J. Am. Chem. Soc..

[cit36] Tian S., Ma H., Wang X., Lv A., Shi H., Geng Y., Li J., Liang F., Su Z. M., An Z., Huang W. (2019). Angew. Chem., Int. Ed..

[cit37] Kong S., Wang H., Liao J., Xiao Y., Yu T., Huang W. (2024). Adv. Mater..

[cit38] Dong M., Lv A., Zou X., Gan N., Peng C., Ding M., Wang X., Zhou Z., Chen H., Ma H., Gu L., An Z., Huang W. (2024). Adv. Mater..

[cit39] Yang J., Gao X., Xie Z., Gong Y., Fang M., Peng Q., Chi Z., Li Z. (2017). Angew. Chem., Int. Ed..

[cit40] Wang Y., Yang J., Fang M., Gong Y., Ren J., Tu L., Tang B. Z., Li Z. (2021). Adv. Funct. Mater..

[cit41] Wang Y., Gao H., Yang J., Fang M., Ding D., Tang B. Z., Li Z. (2021). Adv. Mater..

[cit42] Gao M., Tian Y., Li X., Gong Y., Fang M., Yang J., Li Z. (2023). Angew. Chem., Int. Ed..

[cit43] Zhang Y., Gao M., Wu R., Meng Y., Li N., Chen Z., Fang M., Yang J., Li Z. (2025). Adv. Funct. Mater..

[cit44] Xu D., Wang T., Liu S., Pu G., Yang J., Fang M., Liu X., Li Z. (2025). CCS Chem..

[cit45] Yang J., Zhen X., Wang B., Gao X., Ren Z., Wang J., Xie Y., Li J., Peng Q., Pu K., Li Z. (2018). Nat. Commun..

[cit46] Tian Y., Gong Y., Liao Q., Wang Y., Ren J., Fang M., Yang J., Li Z. (2020). Cell Rep. Phys. Sci..

[cit47] Ren J., Wang Y., Tian Y., Liu Z., Xiao X., Yang J., Fang M., Li Z. (2021). Angew. Chem., Int. Ed..

[cit48] Gao M., Wu R., Zhang Y., Meng Y., Fang M., Yang J., Li Z. (2025). J. Am. Chem. Soc..

[cit49] Tian Y., Yang J., Li A., Ren J., Li X., Fang M., Li Z. (2024). ACS Mater. Lett..

[cit50] Ito H., Ozaki K., Itami K. (2017). Angew. Chem., Int. Ed..

[cit51] Zhao Q., Fu W. C., Kwong F.-Y. (2018). Angew. Chem., Int. Ed..

[cit52] Zhou C., Yang X., Gou L., Li B. (2025). Chem. Sci..

[cit53] Guo W. J., Yan S., Chen L., Qiao L., Xu S., Qi T., Liu B., Peng H.-Q. (2024). Adv. Funct. Mater..

[cit54] Sztranyovszky Z., Langbein W., Muljarov E. A. (2023). Phys. Rev. Res..

[cit55] Hu R., Wang K., Liu J., Zhang J., Yang G., Wan L., Li B. (2025). Chin. Chem. Lett..

[cit56] Liu J., Wang K., Wan L., Yang X., Li B. (2025). Chem. Sci..

[cit57] Li P., Deetz A. M., Hu J., Meyer G. J., Hu K. (2022). J. Am. Chem. Soc..

[cit58] Shields B. J., Doyle A. G. (2016). J. Am. Chem. Soc..

[cit59] Troian-Gautier L., Turlington M. D., Wehlin S. A. M., Maurer A. B., Brady M. D., Swords W. B., Meyer G. J. (2019). Chem. Rev..

[cit60] Xu P., Chen P.-Y., Xu H.-C. (2020). Angew. Chem., Int. Ed..

[cit61] Rohe S., Morris A. O., McCallum T., Barriault L. (2018). Angew. Chem., Int. Ed..

[cit62] Deng H.-P., Zhou Q., Wu J. (2018). Angew. Chem., Int. Ed..

[cit63] Cheng Y., Gu X., Li P. (2013). Org. Lett..

[cit64] Halder S., Mandal S., Kundu A., Mandal B., Adhikari D. (2023). J. Am. Chem. Soc..

[cit65] CCDC 2356081: Experimental Crystal Structure Determination, 2026, 10.5517/ccdc.csd.cc2k2pmz

