# Supplementary material for: Annulative π-extension of phenothiazines: access to room temperature ultralong organic phosphorescent materials
Source: Chem Sci. 2026 Jul 6;17(35):16789–97. doi: 10.1039/d6sc04090j (PMC13416836; doi:10.1039/d6sc04090j)
Supplement: Supplementary file 1 [file SC-017-D6SC04090J-s001.pdf]

Supplementary Information (SI) for Chemical Science.
This journal is © The Royal Society of Chemistry 2026

Supporting Information

Annulative π-Extension of Phenothiazines: Access to Room Temperature Ultralong Organic Phosphorescent Materials

Chunlin Zhou[1,#], Shu Zhao[3,#], Lian Gou[1], Danni Yang[1], Haichao Liu[2]*, Bijin Li[1]*

[1]Chongqing Key Laboratory of Natural Product Synthesis and Drug Research, School of Pharmaceutical Sciences, Chongqing University, Chongqing 401331, P. R. China.

[2]State Key Laboratory of Supramolecular Structure and Materials, College of Chemistry, Jilin University, Changchun, Jilin 130012, P. R. China

[3]Department of Medical Oncology, The Second Medical Center and National Clinical Research Center for Geriatric Diseases, Chinese PLA General Hospital, Beijing 100853, China

[#]The authors contribute equally to this work.

*Corresponding author: Haichao Liu (hcliu@jlu.edu.cn), Bijin Li (bijinli@cqu.edu.cn)

Table of contents

1. General Information 3

2. Experimental Section 4

2.1 Optimization of the reaction conditions 4

2.2. General procedures for synthesis of products..7

2.3. Characterization of products..8

2.4. General procedures for the synthesis of substrates..9

3. Photophysical properties of products 17

3.1. Photophysical properties of **4a-4k**, **4n-4o** and **5a**@PMMA films 17

3.2. Photophysical properties of **4a**, **4c-4o** and **5a-5b**@HEA-AA........................29

3.3. UV-Vis absorption spectra of **4a-5b**@PMMA or HEA-AA films................39

3.4. Photophysical properties of **4a**, **4c**, **4d**, **4j**, **4o** and **5a** in solvent. 42

4. Cyclic voltammogram experiment of **4j** 44

5. The thermal properties of **4b** 45

6. The PXRD properties of **4o** crystal and film...45

7. Calculation 46

8. Cell imaging experiments..50

9. The photocatalyzed reaction with **4d** as photosensitizer....................................51

10. References 53

11. X-Ray Crystallographic Spectrum of **4b**..54

12. NMR spectrum of compounds 56

1. General Information

Unless otherwise noted, commercial available reagents were purchased from commercial suppliers (such as Energy Chemical, Bide Pharmatech Ltd. and Adamas) and used as received. Solvents were generally dried over 4Å molecular sieves. Purification of products was performed by flash column chromatography using silica gel. ^{1}H and ^{13}C NMR spectra were recorded on a Agilent 400MR DD2 spectrometer (400 MHz and 101 MHz, respectively). Chemical shifts are reported parts per million (ppm) referenced to $CDCl_3$ (δ 7.26 ppm), tetramethylsilane (TMS, δ 0.00 ppm) for ^{1}H NMR; $CDCl_3$ (δ 77.16 ppm) for ^{13}C NMR. The following abbreviations (or combinations thereof) were used to explain multiplicities: s = singlet, d = doublet, t = triplet, q = quartet, dd = doublet of doublet, td = triplet of doublet and m = multiplet. To distinguish, some ^{13}C NMR chemical shifts retain two decimal places. High-resolution mass spectra (HRMS) were obtained on an Agilent 6546 Q-TOF LC/MS system with an ESI source at the School of Pharmaceutical Sciences of Chongqing University. UV−vis spectra were recorded on Agilent Cary60 spectrometer. Fluorescence spectra were collected on HITACHI F-700003040428 Fluorescence Spectrometer or Agilent Technologies Cary Eclipse Fluorescence Spectrometer. The phosphorescence spectra and absolute quantum yields were taken using Edinburgh Instruments FLS1000 fluorescence spectrometer. The excited-state lifetimes were performed using Edinburgh Instruments FLS1000.

2. Experimental Section

2.1 Optimization of the reaction conditions

The oven-dried Schlenk tube (10 mL) equipped with a stirring bar was charged with substrates **1a** (0.1 mmol, 1.0 equiv), **2a** (0.2 mmol, 2.0 equiv), **3a** (0.15 mmol, 1.5 equiv), $Pd(OAc)_2$ (10 mol%), ligand (25 mol%) and base (3.0-4.5 equiv) in the air. Followed by the mediator smNBE (1.5 equiv) and the anhydrous solvent (0.1 M) was added via syringe under nitrogen flow. Then the tube was evacuated and back-filled with N_2 for three times. Finally, the reaction mixture was stirred at 130 °C for 24-72 h and then cooled to room temperature. Upon completion of the reaction, the mixture was diluted with 5 mL EtOAc and filtered through a short pad of Celite and the Celite pad were washed with an additional EtOAc (10 mL × 5). The filtrate was concentrated *in vacuo* to give the crude product. The crude ^1H NMR spectrum was taken using CH_2Br_2 (0.1 mmol) as internal standard.

Table S1. Screening of the reaction conditions.

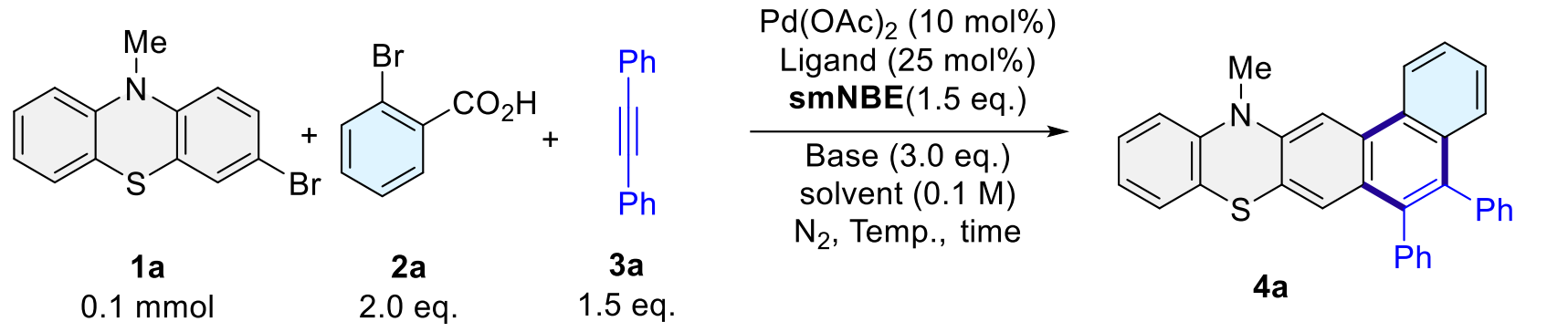

NO.	Ligand (25 mol%)	smNBE (1.5 eq.)	Base (3.0 eq.)	Solvent (0.1M)	Temp.	time	Yield[a] (%)
1	L1	N1	K_2CO_3	DMF	130 °C	24 h	n.d.
2	L1	N2	K_2CO_3	DMF	130 °C	24 h	18
3	L1	N2	K_2CO_3	DMA	130 °C	24 h	15
4	L1	N2	K_2CO_3	toluene	130 °C	24 h	28
5	L1	N2	K_2CO_3	PhCl	130 °C	24 h	22
6	L1	N2	K_2CO_3	dioxane	130 °C	24 h	8
7	L1	N2	K_2CO_3	DCE	130 °C	24 h	5
8	L2	N2	K_2CO_3	toluene	130 °C	24 h	19
9	L3	N2	K_2CO_3	toluene	130 °C	24 h	12
10	L4	N2	K_2CO_3	toluene	130 °C	24 h	21
11	L5	N2	K_2CO_3	toluene	130 °C	24 h	4
[c]12	L6	N2	K_2CO_3	toluene	130 °C	24 h	8
13	L7	N2	K_2CO_3	toluene	130 °C	24 h	35
14	L7	N2	K_2CO_3	toluene	150 °C	24 h	5
15	L7	N2	K_2CO_3	toluene	140 °C	24 h	21
16	L7	N2	K_2CO_3	toluene	120 °C	24 h	8
17	L7	N2	Cs_2CO_3	toluene	130 °C	24 h	12
18	L7	N2	Na_2CO_3	toluene	130 °C	24 h	10
19	L7	N2	K_3PO_4	toluene	130 °C	24 h	18
[d]20	L7	N2	K_2CO_3	toluene	130 °C	24 h	7
21	L7	N2 (1.0 eq.)	K_2CO_3	toluene	130 °C	24 h	15
22	L7	N2 (2.0 eq.)	K_2CO_3	toluene	130 °C	24 h	30
23	L7	N2	K_2CO_3 (4.5 eq.)	toluene	130 °C	24 h	40
24	L7	N2	K_2CO_3 (4.5 eq.)	toluene	130 °C	72 h	54[b]
[e]25	L7	N2	K_2CO_3 (4.5 eq.)	toluene	130 °C	72 h	27
26	L7	N2	-	toluene	130 °C	72 h	n.d.
27	L7	N2	PivOH (3.0 eq.)	toluene	130 °C	72 h	n.d.

[a]Yield was determined by ^1H NMR with CH_2Br_2 (0.1mmol) as internal standard. [b]Isolated yield. [c]12 mol% ligand was used. [d]12.5 mol% ligand was used. [e]In air condition. ligand was used. n.d. = not detected.

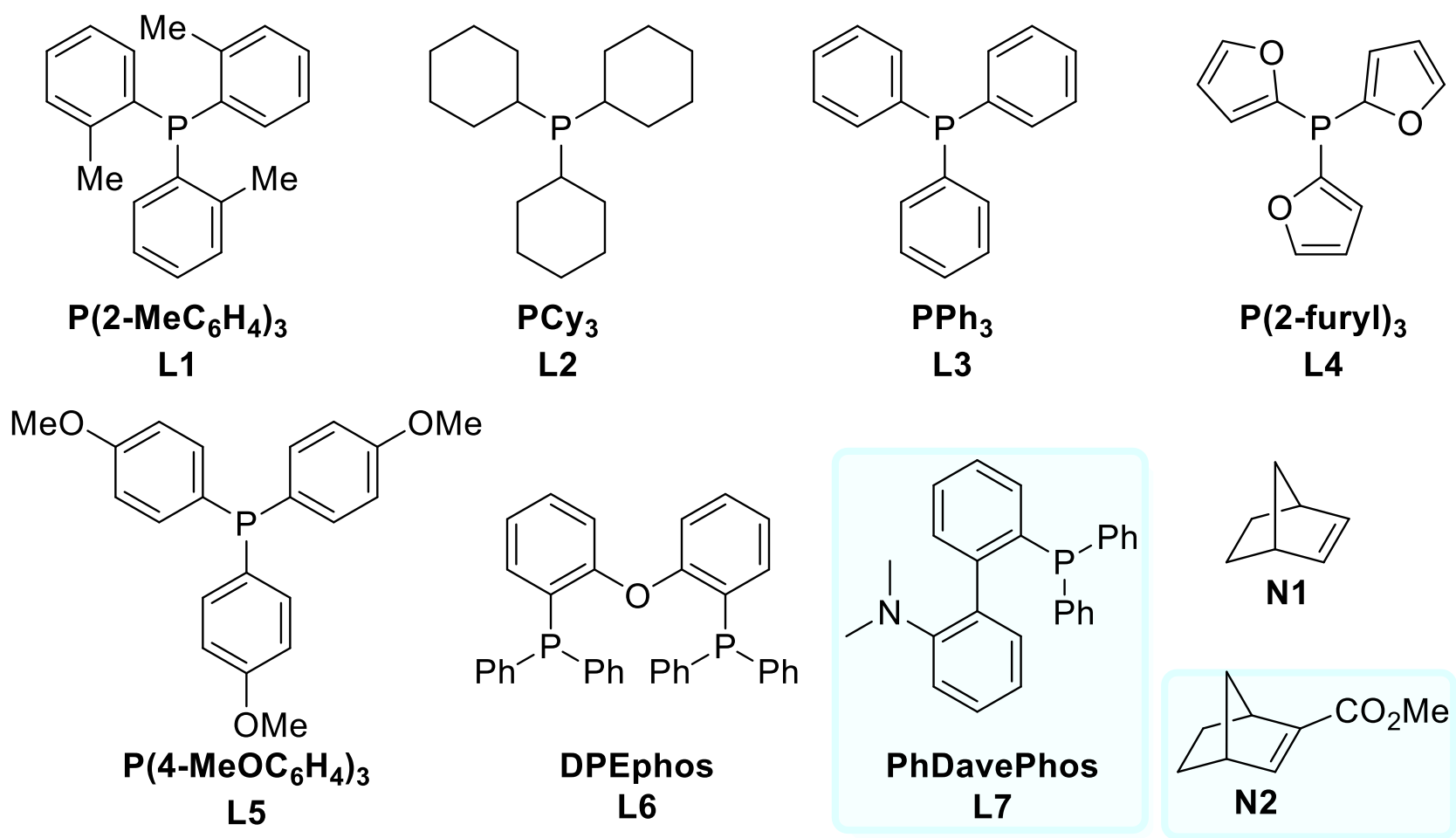

Fig. S1 NBE mediators and ligands.

Reaction mechanism: a possible mechanism pathway is proposed (Fig. S2).

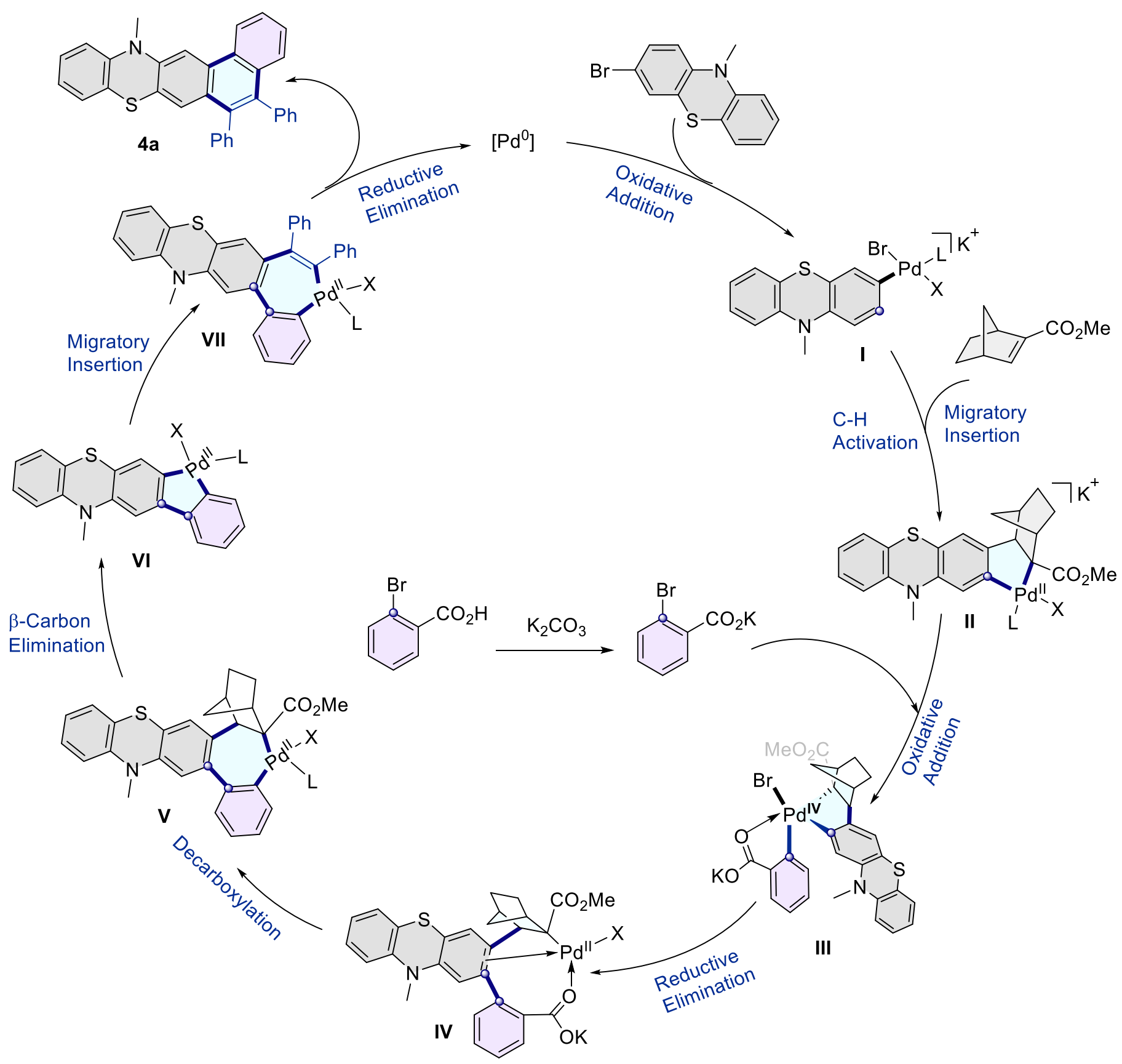

Fig. S2 Proposed reaction mechanism.

2.2. General procedures for synthesis of products

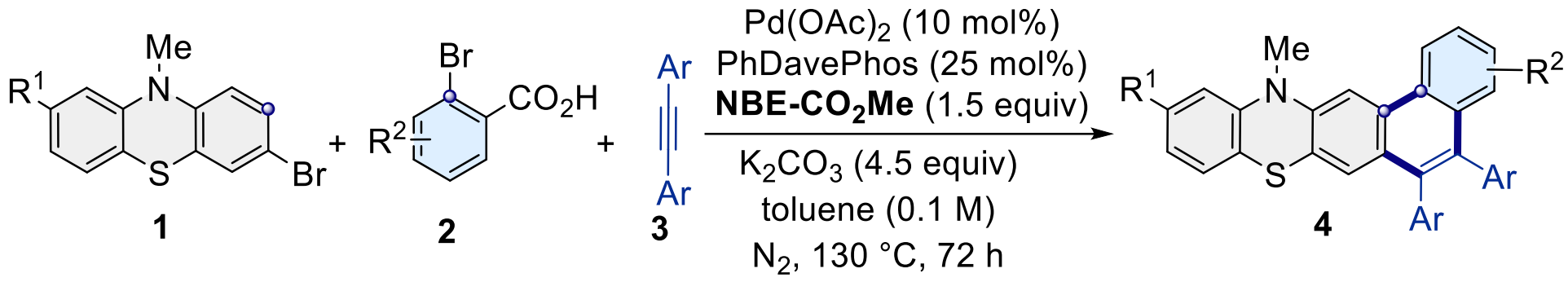

The oven-dried Schlenk tube (10 mL) equipped with a stirring bar was charged with substrates **1** (0.1 mmol, 1.0 equiv), **2** (0.2 mmol, 2.0 equiv), **3** (0.15 mmol, 1.5 equiv), $Pd(OAc)_2$ (10 mol%), PhDavePhos (25 mol%) and K_2CO_3 (4.5 equiv) in the air. Followed by the mediator NBE-CO_2Me (1.5 equiv) and the anhydrous toluene (1.0 mL) were added via syringe under nitrogen flow. Then the tube was evacuated and back-filled with N_2 for three times. Finally, the reaction mixture was stirred at 130 °C for 72 h and then cooled to room temperature. Upon completion of the reaction, the mixture was diluted with 5 mL EtOAc and filtered through a short pad of Celite and the Celite pad were washed with an additional EtOAc (10 mL × 5). The filtrate was concentrated *in vacuo* to give the crude product. The crude ^{1}H NMR spectrum was taken using CH_2Br_2 (0.1 mmol) as internal standard. Finally, the crude product was purified via silica gel column chromatography using PE/EtOAc (50:1-20:1) as the eluent.

2.3. Characterization of products

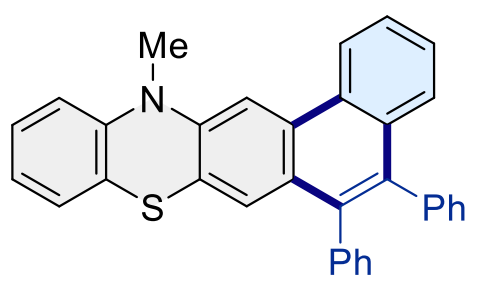

5,6-diphenyl-13-methyl-naphtho[1,2-*b*]phenothiazine (4a)

Yield: 54%, 24.9 mg. ^{1}H NMR (400 MHz, Chloroform-*d*) δ 8.67 (d, J = 8.4 Hz, 1H), 8.03 (s, 1H), 7.62 (t, J = 7.2 Hz, 1H), 7.53 (d, J = 8.0 Hz, 1H), 7.44 (t, J = 7.2 Hz, 1H), 7.30 (s, 1H), 7.27 – 7.15 (m, 7H), 7.12 (t, J = 8.0 Hz, 5H), 6.97 – 6.86 (m, 2H), 3.64 (s, 3H). ^{13}C NMR (101 MHz, Chloroform-*d*) δ 145.3, 144.3, 139.7, 139.4, 136.5, 135.7, 132.3, 131.3, 131.1, 130.0, 129.6, 128.2, 128.1, 127.8, 127.7, 127.3, 126.7, 126.67, 126.5, 126.2, 125.6, 125.1, 123.2, 122.6, 122.3, 114.7, 106.1, 36.0. HRMS (m/z, ESI):

calcd for $C_{33}H_{24}NS^+$ $(M+H)^+$ 466.1551, found 466.1571.

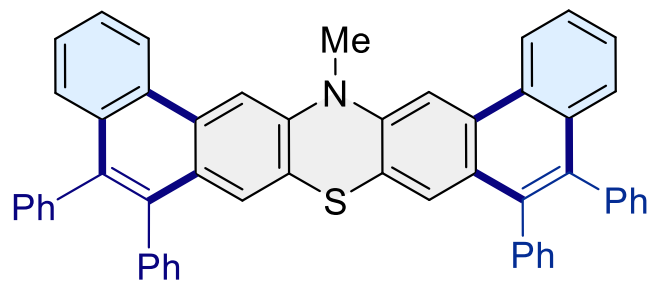

17-methyl-5,6,10,11-tetraphenyl-dinaphtho[1,2-*b*:2′,1′-*i*]phenothiazine (4b)

Note: 0.05 mmol scale of 3,7-dibromo-10-methyl-10*H*-phenothiazine was used as substrate. Yield: 30%, 11.0 mg. ^{1}H NMR (400 MHz, Chloroform-*d*) δ 8.73 (d, J = 8.4 Hz, 2H), 8.16 (s, 2H), 7.66 (t, J = 7.2 Hz, 2H), 7.54 (d, J = 7.6 Hz, 2H), 7.51 – 7.43 (m, 2H), 7.29 (s, 2H), 7.25 – 7.11 (m, 16H), 7.09 (d, J = 6.8 Hz, 4H), 4.05 – 3.85 (m, 3H). ^{13}C NMR (101 MHz, Chloroform-*d*) δ 143.7, 139.6, 139.3, 136.5, 135.8, 132.3, 131.3, 131.1, 130.0, 129.6, 128.2, 128.16, 127.8, 127.7, 126.8, 126.6, 126.3, 125.7, 124.6, 122.3, 106.7, 36.5. HRMS (m/z, ESI): calcd for $C_{53}H_{36}NS^+$ $(M+H)^+$ 718.2490, found 718.2483.

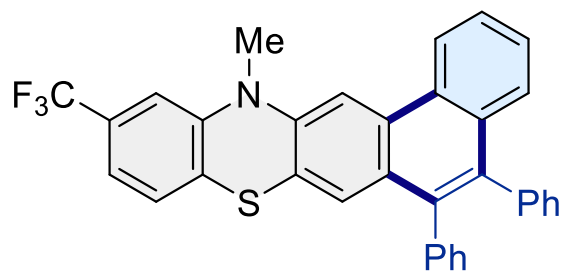

5,6-diphenyl-13-methyl-11-(trifluoromethyl)-naphtho[1,2-*b*]phenothiazine (4c)

Yield: 50%, 24.7 mg. ^{1}H NMR (400 MHz, Chloroform-*d*) δ 8.58 (d, J = 8.4 Hz, 1H), 7.96 (s, 1H), 7.55 (t, J = 7.2 Hz, 1H), 7.45 (d, J = 8.0 Hz, 1H), 7.38 (t, J = 7.2 Hz, 1H), 7.20 (s, 1H), 7.18 – 7.00 (m, 12H), 6.97 (s, 1H), 3.57 (s, 3H). ^{13}C NMR (101 MHz, Chloroform-*d*) δ 145.6, 143.3, 139.5, 139.2, 136.3, 136.1, 132.3, 131.2, 131.1, 130.1, 129.8, 129.5, 128.5, 128.2, 128.1, 127.9, 127.7, 127.2, 126.9, 126.8, 126.6, 126.4, 125.7, 124.3 (q, $J_{C\text{-}F}$ = 273.2 Hz), 123.8, 122.2, 119.2 (q, $J_{C\text{-}F}$ = 3.9 Hz), 111.1 (q, $J_{C\text{-}F}$ = 3.6 Hz), 106.6, 36.0. HRMS (m/z, ESI): calcd for $C_{34}H_{23}F_3NS^+$ $(M+H)^+$ 534.1425, found 534.1424.

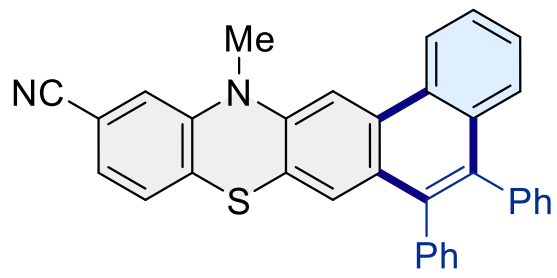

11-cyano-5,6-diphenyl-13-methyl-naphtho[1,2-*b*]phenothiazine (4d)

Yield: 50%, 24.7 mg. ^{1}H NMR (400 MHz, Chloroform-*d*) δ 8.67 (d, J = 8.4 Hz, 1H), 8.05 (s, 1H), 7.65 (t, J = 7.6 Hz, 1H), 7.54 (d, J = 8.4 Hz, 1H), 7.48 (t, J = 7.2 Hz, 1H),

7.28 – 7.16 (m, 8H), 7.15 – 7.07 (m, 5H), 7.05 (s, 1H), 3.63 (s, 3H). ^{13}C NMR (101 MHz, Chloroform-*d*) δ 145.8, 142.8, 139.4, 139.1, 136.4, 136.3, 132.4, 131.2, 131.1, 130.5, 130.2, 129.4, 128.6, 128.2, 127.9, 127.8, 127.5, 127.0, 126.9, 126.7, 126.5, 126.1, 125.8, 123.2, 122.2, 119.1, 117.1, 111.1, 106.9, 36.1. HRMS (m/z, ESI): calcd for $C_{34}H_{23}N_2S^+$ $(M+H)^+$ 491.1504, found 491.1523.

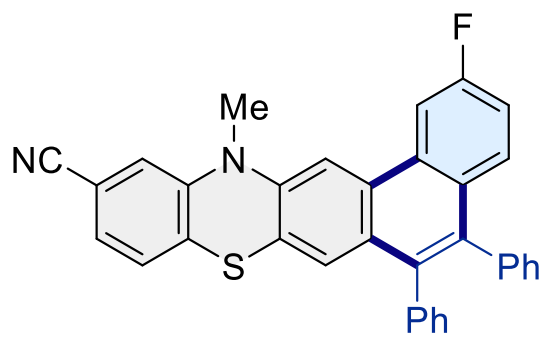

11-cyano-5,6-diphenyl-2-fluoro-13-methyl-naphtho[1,2-*b*]phenothiazine (4e)

Yield: 31%, 15.7 mg. ^{1}H NMR (400 MHz, Chloroform-*d*) δ 8.26 (d, *J* = 10.8 Hz, 1H), 7.87 (s, 1H), 7.53 (dd, *J* = 8.8, 6.0 Hz, 1H), 7.25 – 7.17 (m, 8H), 7.16 (s, 1H), 7.14 – 7.05 (m, 6H), 3.64 (s, 3H). ^{13}C NMR (101 MHz, Chloroform-*d*) δ 161.5 (d, $J_{C\text{-}F}$ = 247.0 Hz), 145.6, 142.8, 139.0 (d, $J_{C\text{-}F}$ = 34.9 Hz), 135.7 (d, $J_{C\text{-}F}$ = 48.2 Hz), 131.1, 131.0, 130.7, 130.6, 130.3, 129.5, 129.1, 128.9, 127.9, 127.83, 127.8, 127.7, 127.5, 126.9, 126.8, 126.2, 125.9, 124.0, 119.1, 117.1, 115.9 (d, $J_{C\text{-}F}$ = 23.5 Hz), 111.1, 107.2 (d, $J_{C\text{-}F}$ = 21.9 Hz), 106.9, 36.1. HRMS (m/z, ESI): calcd for $C_{34}H_{22}FN_2S^+$ $(M+H)^+$ 509.1482, found 509.1480.

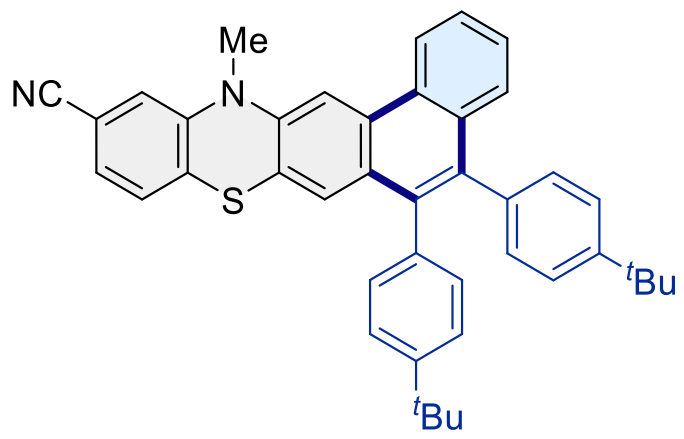

5,6-bis(4-(*tert*-butyl)phenyl)-11-cyano-13-methyl-naphtho[1,2-*b*]phenothiazine (4f)

Yield: 48%, 28.9 mg. ^{1}H NMR (400 MHz, Chloroform-*d*) δ 8.67 (d, *J* = 8.4 Hz, 1H), 8.06 (s, 1H), 7.70 (d, *J* = 8.4 Hz, 1H), 7.64 (t, *J* = 7.2 Hz, 1H), 7.49 (t, *J* = 7.6 Hz, 1H), 7.45 (s, 1H), 7.22 – 7.12 (m, 6H), 7.06 (s, 1H), 6.97 (t, *J* = 8.4 Hz, 4H), 3.64 (s, 3H), 1.27 (s, 9H), 1.26 (s, 9H). ^{13}C NMR (101 MHz, Chloroform-*d*) δ 149.3, 149.2, 145.8, 142.6, 136.8, 136.6, 136.3, 136.0, 132.4, 130.9, 130.7, 130.6, 130.1, 129.3, 128.6, 128.3, 127.5, 126.9, 126.3, 126.0, 125.9, 124.4, 124.3, 123.0, 122.2, 119.2, 117.0, 110.9, 106.9, 36.1, 34.54, 34.5, 31.4. HRMS (m/z, ESI): calcd for $C_{42}H_{39}N_2S^+$ $(M+H)^+$ 603.2829,

found 603.2830.

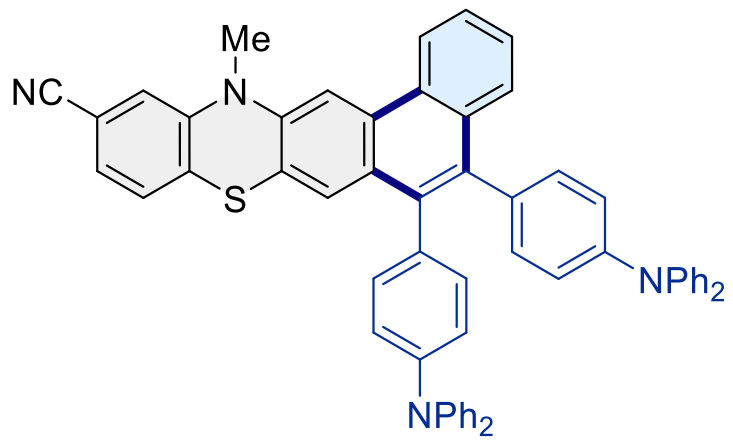

5,6-bis(4-(diphenylamino)phenyl)-11-cyano-13-methyl-naphtho[1,2-*b*]phenothiazine (4g)

Yield: 25%, 20.6 mg. ^{1}H NMR (400 MHz, Chloroform-*d*) δ 8.67 (d, *J* = 8.4 Hz, 1H), 8.06 (s, 1H), 7.79 (d, *J* = 8.4 Hz, 1H), 7.67 (t, *J* = 7.2 Hz, 1H), 7.56 (d, *J* = 7.6 Hz, 1H), 7.53 (s, 1H), 7.31 – 7.20 (m, 7H), 7.15 – 7.06 (m, 10H), 7.05 – 6.93 (m, 14H), 3.65 (s, 3H). ^{13}C NMR (101 MHz, Chloroform-*d*) δ 147.8, 147.76, 147.0, 146.3, 146.2, 145.69, 145.6, 142.6, 136.3, 136.2, 133.7, 133.2, 132.17, 132.1, 132.0, 130.4, 130.1, 129.34, 129.3, 128.4, 128.1, 127.4, 126.9, 126.4, 126.0, 125.7, 124.3, 124.2, 123.1, 122.9, 122.8, 122.7, 122.2, 119.0, 117.0, 110.9, 106.8, 36.0. HRMS (m/z, ESI): calcd for $C_{58}H_{41}N_4S^+$ $(M+H)^+$ 825.3046, found 825.3044.

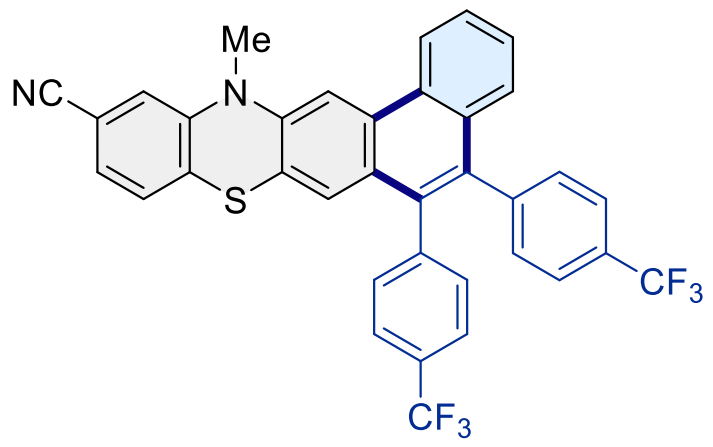

5,6-bis(4-(trifluoromethyl)phenyl)-11-cyano-13-methyl-naphtho[1,2-*b*]phenothiazine (4h)

Yield: 30%, 18.0 mg. ^{1}H NMR (400 MHz, Chloroform-*d*) δ 8.69 (d, *J* = 8.4 Hz, 1H), 8.06 (s, 1H), 7.71 (t, *J* = 7.6 Hz, 1H), 7.52 (t, *J* = 7.6 Hz, 5H), 7.41 (d, *J* = 8.4 Hz, 1H), 7.28 – 7.19 (m, 4H), 7.19 – 7.13 (m, 2H), 7.12 (s, 1H), 7.07 (s, 1H), 3.64 (s, 3H). ^{13}C NMR (101 MHz, Chloroform-*d*) δ 145.5, 143.3, 142.8, 142.5, 135.0, 134.9, 131.5, 131.4, 131.3, 130.5, 130.1, 129.54, 129.5 (q, $J_{C\text{-}F}$ = 17.5 Hz), 129.2 (q, $J_{C\text{-}F}$ = 17.3 Hz), 127.8, 127.7, 127.6, 127.4, 127.2, 126.3, 125.3, 125.2 (q, $J_{C\text{-}F}$ = 3.8 Hz), 125.0 (q, $J_{C\text{-}F}$ = 3.9 Hz), 124.2 (q, $J_{C\text{-}F}$ = 273.2 Hz), 124.1 (q, $J_{C\text{-}F}$ = 273.9 Hz), 123.8, 122.5, 119.0, 117.2, 111.2, 106.9, 36.1. HRMS (m/z, ESI): calcd for $C_{36}H_{21}F_6N_2S^+$ $(M+H)^+$ 627.1324, found 627.1311.

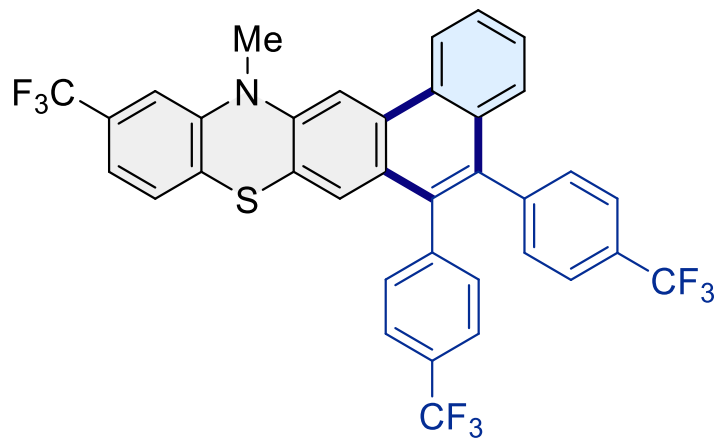

5,6-bis(4-(trifluoromethyl)phenyl)-13-methyl-11-(trifluoromethyl)-naphtho[1,2-*b*]phenothiazine (4i)

Yield: 28%, 19.0 mg. ^{1}H NMR (400 MHz, Chloroform-*d*) δ 8.70 (d, *J* = 8.4 Hz, 1H), 8.07 (s, 1H), 7.70 (t, *J* = 7.2 Hz, 1H), 7.58 – 7.48 (m, 6H), 7.41 (d, *J* = 8.4 Hz, 1H), 7.25 – 7.17 (m, 5H), 7.14 (s, 1H), 7.09 (s, 1H), 3.69 (s, 3H). ^{13}C NMR (101 MHz, Chloroform-*d*) δ 145.4, 143.9, 142.9, 142.6, 135.0, 134.7, 131.5, 131.3, 130.4, 129.6, 129.4, 129.2, 127.8, 127.6, 127.3, 127.1, 125.22, 125.2, 125.16, 125.14, 125.1, 125.08, 125.0, 125.04, 125.0, 124.98, 124.49, 122.47 124.2 (q, $J_{C\text{-}F}$ = 273.3 Hz), 121.6, 119.5 (q, $J_{C\text{-}F}$ = 4.0 Hz), 111.3 (q, $J_{C\text{-}F}$ = 3.3 Hz), 106.7, 36.1. HRMS (m/z, ESI): calcd for $C_{36}H_{21}F_9NS^+$ $(M+H)^+$ 670.1246, found 670.1250.

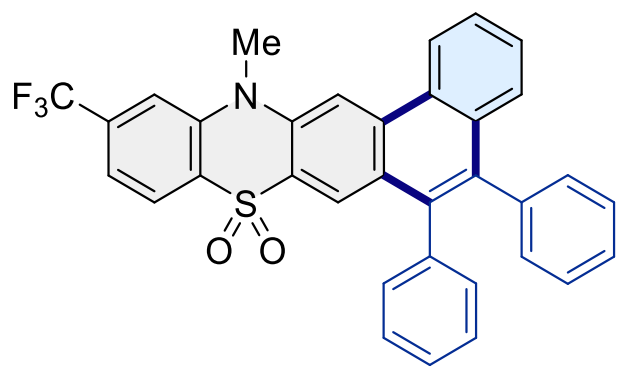

5,6-diphenyl-13-methyl-11-(trifluoromethyl)-naphtho[1,2-*b*]phenothiazine-*S,S*-dioxide (4j)

Yield: 59%, 33.7 mg. ^{1}H NMR (400 MHz, Chloroform-*d*) δ 8.79 (d, *J* = 8.4 Hz, 1H), 8.59 (s, 1H), 8.37 (s, 1H), 8.19 (d, *J* = 8.0 Hz, 1H), 7.71 (t, *J* = 6.8 Hz, 1H), 7.65 – 7.54 (m, 3H), 7.49 (d, *J* = 8.0 Hz, 1H), 7.31 – 7.18 (m, 6H), 7.18 – 7.07 (m, 4H), 3.98 (s, 3H). ^{13}C NMR (101 MHz, Chloroform-*d*) δ 143.0, 139.4, 138.8, 138.0, 137.4, 137.0, 135.1 (q, $J_{C\text{-}F}$ = 33.0 Hz), 133.8, 133.6, 131.0, 128.8, 128.5 (q, $J_{C\text{-}F}$ = 6.7 Hz), 128.1, 127.9, 127.5, 127.47, 127.4, 126.9, 125.1, 124.9, 124.8, 124.34, 124.3, 123.3 (q, $J_{C\text{-}F}$ = 274.7 Hz), 123.1, 123.06, 118.2 (q, $J_{C\text{-}F}$ = 6.5 Hz), 113.1 (q, $J_{C\text{-}F}$ = 4.7 Hz), 108.6, 36.5. HRMS (m/z, ESI): calcd for $C_{34}H_{23}F_3NO_2S^+$ $(M+H)^+$ 566.1396, found 566.1398.

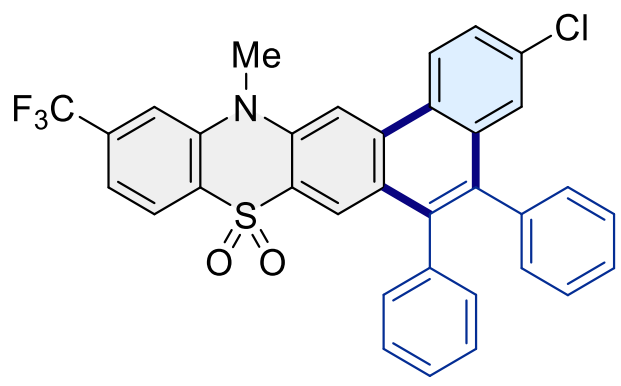

3-chloro-5,6-diphenyl-13-methyl-11-(trifluoromethyl)-naphtho[1,2-*b*]phenothiazine-*S,S*-dioxide (4k)

Yield: 51%, 30.6 mg. ^{1}H NMR (400 MHz, Chloroform-*d*) δ 8.71 (d, *J* = 8.8 Hz, 1H), 8.51 (s, 1H), 8.35 (s, 1H), 8.19 (d, *J* = 8.0 Hz, 1H), 7.65 (d, *J* = 8.8 Hz, 1H), 7.62 – 7.55 (m, 2H), 7.51 (d, *J* = 8.0 Hz, 1H), 7.31 – 7.19 (m, 6H), 7.16 – 7.05 (m, 4H), 3.99 (s, 3H). ^{13}C NMR (101 MHz, Chloroform-*d*) δ 143.0, 139.7, 138.4, 138.1, 137.7, 136.5, 135.3 (q, $J_{C\text{-}F}$ = 32.2 Hz), 134.9, 134.8, 133.3, 130.9, 130.8, 128.2, 128.1, 127.6, 127.5, 127.4, 127.3, 127.2, 125.4, 124.9, 124.8, 124.6, 123.3 (q, $J_{C\text{-}F}$ = 274.5 Hz), 118.4 (q, $J_{C\text{-}F}$ = 3.2Hz), 113.2 (q, $J_{C\text{-}F}$ = 4.2 Hz), 109.6, 108.5, 104.2, 36.5. HRMS (m/z, ESI): calcd for $C_{34}H_{22}ClF_3NO_2S^+$ $(M+H)^+$ 600.1007, found 600.1012.

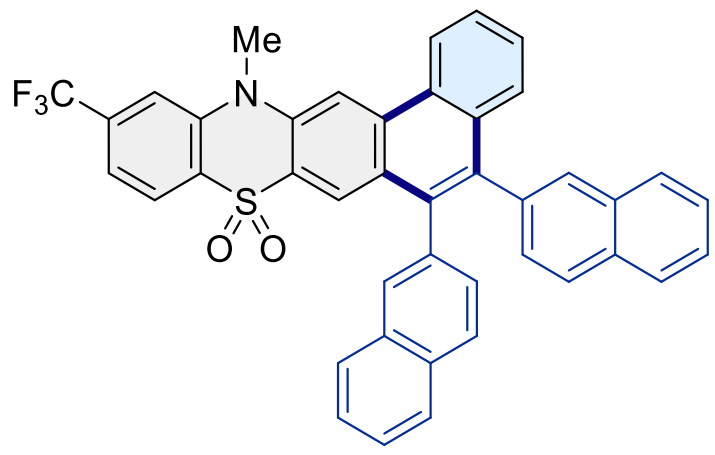

5,6-di(naphthalen-2-yl)-13-methyl-11-(trifluoromethyl)-naphtho[1,2-*b*]phenothiazine-*S,S*-dioxide (4l)

Yield: 65%, 43.2 mg. ^{1}H NMR (400 MHz, Chloroform-*d*) δ 8.81 (d, *J* = 8.4 Hz, 1H), 8.57 (s, 1H), 8.36 (s, 1H), 8.12 (d, *J* = 8.4 Hz, 1H), 7.75 – 7.62 (m, 9H), 7.59 – 7.49 (m, 3H), 7.45 (d, *J* = 8.0 Hz, 1H), 7.38 – 7.27 (m, 6H), 3.87 (s, 3H). ^{13}C NMR (101 MHz, Chloroform-*d*) δ 143.0, 139.5, 137.6, 137.5, 137.14, 137.1, 136.4, 136.3, 135.6, 135.5, 135.1 (q, $J_{C\text{-}F}$ = 28.6 Hz), 133.9, 133.8, 133.0, 132.4, 132.2, 130.3, 130.2, 129.9, 129.8, 129.1, 128.9, 128.8, 128.7, 128.6, 128.0, 127.9, 127.8, 127.7, 127.6, 127.5, 127.1, 126.2, 125.2, 124.9, 124.3, 123.4 (q, $J_{C\text{-}F}$ = 274.9 Hz), 123.2, 118.2 (q, $J_{C\text{-}F}$ = 4.2 Hz), 113.1 (q, $J_{C\text{-}F}$ = 5.4 Hz), 108.7, 36.4. HRMS (m/z, ESI): calcd for $C_{42}H_{27}F_3NO_2S^+$ $(M+H)^+$ 666.1710, found 666.1702.

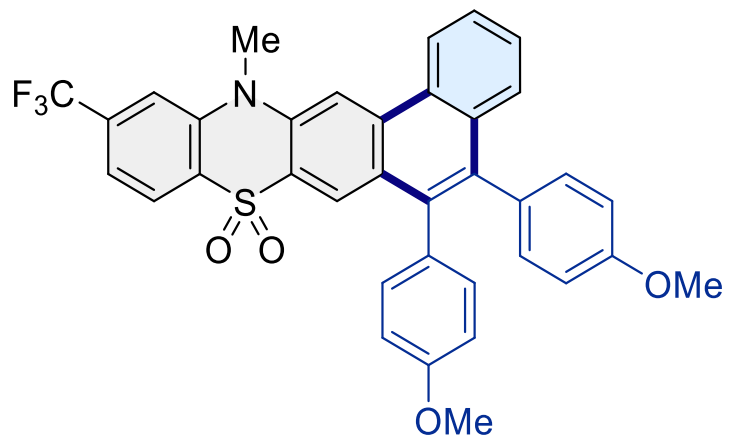

5,6-bis(4-methoxyphenyl)-13-methyl-11-(trifluoromethyl)-naphtho[1,2-*b*]phenothiazine-*S,S*-dioxide (4m)

Yield: 42%, 26.3 mg. ^{1}H NMR (400 MHz, Chloroform-*d*) δ 8.78 (d, J = 8.4 Hz, 1H), 8.58 (s, 1H), 8.40 (s, 1H), 8.20 (d, J = 8.0 Hz, 1H), 7.71 (t, J = 7.2 Hz, 1H), 7.65 (d, J = 8.0 Hz, 1H), 7.62 – 7.54 (m, 2H), 7.50 (d, J = 8.0 Hz, 1H), 7.09 – 6.99 (m, 4H), 6.87 – 6.76 (m, 4H), 4.01 (s, 3H), 3.82 (s, 3H), 3.80 (s, 3H). ^{13}C NMR (101 MHz, Chloroform-*d*) δ 158.6, 158.3, 143.1, 139.3, 137.4, 137.1, 135.2 (q, $J_{C\text{-}F}$ = 32.8 Hz), 134.6, 134.1, 133.8, 132.1, 131.2, 130.4, 128.8, 128.5, 128.1, 127.6, 126.8, 125.1, 124.9, 124.4, 123.4 (q, $J_{C\text{-}F}$ = 274.5 Hz), 123.0, 118.2 (q, $J_{C\text{-}F}$ = 3.7 Hz), 113.7, 113.4, 113.1 (q, $J_{C\text{-}F}$ = 4.2 Hz), 108.5, 55.3, 36.5. HRMS (m/z, ESI): calcd for $C_{36}H_{27}F_3NO_4S^+$ $(M+H)^+$ 626.1608, found 626.1601.

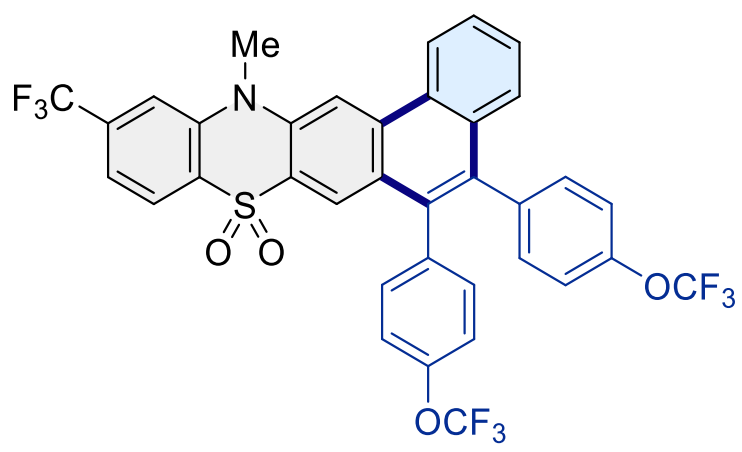

5,6-bis(4-(trifluoromethoxy)phenyl)-13-methyl-11-(trifluoromethyl)-naphtho[1,2-*b*]phenothiazine-*S,S*-dioxide (4n)

Yield: 57%, 42.0 mg. ^{1}H NMR (400 MHz, Chloroform-*d*) δ 8.82 (d, J = 8.0 Hz, 1H), 8.62 (s, 1H), 8.34 (s, 1H), 8.21 (d, J = 8.0 Hz, 1H), 7.78 (t, J = 6.8 Hz, 1H), 7.69 – 7.57 (m, 3H), 7.53 (d, J = 8.0 Hz, 1H), 7.20 – 7.06 (m, 8H), 4.03 (s, 3H). ^{13}C NMR (101 MHz, Chloroform-*d*) δ 148.6, 148.3, 143.0, 139.8, 137.3, 136.6, 136.5, 136.1, 135.4 (q, $J_{C\text{-}F}$ = 33.0 Hz), 134.0, 133.0, 132.45, 132.4, 129.0, 128.98, 128.3, 127.6, 127.5, 126.9, 125.5, 125.0, 124.0, 123.3 (q, $J_{C\text{-}F}$ = 274.5 Hz), 123.28, 120.8, 120.65, 120.6 (q, $J_{C\text{-}F}$ = 258.7 Hz), 118.5 (q, $J_{C\text{-}F}$ = 3.4 Hz), 113.2 (q, $J_{C\text{-}F}$ = 3.6 Hz), 108.8, 36.6. HRMS (m/z, ESI): calcd for $C_{36}H_{21}F_9NO_4S^+$ $(M+H)^+$ 734.1043, found 734.1072.

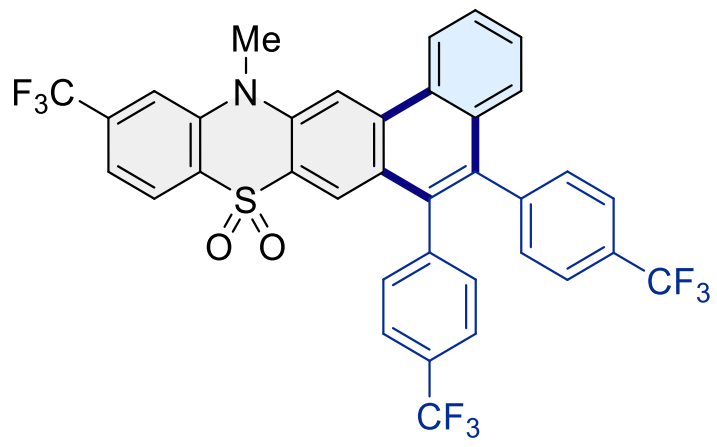

5,6-bis(4-(trifluoromethyl)phenyl)-13-methyl-11-(trifluoromethyl)-naphtho[1,2-*b*]phenothiazine-*S,S*-dioxide (4o)

Yield: 60%, 42.0 mg. ^{1}H NMR (400 MHz, Chloroform-*d*) δ 8.84 (d, J = 8.4 Hz, 1H), 8.63 (s, 1H), 8.25 (s, 1H), 8.20 (d, J = 8.0 Hz, 1H), 7.79 (t, J = 7.2 Hz, 1H), 7.68 – 7.60 (m, 2H), 7.60 – 7.46 (m, 6H), 7.31 – 7.23 (m, 4H), 4.03 (s, 3H). ^{13}C NMR (101 MHz, Chloroform-*d*) δ 143.0, 142.3, 141.5, 139.9, 136.3, 135.7, 135.4 (q, J_{C-F} = 33.1 Hz), 134.0, 132.8, 131.35, 131.3, 130.0 (q, J_{C-F} = 36.4 Hz), 129.6 (q, J_{C-F} = 36.2 Hz), 129.1, 129.0, 128.2, 127.7, 127.5, 126.6, 125.5, 125.4 (q, J_{C-F} = 4.1 Hz), 125.2 (q, J_{C-F} = 3.8 Hz), 125.0, 123.9, 123.34, 123.3 (q, J_{C-F} = 274.8 Hz), 121.2 (q, J_{C-F} = 229.1 Hz), 118.6 (q, J_{C-F} = 3.9 Hz), 113.3 (q, J_{C-F} = 3.9 Hz), 108.8, 36.6. HRMS (m/z, ESI): calcd for $C_{36}H_{21}F_9NO_2S^+$ $(M+H)^+$ 702.1144, found 702.1113.

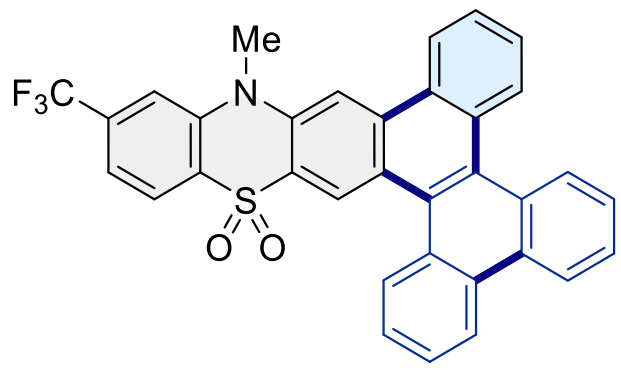

10-methyl-12-(trifluoromethyl)-benzo[11,12]chryseno[5,6-*b*]phenothiazine-*S,S*-dioxide (5a)

Note: 0.05 mmol scale of **4j** was used. Yield: 44%, 12.3 mg. ^{1}H NMR (400 MHz, Chloroform-*d*) δ 9.27 (s, 1H), 8.70 – 8.60 (m, 1H), 8.60 – 8.42 (m, 3H), 8.42 – 8.31 (m, 2H), 8.28 (dd, J = 8.0, 2.4 Hz, 2H), 7.72 – 7.32 (m, 8H). ^{13}C NMR (101 MHz, Chloroform-*d*) δ 142.8, 139.3, 134.8, 131.07, 131.05, 130.7, 129.4, 129.1, 128.7, 128.6, 128.4, 128.34, 128.3, 128.2, 127.6, 127.4, 127.2, 127.16, 127.1, 127.07, 126.9, 126.8, 125.4 (q, J_{C-F} = 239.1 Hz), 125.0, 124.9, 124.5, 124.4, 124.2, 123.8 (q, J_{C-F} = 2.5 Hz), 123.5, 118.4 (q, J_{C-F} = 3.9 Hz), 113.1 (q, J_{C-F} = 3.3 Hz), 109.7, 36.4. HRMS (m/z, ESI): calcd for $C_{34}H_{21}F_3NO_2S^+$ $(M+H)^+$ 564.1240, found 564.1229.

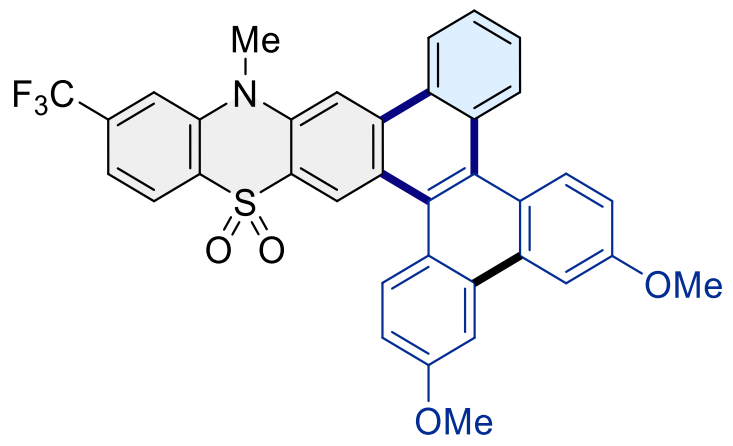

2,19-dimethoxy-10-methyl-12-(trifluoromethyl)-benzo[11,12]chryseno[5,6-*b*]phenothiazine-*S,S*-dioxide (5b)

Note: The solubility of **5b** is poor in deuterated solvent. 0.05 mmol scale of **4m** was used, Yield: 50%, 15.6 mg. ^{1}H NMR (400 MHz, Chloroform-*d*) δ 9.16 (s, 1H), 8.82 (s, 1H), 8.63 – 8.54 (m, 2H), 8.46 (d, J = 8.4 Hz, 1H), 8.43 – 8.32 (m, 2H), 8.26 (d, J = 7.6 Hz, 2H), 7.99 (s, 1H), 7.66 (d, J = 7.6 Hz, 1H), 7.61 (s, 2H), 7.54 (d, J = 8.8 Hz, 2H), 4.09 (s, 3H), 4.00 (s, 6H). HRMS (m/z, ESI): calcd for $C_{36}H_{25}F_3NO_4S$ $(M+H)^+$ 624.1451, found 624.1401.

2.4. General procedures for the synthesis of substrates

(a) The synthesis of bromo-substituted phenothiazine derivatives:

The bromo-subsittuted phenothiazine derivatives were prepared according to the reported literatures and the NMR spectrum of substrates **1a** and **1b** are accordance with the reported literatures [S1, S2].

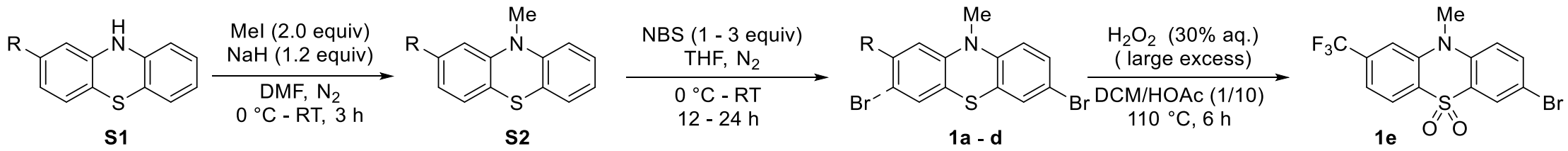

Characterization of substrates 1c and 1d:

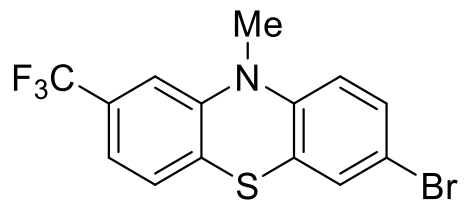

7-bromo-10-methyl-2-(trifluoromethyl)-phenothiazine (1c): ^{1}H NMR (400 MHz, Chloroform-*d*) δ 7.27 (dd, J = 8.4, 2.0 Hz, 1H), 7.22 (d, J = 1.6 Hz, 1H), 7.18 (s, 2H), 6.95 (s, 1H), 6.66 (d, J = 8.8 Hz, 1H), 3.36 (s, 3H). ^{13}C NMR (101 MHz, Chloroform-*d*) δ 145.9, 144.3, 130.6, 130.1 (q, J_{C-F} = 32.5 Hz), 129.6, 127.7, 127.4, 124.7, 124.2 (q, J_{C-F} = 272.2 Hz), 119.5 (q, J_{C-F} = 4.0 Hz), 115.7, 115.5, 110.7 (q, J_{C-F} = 3.7 Hz), 35.6. HRMS (m/z, ESI): calcd for $C_{14}H_{10}BrF_3NS^+$ $(M+H)^+$ 359.9591, found 359.9605.

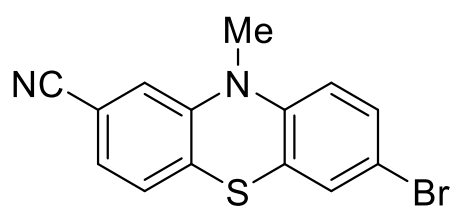

7-bromo-2-cyano-10-methyl-phenothiazine (1d): ^{1}H NMR (400 MHz, Chloroform-*d*) δ 7.29 (dd, *J* = 8.8, 2.4 Hz, 1H), 7.20 (dd, *J* = 10.0, 2.0 Hz, 2H), 7.14 (d, *J* = 8.0 Hz, 1H), 6.94 (s, 1H), 6.66 (d, *J* = 8.4 Hz, 1H), 3.33 (s, 3H). ^{13}C NMR (101 MHz, Chloroform-*d*) δ 146.0, 143.8, 130.9, 130.0, 129.6, 127.6, 126.4, 124.2, 118.9, 116.7, 115.9, 115.8, 111.3, 35.6. HRMS (m/z, ESI): calcd for $C_{14}H_{10}BrN_2S^+$ $(M+H)^+$ 316.9670, found 316.9695.

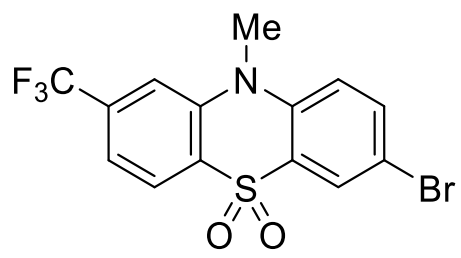

7-bromo-10-methyl-2-(trifluoromethyl)-phenothiazine-*S,S*-dioxide (1e): ^{1}H NMR (400 MHz, Chloroform-*d*) δ 8.25 – 8.17 (m, 2H), 7.75 (dd, *J* = 9.2, 2.4 Hz, 1H), 7.60 – 7.51 (m, 2H), 7.24 (d, *J* = 9.2 Hz, 1H), 3.76 (s, 3H). ^{13}C NMR (101 MHz, Chloroform-*d*) δ 142.2, 140.9, 136.6, 135.3 (q, $J_{C\text{-}F}$ = 32.9 Hz), 127.0, 126.4, 125.8, 124.9, 123.2 (q, $J_{C\text{-}F}$ = 274.4 Hz), 118.8 (q, $J_{C\text{-}F}$ = 3.6 Hz), 117.9, 115.3, 113.2 (q, $J_{C\text{-}F}$ = 4.2 Hz), 36.3. HRMS (m/z, ESI): calcd for $C_{14}H_{10}BrF_3NO_2S^+$ $(M+H)^+$ 391.9562, found 391.9565.

(b) The synthesis of symmetrical 1,2-diarylalkynes:

All the symmetrical 1,2-diarylalkynes were prepared according to the reported literatures[S3].

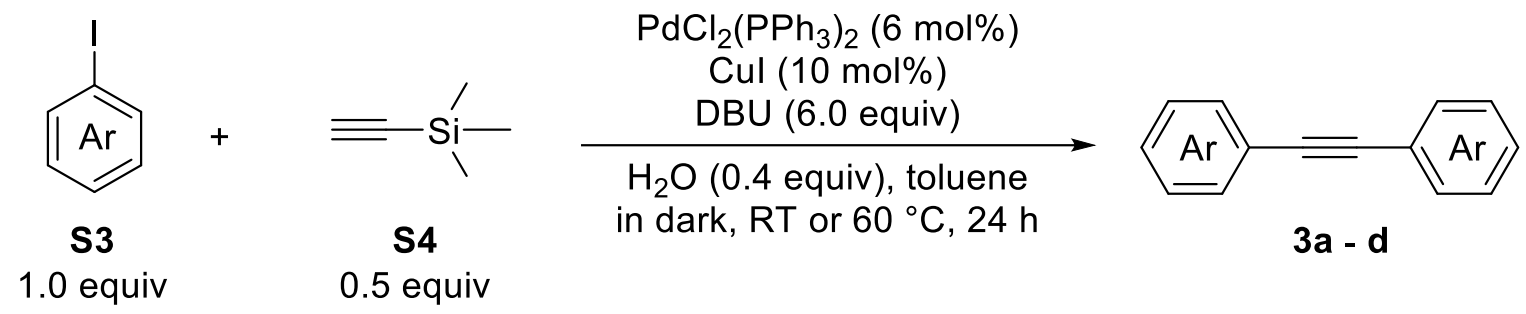

(c) The synthesis of norbornene mediators:

The structurally modified norbornenes were synthesized following the procedure as described in the literature [S4].

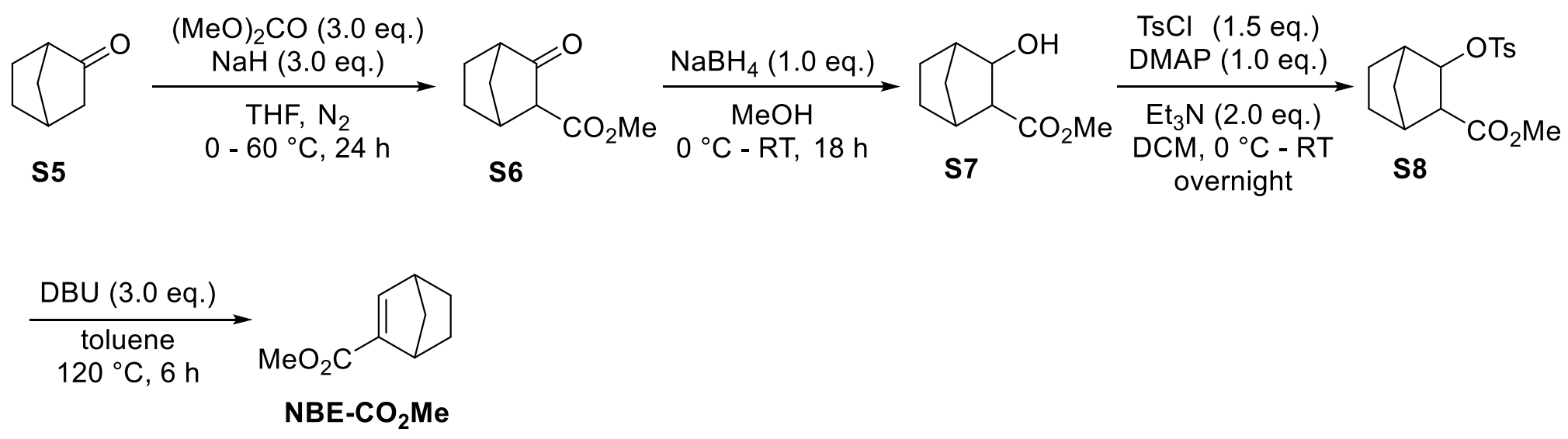

3. Photophysical properties of products

3.1. Photophysical properties of 4a-4k, 4n-4o and 5a@PMMA films

General procedures for preparation of PMMA film samples: PMMA (100 mg) and product (**4a-4m**, 1.0 mg) were mixed with DCM (1.0 mL) in a 5.0 mL sample bottle. The mixture was ultrasonicated at room temperature until the PMMA particles completely dissolved and then the mixture was dropped onto a glass substrate. After evaporation of the solvent, the films were placed in a drying oven at 50 °C for 6 h.

Fluorescence spectra were collected on HITACHI F-700003040428 fluorescence spectrometer and Edinburgh FLS1000. The phosphorescence spectra, excited-state lifetimes and absolute quantum yields were taken using Edinburgh Instruments FLS1000 fluorescence spectrometer. In a typical experiment, a PMMA film doped with desired phenothiazine-besed polycyclic aromatic hydrocarbons for the collection of emission spectrum, excited-state lifetimes and absolute quantum yields were prepared respectively.

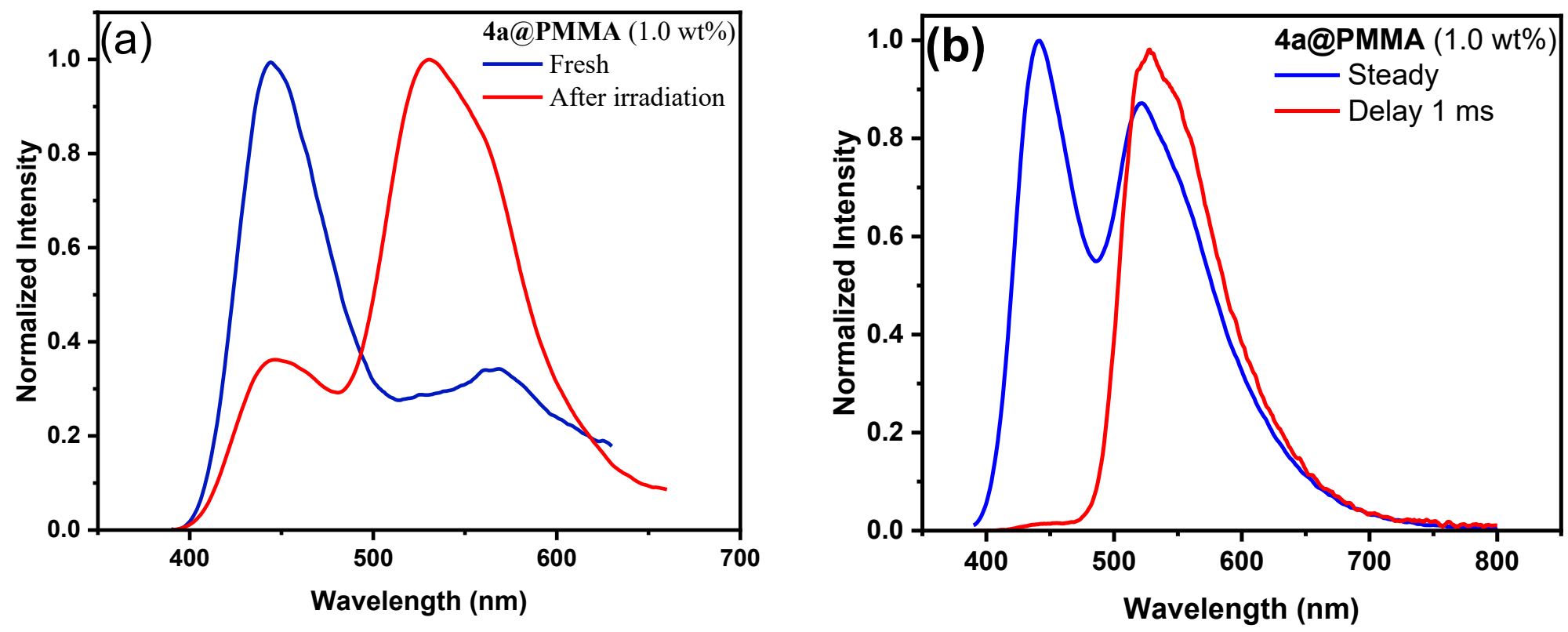

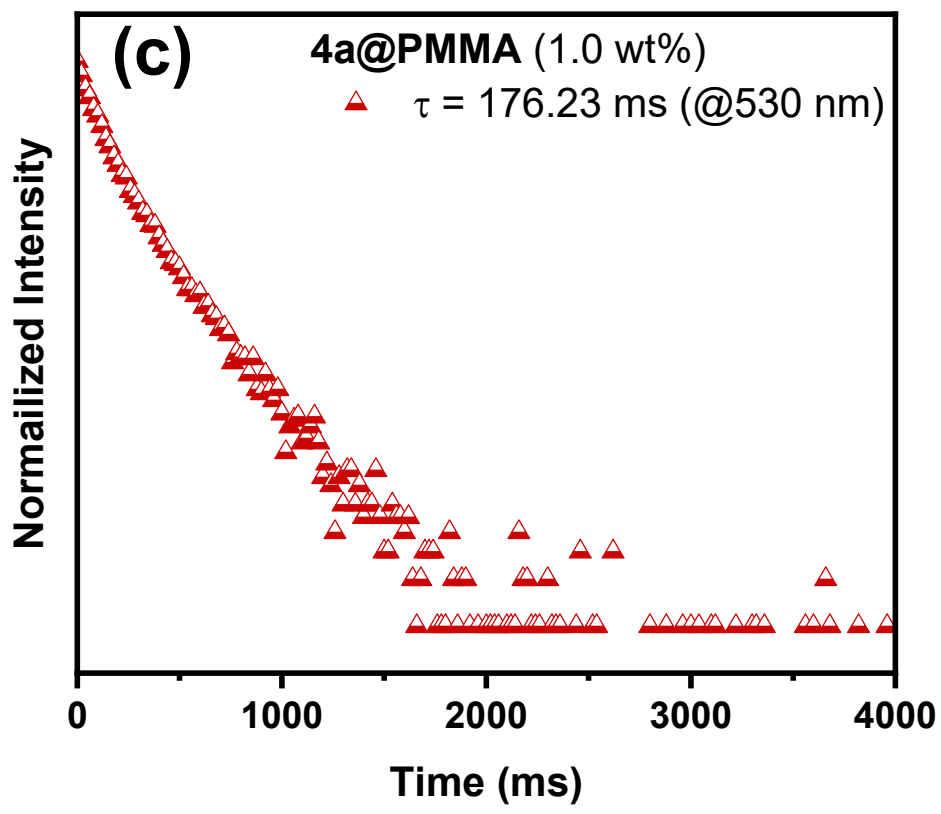

Fig. S3 (a) Steady state fluorescence (FL) spectra of **4a@PMMA** (1.0 wt%) before and after UV irradiation. (b) Steady-state FL and delayed phosphorescence (PL) spectra of **4a@PMMA** (1.0 wt%). (c) The time resolved PL-decay curve for phosphorescence at 530 nm of **4a@PMMA**. (d) The time resolved FL-decay curve for fluorescence at 440 nm of **4a@PMMA**. Fluorescence Spectrometer: Edinburgh FLS1000 and HITACHI F-7000. λ_{ex} = 365 nm. Irradiation condition: UV 365 nm, 30 s.

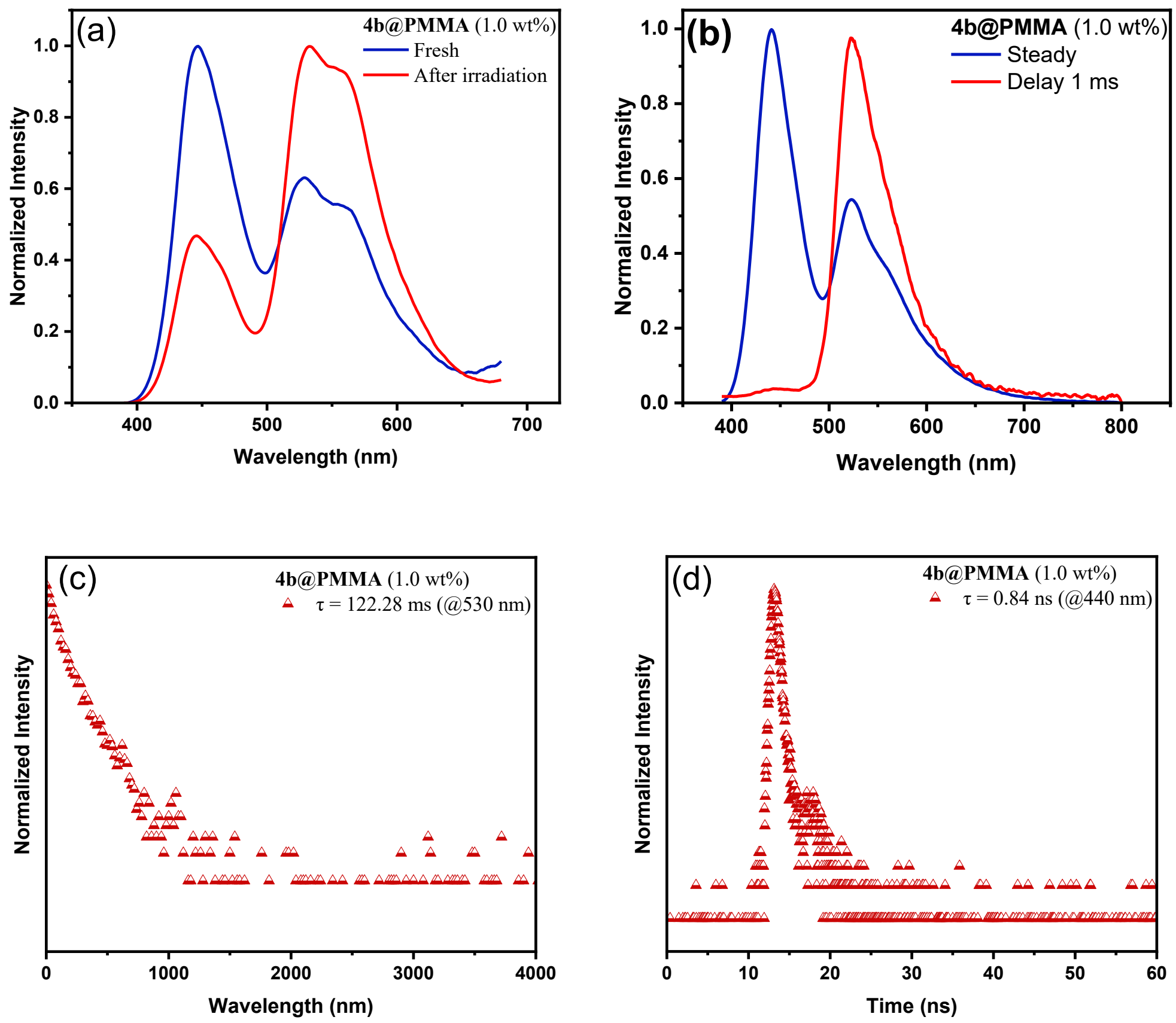

Fig. S4 (a) Steady state fluorescence (FL) spectra of **4b@PMMA** (1.0 wt%) before and after UV irradiation. (b) Steady-state FL and delayed PL spectra of **4b@PMMA** (1.0

wt%). (c) The time resolved PL-decay curve for phosphorescence at 530 nm of **4b@PMMA**. (d) The time resolved FL-decay curve for fluorescence at 440 nm of **4b@PMMA**. Fluorescence Spectrometer: Edinburgh FLS1000 and HITACHI F-7000. λ_{ex} = 365 nm. Irradiation condition: UV 365 nm, 30 s.

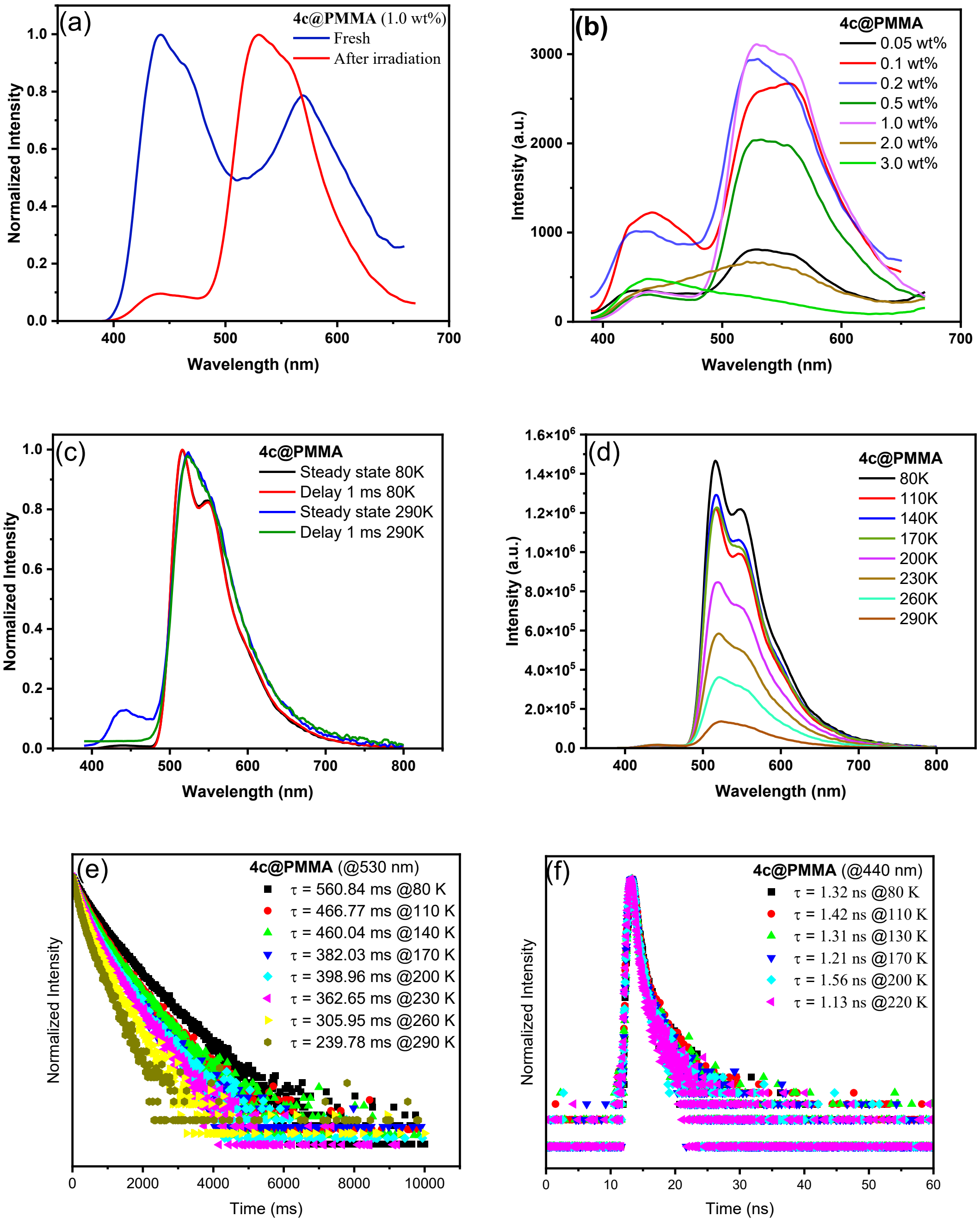

Fig. S5 (a) Steady state fluorescence (FL) spectra of **4c@PMMA** (1.0 wt%) before and after UV irradiation. (b) Steady state FL spectra of **4c@PMMA** with different concentrations after UV irradiation. (c) Steady-state FL and delayed PL spectra of **4c@PMMA** (1.0 wt%) at 80K and 290K. (d) Steady state PL of **4c@PMMA** (1.0 wt%)

at different temperature. (e) The time resolved PL-decay curve for phosphorescence at 530 nm of **4c@PMMA** (1.0 wt%) at different temperature. (f) The time resolved FL-decay curve for fluorescence at 440 nm of **4c@PMMA** (1.0 wt%) at different temperature. Fluorescence Spectrometer: Edinburgh FLS1000 and HITACHI F-7000. λ_{ex} = 365 nm. Irradiation condition: UV 365 nm, 30 s.

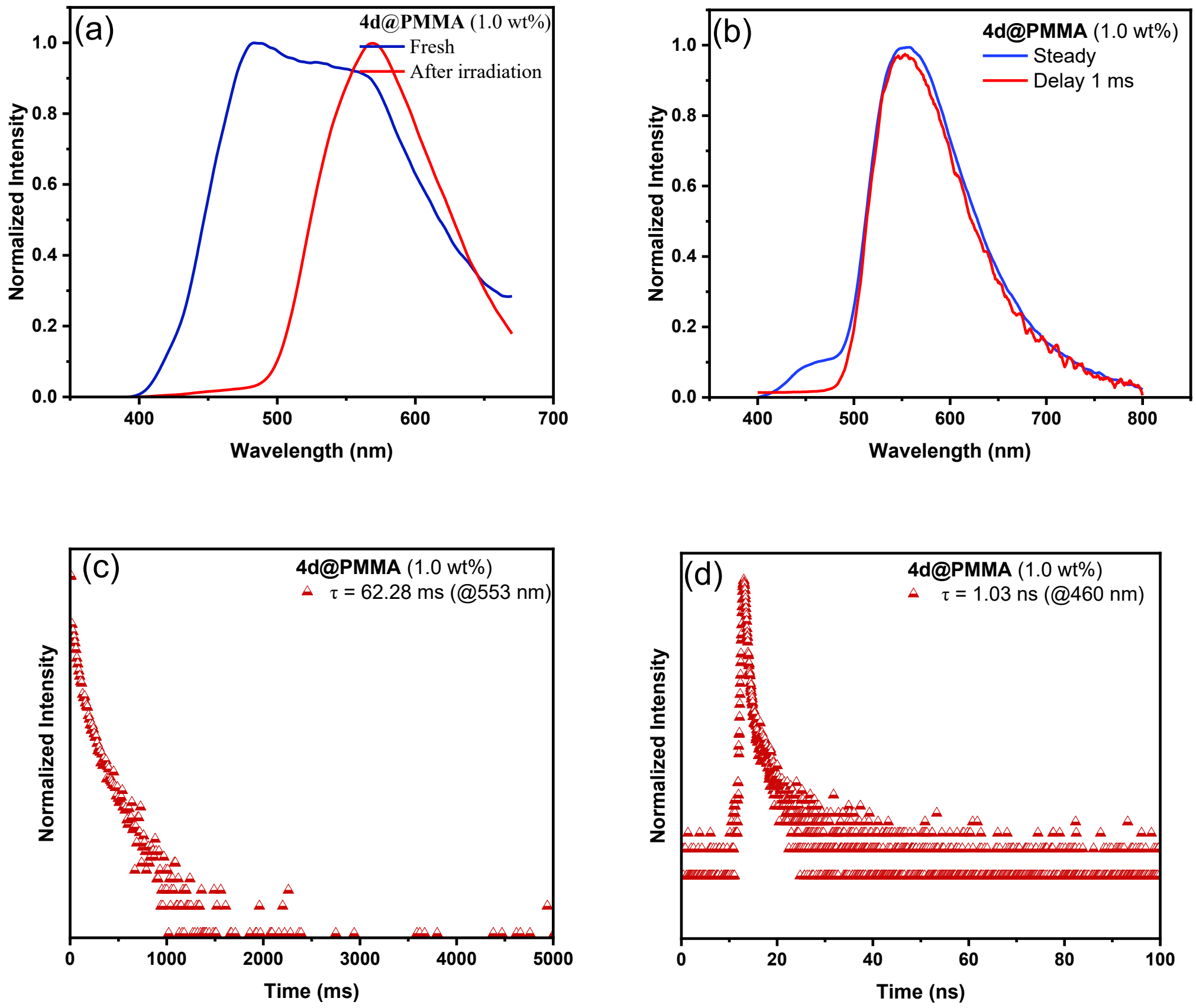

Fig. S6 (a) Steady state fluorescence (FL) spectra of **4d@PMMA** (1.0 wt%) before and after UV irradiation. (b) Steady-state FL and delayed PL spectra of **4d@PMMA** (1.0 wt%). (c) The time resolved PL-decay curve for phosphorescence at 553 nm of **4d@PMMA**. (d) The time resolved FL-decay curve for fluorescence at 460 nm of **4d@PMMA**. Fluorescence Spectrometer: Edinburgh FLS1000 and HITACHI F-7000. λ_{ex} = 365 nm. Irradiation condition: UV 365 nm, 30 s.

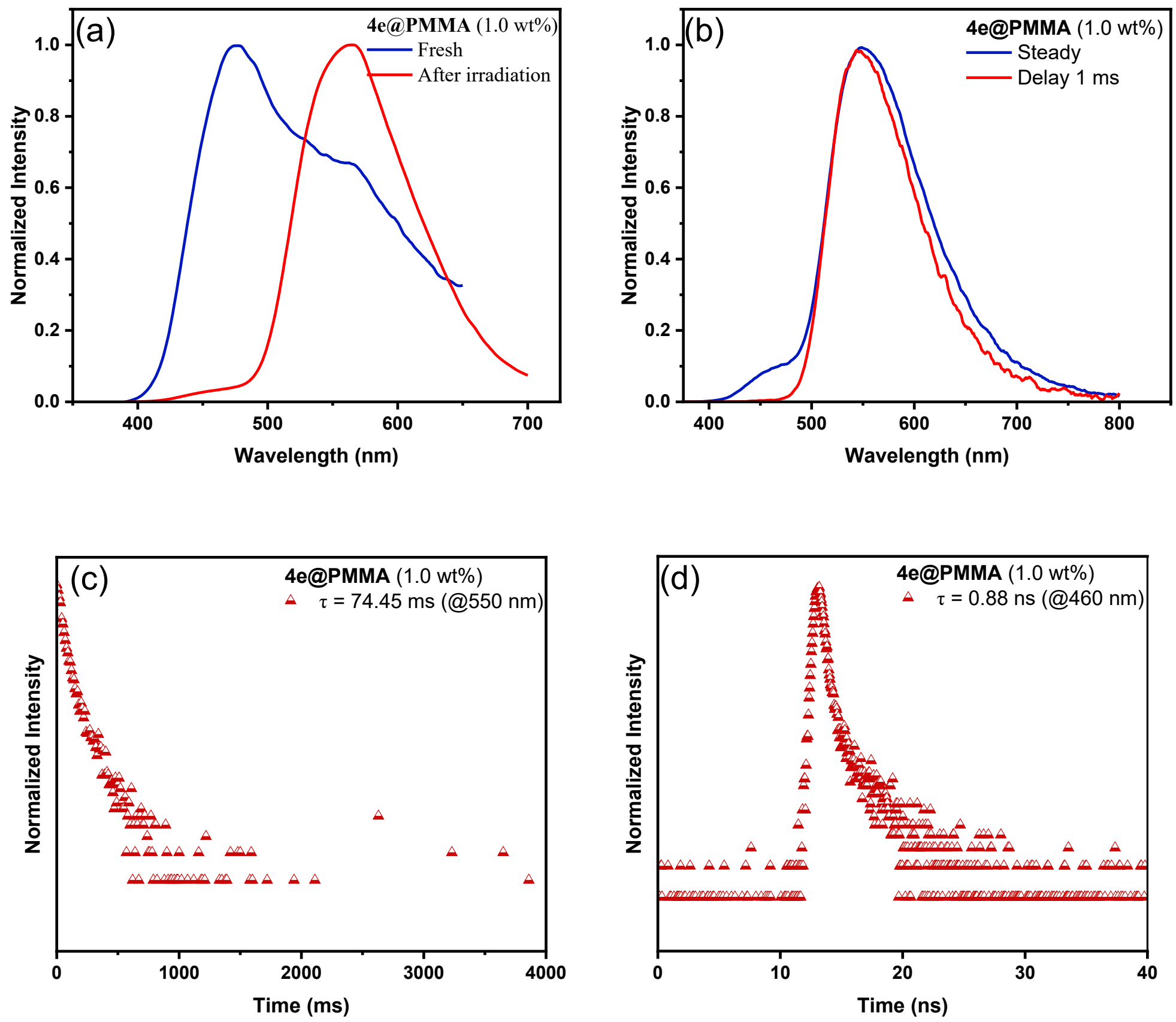

Fig. S7 (a) Steady state fluorescence (FL) spectra of **4e@PMMA** (1.0 wt%) before and after UV irradiation. (b) Steady-state FL and delayed PL spectra of **4e@PMMA** (1.0 wt%). (c) The time resolved PL-decay curve for phosphorescence at 550 nm of **4e@PMMA**. (d) The time resolved FL-decay curve for fluorescence at 460 nm of **4e@PMMA**. Fluorescence Spectrometer: Edinburgh FLS1000 and HITACHI F-7000. λ_{ex} = 365 nm. Irradiation condition: UV 365 nm, 30 s.

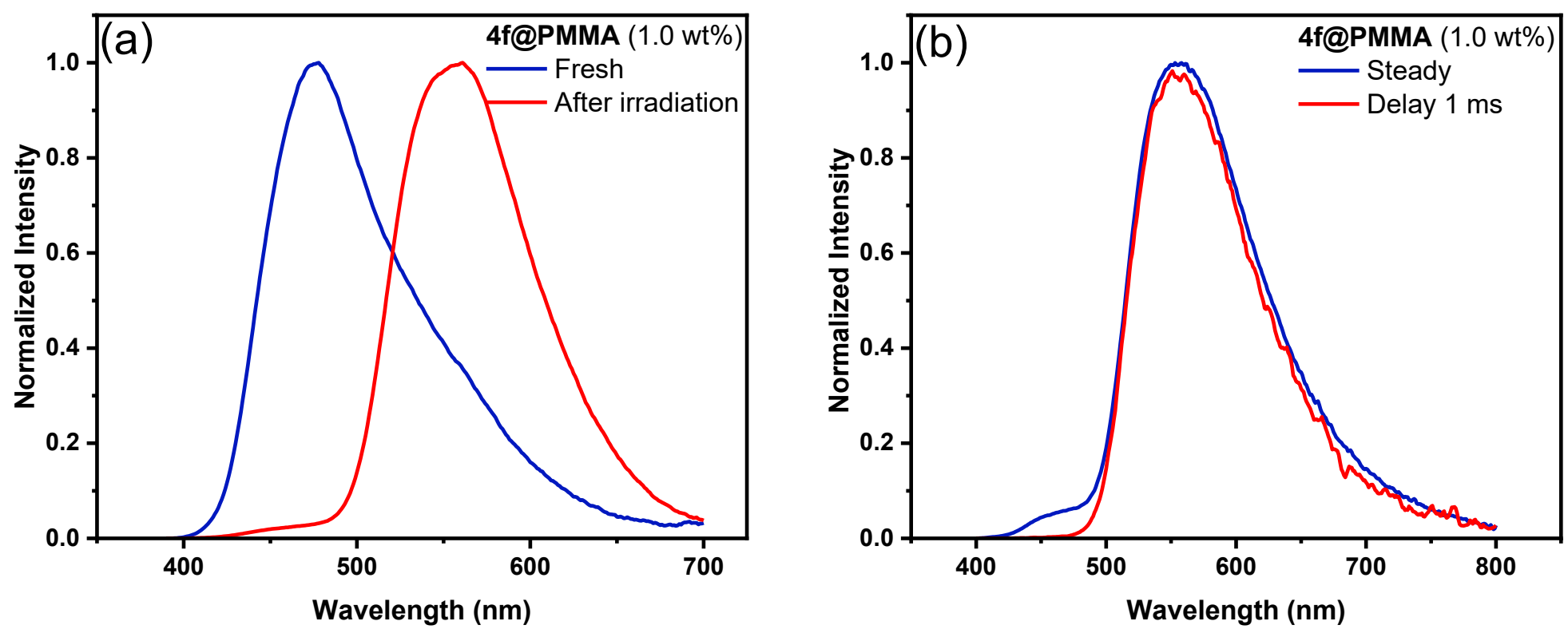

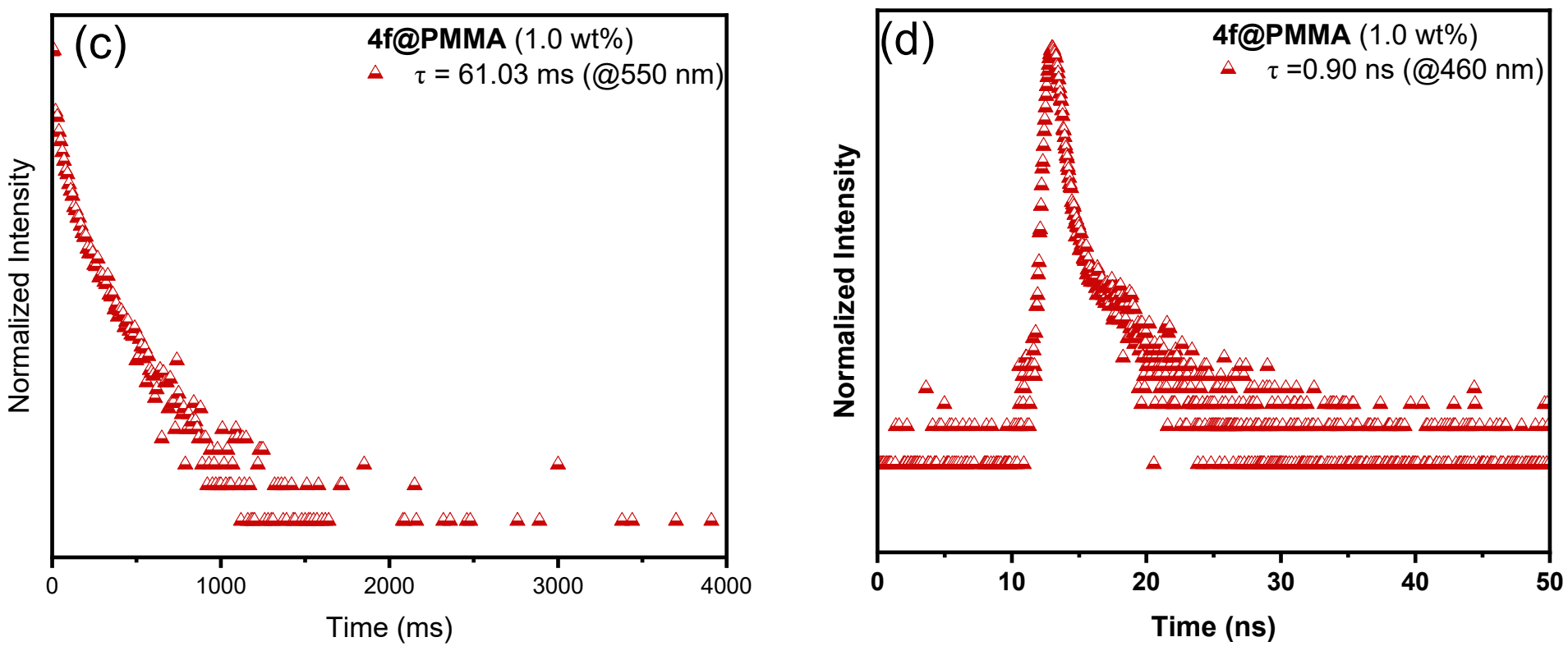

Fig. S8 (a) Steady state fluorescence (FL) spectra of **4f@PMMA** (1.0 wt%) before and after UV irradiation. (b) Steady-state FL and delayed PL spectra of **4f@PMMA** (1.0 wt%). (c) The time resolved PL-decay curve for phosphorescence at 550 nm of **4f@PMMA**. (d) The time resolved FL-decay curve for fluorescence at 460 nm of **4f@PMMA**. Fluorescence Spectrometer: Edinburgh FLS1000 and HITACHI F-7000. λ_{ex} = 365 nm. Irradiation condition: UV 365 nm, 30 s.

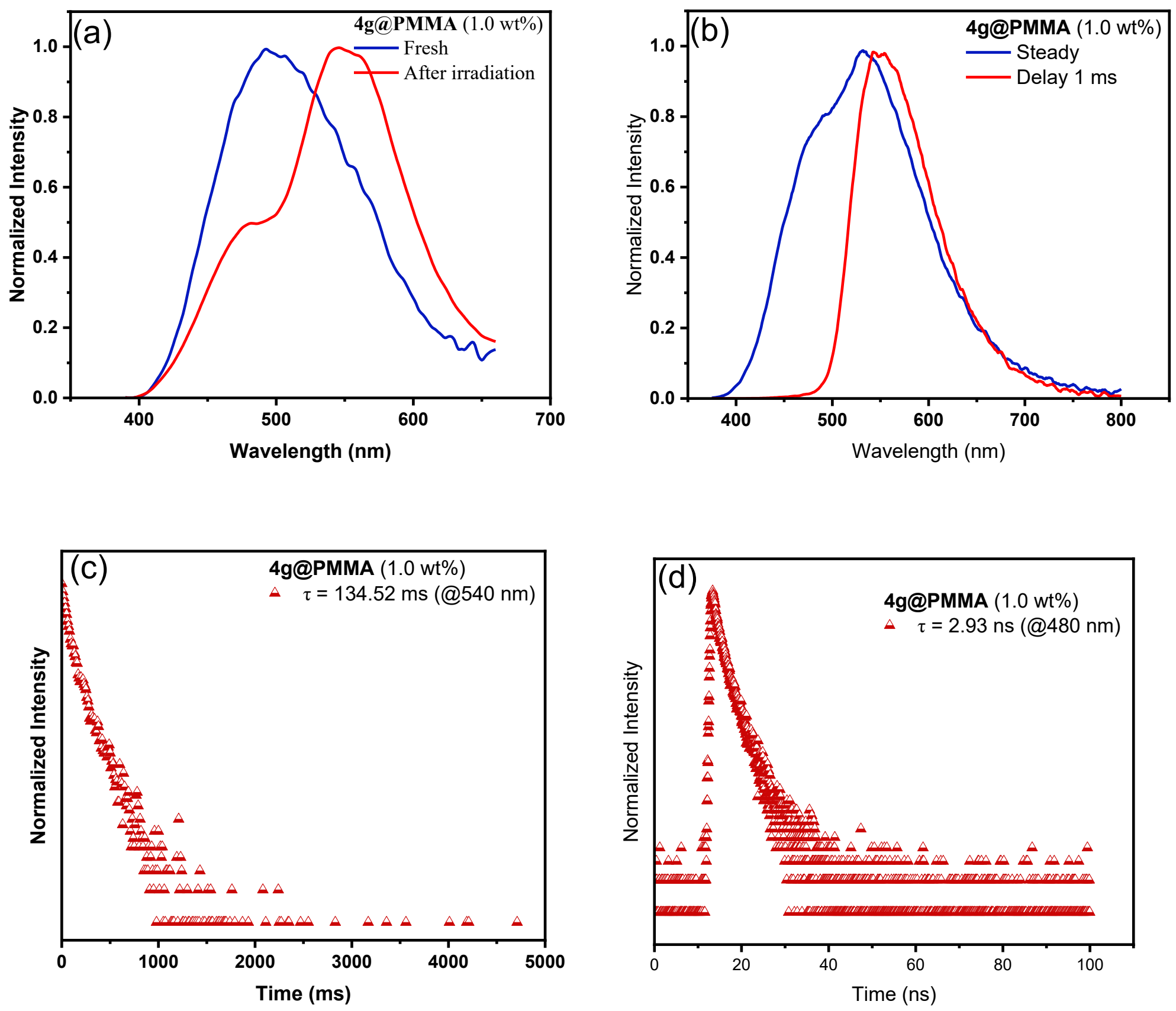

Fig. S9 (a) Steady state fluorescence (FL) spectra of **4g@PMMA** (1.0 wt%) before and after UV irradiation. (b) Steady-state FL and delayed PL spectra of **4g@PMMA** (1.0

wt%). (c) The time resolved PL-decay curve for phosphorescence at 540 nm of **4g@PMMA**. (d) The time resolved FL-decay curve for fluorescence at 480 nm of **4g@PMMA**. Fluorescence Spectrometer: Edinburgh FLS1000 and HITACHI F-7000. λ_{ex} = 365 nm. Irradiation condition: UV 365 nm, 30 s.

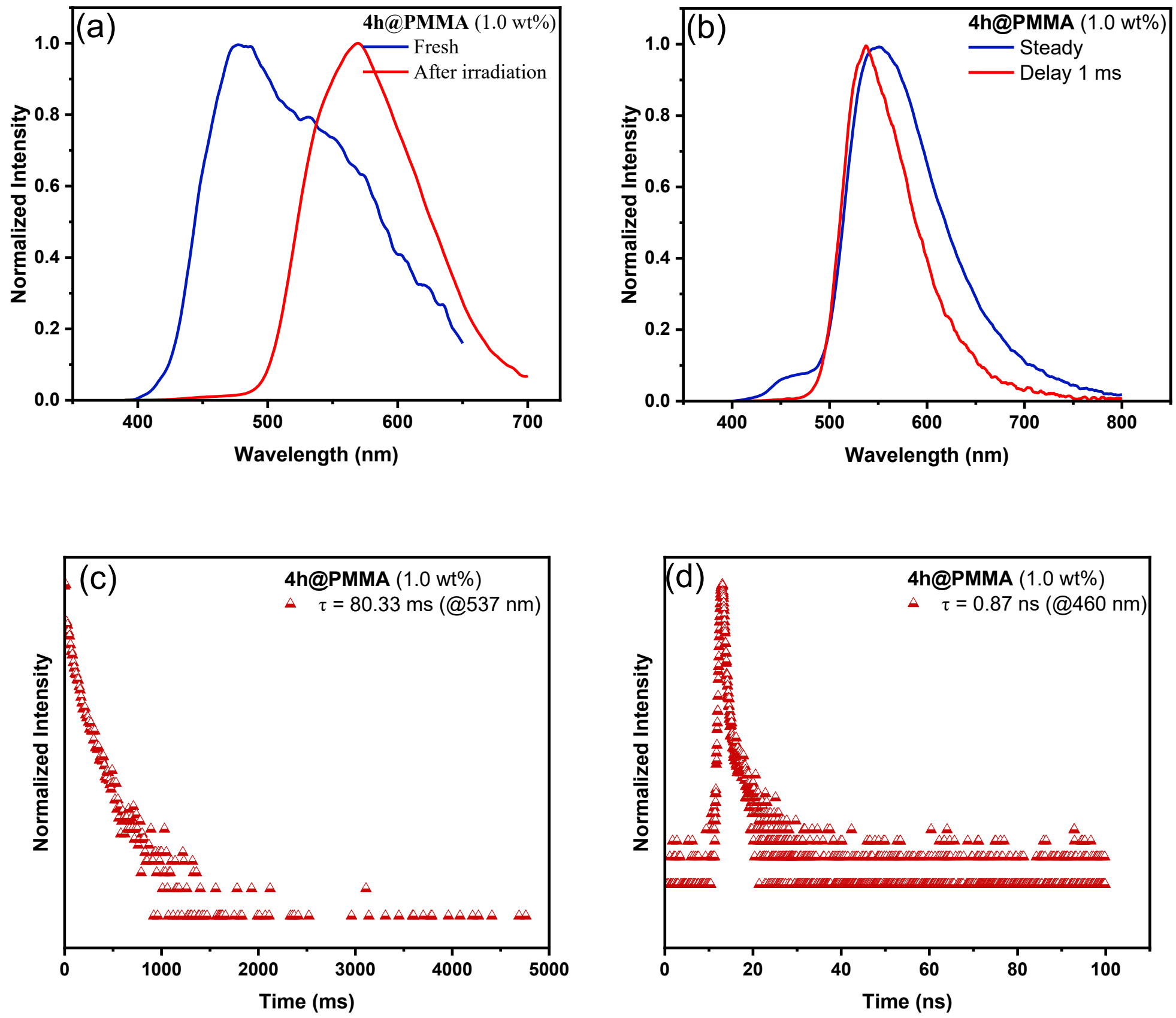

Fig. S10 (a) Steady state fluorescence (FL) spectra of **4h@PMMA** (1.0 wt%) before and after UV irradiation. (b) Steady-state FL and delayed PL spectra of **4h@PMMA** (1.0 wt%). (c) The time resolved PL-decay curve for phosphorescence at 537 nm of **4h@PMMA**. (d) The time resolved FL-decay curve for fluorescence at 460 nm of **4h@PMMA**. Fluorescence Spectrometer: Edinburgh FLS1000 and HITACHI F-7000. λ_{ex} = 365 nm. Irradiation condition: UV 365 nm, 30 s.

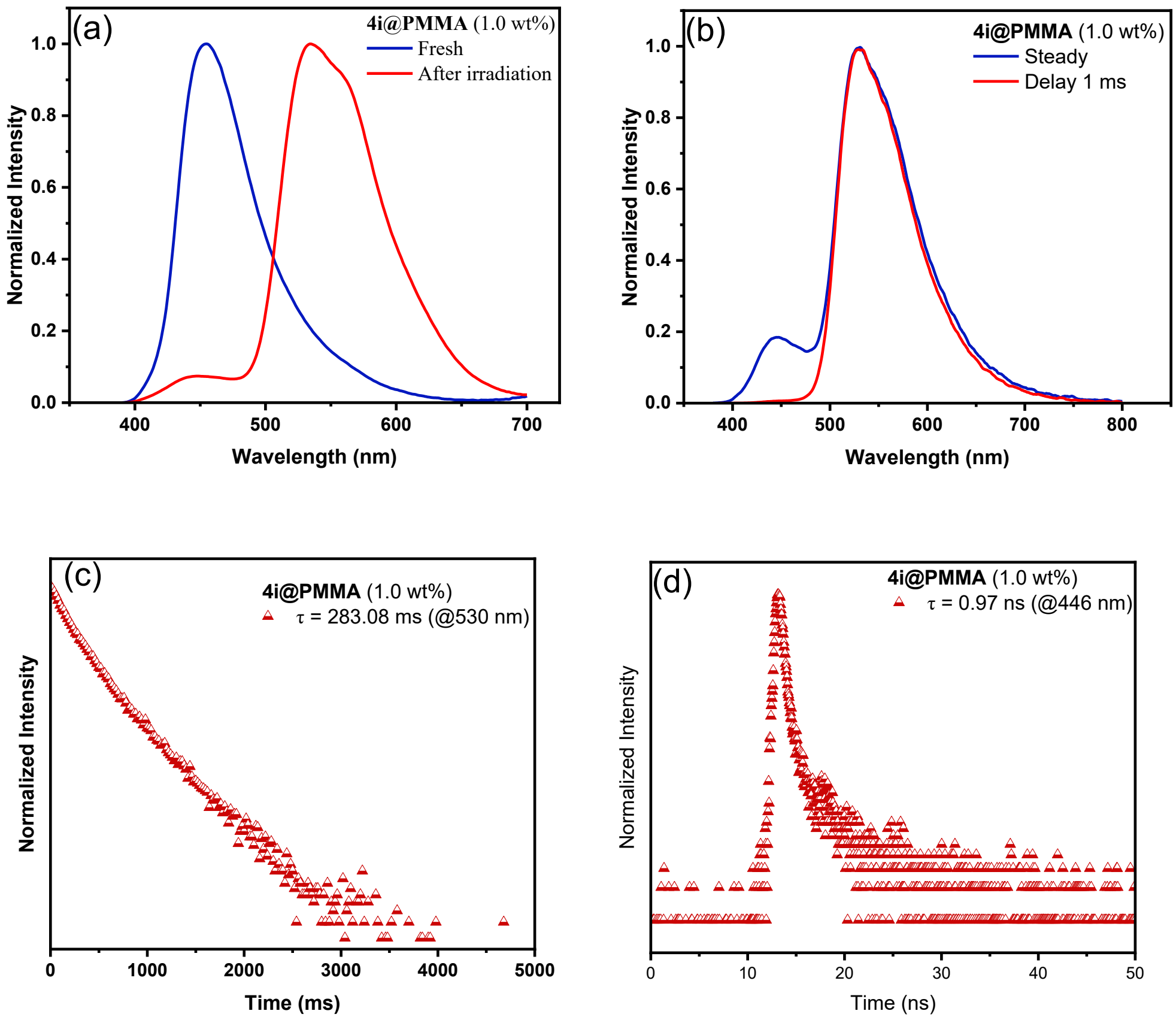

Fig. S11 (a) Steady state fluorescence (FL) spectra of **4i@PMMA** (1.0 wt%) before and after UV irradiation. (b) Steady-state FL and delayed PL spectra of **4i@PMMA** (1.0 wt%). (c) The time resolved PL-decay curve for phosphorescence at 530 nm of **4i@PMMA**. (d) The time resolved FL-decay curve for fluorescence at 446 nm of **4i@PMMA**. Fluorescence Spectrometer: Edinburgh FLS1000 and HITACHI F-7000. λ_{ex} = 365 nm. Irradiation condition: UV 365 nm, 30 s.

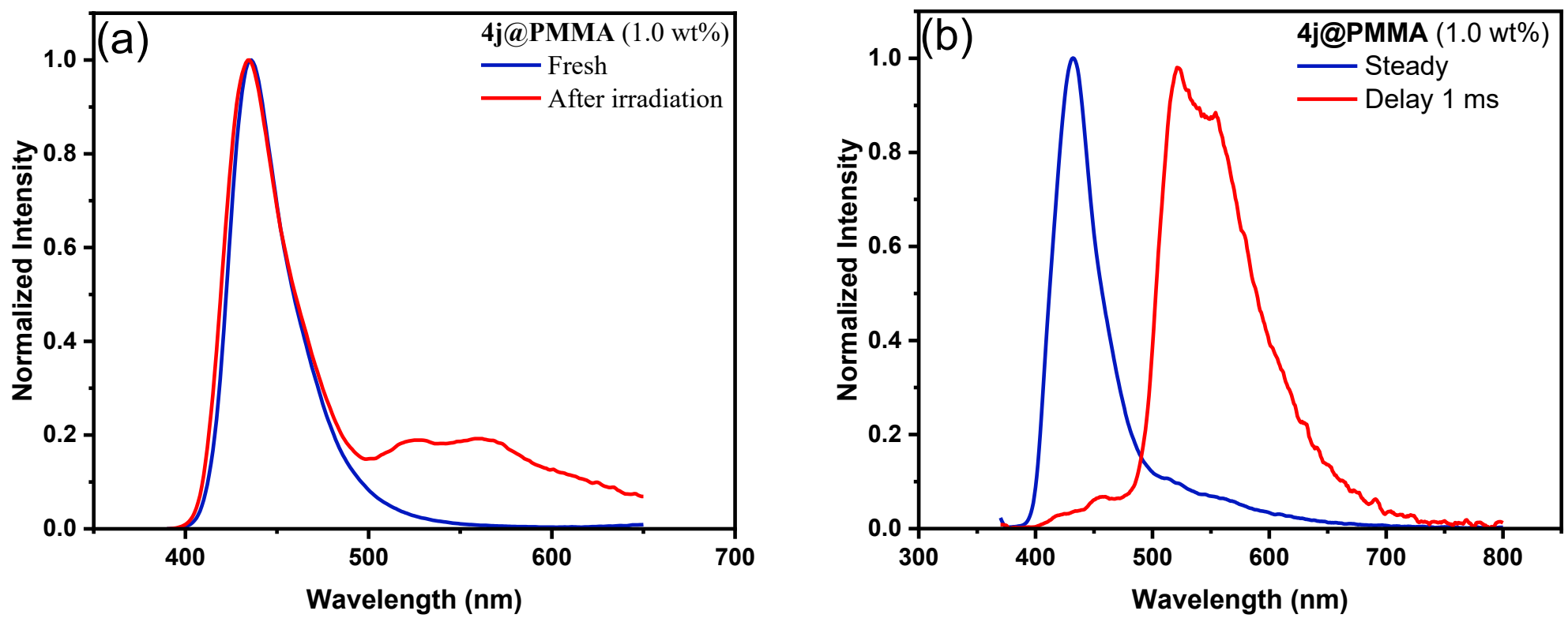

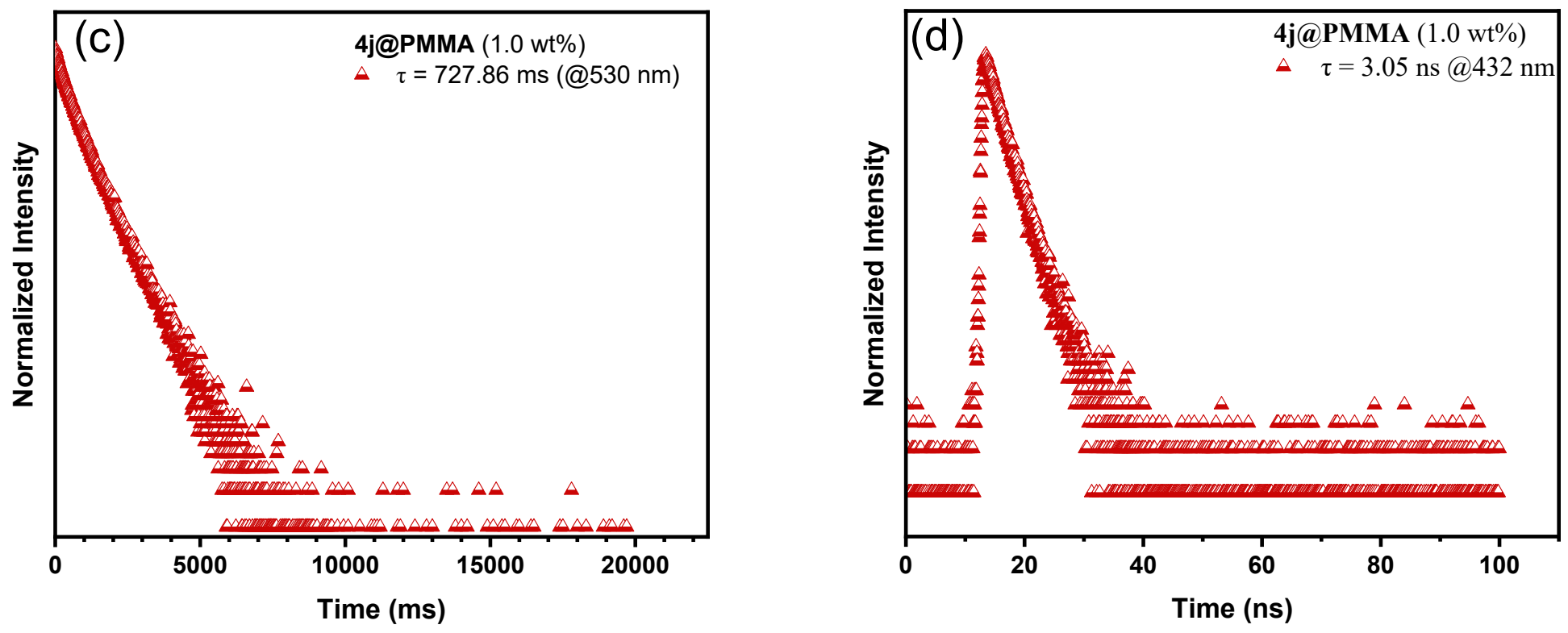

Fig. S12 (a) Steady state fluorescence (FL) spectra of **4j@PMMA** (1.0 wt%) before and after UV irradiation. (b) Steady-state FL and delayed PL spectra of **4j@PMMA** (1.0 wt%). (c) The time resolved PL-decay curve for phosphorescence at 530 nm of **4j@PMMA**. (d) The time resolved FL-decay curve for fluorescence at 432 nm of **4j@PMMA**. Fluorescence Spectrometer: Edinburgh FLS1000 and HITACHI F-7000. λ_{ex} = 365 nm. Irradiation condition: UV 365 nm, 30 s.

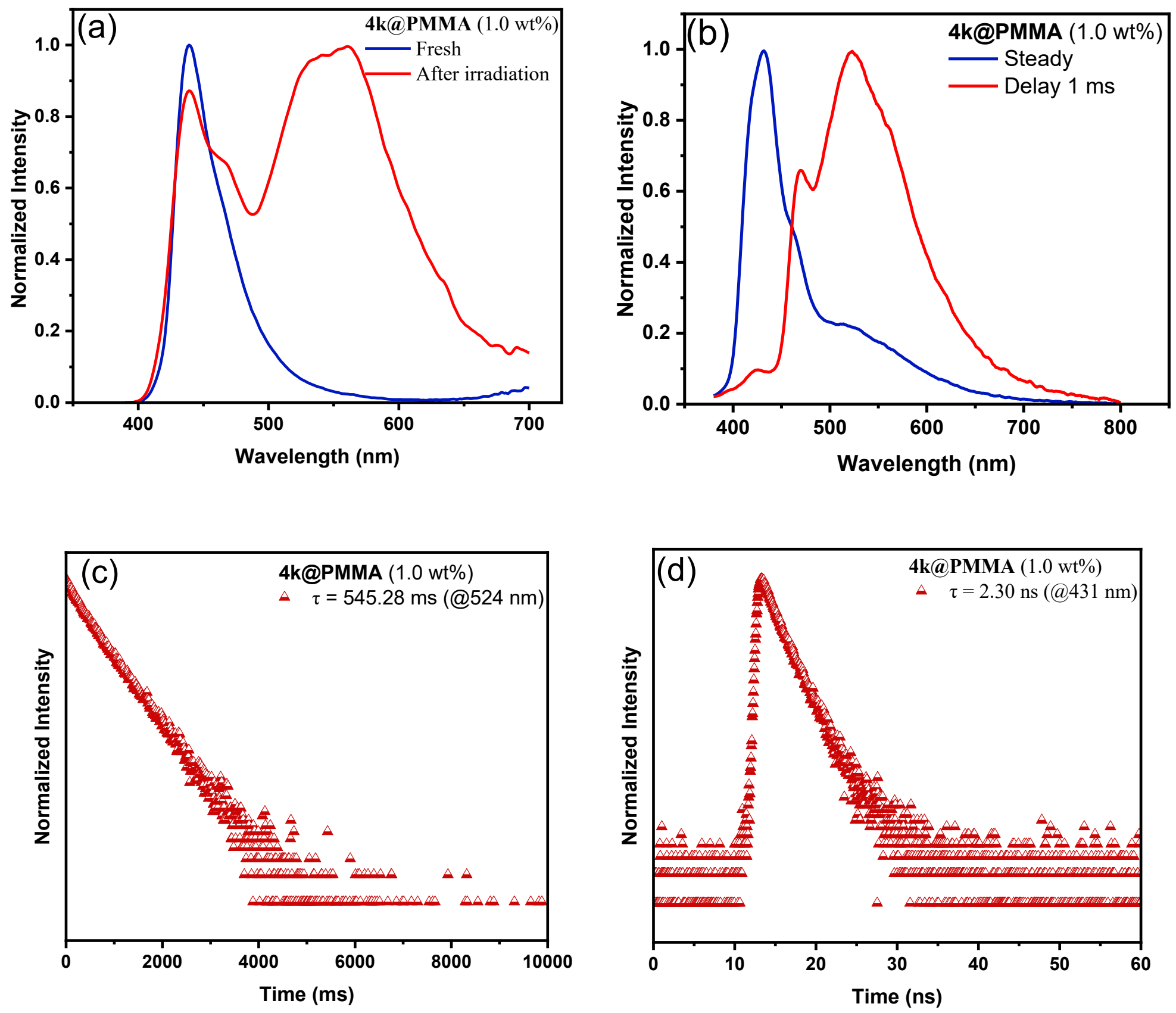

Fig. S13 (a) Steady state fluorescence (FL) spectra of **4k@PMMA** (1.0 wt%) before and after UV irradiation. (b) Steady-state FL and delayed PL spectra of **4k@PMMA**

(1.0 wt%). (c) The time resolved PL-decay curve for phosphorescence at 524 nm of **4k@PMMA**. (d) The time resolved FL-decay curve for fluorescence at 431 nm of **4k@PMMA**. Fluorescence Spectrometer: Edinburgh FLS1000 and HITACHI F-7000. λ_{ex} = 365 nm. Irradiation condition: UV 365 nm, 30 s.

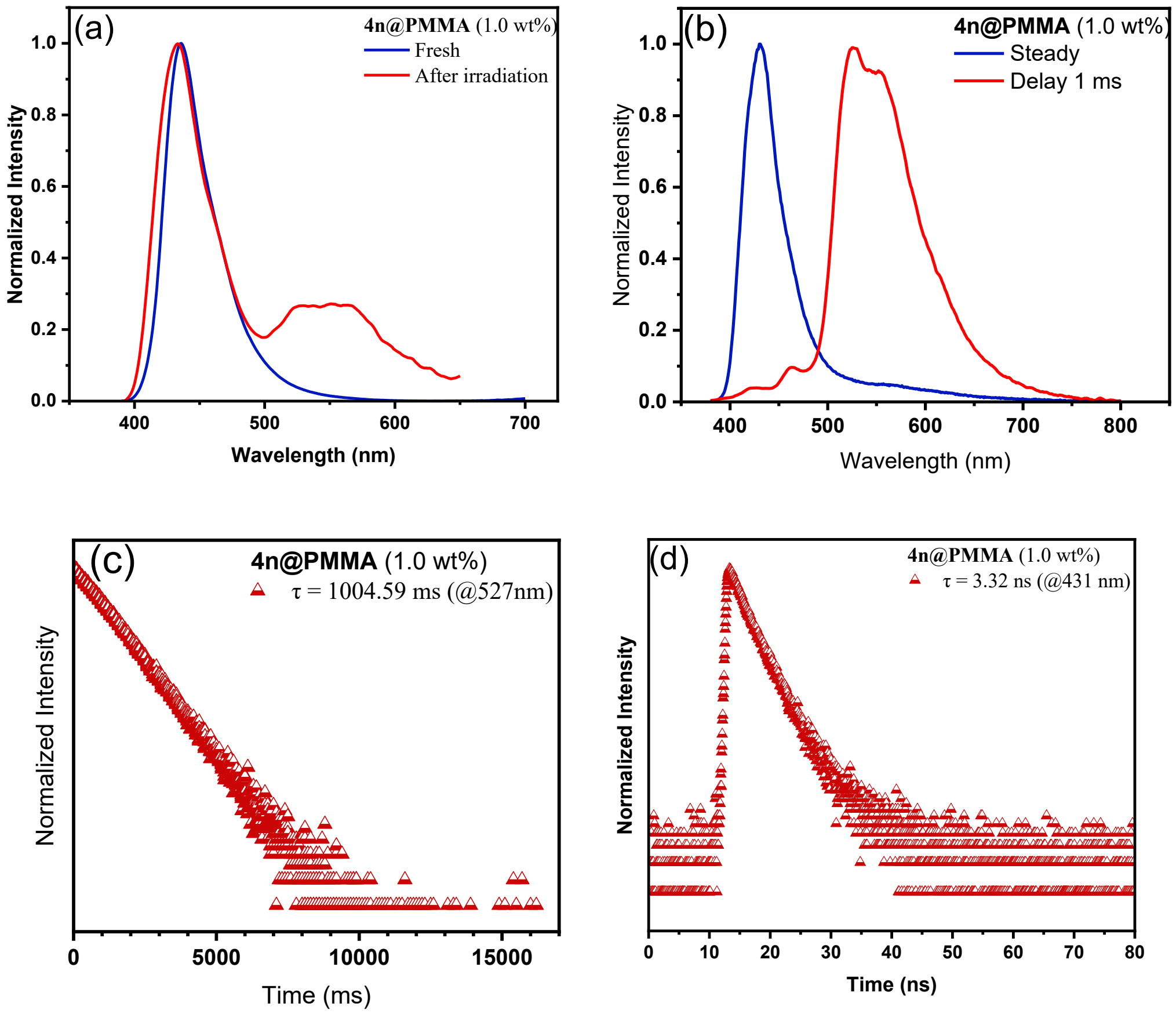

Fig. S14 (a) Steady state fluorescence (FL) spectra of **4n@PMMA** (1.0 wt%) before and after UV irradiation. (b) Steady-state FL and delayed PL spectra of **4n@PMMA** (1.0 wt%). (c) The time resolved PL-decay curve for phosphorescence at 527 nm of **4n@PMMA**. (d) The time resolved FL-decay curve for fluorescence at 431 nm of **4n@PMMA**. Fluorescence Spectrometer: Edinburgh FLS1000 and HITACHI F-7000. λ_{ex} = 365 nm. Irradiation condition: UV 365 nm, 30 s.

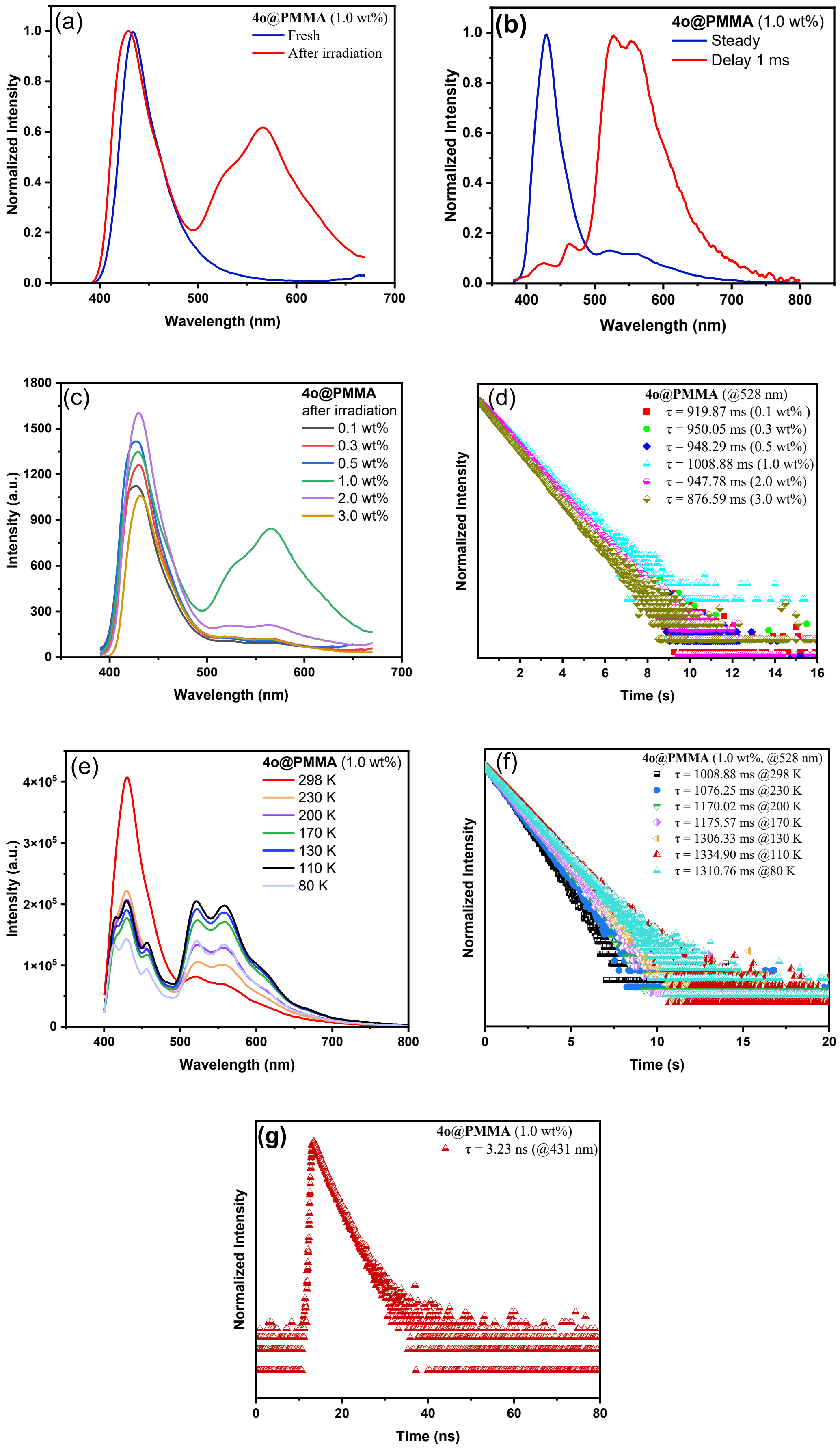
(a)
4o@PMMA (1.0 wt%)
Fresh
After irradiation
Normalized Intensity
Wavelength (nm)
(b)
4o@PMMA (1.0 wt%)
Steady
Delay 1 ms
Normalized Intensity
Wavelength (nm)
(c)
4o@PMMA
after irradiation
0.1 wt%
0.3 wt%
0.5 wt%
1.0 wt%
2.0 wt%
3.0 wt%
Intensity (a.u.)
Wavelength (nm)
(d)
4o@PMMA (@528 nm)
τ = 919.87 ms (0.1 wt%)
τ = 950.05 ms (0.3 wt%)
τ = 948.29 ms (0.5 wt%)
τ = 1008.88 ms (1.0 wt%)
τ = 947.78 ms (2.0 wt%)
τ = 876.59 ms (3.0 wt%)
Normalized Intensity
Time (s)
(e)
4o@PMMA (1.0 wt%)
298 K
230 K
200 K
170 K
130 K
110 K
80 K
Intensity (a.u.)
Wavelength (nm)
(f)
4o@PMMA (1.0 wt%, @528 nm)
τ = 1008.88 ms @298 K
τ = 1076.25 ms @230 K
τ = 1170.02 ms @200 K
τ = 1175.57 ms @170 K
τ = 1306.33 ms @130 K
τ = 1334.90 ms @110 K
τ = 1310.76 ms @80 K
Normalized Intensity
Time (s)
(g)
4o@PMMA (1.0 wt%)
τ = 3.23 ns (@431 nm)
Normalized Intensity
Time (ns)

Fig. S15 (a) Steady state fluorescence (FL) spectra of **4o@PMMA** (1.0 wt%) before and after UV irradiation. (b) Steady-state FL and delayed PL spectra of **4o@PMMA** (1.0 wt%). (c) Steady state PL of **4o@PMMA** with different concentration after UV irradiation. (d) The time resolved PL-decay curve for phosphorescence at 528 nm of **4o@PMMA** with different concentration. (e) Steady state PL of **4o@PMMA** (1.0 wt%) at different temperature. (f) The time resolved PL-decay curve for phosphorescence at 528 nm of **4o@PMMA** (1.0 wt%) at different temperature. (g) The time resolved FL-decay curve for fluorescence at 431 nm of **4o@PMMA** (1.0 wt%) at different temperature. Fluorescence Spectrometer: Edinburgh FLS1000 and HITACHI F-7000. λ_{ex} = 365 nm. Irradiation condition: UV 365 nm, 30 s.

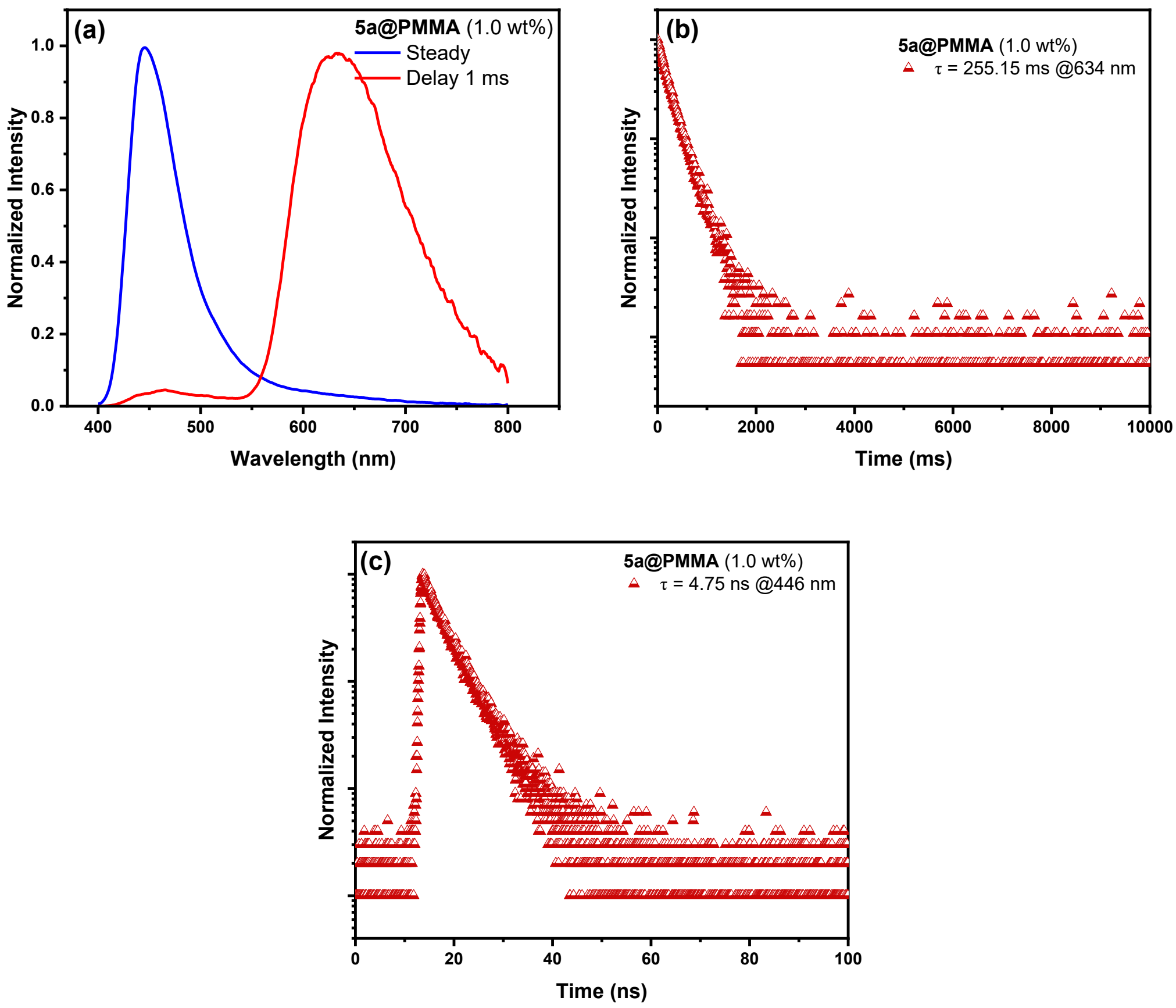

Fig. S16 (a) Steady-state FL and delayed PL spectra of **5a@PMMA** (1.0 wt%). (b) The time resolved PL-decay curve for phosphorescence at 634 nm of **5a@PMMA**. (c) The time resolved FL-decay curve for fluorescence at 446 nm of **5a@PMMA**. Fluorescence Spectrometer: Edinburgh FLS1000 and HITACHI F-7000. λ_{ex} = 365 nm. Irradiation condition: UV 365 nm, 30 s.

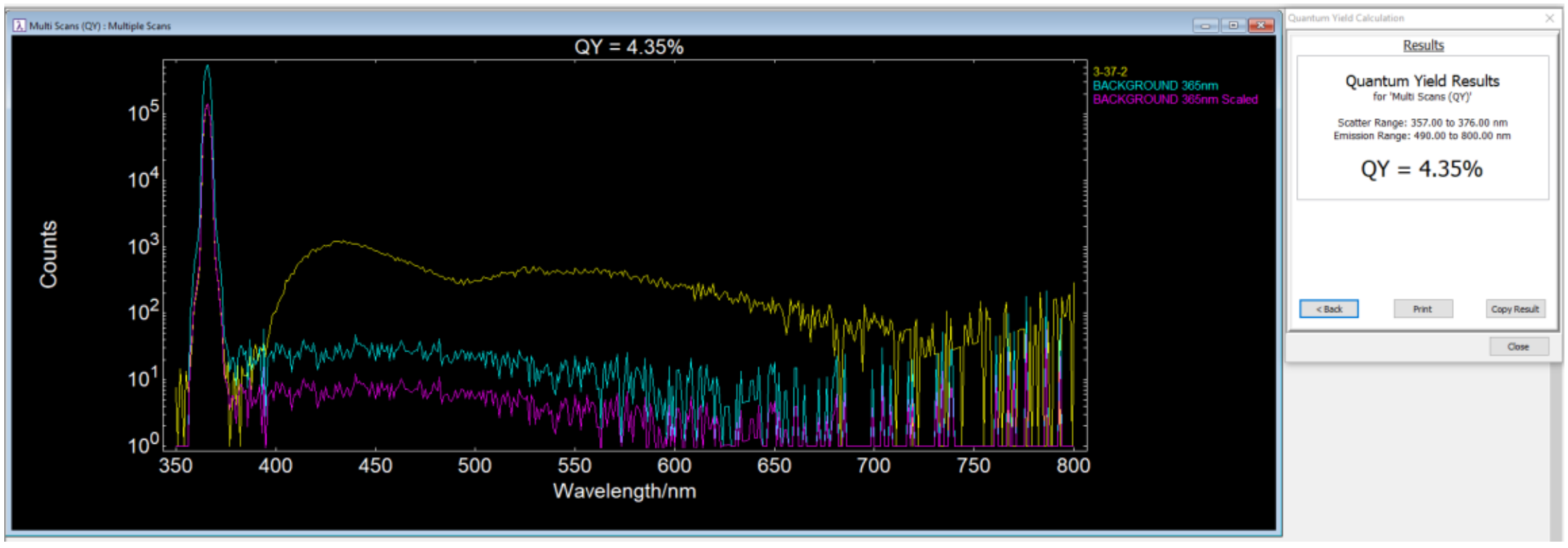

Fig. S17 The absolute phosphorescence quantum yield of **4n@PMMA** (1.0 wt%)

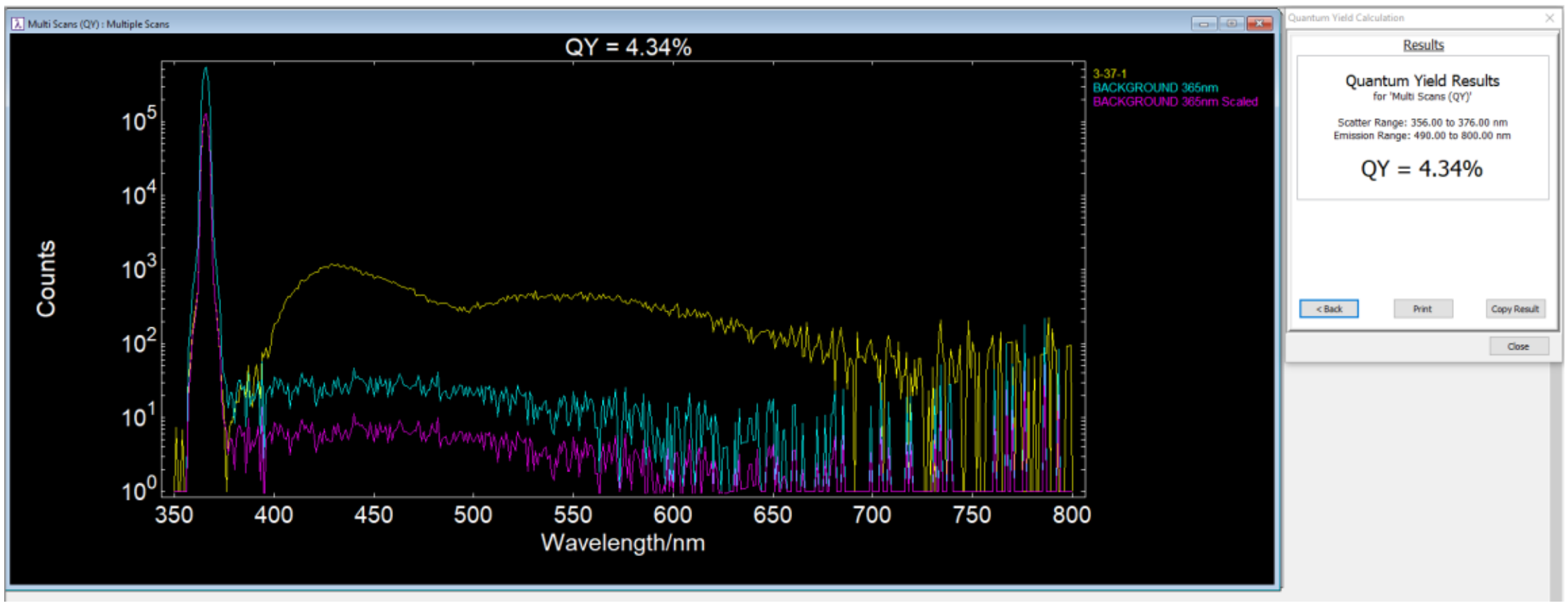

Fig. S18 The absolute phosphorescence quantum yield of **4o@PMMA** (1.0 wt%)

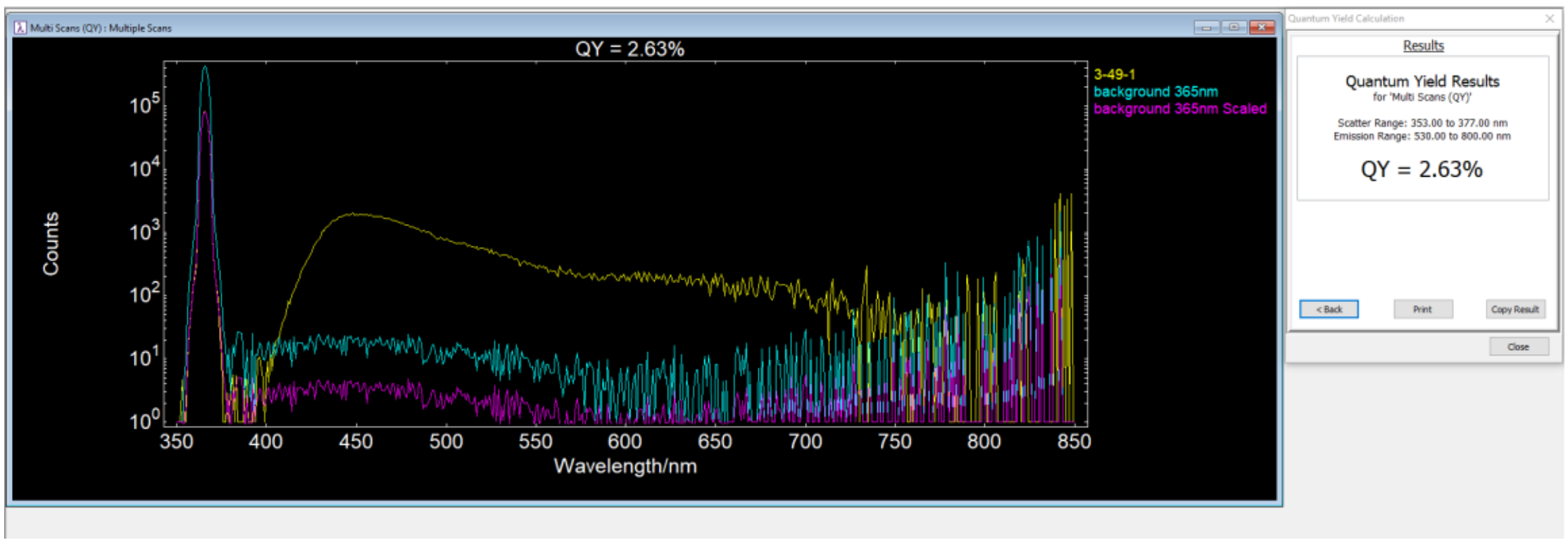

Fig. S19 The absolute phosphorescence quantum yield of **5a@PMMA** (1.0 wt%)

3.2. Photophysical properties of 4a, 4c-4o and 5a-5b@HEA-AA

General procedures for preparation of HEA-AA film samples: Crosslinker poly(ethylene glycol) diacrylate (PEGDA, 1.5 wt%) and photoinitiator diphenyl(2,4,6-trimethylbenzoyl)phosphine oxide (TPO, 1.5 wt%) were added to the mixture of

monomers hydroxyethyl acrylate (HEA) and acrylic acid (AA) (weight ratio: HEA/AA = 7/3). Then phenothiazine-besed polycyclic aromatic hydrocarbons (0.05 wt%) was incorporated into the resin as guest molecules. The mixture was ultrasonicated at room temperature until the solid compound completely dissolved to obtain a homogeneous solution of precursors. The homogeneous solution was dropped onto a glass substrate and exposed to 365nm UV light for 15 min. The films were placed in a drying oven at 60 °C for 6 h.

Fluorescence spectra were collected on Edinburgh FLS1000. The phosphorescence spectra, excited-state lifetimes and absolute quantum yields were taken using Edinburgh Instruments FLS1000 fluorescence spectrometer. In a typical experiment, a HEA-AA (HEA:AA =7:3) film doped with desired phenothiazine-besed polycyclic aromatic hydrocarbons (0.05 wt%) for the collection of emission spectrum excited-state lifetimes and absolute quantum yields were prepared according to the reported literature[S5].

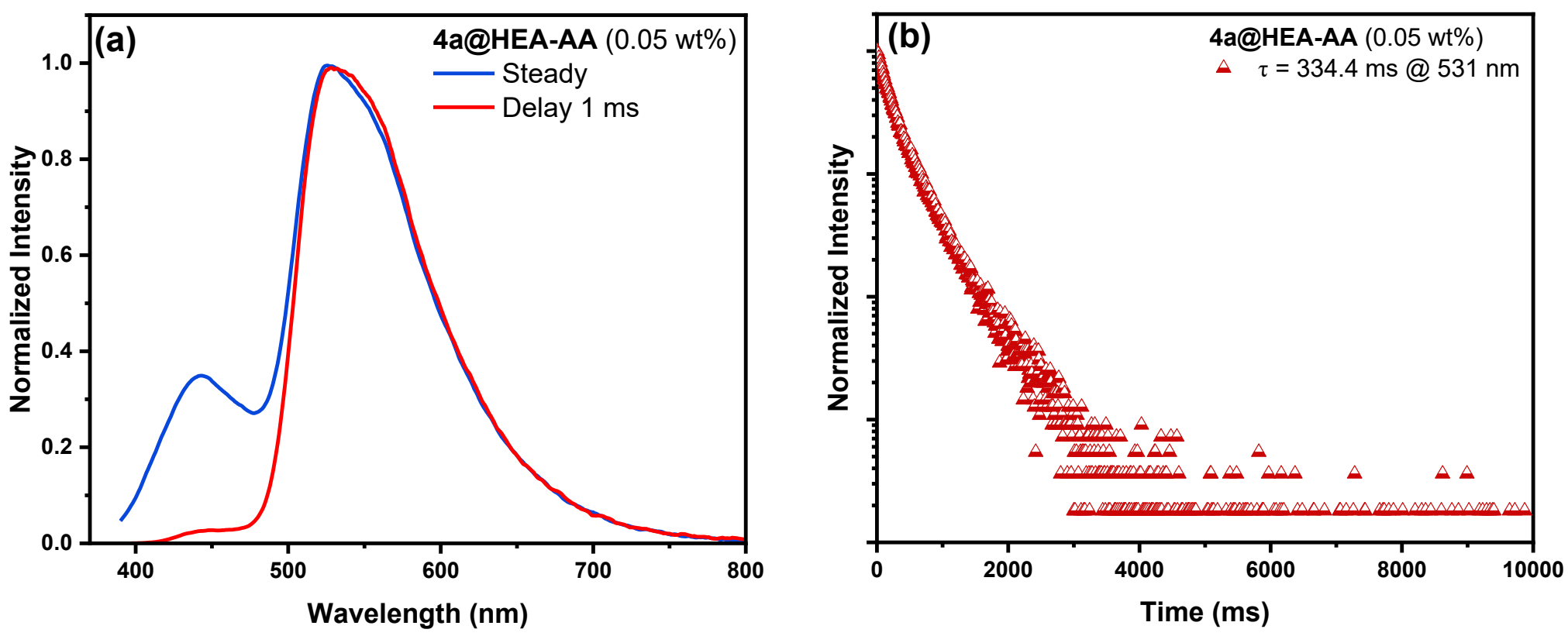

Fig. S20 (a) Steady-state FL and delayed PL spectra of **4a@HEA-AA** (0.05 wt%). (b) The time-resolved PL-decay curve for phosphorescence at 531 nm of **4a@HEA-AA** (0.05 wt%). The weight ratio of HEA and AA is HEA/AA = 7/3. Fluorescence Spectrometer: Edinburgh FLS1000. λ_{ex} = 365 nm. Irradiation condition: UV 365 nm, 30 s.

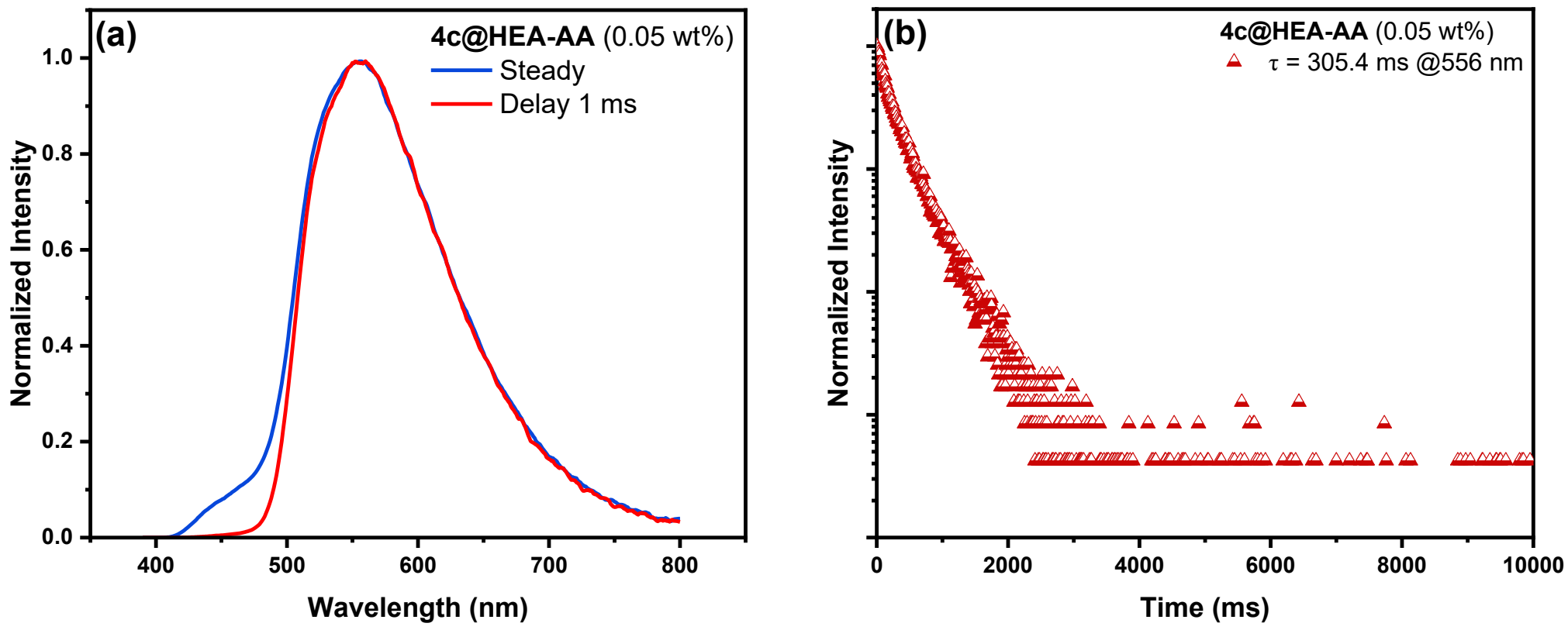

Fig. S21 (a) Steady-state FL and delayed PL spectra of **4c@HEA-AA** (0.05 wt%). (b) The time resolved PL-decay curve for phosphorescence at 556 nm of **4c@HEA-AA** (0.05 wt%). The weight ratio of HEA and AA is HEA/AA = 7/3. Fluorescence Spectrometer: Edinburgh FLS1000. λ_{ex} = 365 nm. Irradiation condition: UV 365 nm, 30 s.

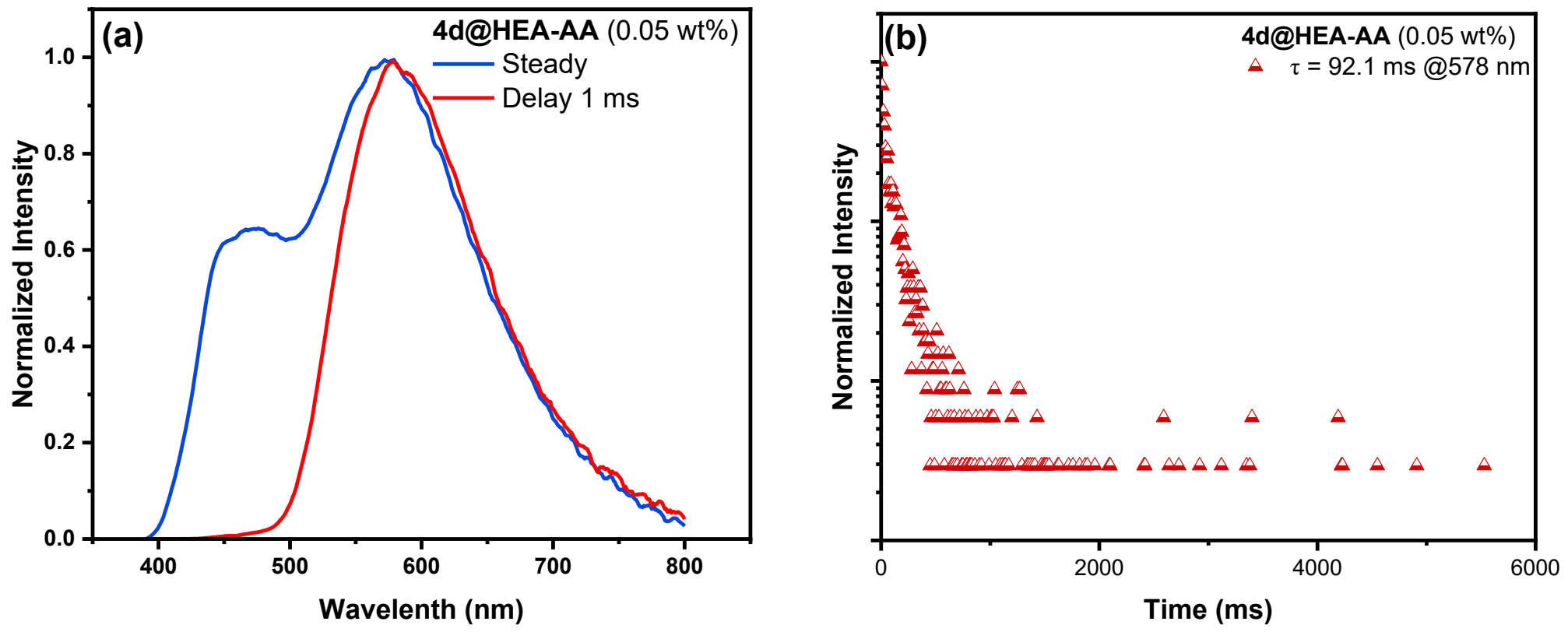

Fig. S22 (a) Steady-state FL and delayed PL spectra of **4d@HEA-AA** (0.05 wt%). (b) The time resolved PL-decay curve for phosphorescence at 578 nm of **4d@HEA-AA** (0.05 wt%). The weight ratio of HEA and AA is HEA/AA = 7/3. Fluorescence Spectrometer: Edinburgh FLS1000. λ_{ex} = 365 nm. Irradiation condition: UV 365 nm, 30 s.

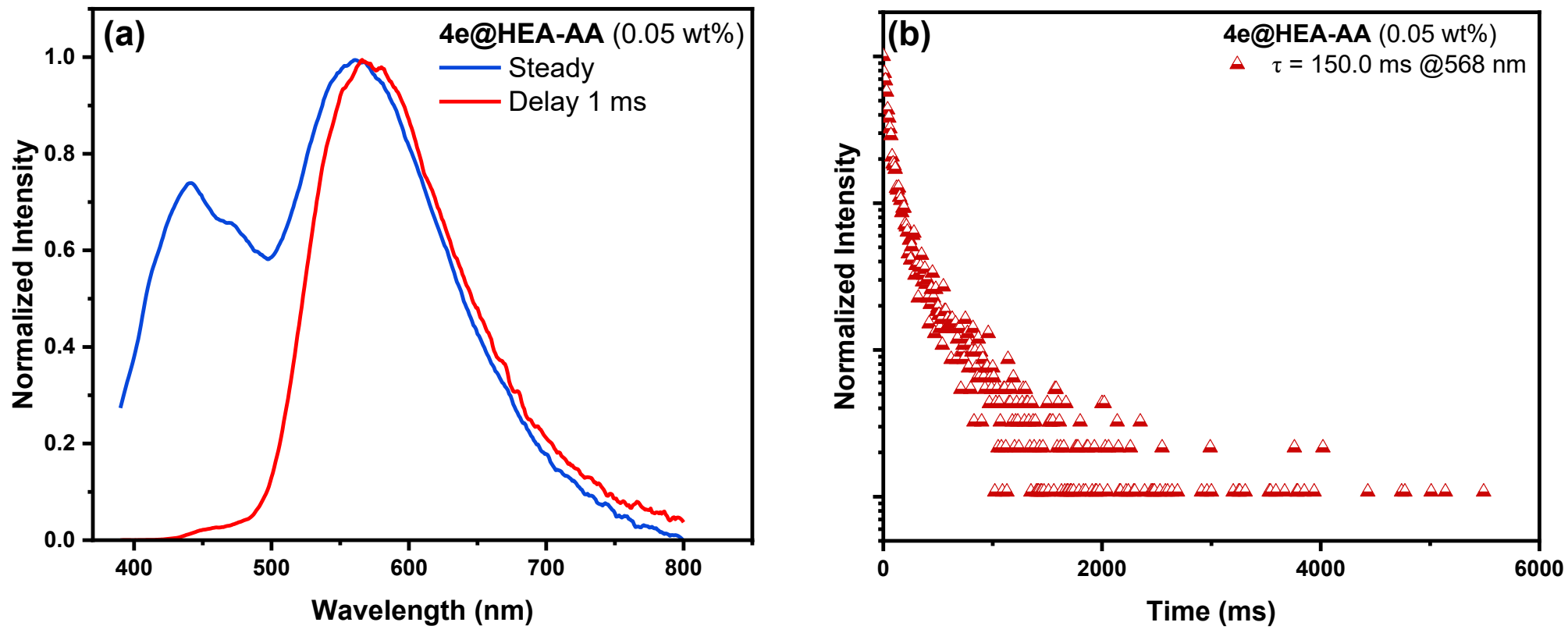

Fig. S23 (a) Steady-state FL and delayed PL spectra of **4e@HEA-AA** (0.05 wt%). (b) The time resolved PL-decay curve for phosphorescence at 568 nm of **4e@HEA-AA** (0.05 wt%). The weight ratio of HEA and AA is HEA/AA = 7/3. Fluorescence Spectrometer: Edinburgh FLS1000. λ_{ex} = 365 nm. Irradiation condition: UV 365 nm, 30 s.

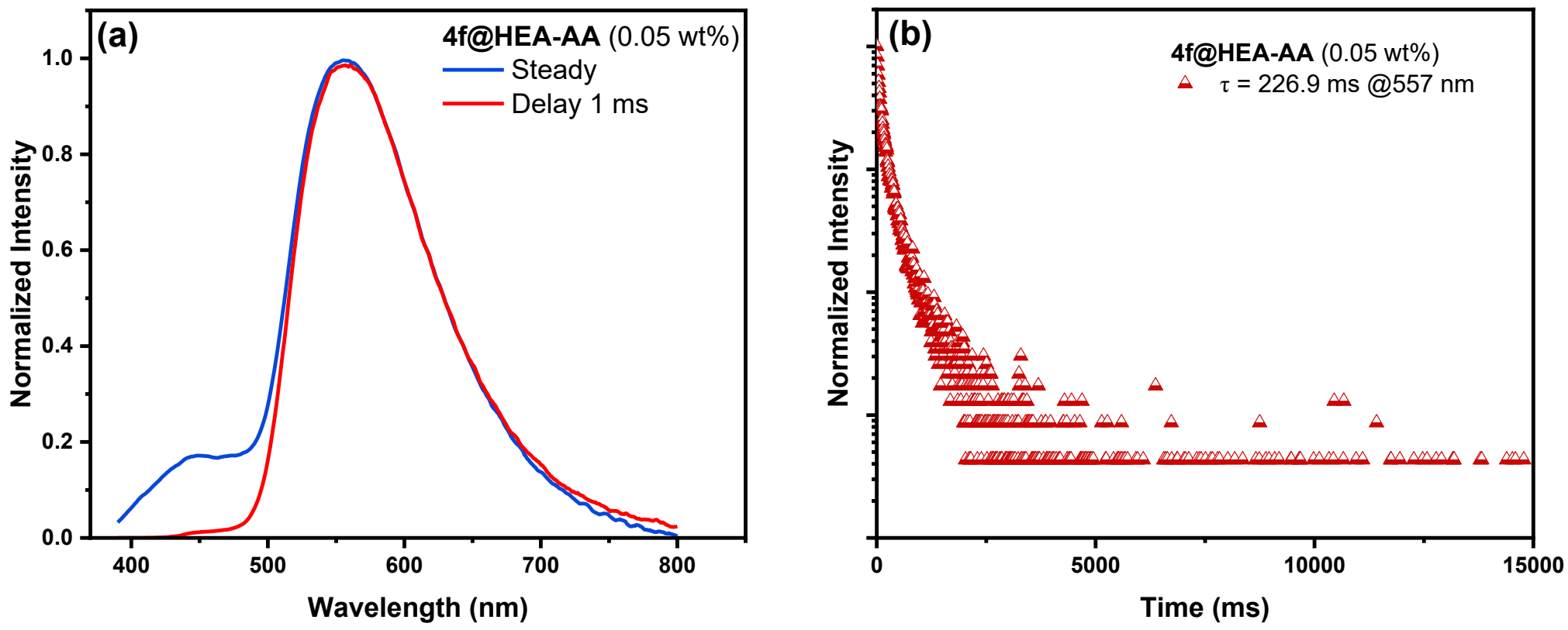

Fig. S24 (a) Steady-state FL and delayed PL spectra of **4f@HEA-AA** (0.05 wt%). (b) The time resolved PL-decay curve for phosphorescence at 557 nm of **4f@HEA-AA** (0.05 wt%). The weight ratio of HEA and AA is HEA/AA = 7/3. Fluorescence Spectrometer: Edinburgh FLS1000. λ_{ex} = 365 nm. Irradiation condition: UV 365 nm, 30 s.

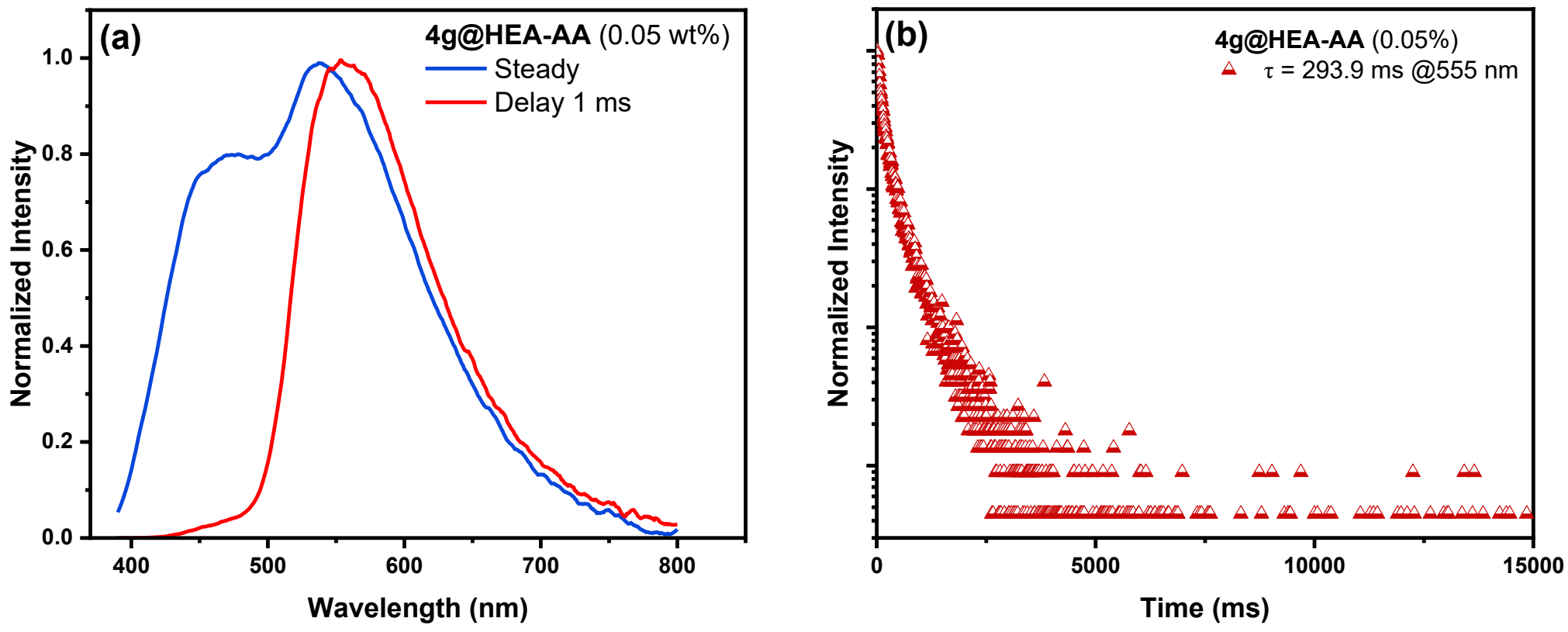

Fig. S25 (a) Steady-state FL and delayed PL spectra of **4g@HEA-AA** (0.05 wt%). (b) The time resolved PL-decay curve for phosphorescence at 555 nm of **4g@HEA-AA** (0.05 wt%). The weight ratio of HEA and AA is HEA/AA = 7/3. Fluorescence Spectrometer: Edinburgh FLS1000. λ_{ex} = 365 nm. Irradiation condition: UV 365 nm, 30 s.

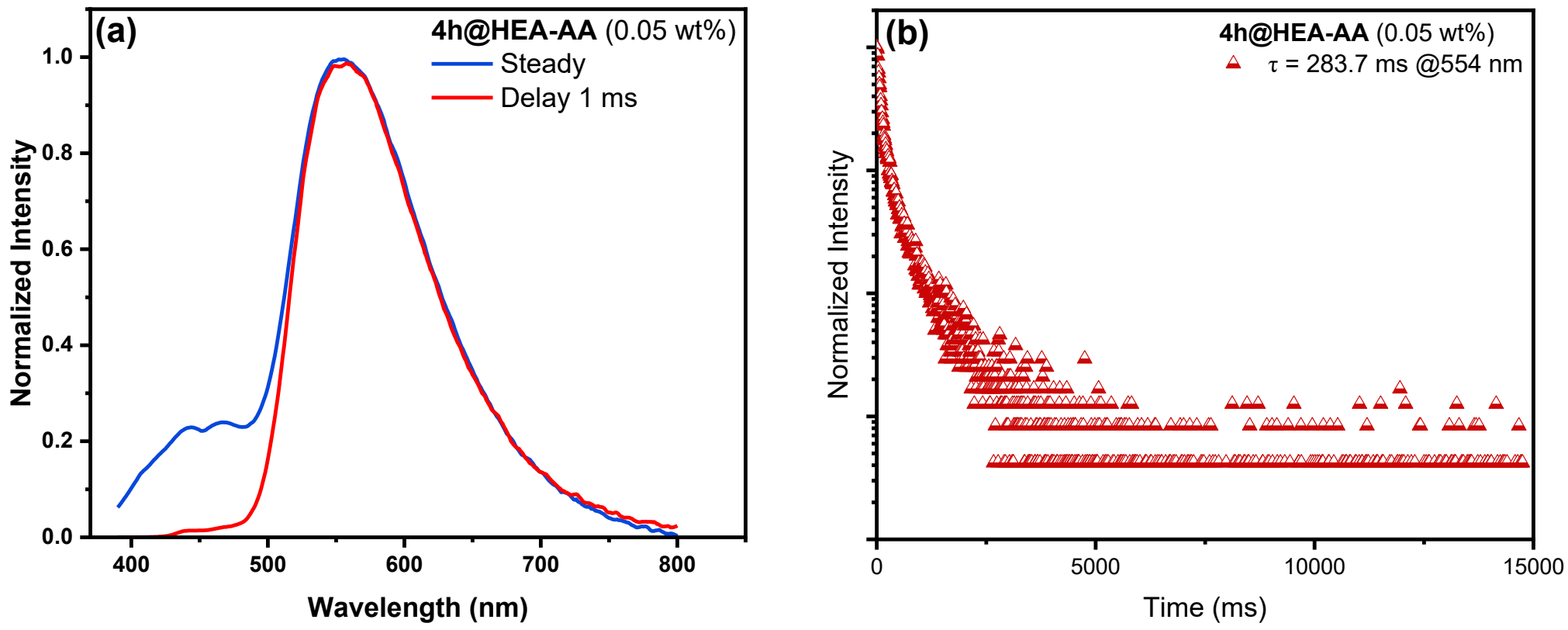

Fig. S26 (a) Steady-state FL and delayed PL spectra of **4h@HEA-AA** (0.05 wt%). (b) The time resolved PL-decay curve for phosphorescence at 554 nm of **4h@HEA-AA** (0.05 wt%). The weight ratio of HEA and AA is HEA/AA = 7/3. Fluorescence Spectrometer: Edinburgh FLS1000. λ_{ex} = 365 nm. Irradiation condition: UV 365 nm, 30 s.

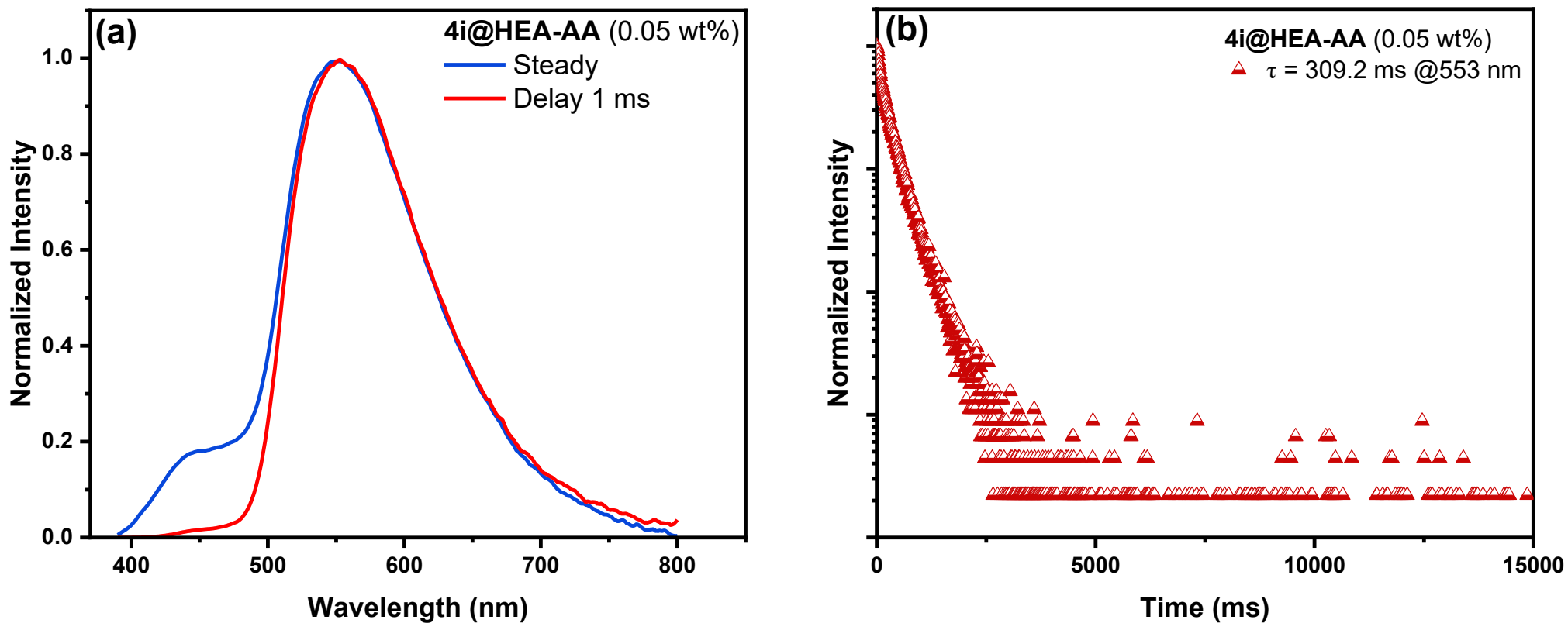

Fig. S27 (a) Steady-state FL and delayed PL spectra of **4i@HEA-AA** (0.05 wt%). (b) The time resolved PL-decay curve for phosphorescence at 553 nm of **4i@HEA-AA** (0.05 wt%). The weight ratio of HEA and AA is HEA/AA = 7/3. Fluorescence Spectrometer: Edinburgh FLS1000. λ_{ex} = 365 nm. Irradiation condition: UV 365 nm, 30 s.

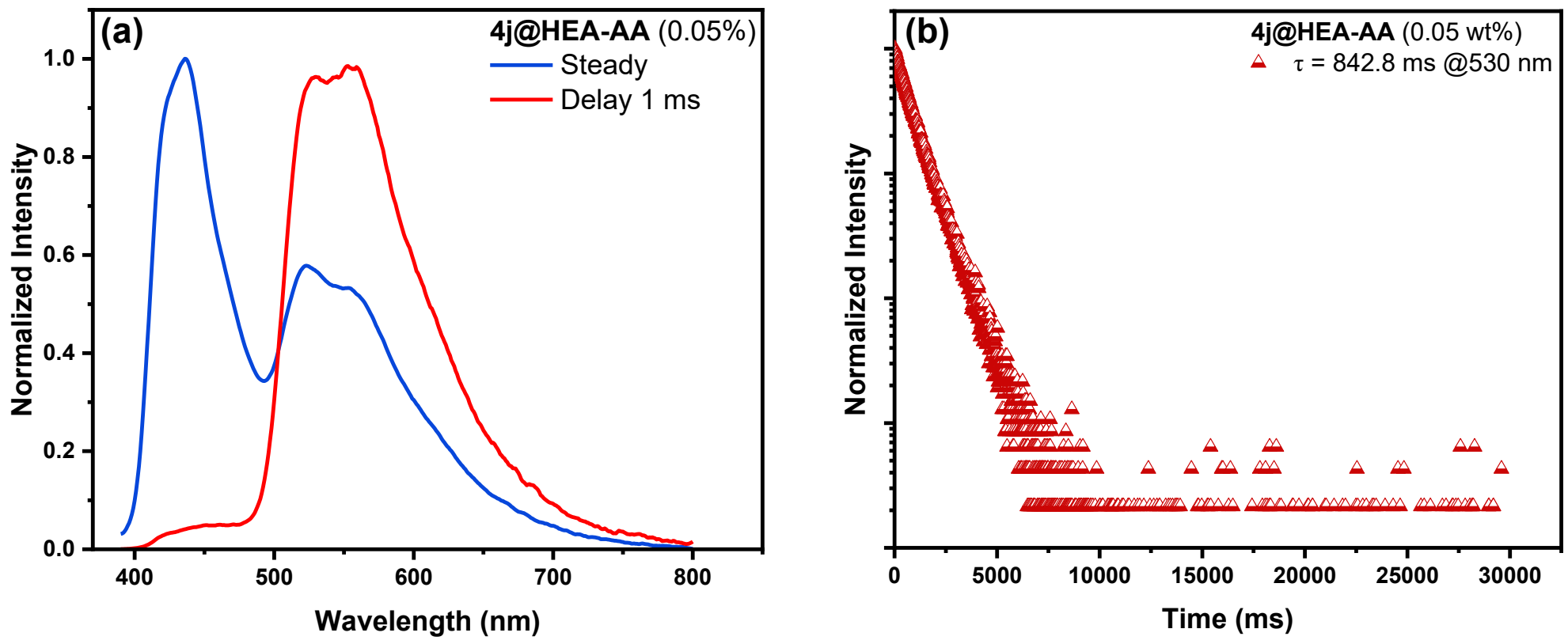

Fig. S28 (a) Steady-state FL and delayed PL spectra of **4j@HEA-AA** (0.05 wt%). (b) The time resolved PL-decay curve for phosphorescence at 530 nm of **4j@HEA-AA** (0.05 wt%). The weight ratio of HEA and AA is HEA/AA = 7/3. Fluorescence Spectrometer: Edinburgh FLS1000. λ_{ex} = 365 nm. Irradiation condition: UV 365 nm, 30 s.

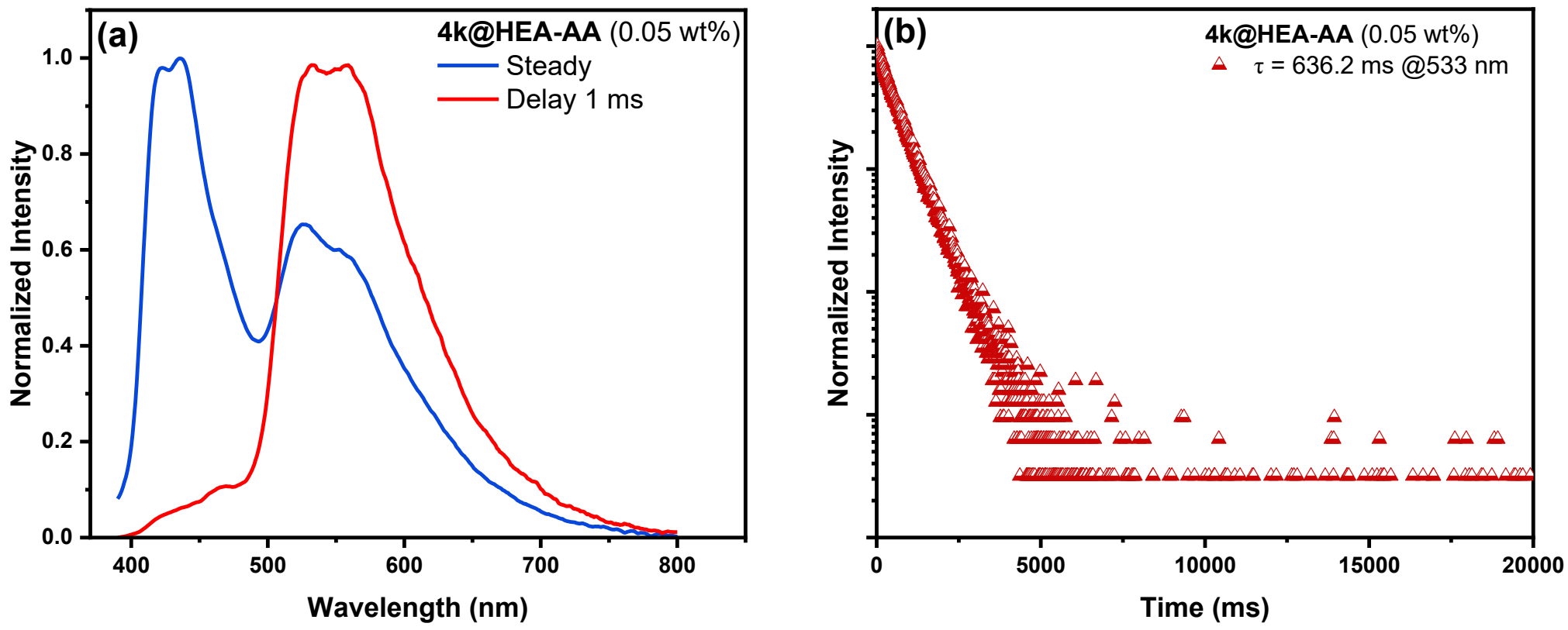

Fig. S29 (a) Steady-state FL and delayed PL spectra of **4k@HEA-AA** (0.05 wt%). (b) The time resolved PL-decay curve for phosphorescence at 533 nm of **4k@HEA-AA** (0.05 wt%). The weight ratio of HEA and AA is HEA/AA = 7/3. Fluorescence Spectrometer: Edinburgh FLS1000. λ_{ex} = 365 nm. Irradiation condition: UV 365 nm, 30 s.

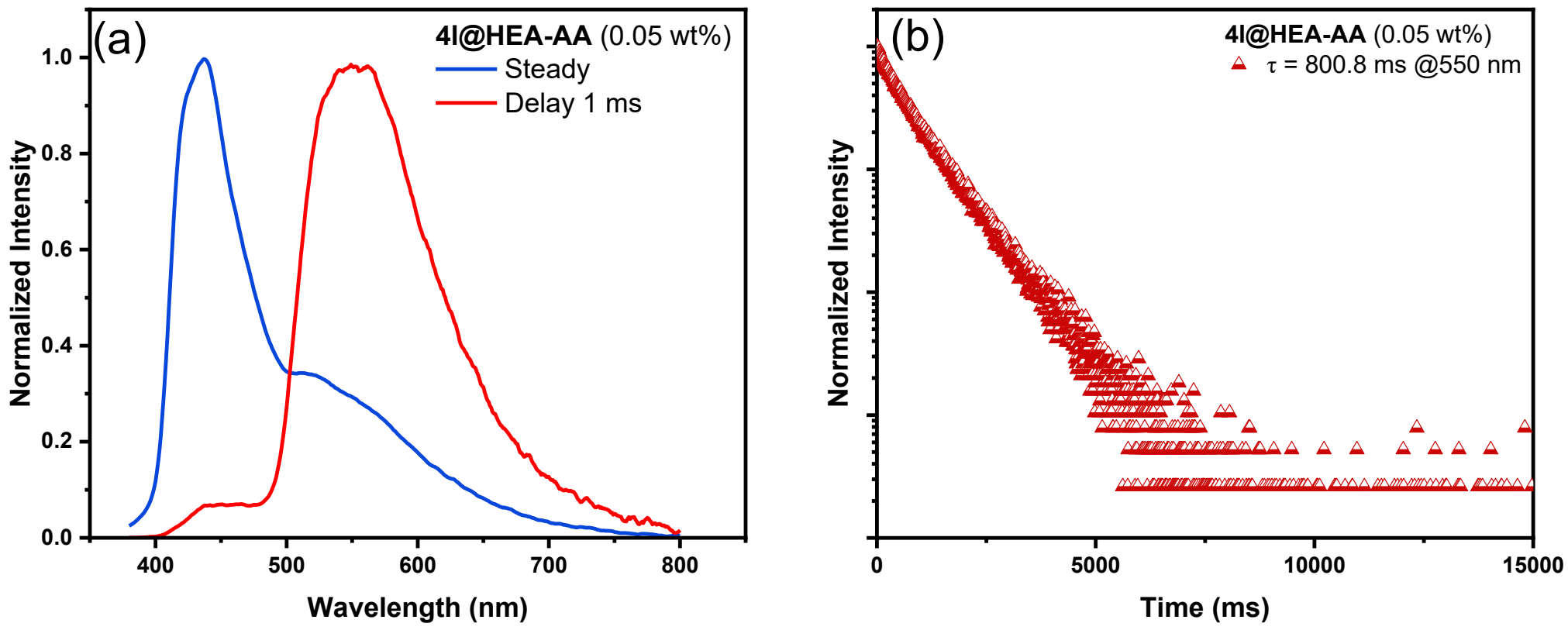

Fig. S30 (a) Steady-state FL and delayed PL spectra of **4l@HEA-AA** (0.05 wt%). (b) The time resolved PL-decay curve for phosphorescence at 550 nm of **4l@HEA-AA** (0.05 wt%). The weight ratio of HEA and AA is HEA/AA = 7/3. Fluorescence Spectrometer: Edinburgh FLS1000. λ_{ex} = 365 nm. Irradiation condition: UV 365 nm, 30 s.

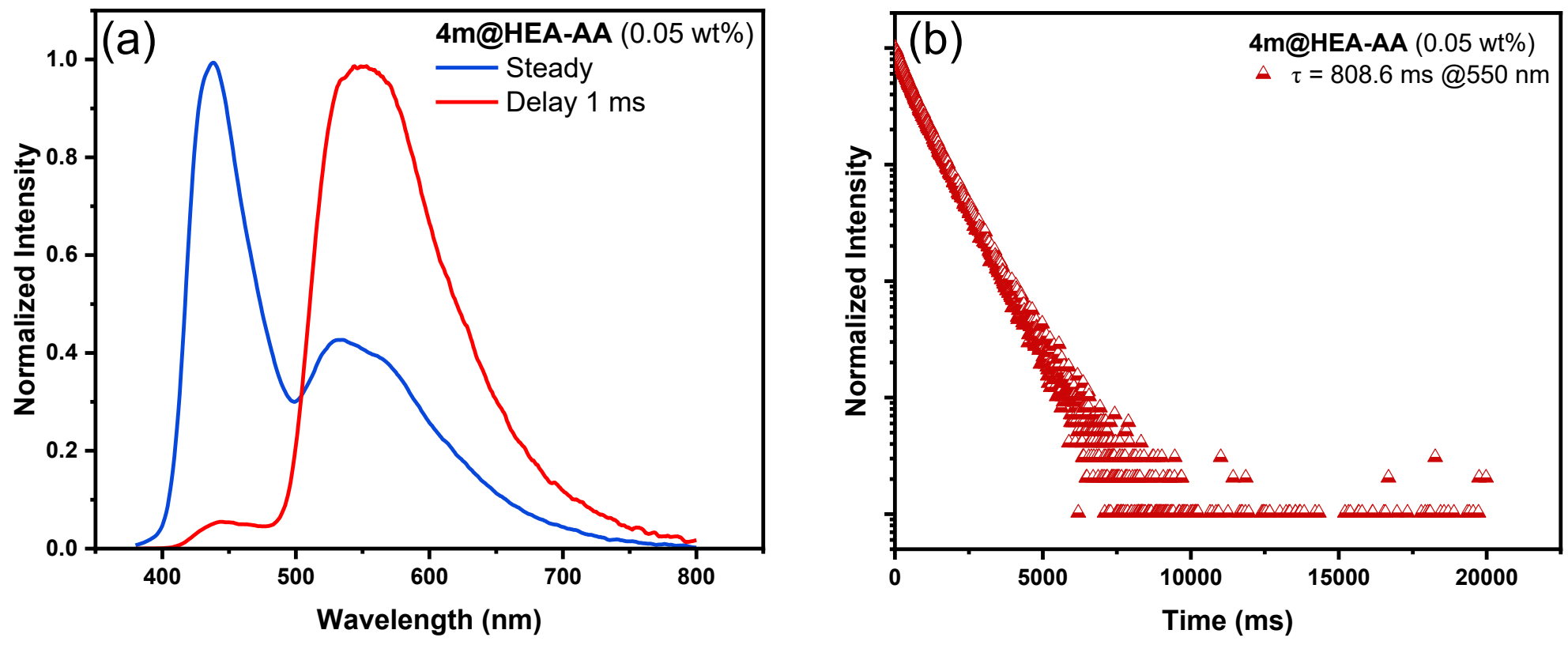

Fig. S31 (a) Steady-state FL and delayed PL spectra of **4m@HEA-AA** (0.05 wt%). (b) The time resolved PL-decay curve for phosphorescence at 550 nm of **4m@HEA-AA** (0.05 wt%). The weight ratio of HEA and AA is HEA/AA = 7/3. Fluorescence Spectrometer: Edinburgh FLS1000. λ_{ex} = 365 nm. Irradiation condition: UV 365 nm, 30 s.

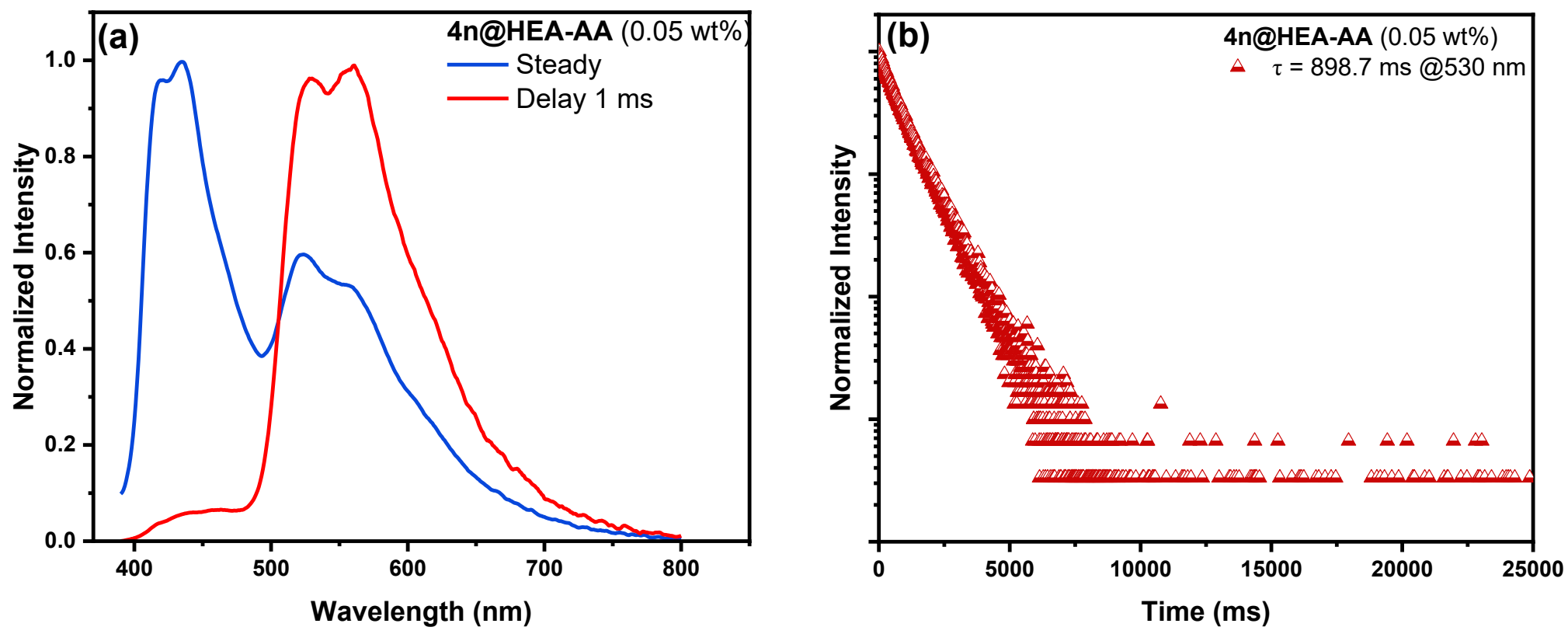

Fig. S32 (a) Steady-state FL and delayed PL spectra of **4n@HEA-AA** (0.05 wt%). (b) The time resolved PL-decay curve for phosphorescence at 530 nm of **4n@HEA-AA** (0.05 wt%). The weight ratio of HEA and AA is HEA/AA = 7/3. Fluorescence Spectrometer: Edinburgh FLS1000. λ_{ex} = 365 nm. Irradiation condition: UV 365 nm, 30 s.

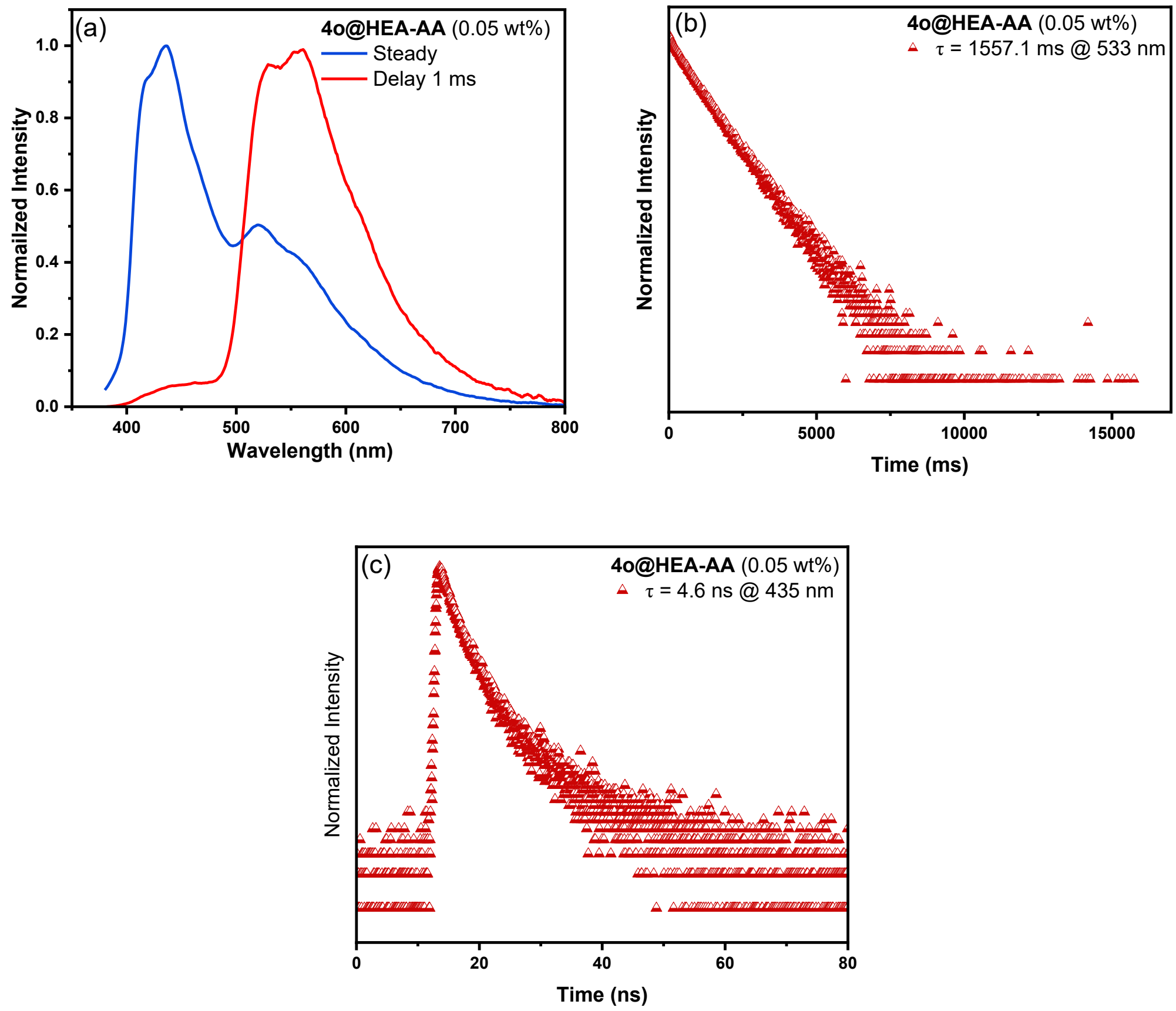

Fig. S33 (a) Steady-state FL and delayed PL spectra of **4o@HEA-AA** (0.05 wt%). (b) The time resolved PL-decay curve for phosphorescence at 533 nm of **4o@HEA-AA** (0.05 wt%). (c) The time resolved FL-decay curve for fluorescence at 435 nm of **4o@HEA-AA** (0.05 wt%). The weight ratio of HEA and AA is HEA/AA = 7/3. Fluorescence Spectrometer: Edinburgh FLS1000. λ_{ex} = 365 nm. Irradiation condition: UV 365 nm, 30 s.

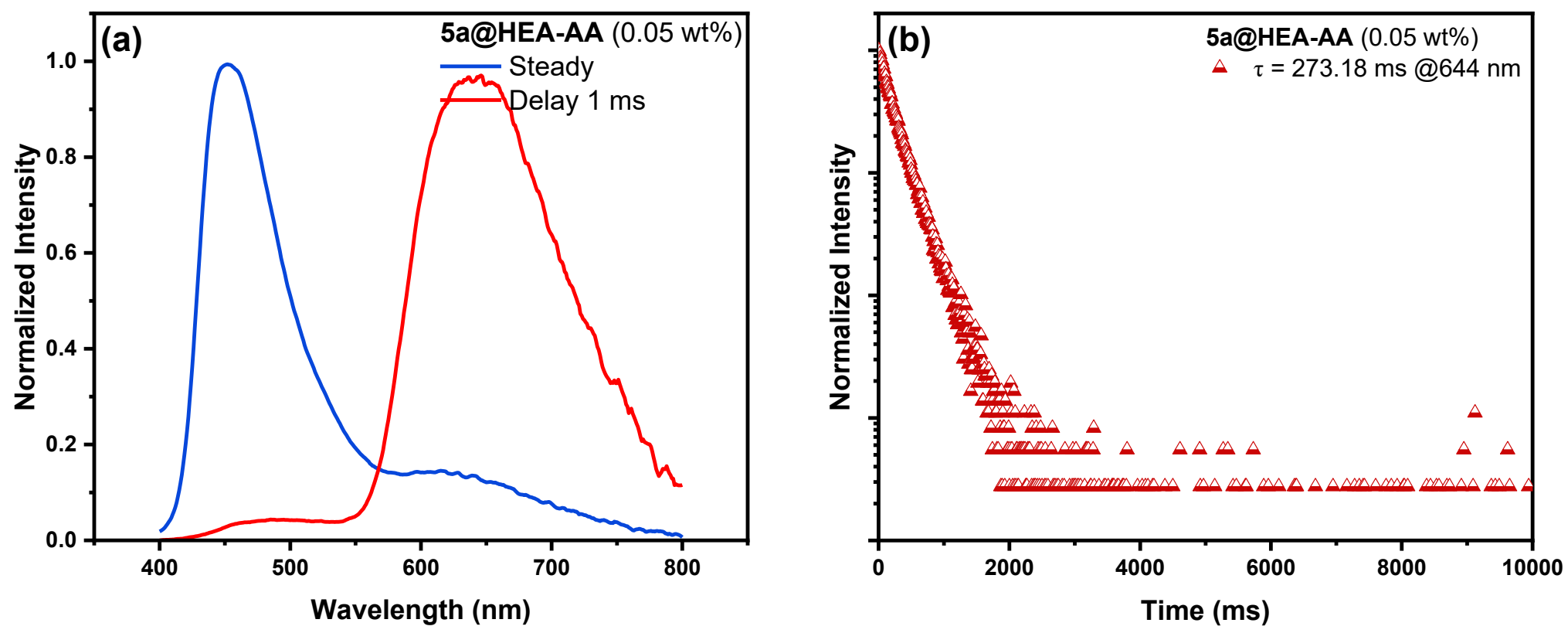

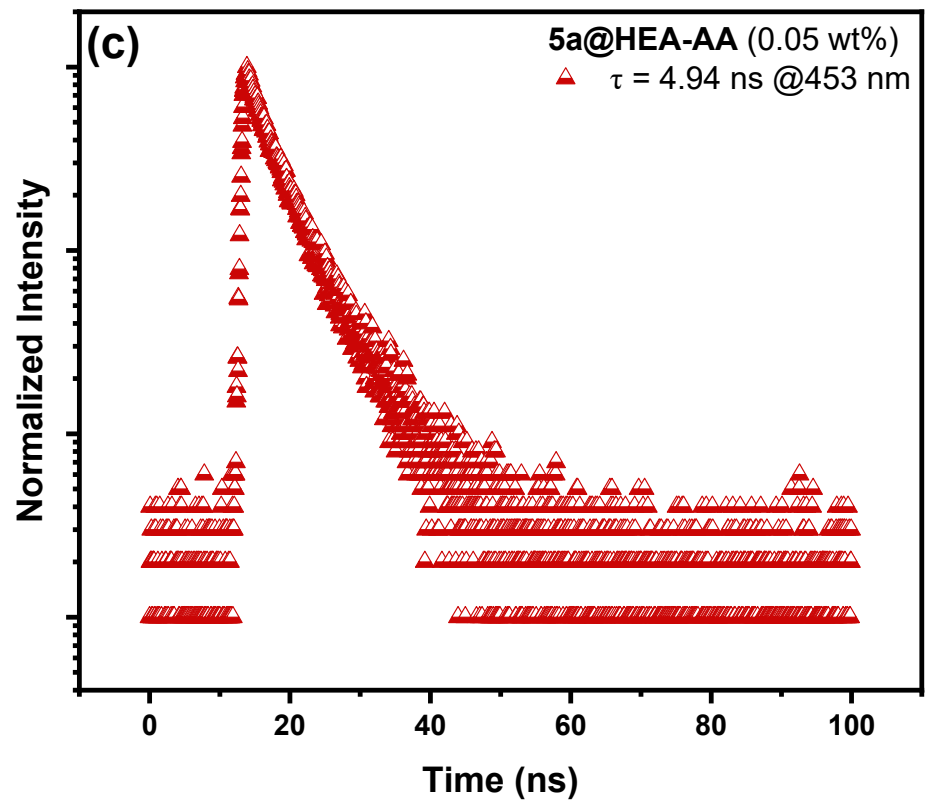

Fig. S34 (a) Steady-state FL and delayed PL spectra of **5a@HEA-AA** (0.05 wt%). (b) The time resolved PL-decay curve for phosphorescence at 644 nm of **5a@HEA-AA** (0.05 wt%). (c) The time resolved FL-decay curve for fluorescence at 453 nm of **5a@HEA-AA** (0.05 wt%). Fluorescence Spectrometer: Edinburgh FLS1000 and HITACHI F-7000. λ_{ex} = 365 nm. Irradiation condition: UV 365 nm, 30 s.

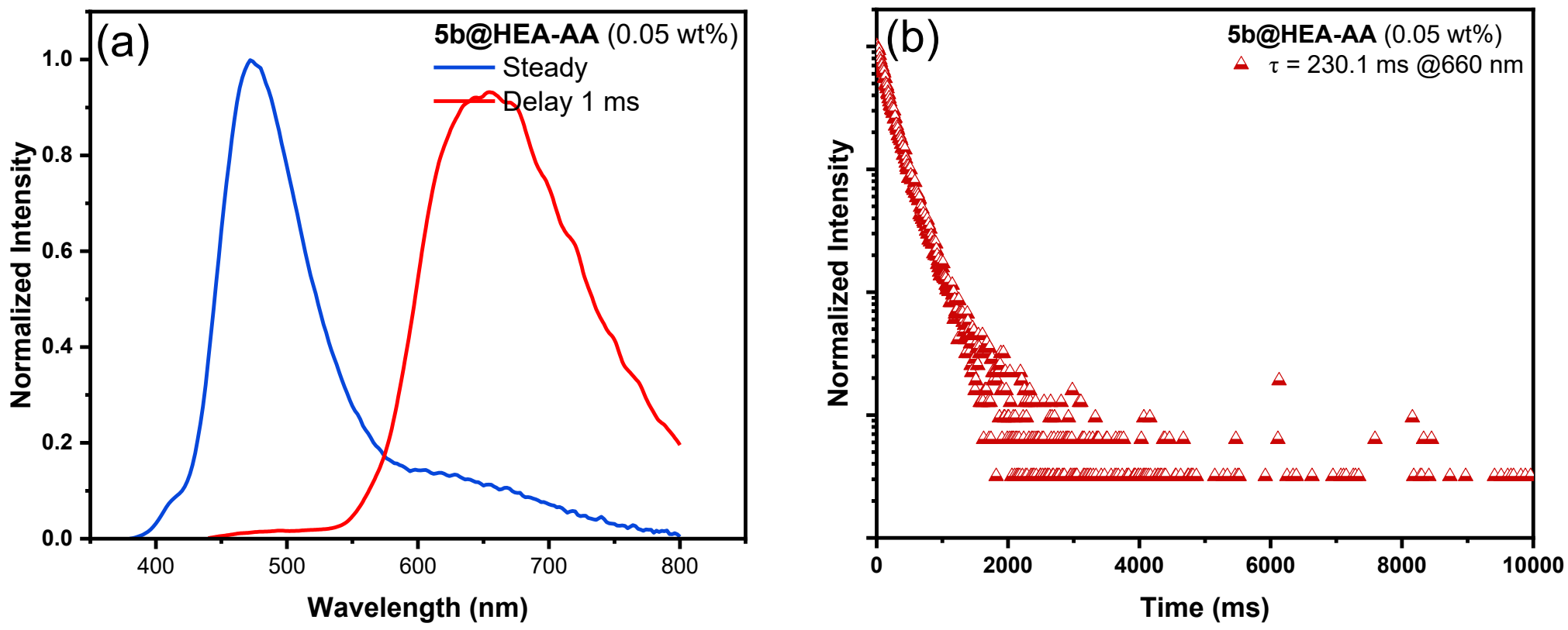

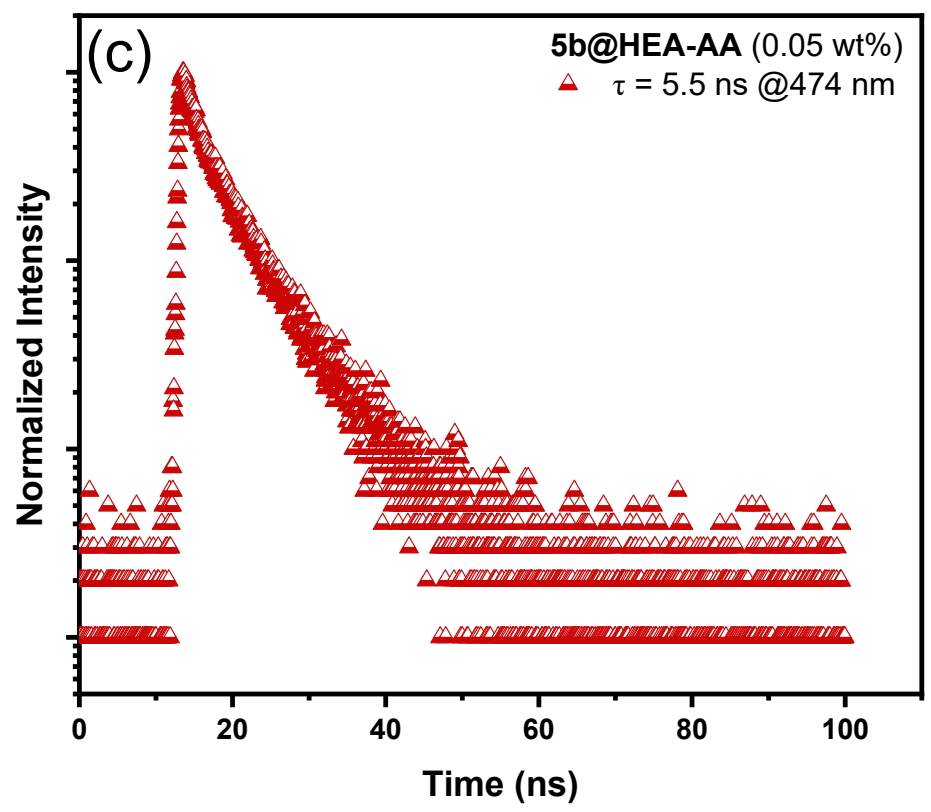

Fig. S35 (a) Steady-state FL and delayed PL spectra of **5b@HEA-AA** (0.05 wt%). (b) The time resolved PL-decay curve for phosphorescence at 660 nm of **5b@HEA-AA** (0.05 wt%). (c) The time resolved FL-decay curve for fluorescence at 474 nm of

5b@HEA-AA (0.05 wt%). Fluorescence Spectrometer: Edinburgh FLS1000 and HITACHI F-7000. Steady-state FL spectra was collected with λ_{ex} = 365 nm, delayed PL spectra was collected with λ_{ex} = 420 nm. Irradiation condition: UV 365 nm, 30 s.

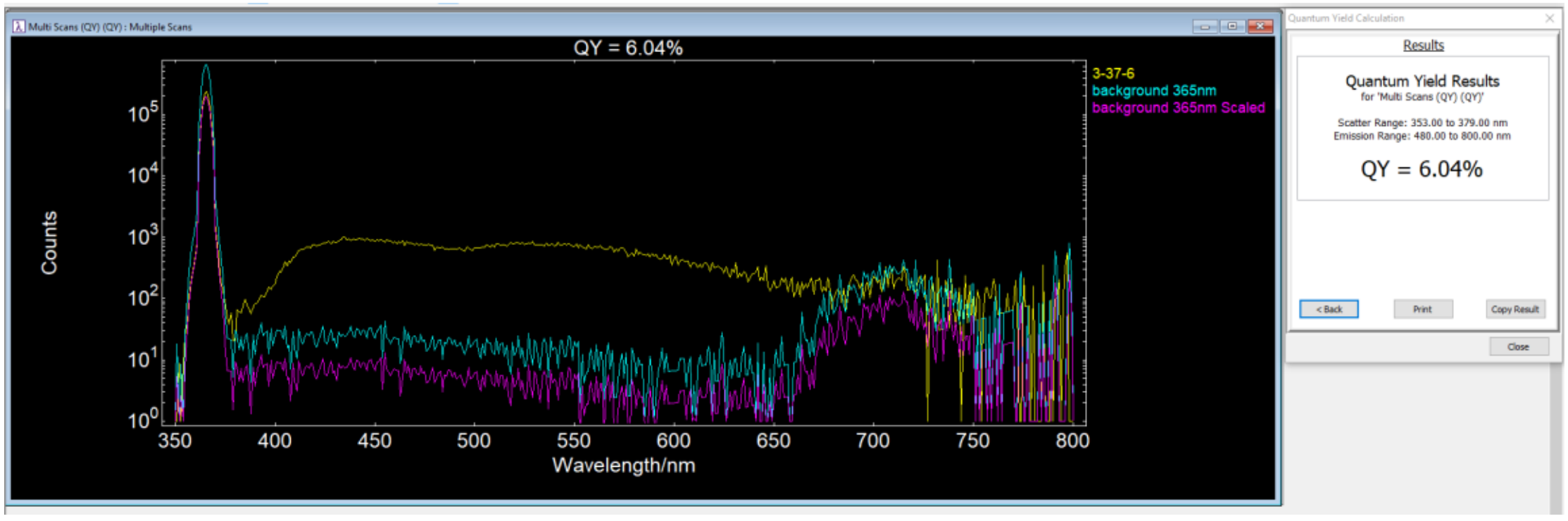

Fig. S36 The absolute phosphorescence quantum yield of **4o@HEA-AA** (0.05 wt%).

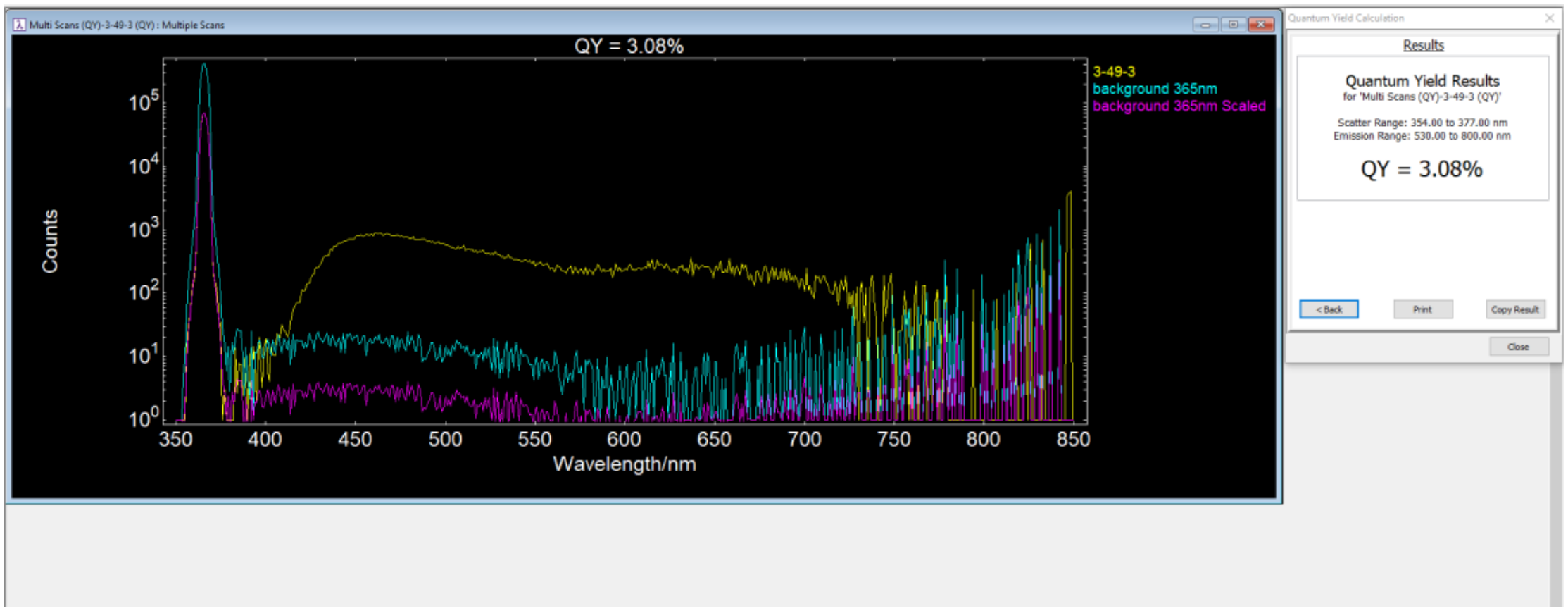

Fig. S37 The absolute phosphorescence quantum yield of **5a@HEA-AA** (0.05 wt%).

3.3. UV-Vis absorption spectra of 4a-5b@PMMA or HEA-AA films

UV−vis spectra were recorded on Agilent Cary60 spectrometer. In a typical experiment, a PMMA film doped with desired phenothiazine-besed polycyclic aromatic hydrocarbons (1.0 wt%) for the collection of UV-vis spectrum were prepared respectively.

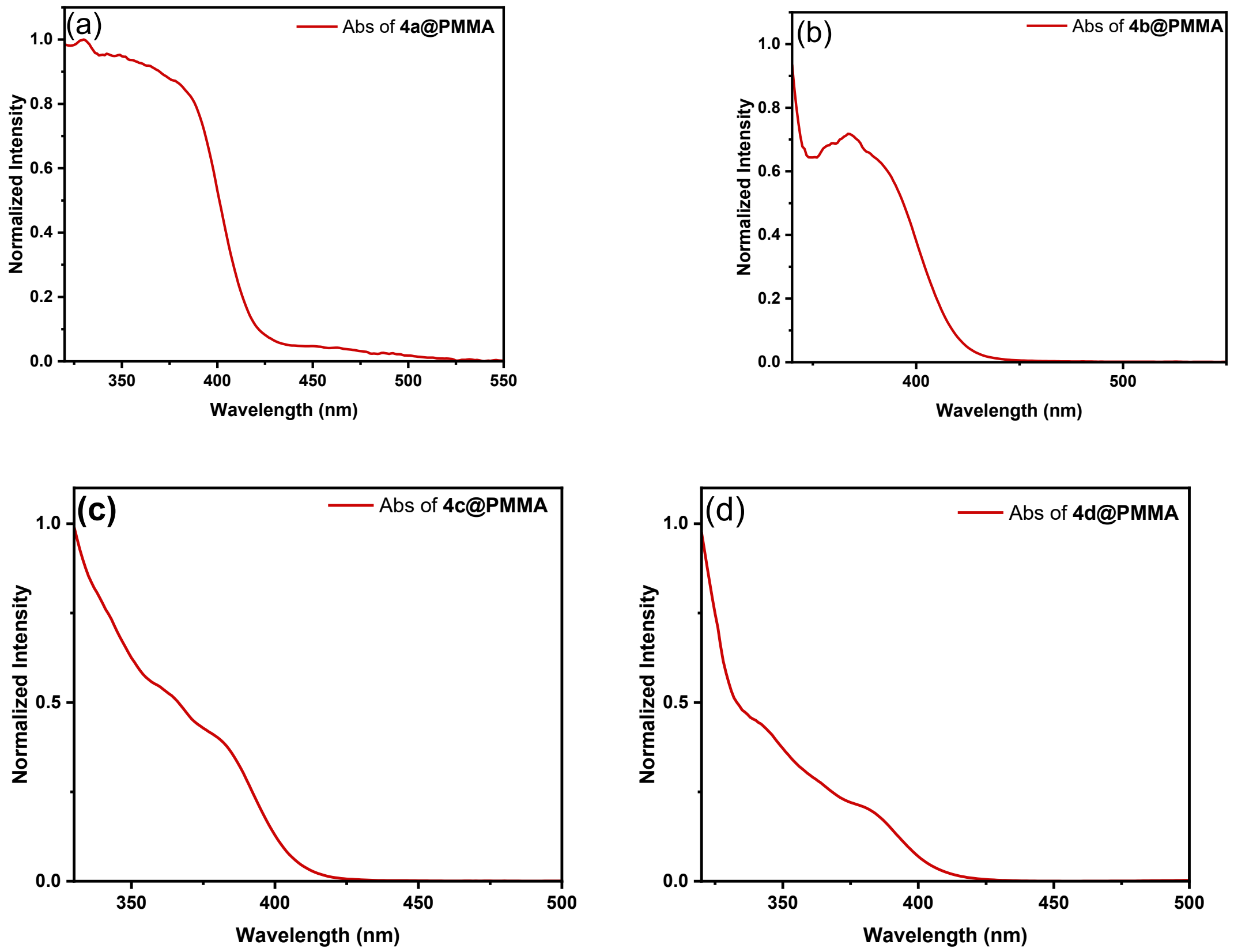

Fig. S38 UV-Vis absorption spectra of **4a-4d@PMMA** (1.0 wt%).

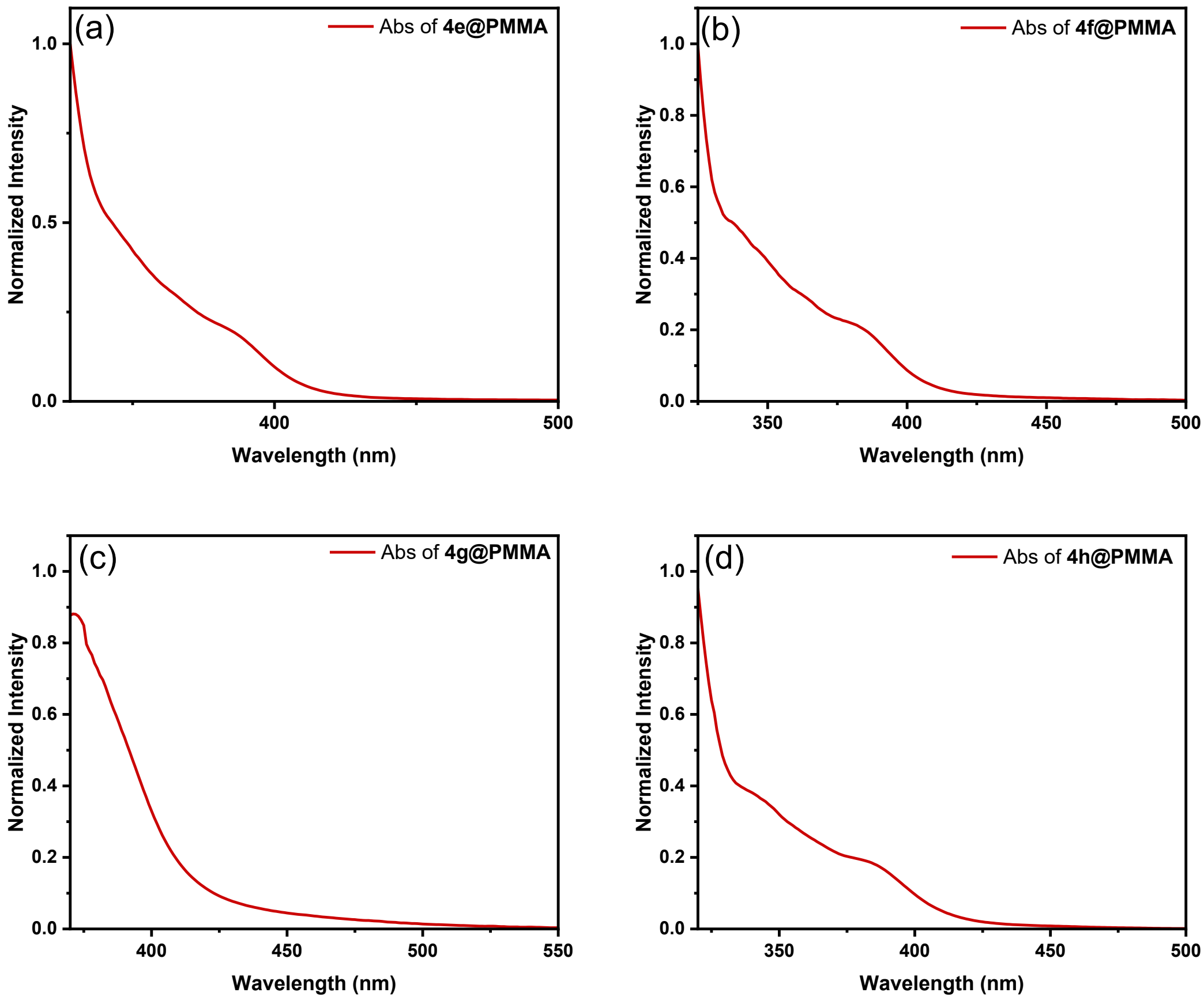

Fig. S39 UV-Vis absorption spectra of **4e-4h@PMMA** (1.0 wt%).

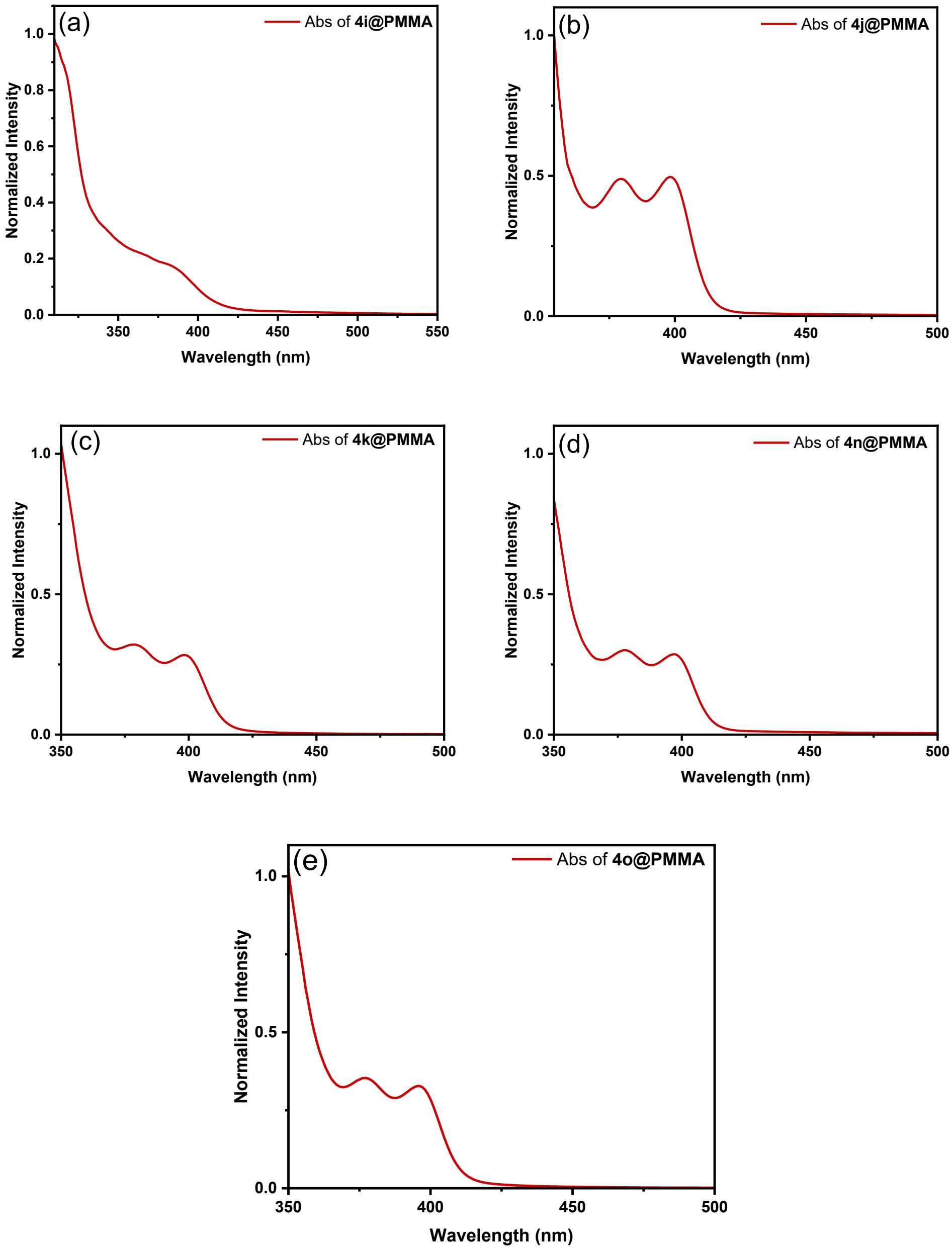

Fig. S40 UV-Vis absorption spectra of **4i-4o@PMMA** (1.0 wt%).

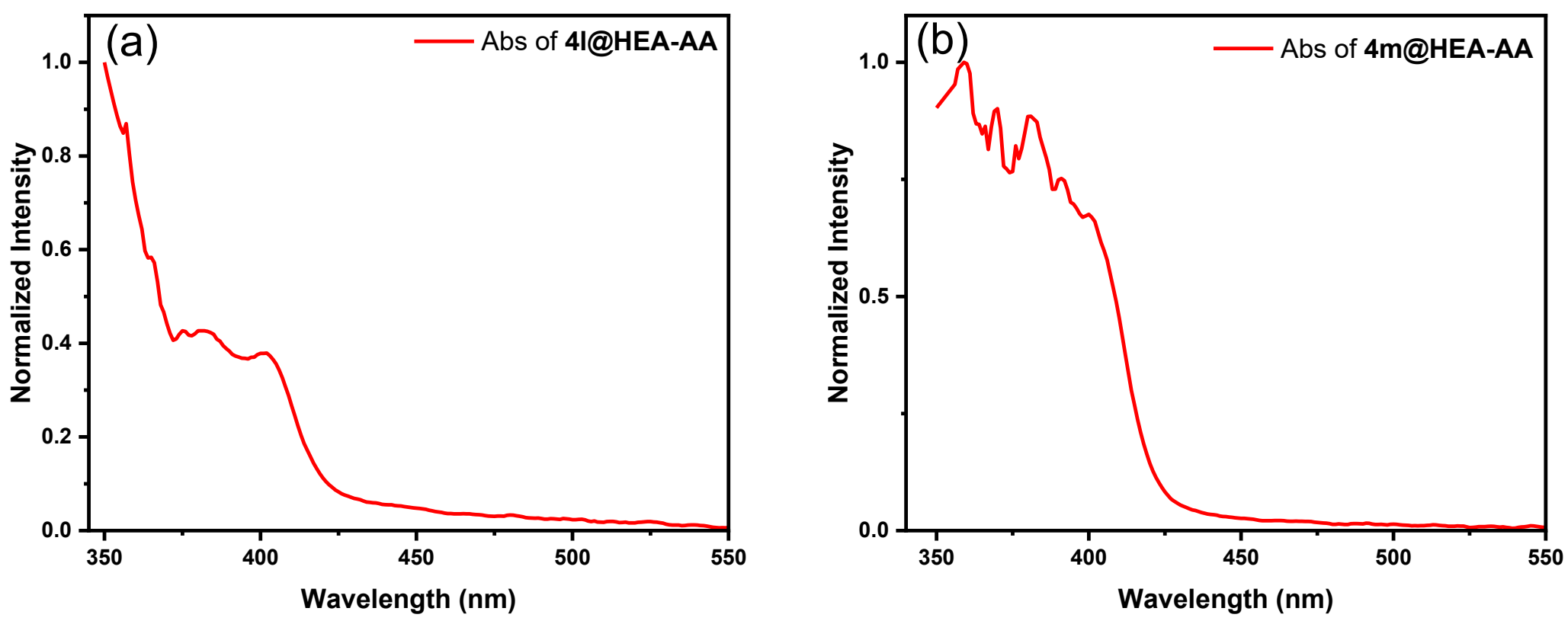

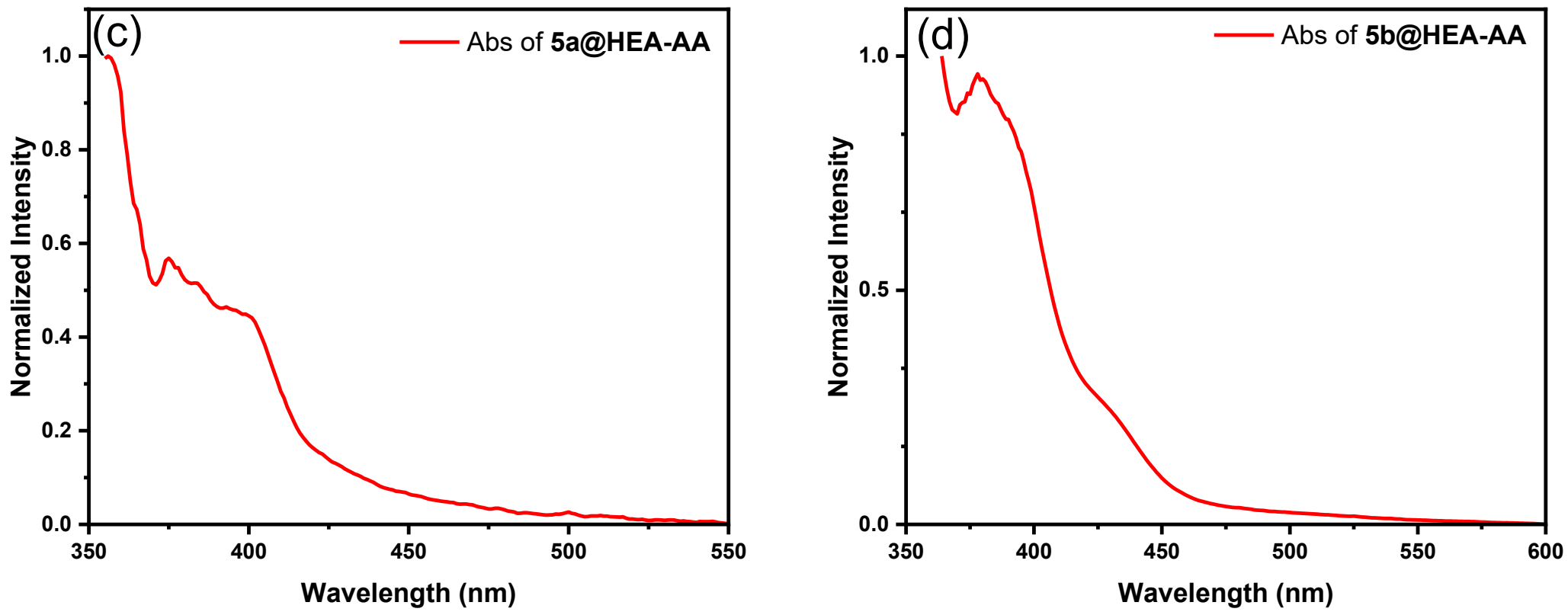

Fig. S41 UV-Vis absorption spectra of **4l**, **4m**, **5a and 5b@HEA-AA** (0.05 wt%).

3.4. Photophysical properties of 4a, 4c, 4d, 4j, 4o and 5a in solvent.

Fluorescence spectra were collected on HITACHI F-700003040428 fluorescence spectrometer In a typical experiment, the solvent of product in toluene, DCM or MeCN were prepared for the collection of emission spectrum respectively.

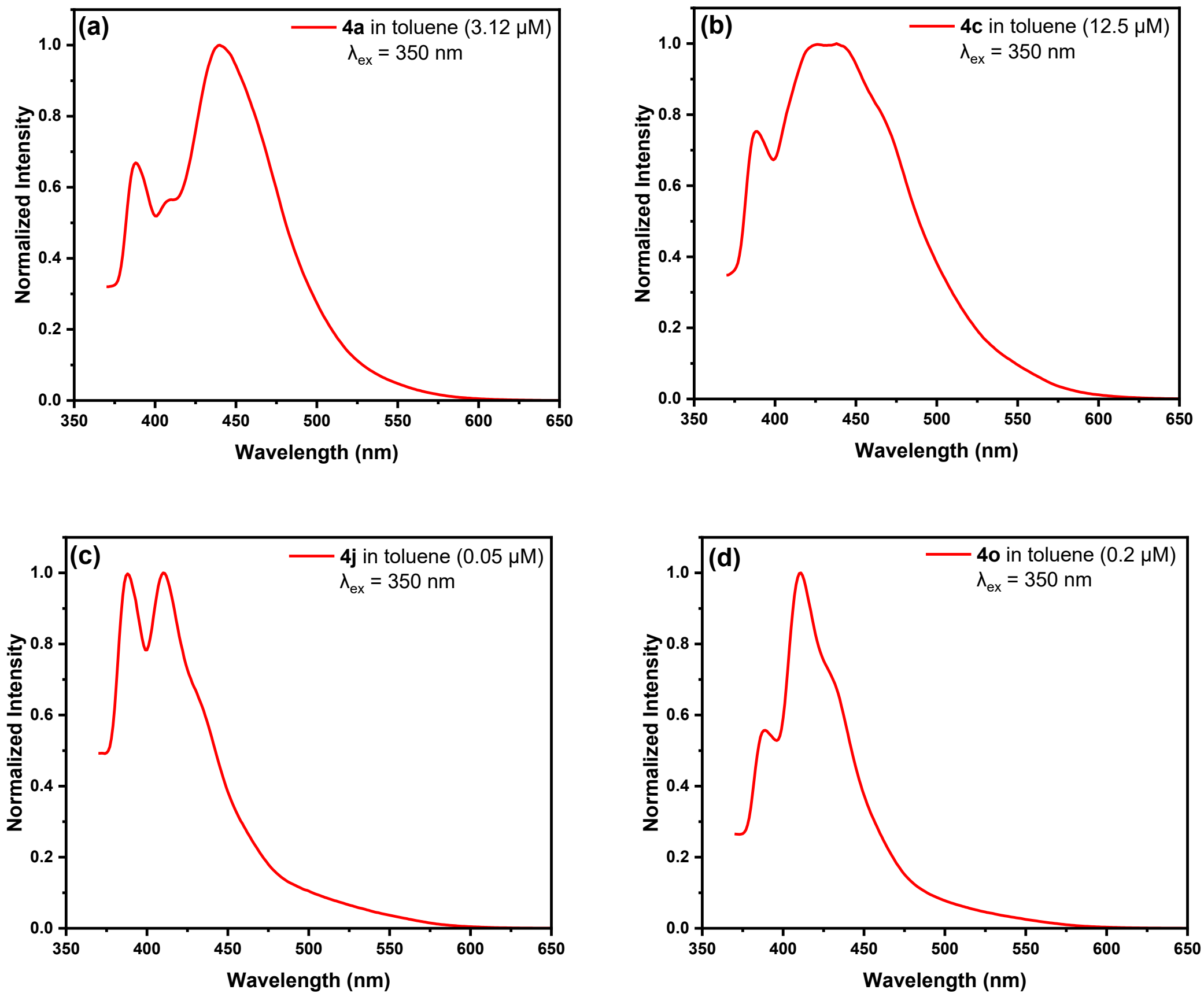

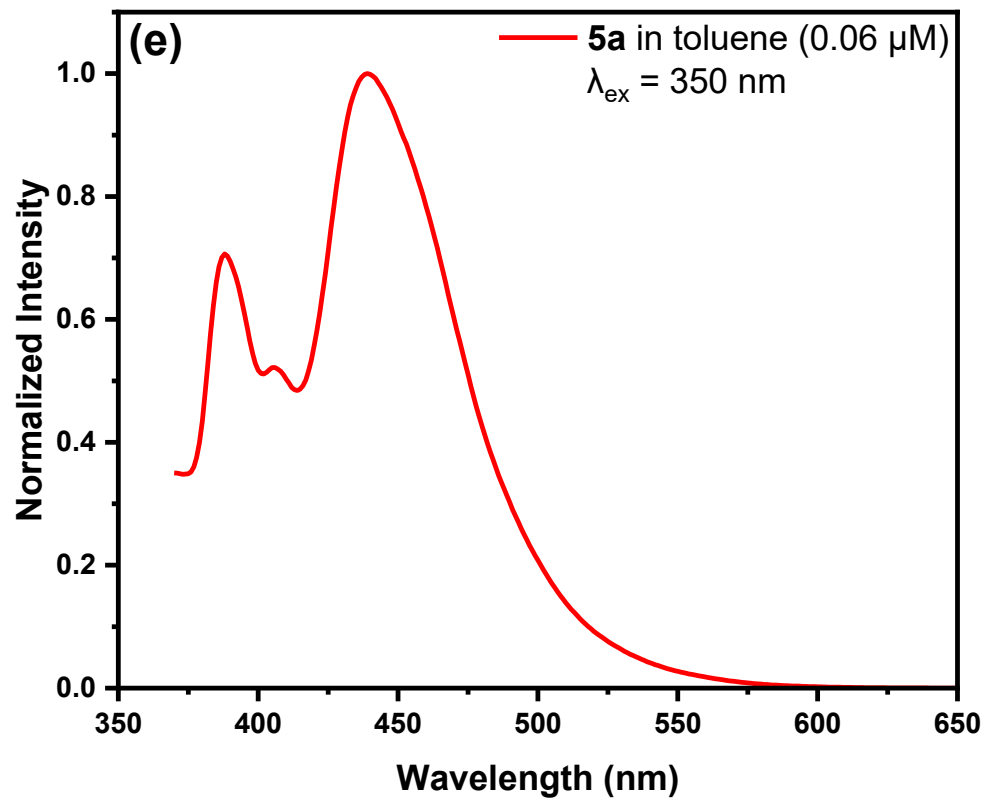

Fig. S42 (a) Normalized fluorescence emission spectra of **4a** in toluene (3.12 μM). (b) Normalized fluorescence emission spectra of **4c** in toluene (12.5 μM). (c) Normalized fluorescence emission spectra of **4j** in toluene (0.05 μM). (d) Normalized fluorescence emission spectra of **4o** in toluene (0.2 μM). (e) Normalized fluorescence emission spectra of **5a** in toluene (0.06 μM).

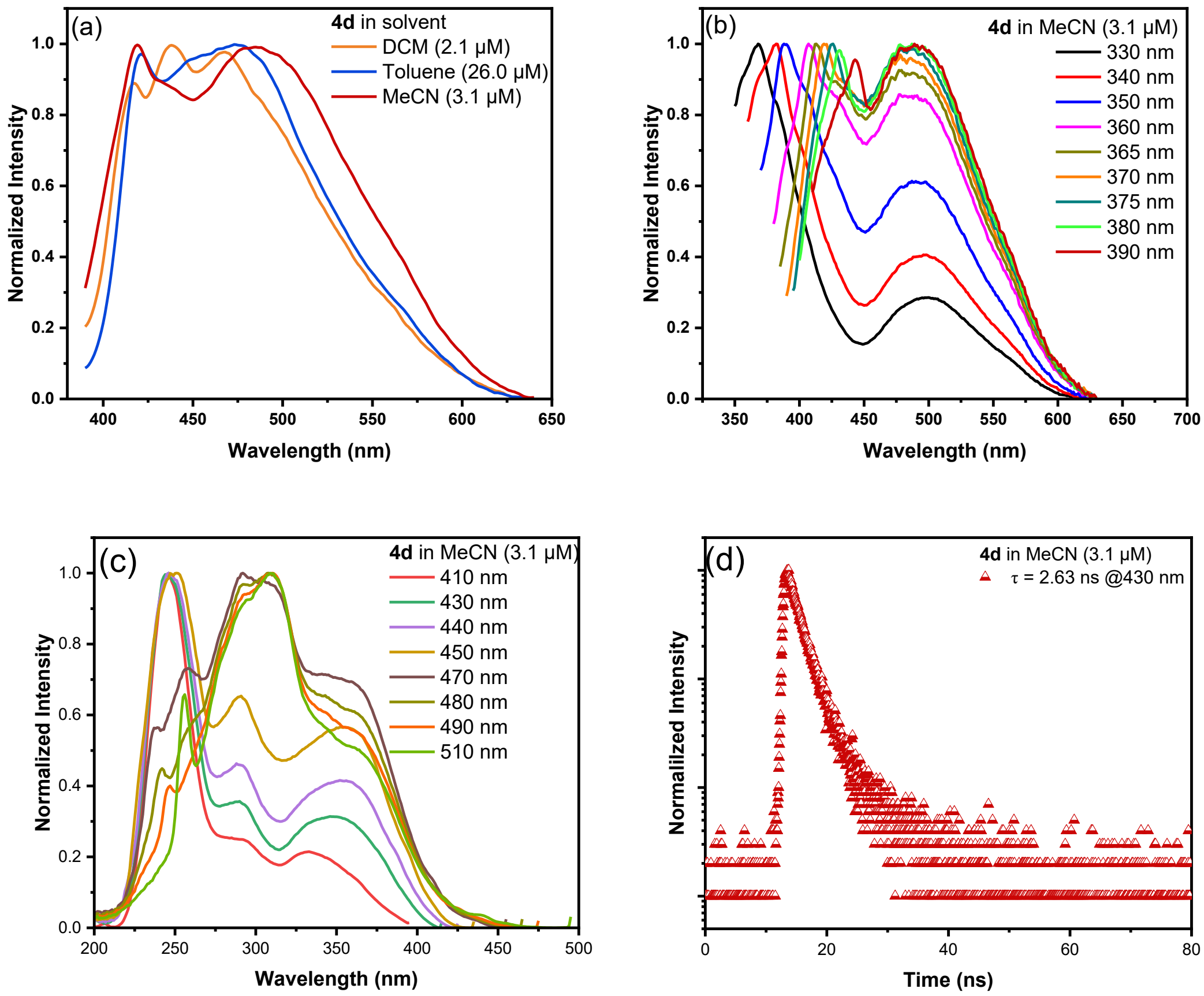

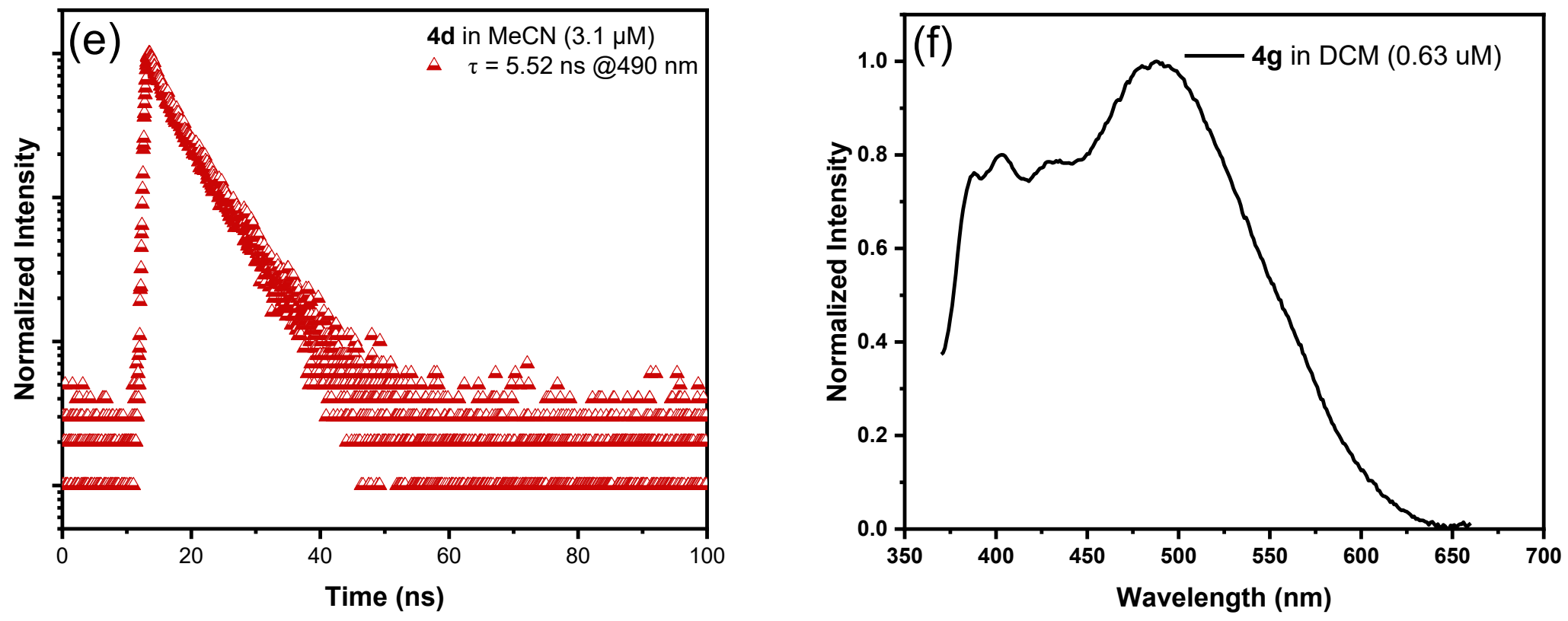

Fig. S43 (a) Normalized fluorescence emission spectra of **4d** in different solvents. (b) Excitation-wavelength-dependent fluorescence spectra of **4d** in MeCN (3.1 μM). Excitation slit: 5.0 nm, Emission slit: 10.0 nm. PMT Voltage = 700 V. (c) Emission-wavelength-dependent excitation spectra of **4d** (3.1 μM in MeCN). Excitation slit: 5.0 nm, Emission slit: 10.0 nm. PMT Voltage = 500 V. (d) The time resolved FL-decay curve for fluorescence at 430 nm of **4d** in MeCN (3.1 μM). (e) The time resolved FL-decay curve for fluorescence at 490 nm of **4d** in MeCN (3.1 μM). (f) Normalized fluorescence emission spectra of **4g** in DCM (0.63 μM).

4. Cyclic voltammogram experiment of 4j

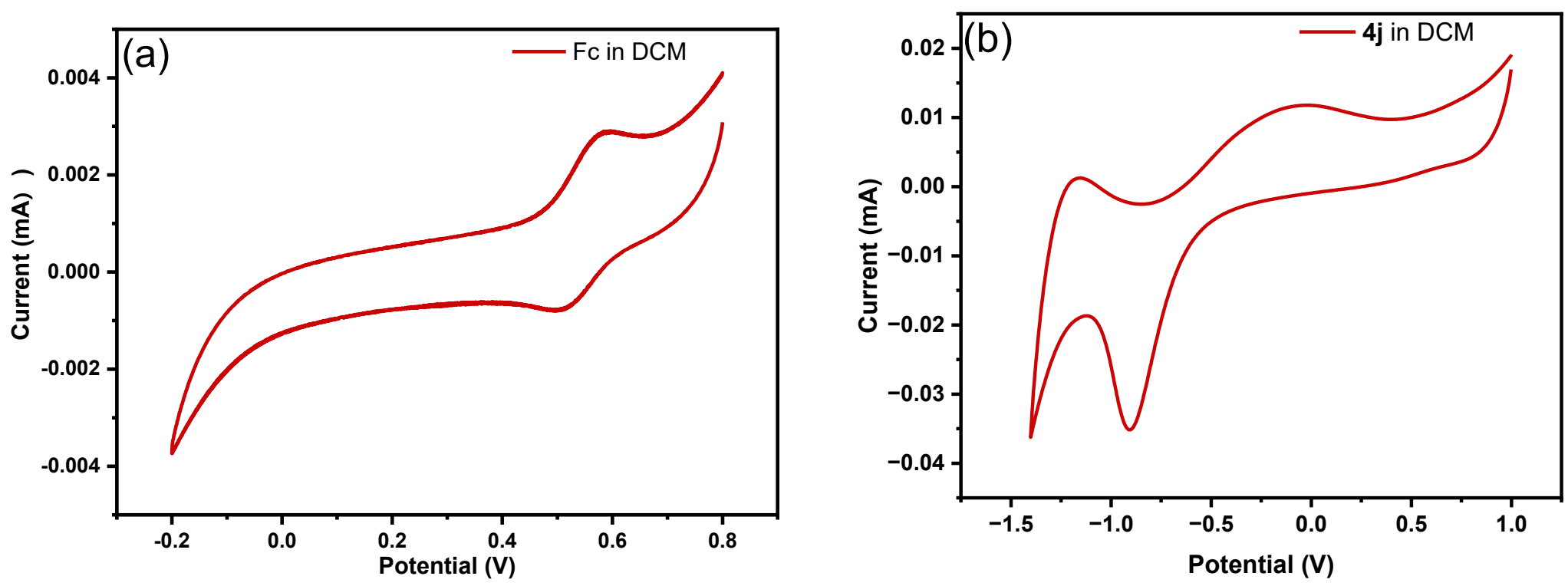

Fig. S44 Cyclic voltammogram of ferrocene (**Fc**) and **4j** in CH_2Cl_2. The oxidation potential of **Fc** and **4j** was determined relative to Ag/Ag+ in the DCM solution (1.0×10^{-4} M).

Table S2. Energy levels of **4j**.

Compound	E_g^{opt} (eV)[a]	E^{red} (eV)[b]	HOMO (eV)[c]	LUMO (eV)[d]
**4j**	2.94	-0.91	-5.25	-3.31

[a] $E_g^{opt} = 1240/\lambda_{onset}$ (eV). [b] Estimated Estimated from the onset voltagels of the cyclic voltammetry in 1.0×10^{-4} M DCM solution. [c] HOMO = LUMO - E_g^{opt} (eV). [d] LUMO = $-[E^{red} - E(Fc/Fc^{+})+4.8]$ (eV).

5. The thermal properties of 4b

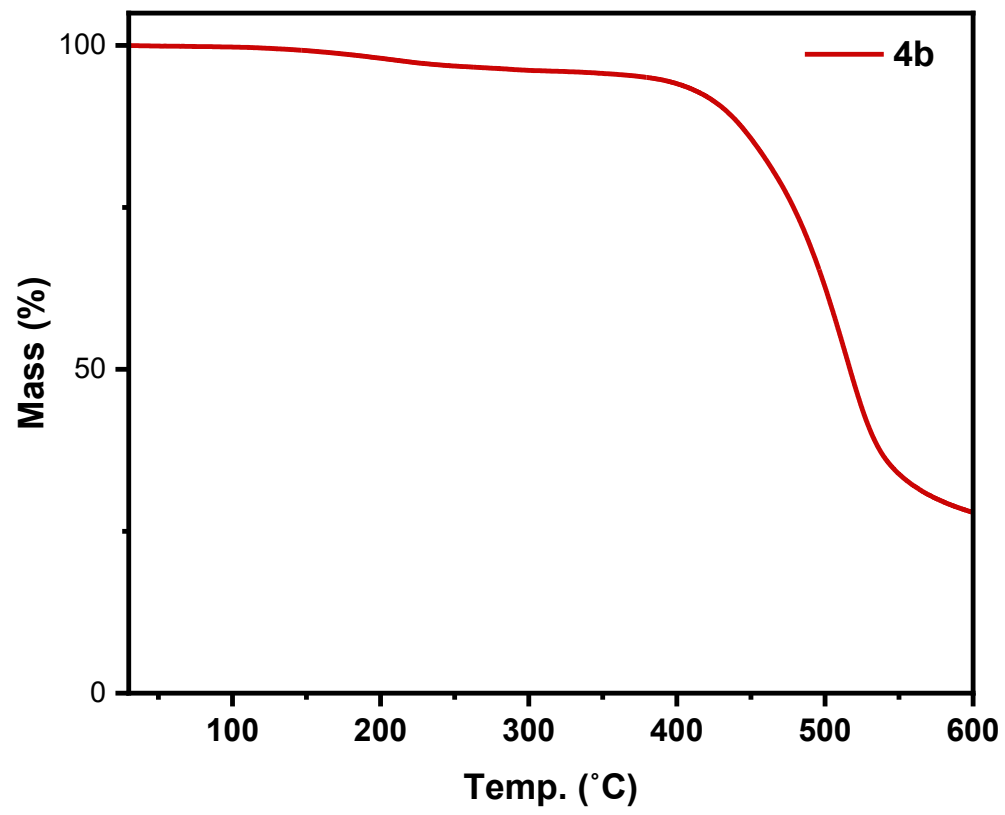

Fig. S45 The thermogravimetric analyzer (TGA) curves of **4b**. Thermo gravimetric analyzer: Mettler Toledo 21007792. Sample: 0.98 mg.

6. The PXRD properties of 4o crystal and film.

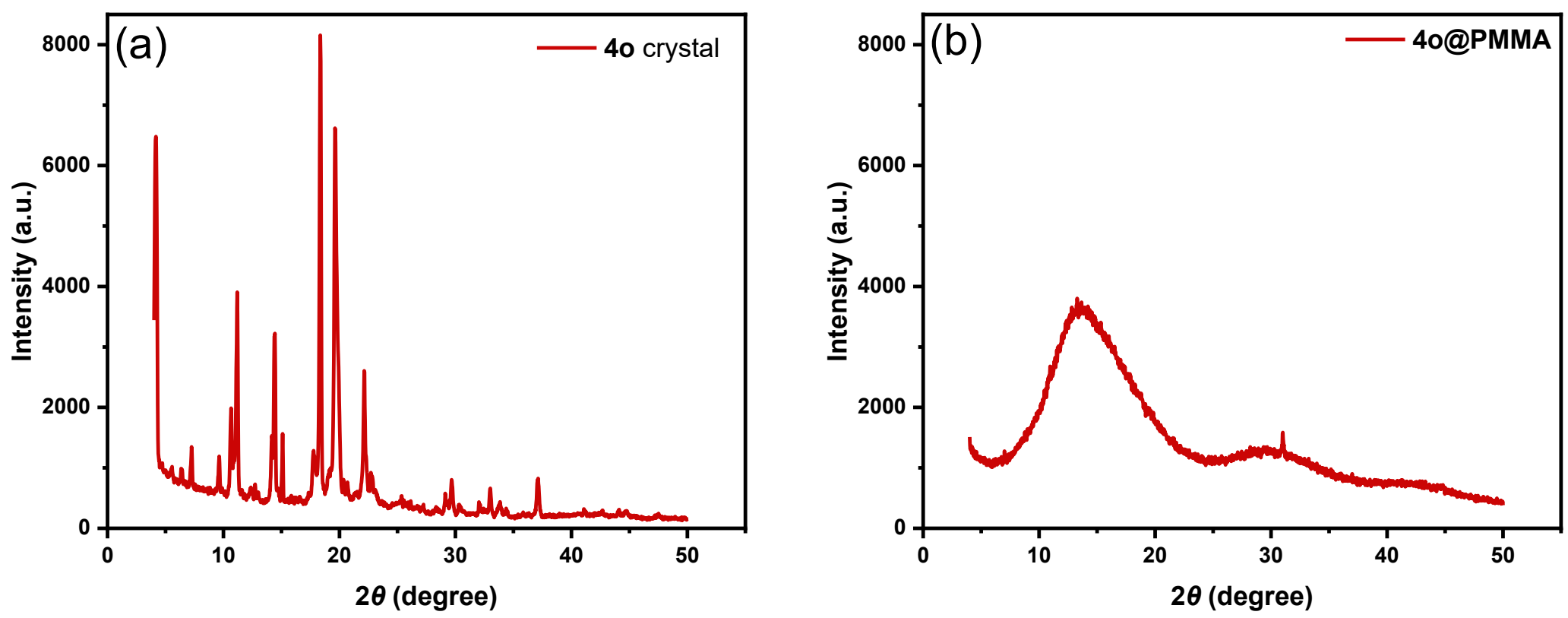

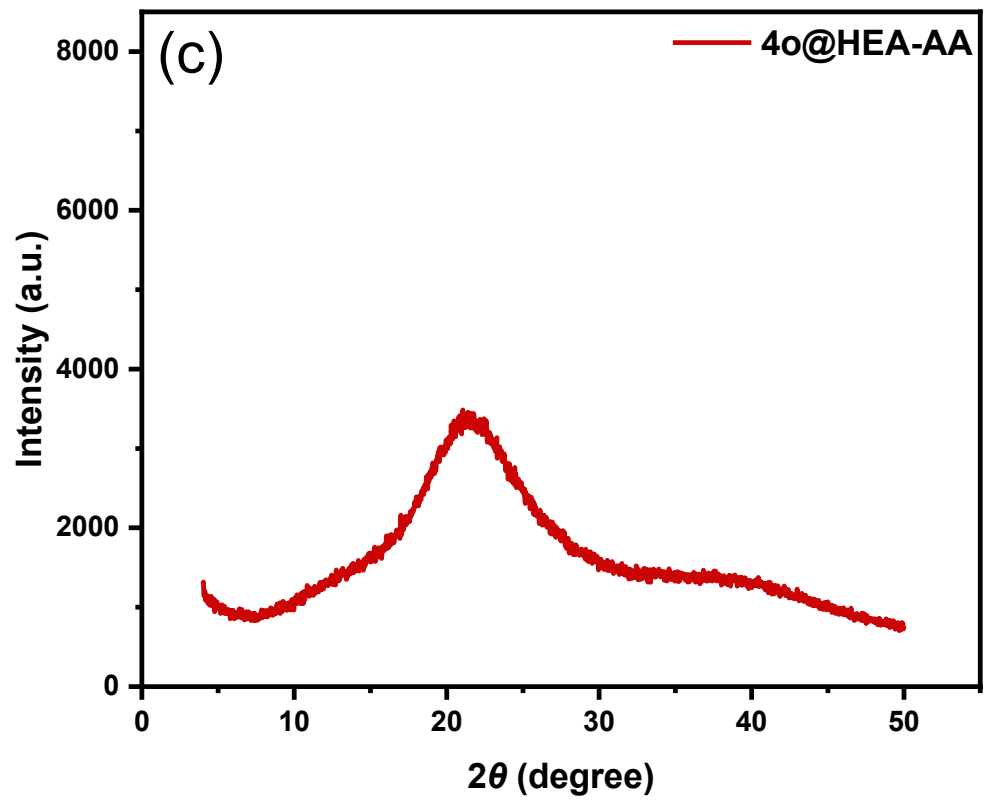

Fig. S46 The PXRD spectrum of **4o** at different states. (a) Crystal. (b) In PMMA film. (c) In HEA-AA film. X-ray powder diffractometer: PANalytical X'Pert Powder.

7. Calculation

Theoretical calculation methods: Time-dependent density functional theory (TDDFT) calculations were worked out using B3LYP/6-31G(d, p) by the Gaussian 16 (Revision A.03) package to optimize the geometries of the lowest singlet states (S_1) for these compounds. The natural transition orbitals (NTOs) of the excited states were evaluated using the dominant "hole-electron" distributions[S6, S7]. The vertical excitation energies and NTOs of singlet states were calculated based on B3LYP/6–31G(d, p), and the vertical excitation energies and NTOs of triplet states were calculated based on TDA–B3LYP/6–31G(d, p). The spin–orbit coupling (SOC) coefficients were quantitatively estimated using B3LYP/6–31G(d, p) by a Beijing density function (BDF) program (Version 2024A)[S8-S12]. The results show that for compound **4a**, the experimental S_1 and T_1 energies are 2.81 eV (441 nm) and 2.34 eV (529 nm), respectively, while the theoretical values are 2.78 eV (446.12 nm) and 2.32 eV (534.98 nm). For compound **4c**, the experimental S_1 and T_1 energies are 2.82 eV (440 nm) and 2.34 eV (523 nm), respectively, with corresponding theoretical values of 2.79 eV (444.62 nm) and 2.32 eV (534.11 nm). For compound **PTZ**, the experimental S_1 and T_1 energies are 2.93 eV (423 nm) and 2.39 eV (518 nm), which match the theoretical value of 2.90 eV (427.30 nm) and 2.39 eV (518.48 nm) exactly. These results thus validate the accuracy of the employed computational methods. The theoretical details are provided in Table S3–S6.

Table S3. The calculated fluorescence emission energies based on the optimized geometries of S_1.

Compounds	λ_F (nm)						
	Experimental value	Based on the optimized geometries of S_1 by B3LYP			Based on the optimized geometries of S_1 by M06-2X		
		M06-2X	**B3LYP**	PBE0	M06-2X	B3LYP	PBE0
**PTZ**	423	390.80	**427.30**	418.05	397.44	432.73	423.67
**4a**	441	379.12	**446.12**	430.30	397.17	451.09	438.22
**4c**	440	392.36	**444.62**	432.42	403.32	453.49	441.64

Table S4. The calculated phosphorescence emission energies based on the optimized geometries of S_1.

Compounds	λ_P (nm)						
	Experimental value	Based on the optimized geometries of S_1 by B3LYP			Based on the optimized geometries of S_1 by B3LYP		
		M06-2X	B3LYP	PBE0	TDA-M06-2X	**TDA-B3LYP**	TDA-PBE0
**PTZ**	518	481.26	554.71	571.06	461.85	**518.48**	517.64
**4a**	529	484.24	578.27	601.49	457.36	**534.98**	529.67
**4c**	523	488.08	575.02	595.89	464.40	**534.11**	530.92

Table S5. The calculated phosphorescence emission energies based on the optimized geometries of T_1.

Compounds	λ_P (nm)						
	Experimental value	Based on the optimized geometries of T_1 by TDA-B3LYP			Based on the optimized geometries of T_1 by TDA-M06-2X		
		TDA-M06-2X	TDA-B3LYP	TDA-PBE0	TDA-M06-2X	TDA-B3LYP	TDA-PBE0
**PTZ**	518	468.13	526.39	525.87	474.46	531.77	531.46
**4a**	529	515.64	589.07	590.17	525.33	599.09	600.87
**4c**	523	515.32	587.72	589.71	524.90	597.89	600.49

Table S6. The calculated phosphorescence emission energies based on the optimized geometries of T_1.

Compounds	λ_P (nm)						
	Experimental value	Based on the optimized geometries of T_1 by TDA-B3LYP			Based on the optimized geometries of T_1 by TDA-M06-2X		
		M06-2X	B3LYP	PBE0	M06-2X	B3LYP	PBE0
**PTZ**	518	488.68	564.74	582.39	494.92	569.62	587.13
**4a**	529	559.63	661.17	704.46	571.05	673.95	718.92
**4c**	523	560.20	662.50	707.89	571.46	675.41	722.55

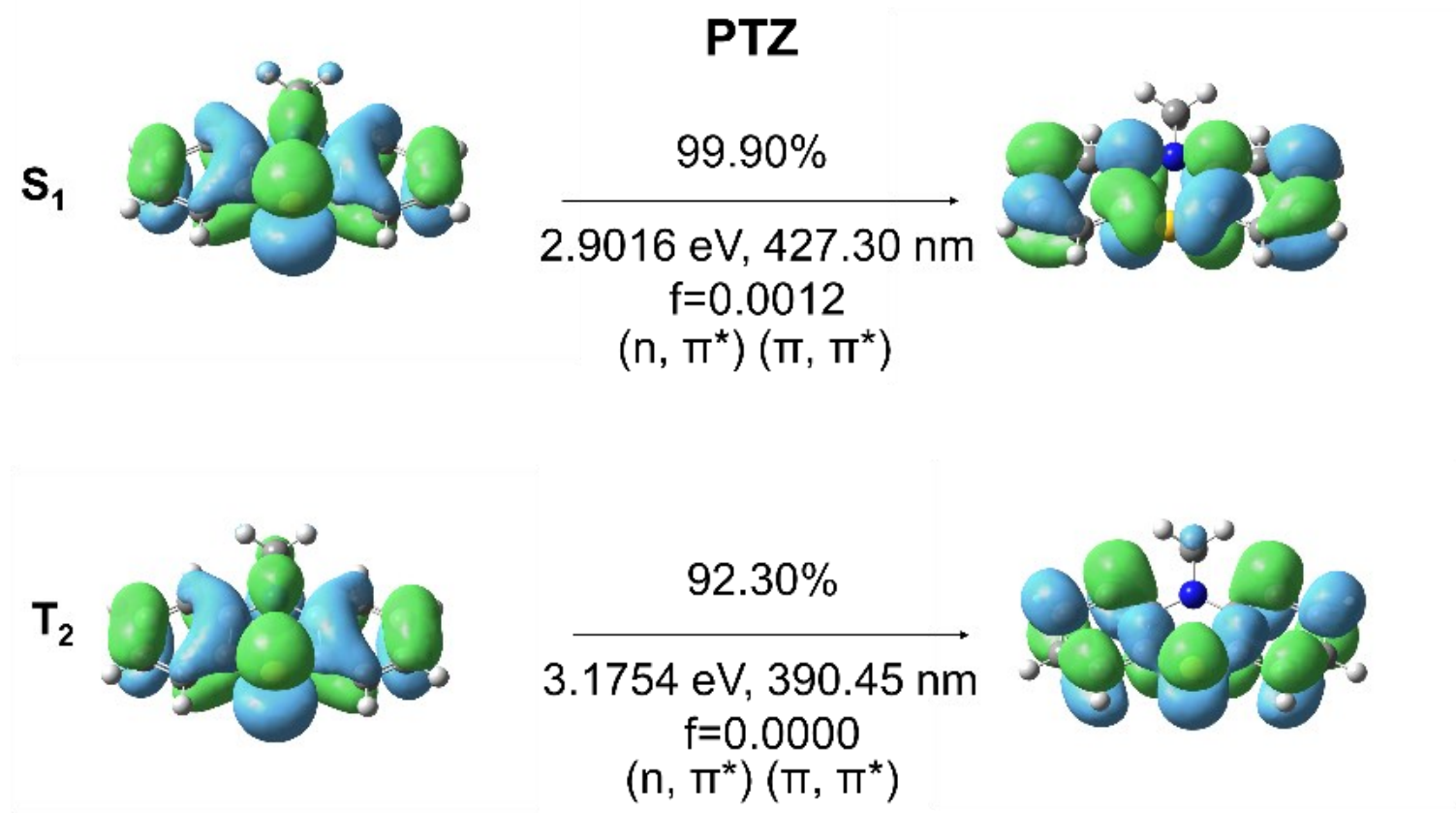

Fig. S47 NTOs based on optimized geometries of S_1 for **PTZ**.

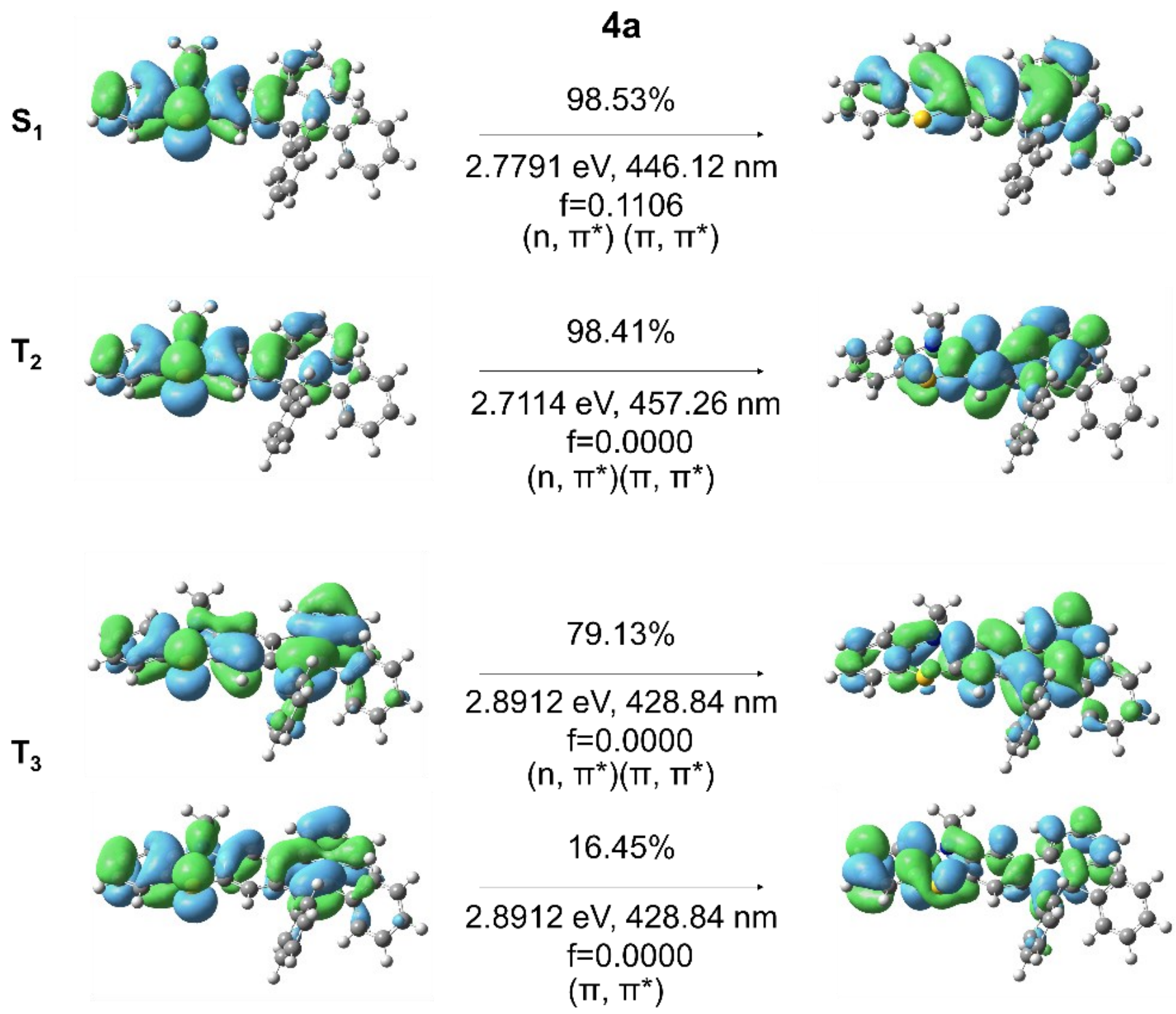

Fig. S48 NTOs based on optimized geometries of S_1 for **4a**.

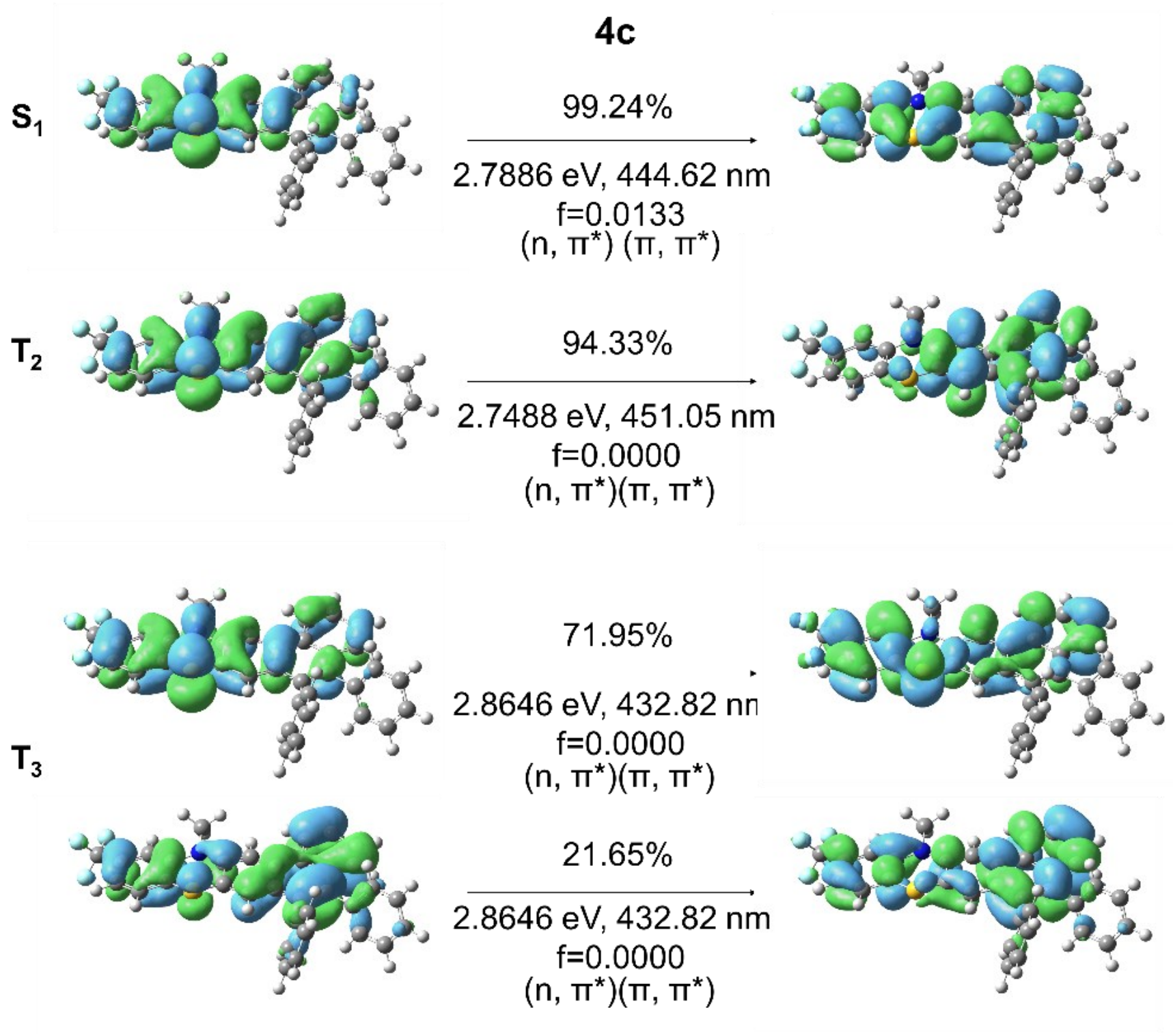

Fig. S49 NTOs based on optimized geometries of S_1 for **4c**.

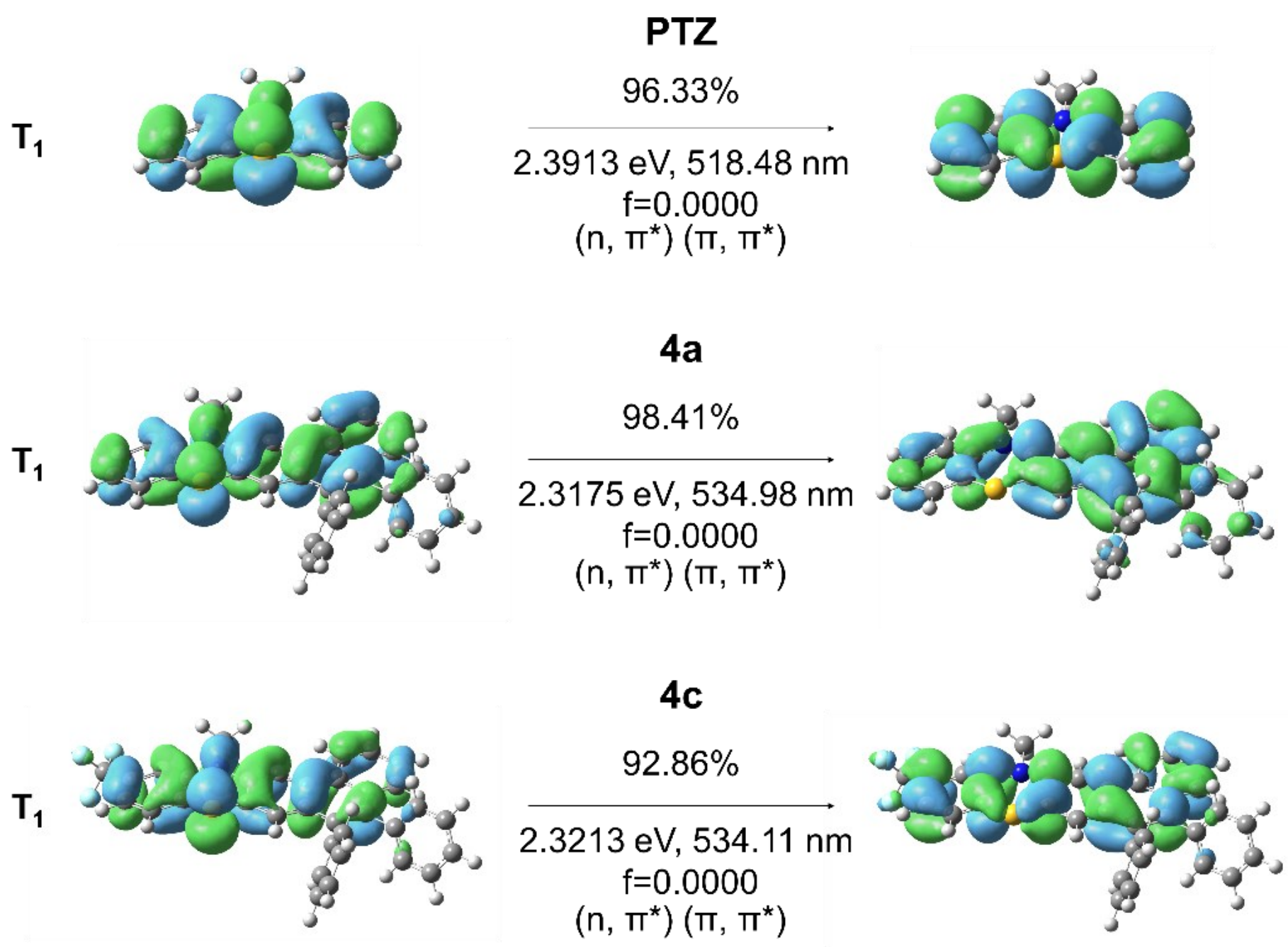

Fig. S50 NTOs based on optimized geometries of S_1 for **PTZ**, **4a**, and **4c**.

8. Cell imaging experiments

Cellular Experiments: The Hela cells were cultured in 10% FBS and 1% penicillin-streptomycin-containing DMEM medium at 37 °C with 5% CO_2.

Confocal imaging experiments: The confocal imaging experiments were performed on Leica TCS SP8. The nanoparticles of product **4g** (**4g** NPs) were used as a fluorescent probe. The nanoparticle of **4g** was prepared as follows: the compound **4g** (2.0 mg) was dissolved in THF (2 mL) followed by ultrasound for 10 min. Poloxamer 188 (60 mg) was added to the solution of **4g** in THF, and the mixture was treated by ultrasound for 10 min. The resulting mixture was stirred for three hours at RT. Then the solvent was removed by reduced pressure. The residue was dissolved in deionized water (2 mL) and stirred overnight at RT to drive the formation of NPs. To remove the residual THF, the nanoparticle suspensions were dialyzed by three times. The concentrations of nanoparticles were calibrated via the UV-vis spectra. Finally, the NPs solution was collected and stored at 4 °C.

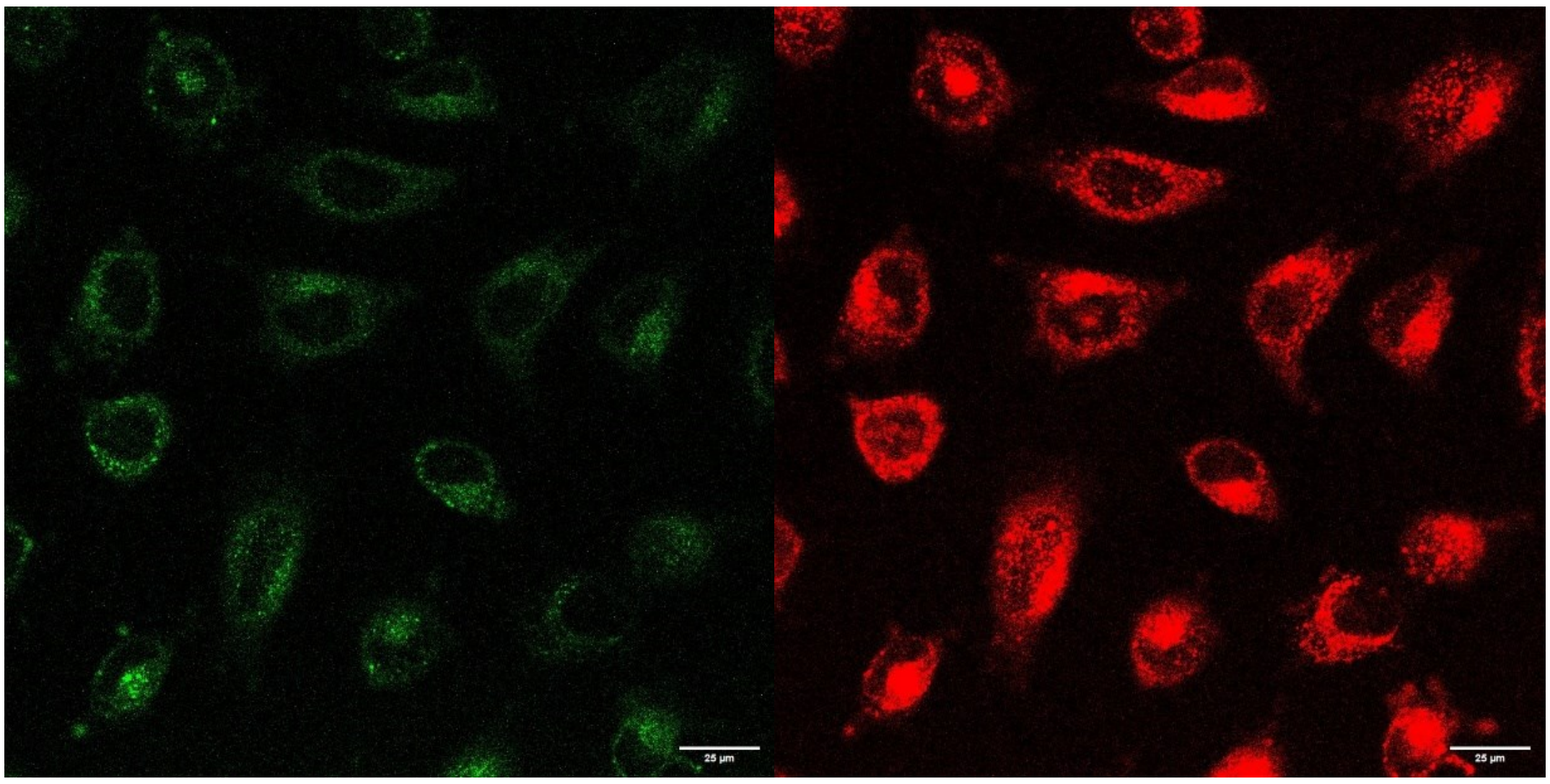

Fig. S51 Fluorescence microscopy images of HeLa cells incubated with **4g** NPs (10 μM) for 2 h at 37 °C. Left image: channel 415–470 nm, excitation 405 nm. Right image: Channel 500–600 nm. Excitation 488 nm. The scale bar is 25.0 μm.

Methyl thiazolyl tetrazolium (MTT) assay: To assess the safe usability of **4g** nanoparticles for biomedical applications, the cytotoxicity experiments in Hela cells were carried out using the standard methyl thiazolyl tetrazolium (MTT) assay.

Cell viability assay: The Hela cells were seeded into 96-well plates and incubated with a standard medium overnight. The cell suspensions were treated with different concentrations of **4g** NPs in a deionized water solution (5, 10, 20, 40, 80 μM) and incubated for another 24 h. Live/dead cell staining was carried out after 20 min white light irradiation and without light irradiation, respectively.

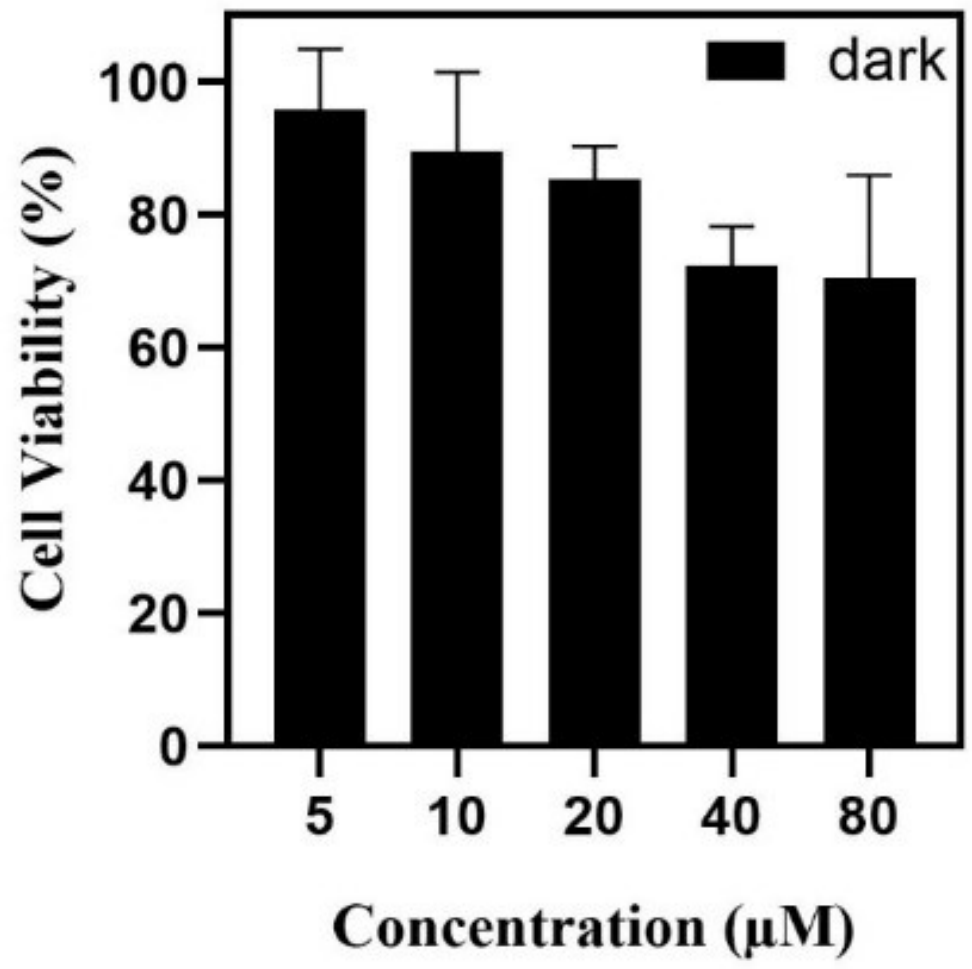

Fig. S52 In vitro cytotoxicity of the **4g** NPs. Cell viabilities of Hela cells incubated with **4g** NPs for 24 h.

9. The photocatalyzed reaction with 4d as photosensitizer

Chloride Oxidation: the general procedures were followed from the reported literature [S13].

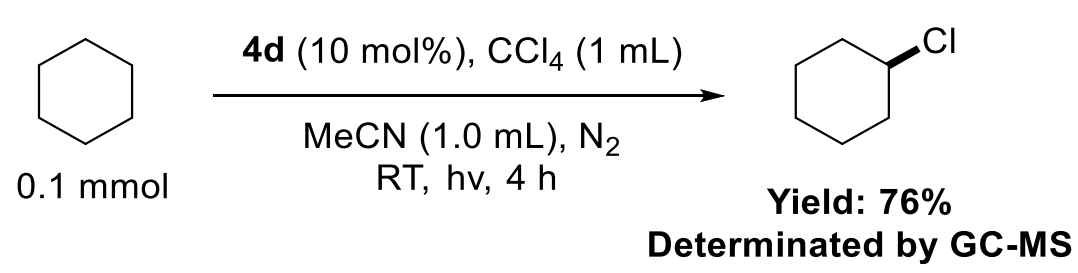

The GC-MS analysis report of chloride oxidation:

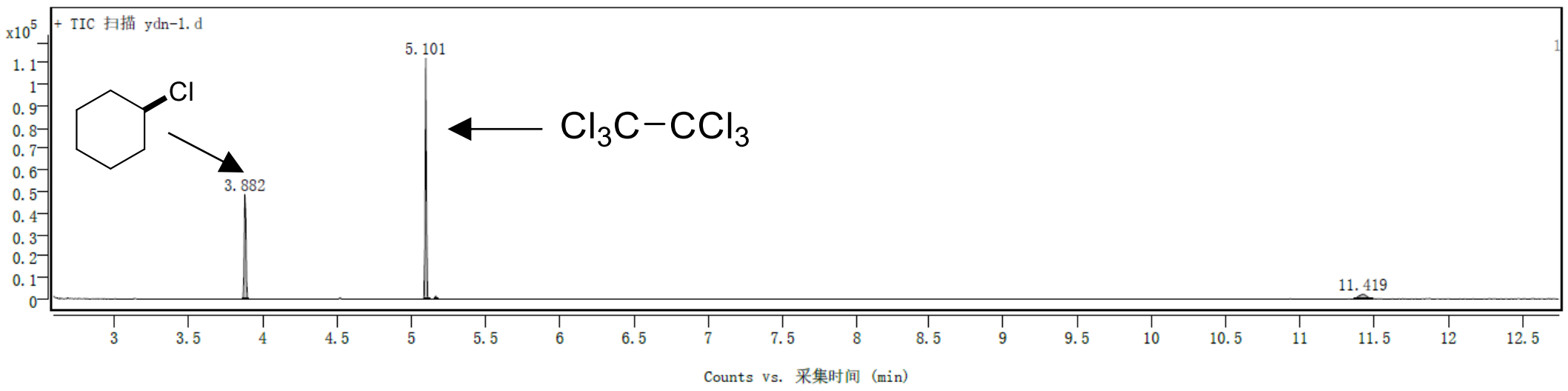

Chromatogram Peaks

峰	起始	RT	终止	峰高	面积	面积 %	SNR
1	3.869	3.882	3.907	48836	40729	62.58	
2	5.089	5.101	5.126	112394	65085	100.00	
3	5.158	5.170	5.183	1378	942	1.45	
4	11.357	11.419	11.482	2342	8567	13.16	

Sample Spectra

+ 扫描 (rt: 3.869-3.900 min)

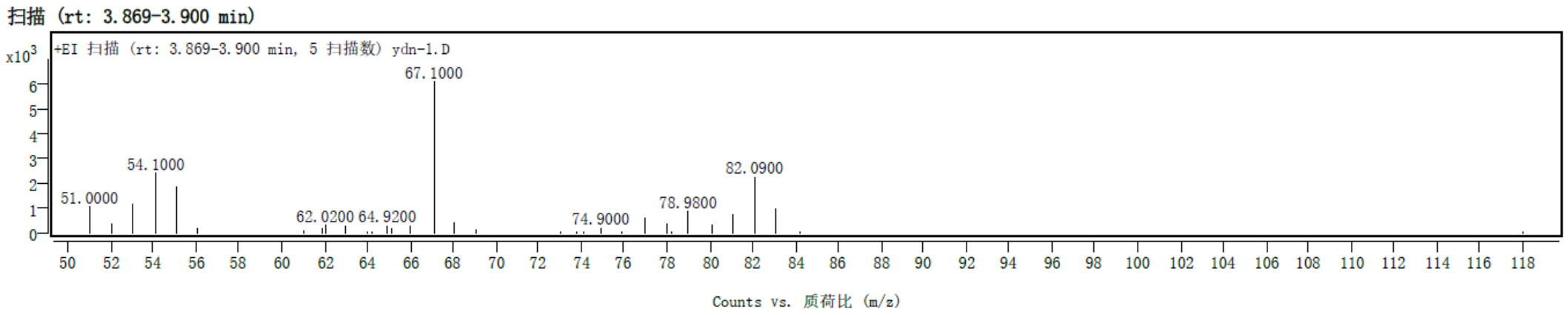

+ 扫描 (rt: 5.095-5.114 min)

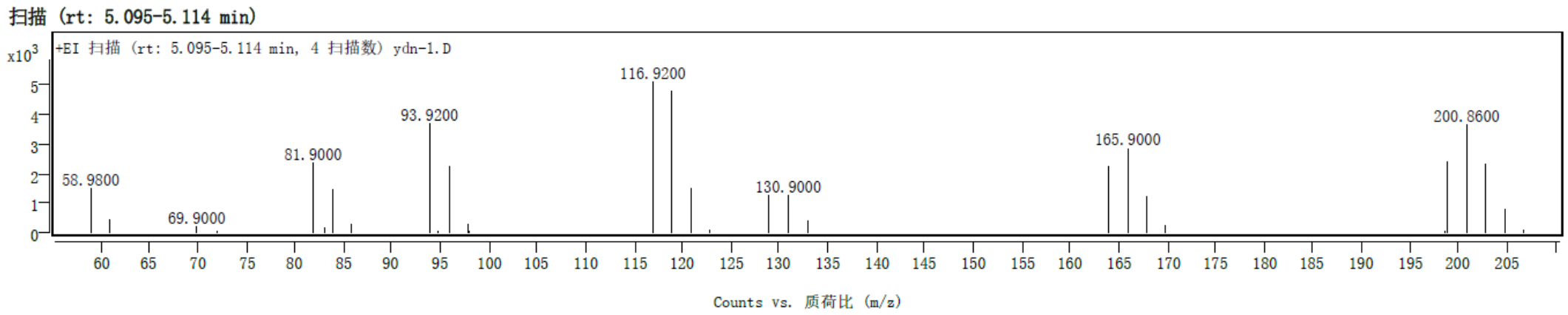

Cross-coupling reaction: the general procedures were followed the reported literature [S14].

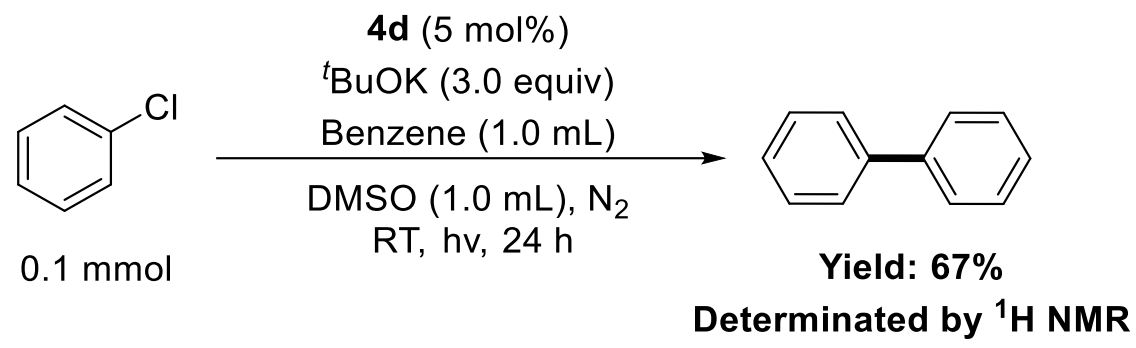

The GC-MS analysis report of cross-coupling reaction:

Sample Chromatograms

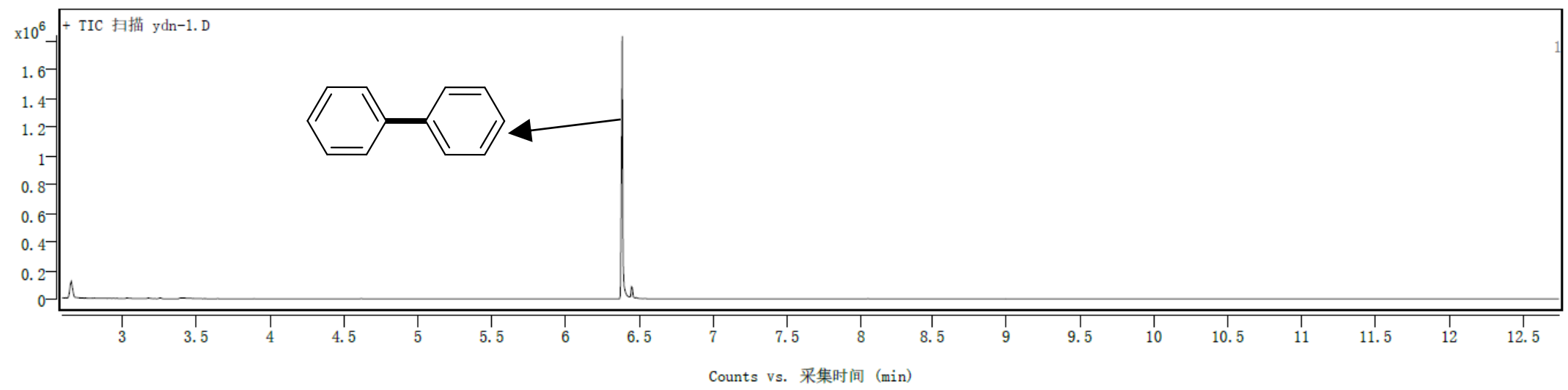

Sample Spectra

+ 扫描 (rt: 6.384-6.396 min)

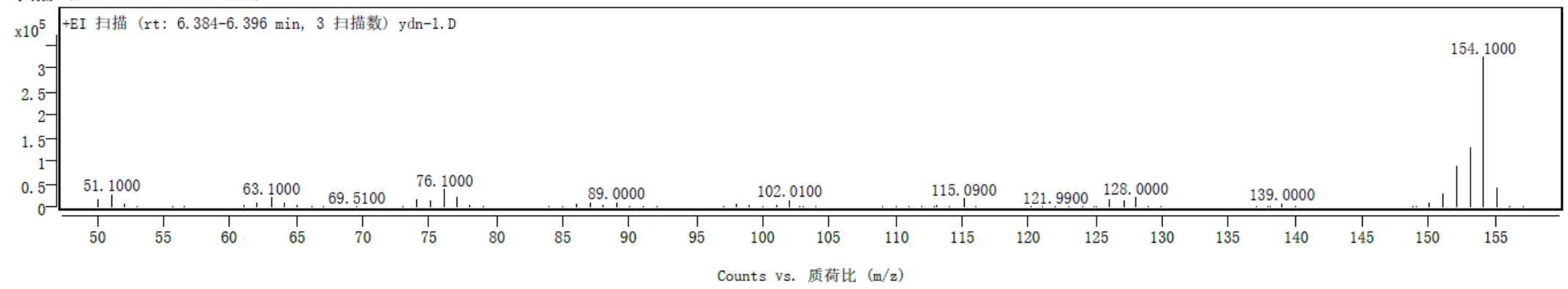

10. References

[S1] (a) P. Dao, F. Ye, Y. Liu, Z. Y. Du, K. Zhang, C. Z. Dong, B. Meunier and H. Chen, *ACS Chem. Neurosci.* **2018**, *8*, 798–806. (b) S.-Y. Su, H.-H. Lin and C.-C. Chang, *J. Mater. Chem.* **2010**, *20*, 8653–8658.

[S2] (a) N. J. Taylor, E. Emer, S. Preshlock, M. Schedler, M. Tredwell, S. Verhoog, J. Mercier, C. Genicot and V. Gouverneur, *J. Am. Chem. Soc.* **2017**, *139*, 8267–8276. (b) J. Zhang, T. Sun, K. Wang, R. Hu, C. Zhou, H. Ge and B. Li, *Chem. Sci.* **2014**, *15*, 12270-12276.

[S3] J. Bai, N. Xu, H. Wang and X. Luan, *Org. Lett.* **2022**, *24*, 5099–5104.

[S4] (a) J. Wang, Z. Dong, C. Yang and G. Dong, *Nat. Chem.* **2019**, *11*, 1106–1112. (b) V. Sukowski, M. Borselen, S. Mathew and M. Á. Fernández-Ibáñez, *Angew. Chem. Int. Ed.* **2022**, *61*, e202201750.

[S5] S. Kong, H. Wang, J. Liao, Y. Xiao, T. Yu and W. Huang, *Adv. Mater.* **2024**, *36*, 2412468.

[S6] M. J. Frisch, G. W. Trucks, H. B. Schlegel, G. E. Scuseria, M. A. Robb, J. R. Cheeseman, G. Scalmani, V. Barone, G. A. Petersson, H. Nakatsuji, X. Li, M. Caricato, A. V. Marenich, J. Bloino, B. G. Janesko, R. Gomperts, B. Mennucci, H. P. Hratchian, J. V. Ortiz, A. F. Izmaylov, J. L. Sonnenberg, D. Williams-Young, F.

Ding, F. Lipparini, F. Egidi, J. Goings, B. Peng, A. Petrone, T. Henderson, D. Ranasinghe, V. G. Zakrzewski, J. Gao, N. Rega, G. Zheng, W. Liang, M. Hada, M. Ehara, K. Toyota, R. Fukuda, J. Hasegawa, M. Ishida, T. Nakajima, Y. Honda, O. Kitao, H. Nakai, T. Vreven, K. Throssell, J. A. Montgomery, Jr., J. E. Peralta, F. Ogliaro, M. J. Bearpark, J. J. Heyd, E. N. Brothers, K. N. Kudin, V. N. Staroverov, T. A. Keith, R. Kobayashi, J. Normand, K. Raghavachari, A. P. Rendell, J. C. Burant, S. S. Iyengar, J. Tomasi, M. Cossi, J. M. Millam, M. Klene, C. Adamo, R. Cammi, J. W. Ochterski, R. L. Martin, K. Morokuma, O. Farkas, J. B. Foresman, D. J. Fox, Gaussian 16, Revision A.03, Gaussian, Inc., Wallingford CT, **2016**.

[S7] R. L. Martin, *J. Comput. Chem.*, **2003**, *118*, 4775-4777.

[S8] W. Liu, G. Hong, D. Dai, L. Li and M. Dolg, *Theor. Chem. Acc.* **1997**, *96*, 75-83.

[S9] W. Liu, F. Wang and L. Li, *J. Theor. Comput. Chem.*, **2003**, *02*, 257-272.

[S10] Y. Zhang, B. Suo, Z. Wang, N. Zhang, Z. Li, Y. Lei, W. Zou, J. Gao, D. Peng, Z. Pu, Y. Xiao, Q. Sun, F. Wang, Y. Ma, X. Wang, Y. Guo and W. Liu, *J. Chem. Phys.* **2020**, *152*, 064113.

[S11] W. Liu, F. Wang and L. Li, In *Recent Advances in Relativistic Molecular Theory*, pp 257-282.

[S12] Z. Wang, Z. Li, Y. Zhang and W. Liu, *J. Chem. Phys.* **2020**, *153*, 164109.

[S13] P. Li, A. M. Deetz, J. Hu, G. J. Meyer and K. Hu, *J. Am. Chem. Soc.* **2022**, *144*, 17604−17610.

[S14] S. Halder, S. Mandal, A. Kundu, B. Mandal and D. Adhikari, *J. Am. Chem. Soc.* **2023**, *145*, 22403−22412.

11. X-Ray Crystallographic Spectrum of 4b

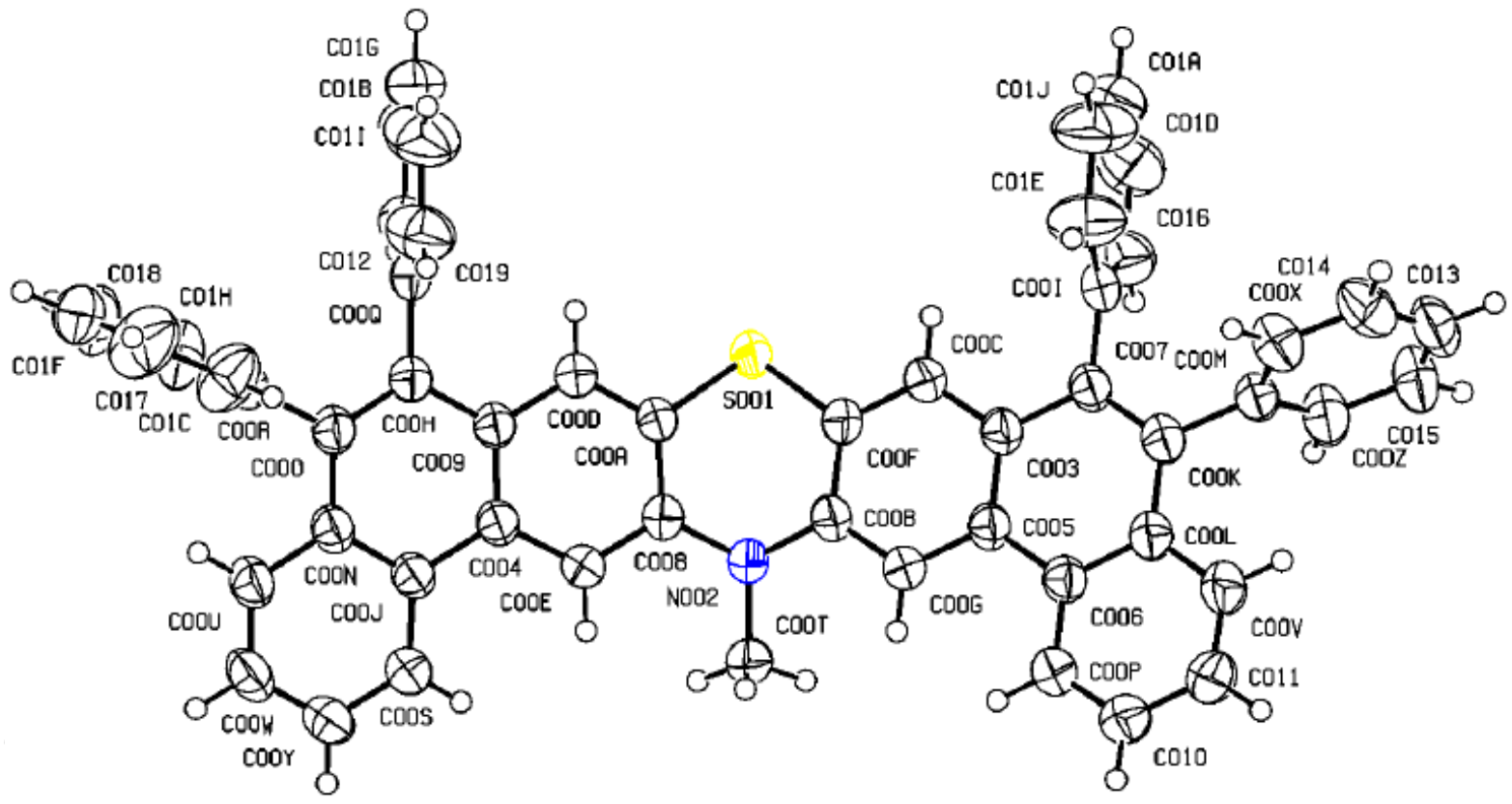

Fig. S53 The crystal structure of **4b** (CCDC 2356081).

Table S7. Crystal data and structure refinement for **4b**.

Identification code	20240423-zcl-2-65_sq	
Empirical formula	$C_{53}H_{35}NS$	
Formula weight	717.88	
Temperature	293(2) K	
Wavelength	1.54184 Å	
Crystal system	Monoclinic	
Space group	C2/c	
Unit cell dimensions	a = 21.6426(3) Å	α= 90°.
	b = 14.5352(2) Å	β= 101.1980(10)°.
	c = 25.8468(3) Å	γ= 90°.
Volume	7976.08(18) Å^3	
Z	8	
Density (calculated)	1.196 g/cm^3	
Absorption coefficient	0.996 mm^{-1}	
F(000)	3008.0	
Crystal size	0.3 x 0.27 x 0.26 mm^3	
Theta range for data collection	6.972 to 140.24°.	
Index ranges	-19<=h<=26, -17<=k<=16, -31<=l<=26	
Reflections collected	20392	
Independent reflections	7510 [R_{int} = 0.0169, R_{sigma} = 0.0181]	
Completeness to theta = 70.120°	0.991	
Refinement method	Full-matrix least-squares on F^2	
Data / restraints / parameters	7510 / 0 / 497	
Goodness-of-fit on F^2	1.035	
Final R indices [I>2sigma(I)]	R_1 = 0.0395, wR_2 = 0.1071	
Final R indices (all data)	R_1 = 0.0499, wR_2 = 0.1168	
Extinction coefficient	n/a	
Largest diff. peak and hole	0.140 and -0.20 e.Å^{-3}	

12. NMR spectrum of compounds

NMR spectrum of products 4a – 4o and 5a-5b:

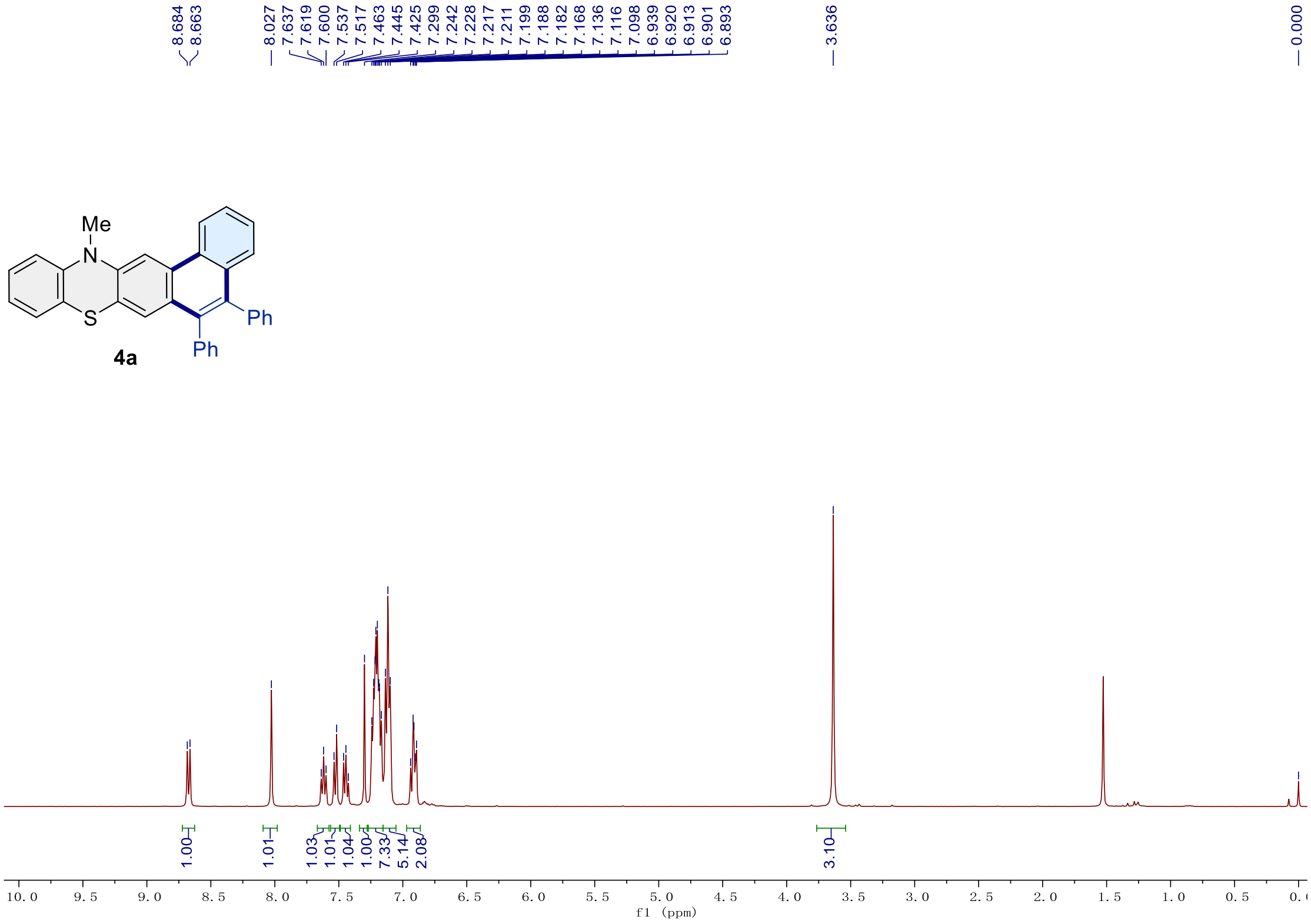

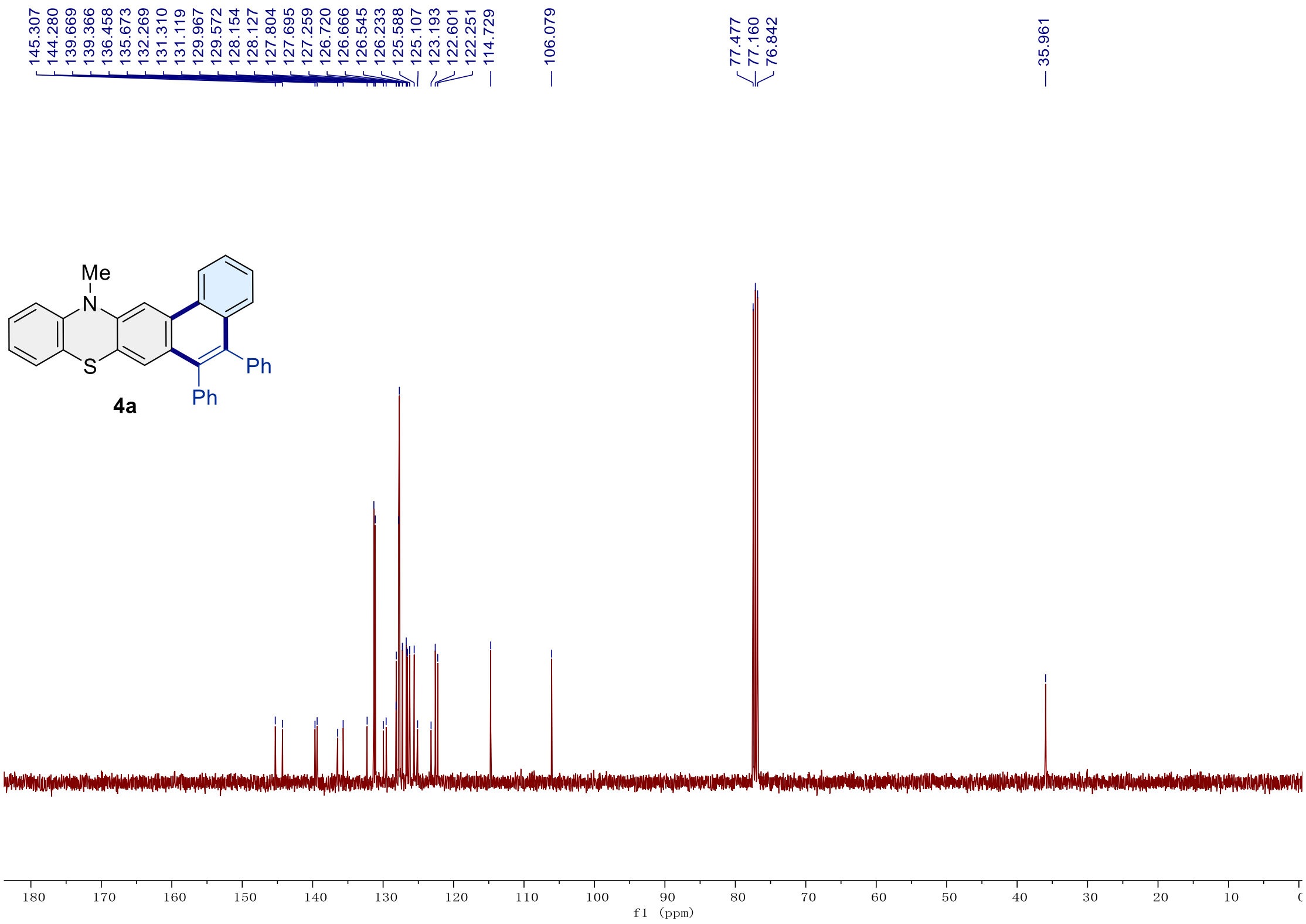

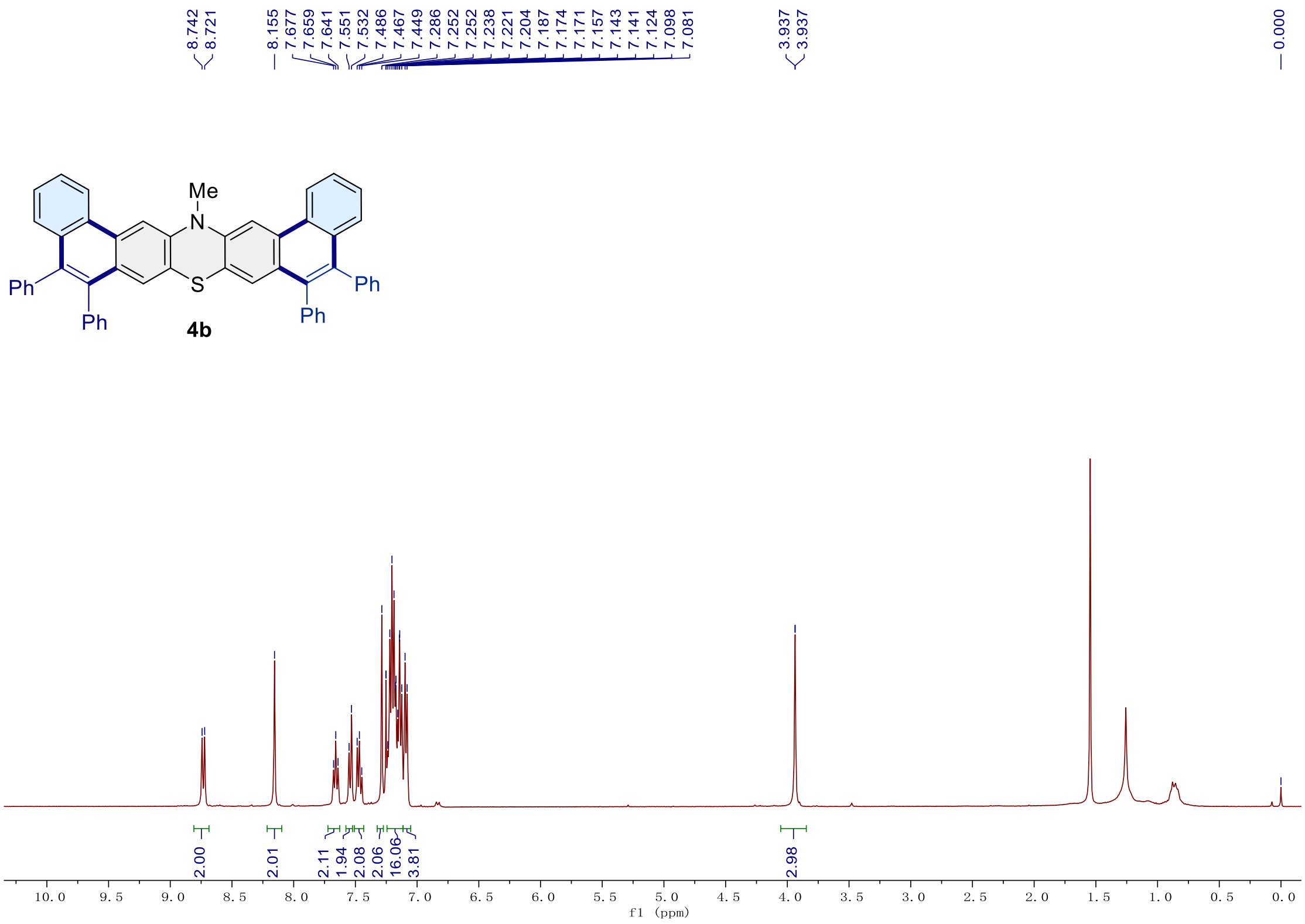
8.742
8.721
8.155
7.677
7.659
7.641
7.551
7.532
7.486
7.467
7.449
7.286
7.252
7.252
7.238
7.221
7.204
7.187
7.174
7.171
7.157
7.143
7.141
7.124
7.098
7.081
3.937
3.937
0.000
Me
N
S
Ph
Ph
Ph
Ph
4b
2.00
2.01
2.11
1.94
2.08
2.06
16.06
3.81
2.98
f1 (ppm)

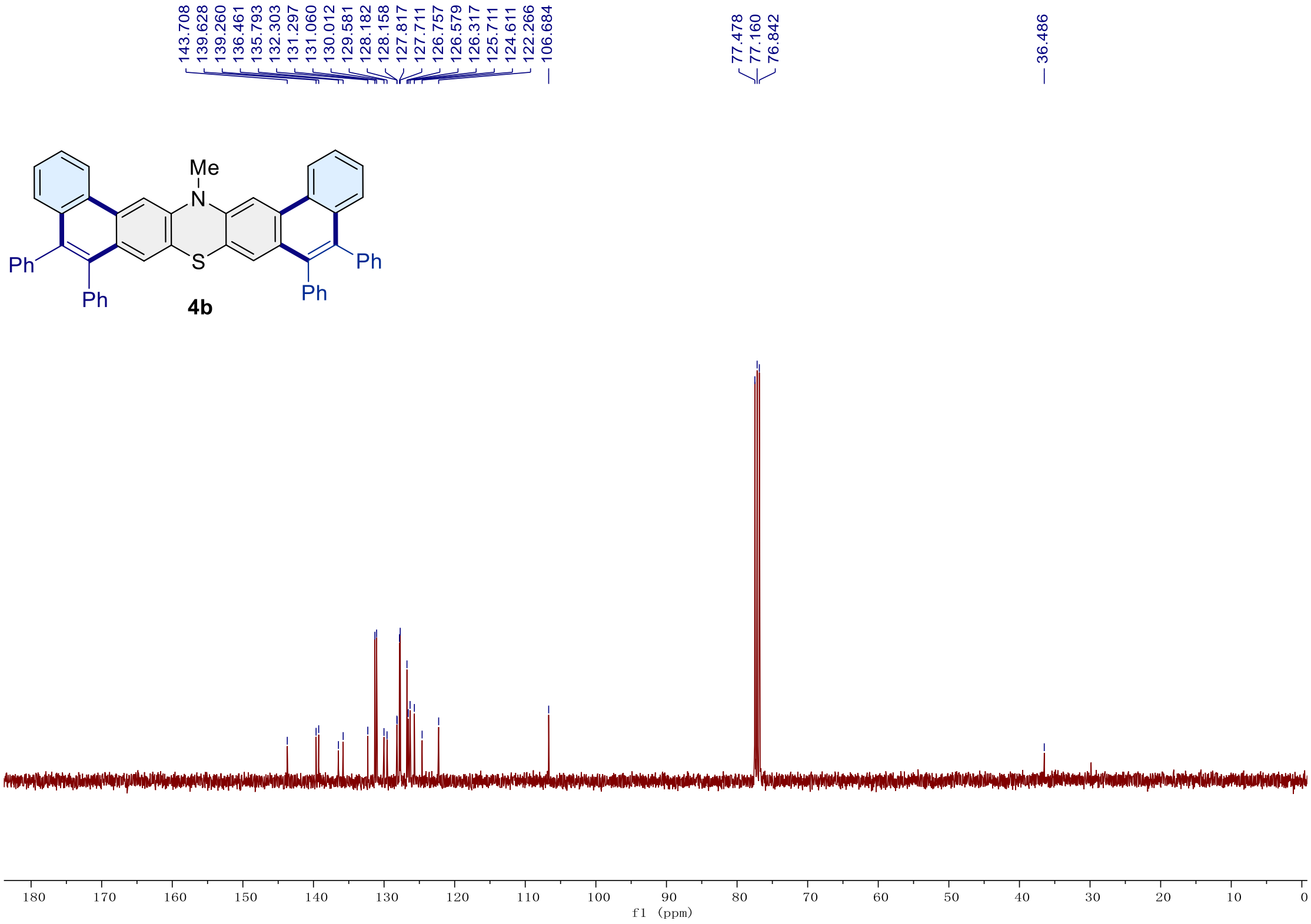
143.708
139.628
139.260
136.461
135.793
132.303
131.297
131.060
130.012
129.581
128.182
128.158
127.817
127.711
126.757
126.579
126.317
125.711
124.611
122.266
106.684
77.478
77.160
76.842
36.486
Me
N
S
Ph
Ph
Ph
Ph
4b
f1 (ppm)

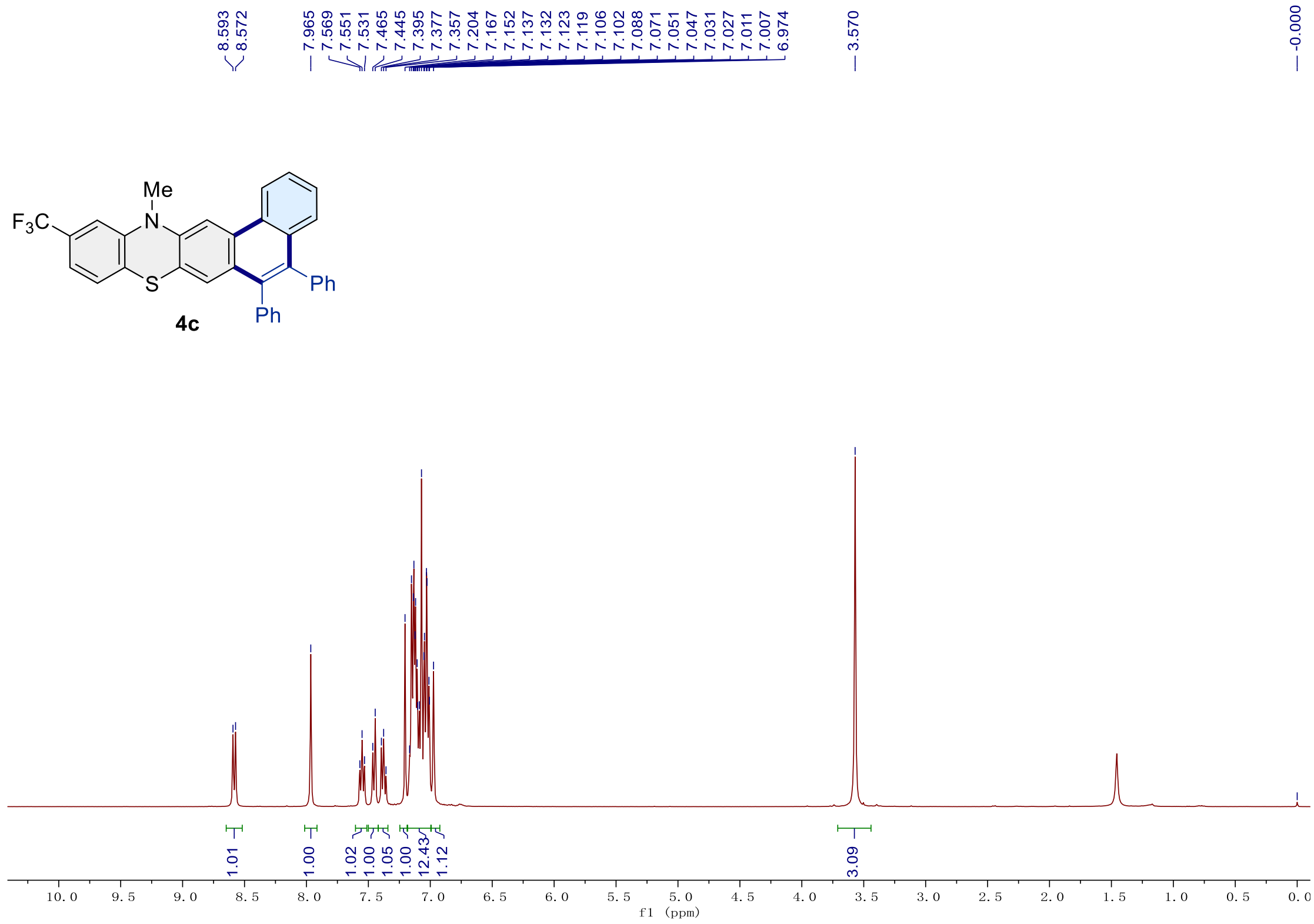

8.593
8.572
7.965
7.569
7.551
7.531
7.465
7.445
7.395
7.377
7.357
7.204
7.167
7.152
7.137
7.132
7.123
7.119
7.106
7.102
7.088
7.071
7.051
7.047
7.031
7.027
7.011
7.007
6.974
3.570
-0.000
Me
N
F3C
S
Ph
Ph
4c
1.01
1.00
1.02
1.00
1.05
1.00
12.43
1.12
3.09
10.0 9.5 9.0 8.5 8.0 7.5 7.0 6.5 6.0 5.5 5.0 4.5 4.0 3.5 3.0 2.5 2.0 1.5 1.0 0.5 0.0
f1 (ppm)

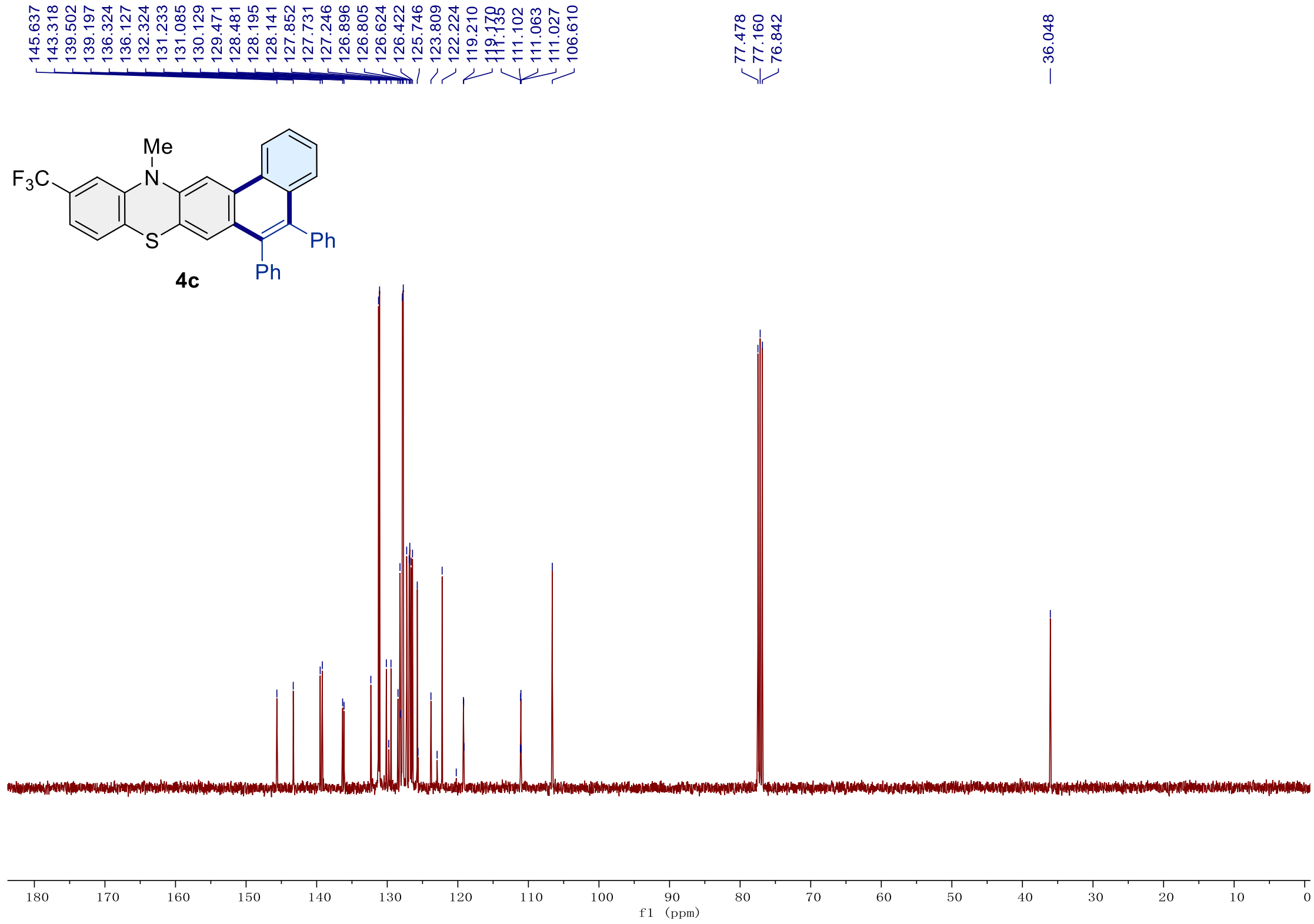

145.637
143.318
139.502
139.197
136.324
136.127
132.324
131.233
131.085
130.129
129.471
128.481
128.195
128.141
127.852
127.731
127.246
126.896
126.805
126.624
126.422
125.746
123.809
122.224
119.210
119.170
111.135
111.102
111.063
111.027
106.610
77.478
77.160
76.842
36.048
Me
N
F3C
S
Ph
Ph
4c
180 170 160 150 140 130 120 110 100 90 80 70 60 50 40 30 20 10 0
f1 (ppm)

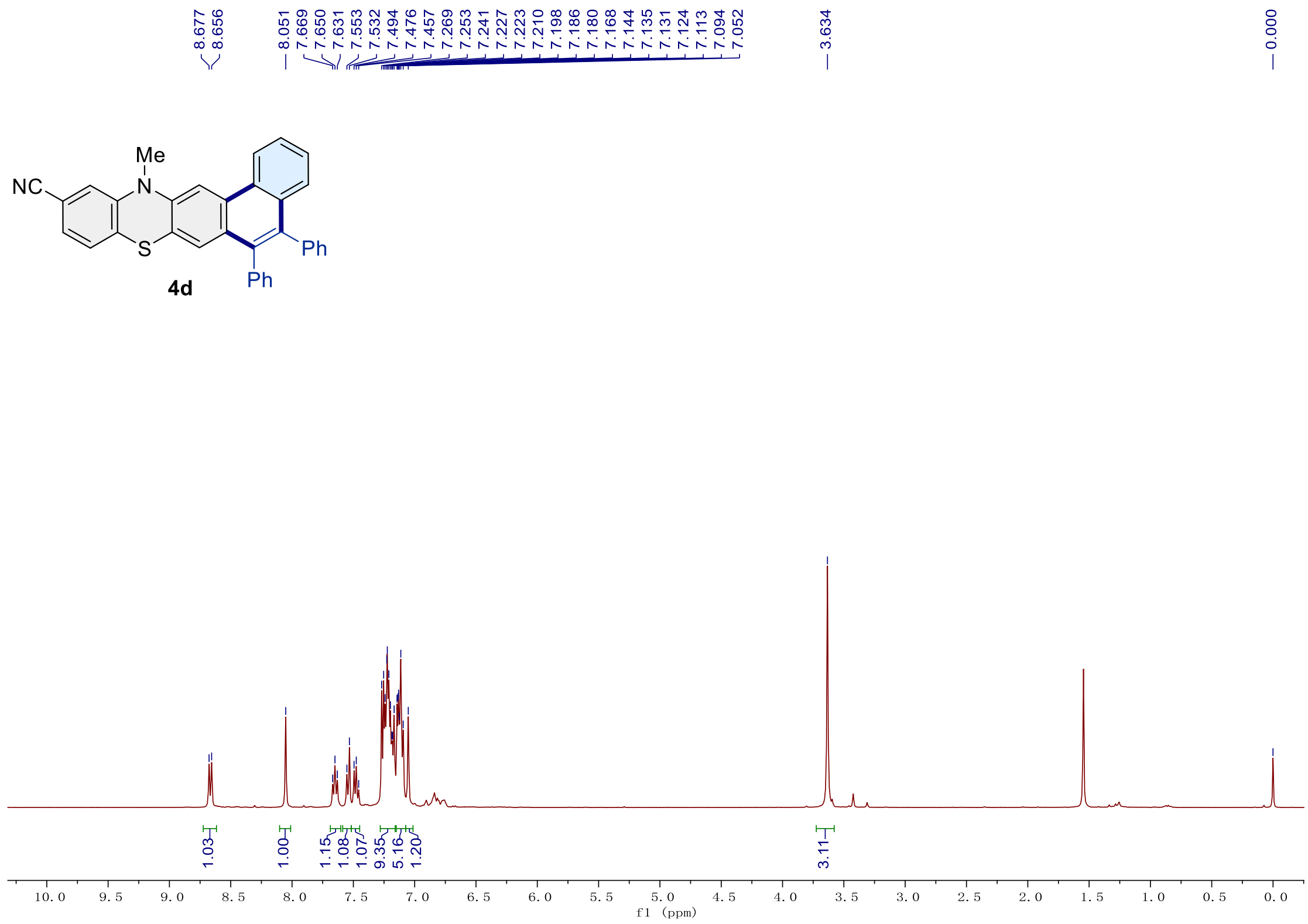
8.677
8.656
8.051
7.669
7.650
7.631
7.553
7.532
7.494
7.476
7.457
7.269
7.253
7.241
7.227
7.223
7.210
7.198
7.186
7.180
7.168
7.144
7.135
7.131
7.124
7.113
7.094
7.052
3.634
0.000
Me
NC
N
S
Ph
Ph
4d
1.03
1.00
1.15
1.08
1.07
9.35
5.16
1.20
3.11
f1 (ppm)

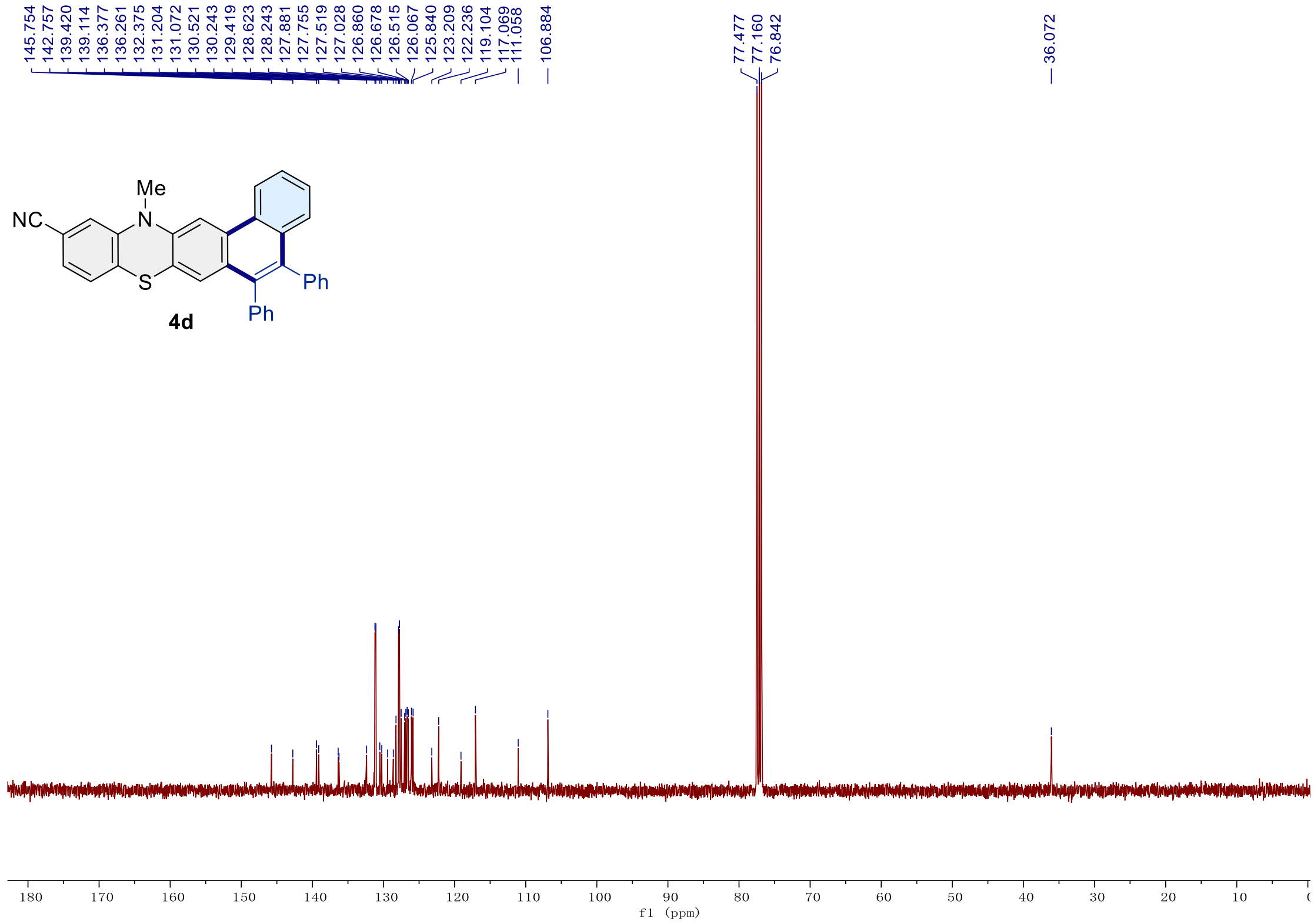
145.754
142.757
139.420
139.114
136.377
136.261
132.375
131.204
131.072
130.521
130.243
129.419
128.623
128.243
127.881
127.755
127.519
127.028
126.860
126.678
126.515
126.067
125.840
123.209
122.236
119.104
117.069
111.058
106.884
77.477
77.160
76.842
36.072
Me
NC
N
S
Ph
Ph
4d
f1 (ppm)

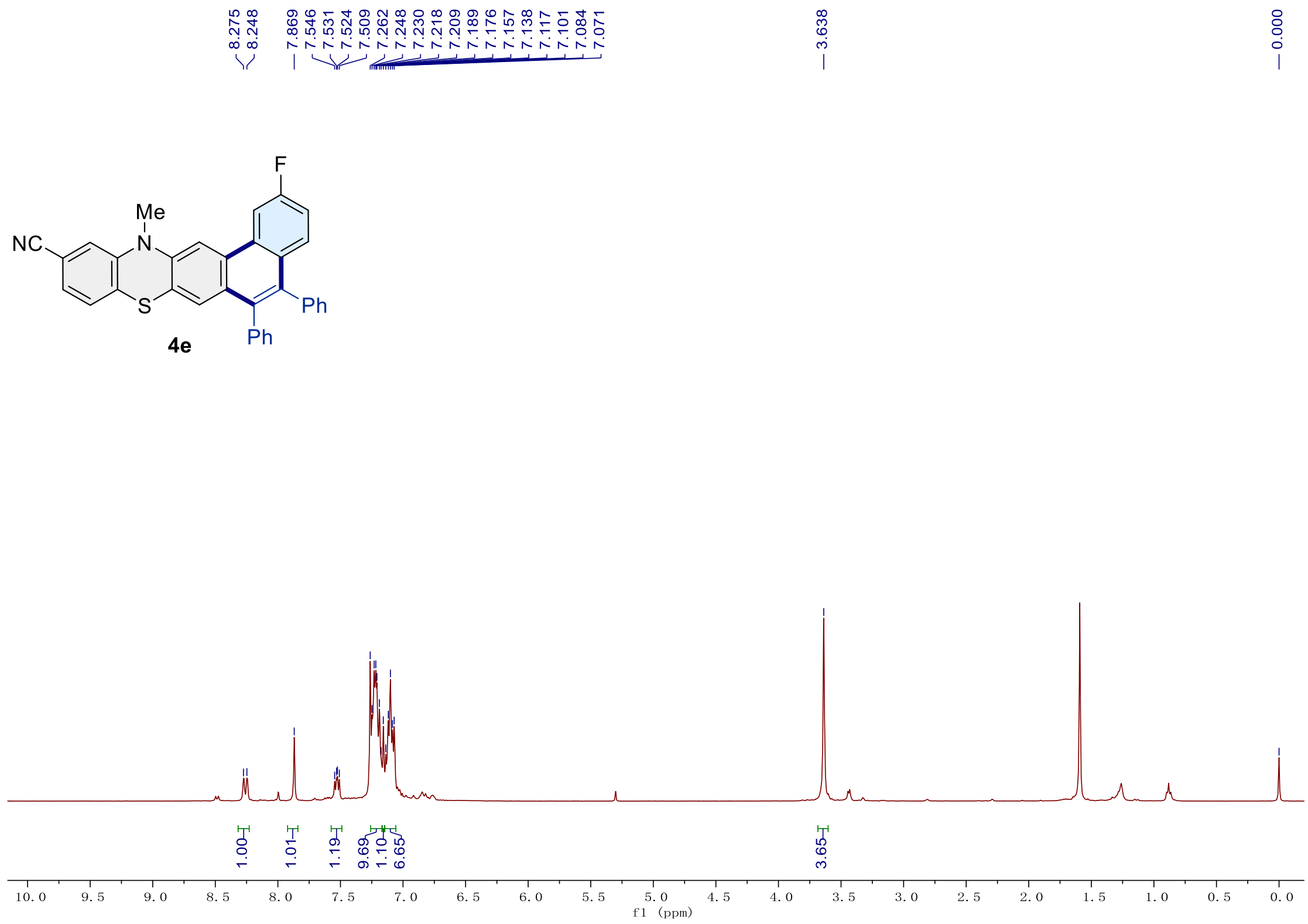
8.275
8.248
7.869
7.546
7.531
7.524
7.509
7.262
7.248
7.230
7.218
7.209
7.189
7.176
7.157
7.138
7.117
7.101
7.084
7.071
3.638
0.000
F
Me
N
NC
S
Ph
Ph
4e
1.00
1.01
1.19
9.69
1.10
6.65
3.65
f1 (ppm)

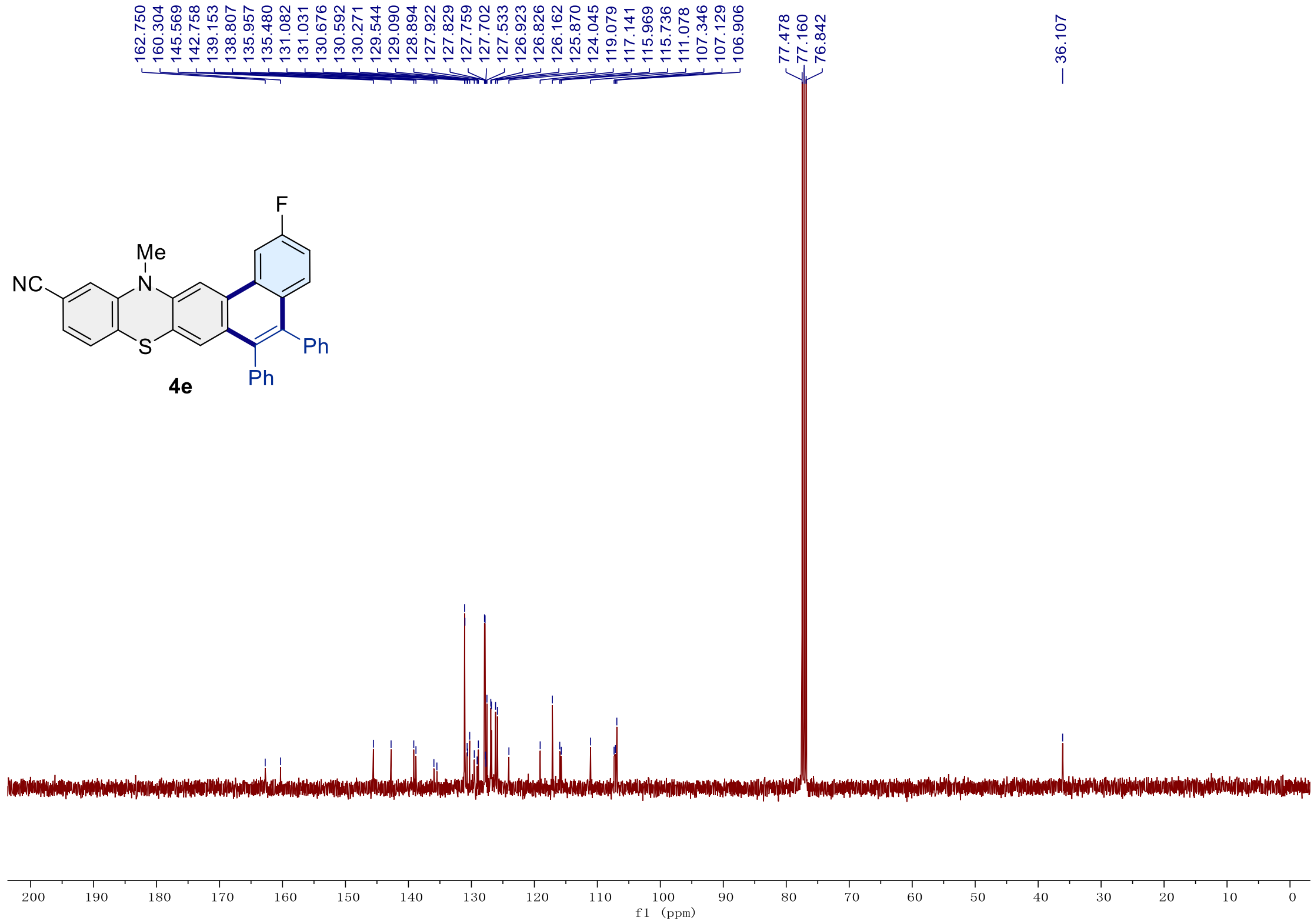
162.750
160.304
145.569
142.758
139.153
138.807
135.957
135.480
131.082
131.031
130.676
130.592
130.271
129.544
129.090
128.894
127.922
127.829
127.759
127.702
127.533
126.923
126.826
126.162
125.870
124.045
119.079
117.141
115.969
115.736
111.078
107.346
107.129
106.906
77.478
77.160
76.842
36.107
F
Me
N
NC
S
Ph
Ph
4e
f1 (ppm)

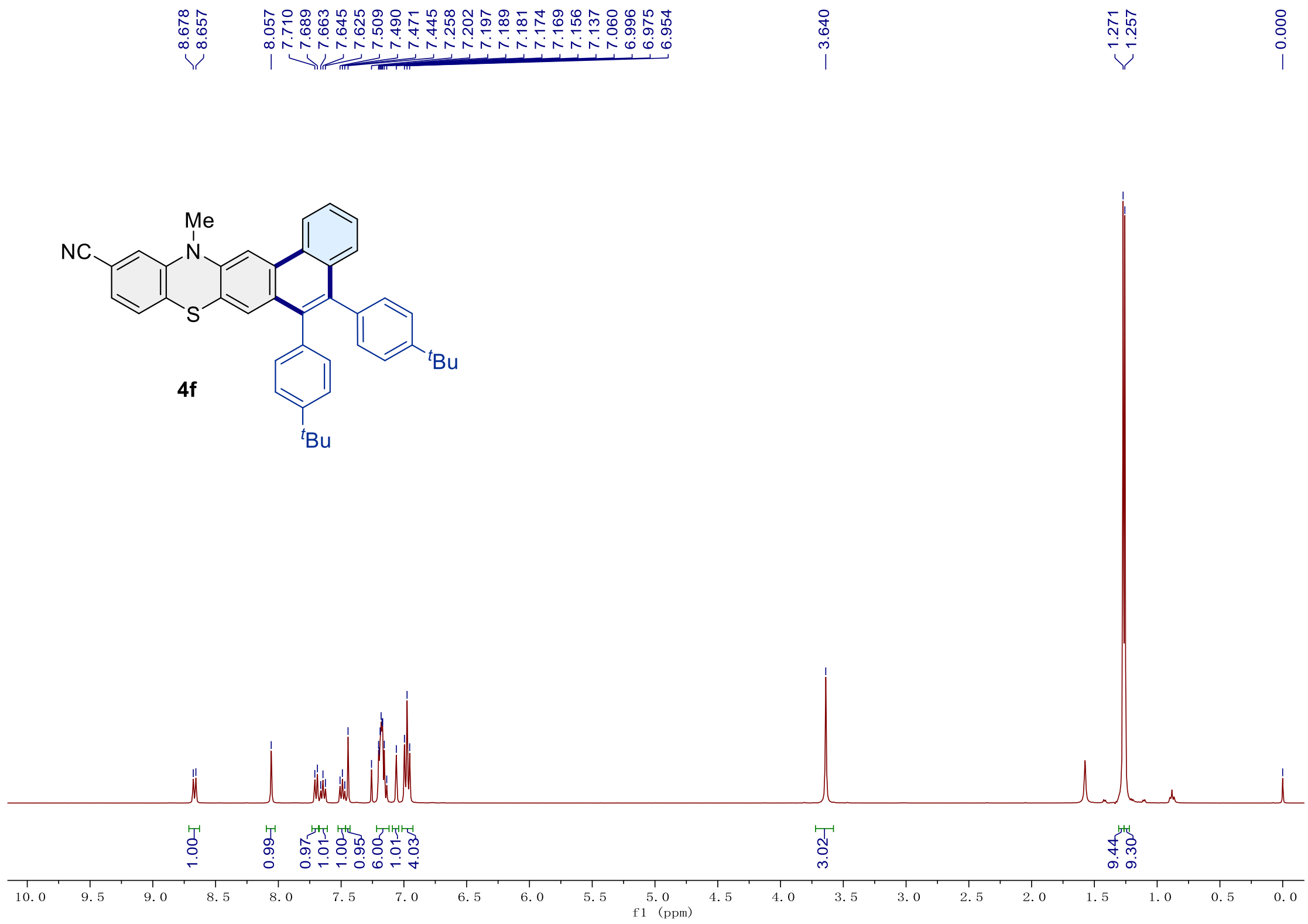
8.678
8.657
8.057
7.710
7.689
7.663
7.645
7.625
7.509
7.490
7.471
7.445
7.258
7.202
7.197
7.189
7.181
7.174
7.169
7.156
7.137
7.060
6.996
6.975
6.954
3.640
1.271
1.257
0.000
NC
Me
N
S
tBu
tBu
4f
1.00
0.99
0.97
1.01
1.00
0.95
6.00
1.01
1.01
4.03
3.02
9.44
9.30
f1 (ppm)

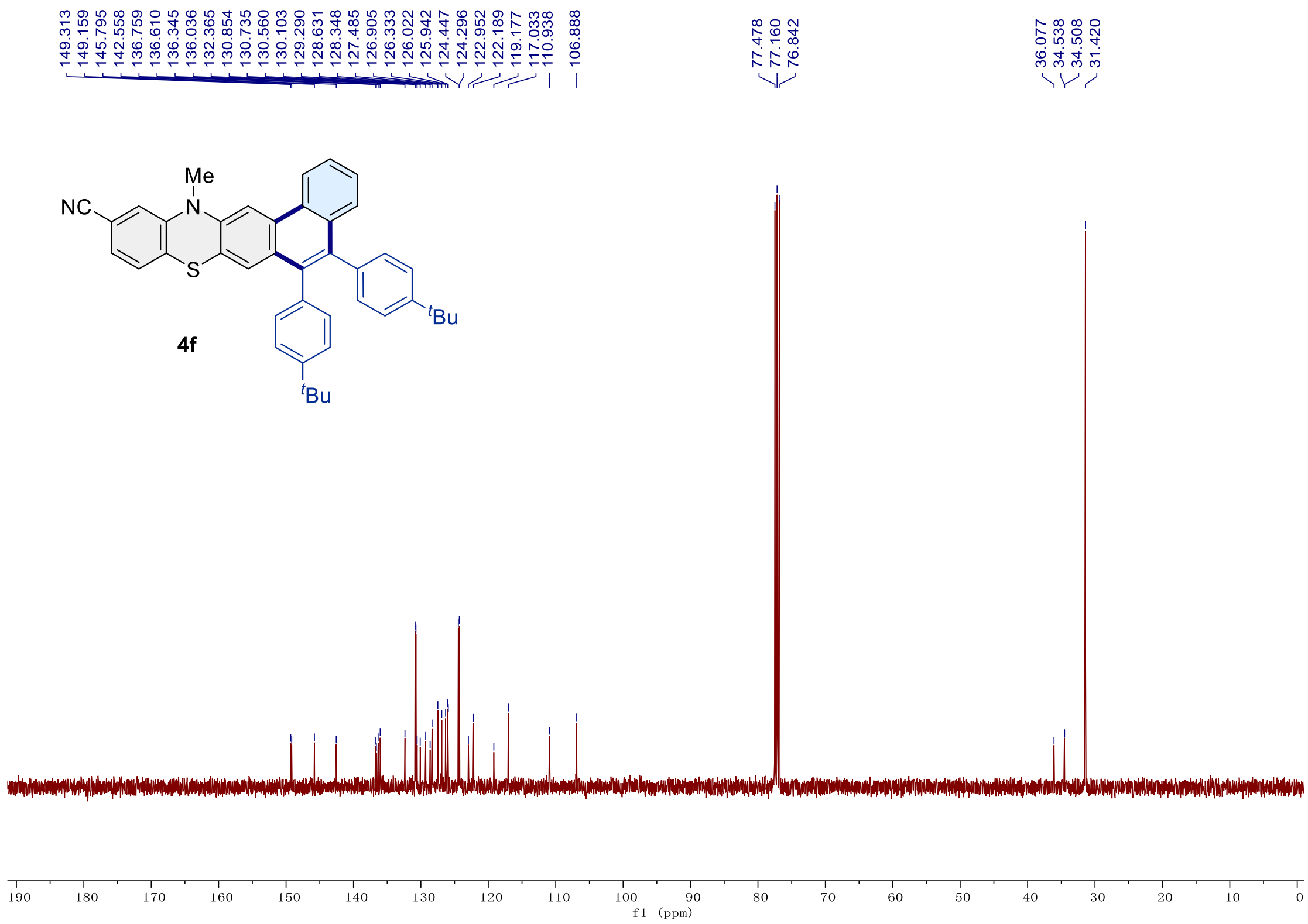
149.313
149.159
145.795
142.558
136.759
136.610
136.345
136.036
132.365
130.854
130.735
130.560
130.103
129.290
128.631
128.348
127.485
126.905
126.333
126.022
125.942
124.447
124.296
122.952
122.189
119.177
117.033
110.938
106.888
77.478
77.160
76.842
36.077
34.538
34.508
31.420
NC
Me
N
S
tBu
tBu
4f
f1 (ppm)

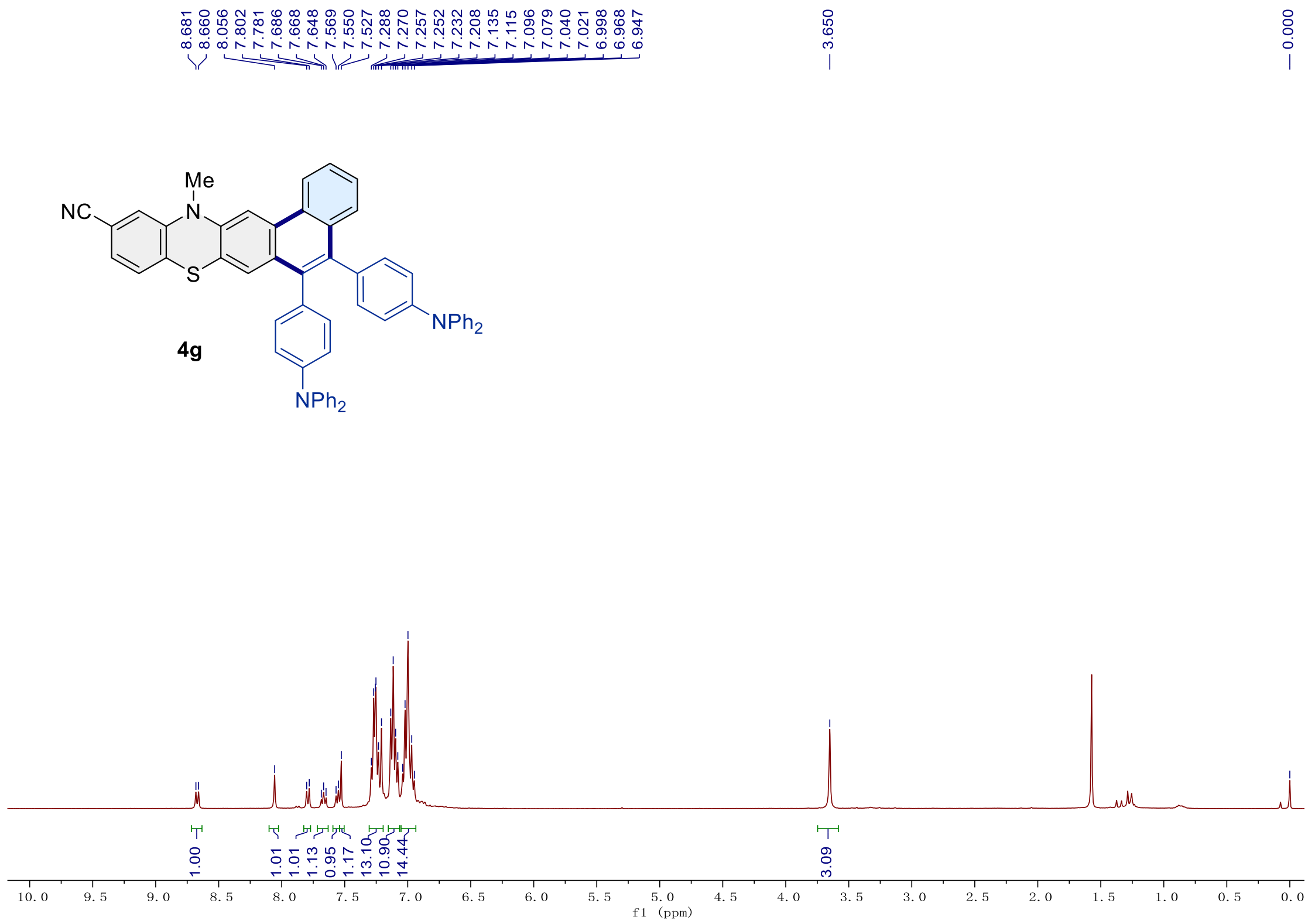

8.681
8.660
8.056
7.802
7.781
7.686
7.668
7.648
7.569
7.550
7.527
7.288
7.270
7.257
7.252
7.232
7.208
7.135
7.115
7.096
7.079
7.040
7.021
6.998
6.968
6.947
3.650
0.000
NC
Me
N
S
NPh2
NPh2
1.00
1.01
1.01
1.13
0.95
1.17
13.10
10.90
14.44
3.09
f1 (ppm)

4g

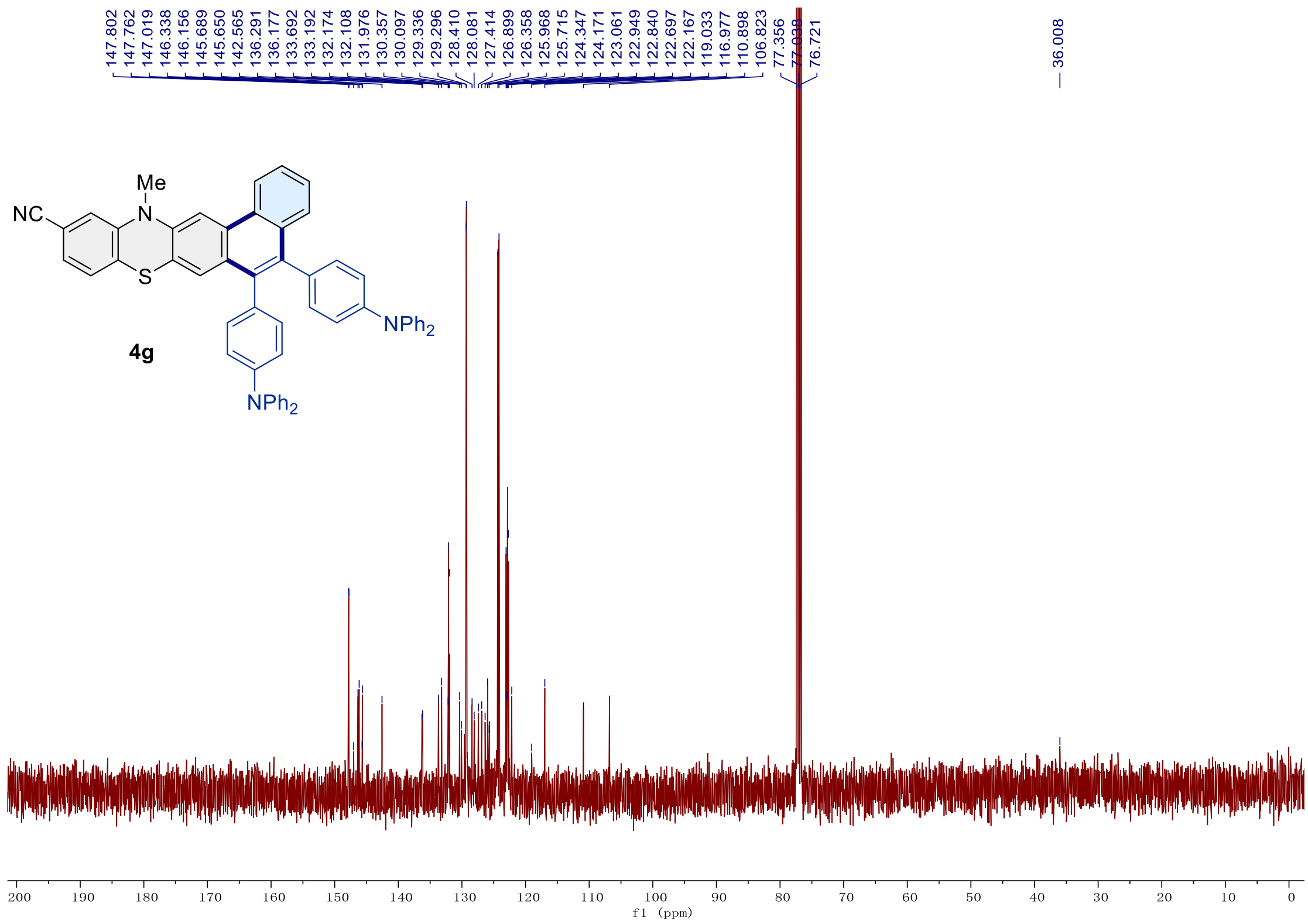

147.802
147.762
147.019
146.338
146.156
145.689
145.650
142.565
136.291
136.177
133.692
133.192
132.174
132.108
131.976
130.357
130.097
129.336
129.296
128.410
128.081
127.414
126.899
126.358
125.968
125.715
124.347
124.171
123.061
122.949
122.840
122.697
122.167
119.033
116.977
110.898
106.823
77.356
77.039
76.721
36.008
NC
Me
N
S
NPh2
NPh2
f1 (ppm)

4g

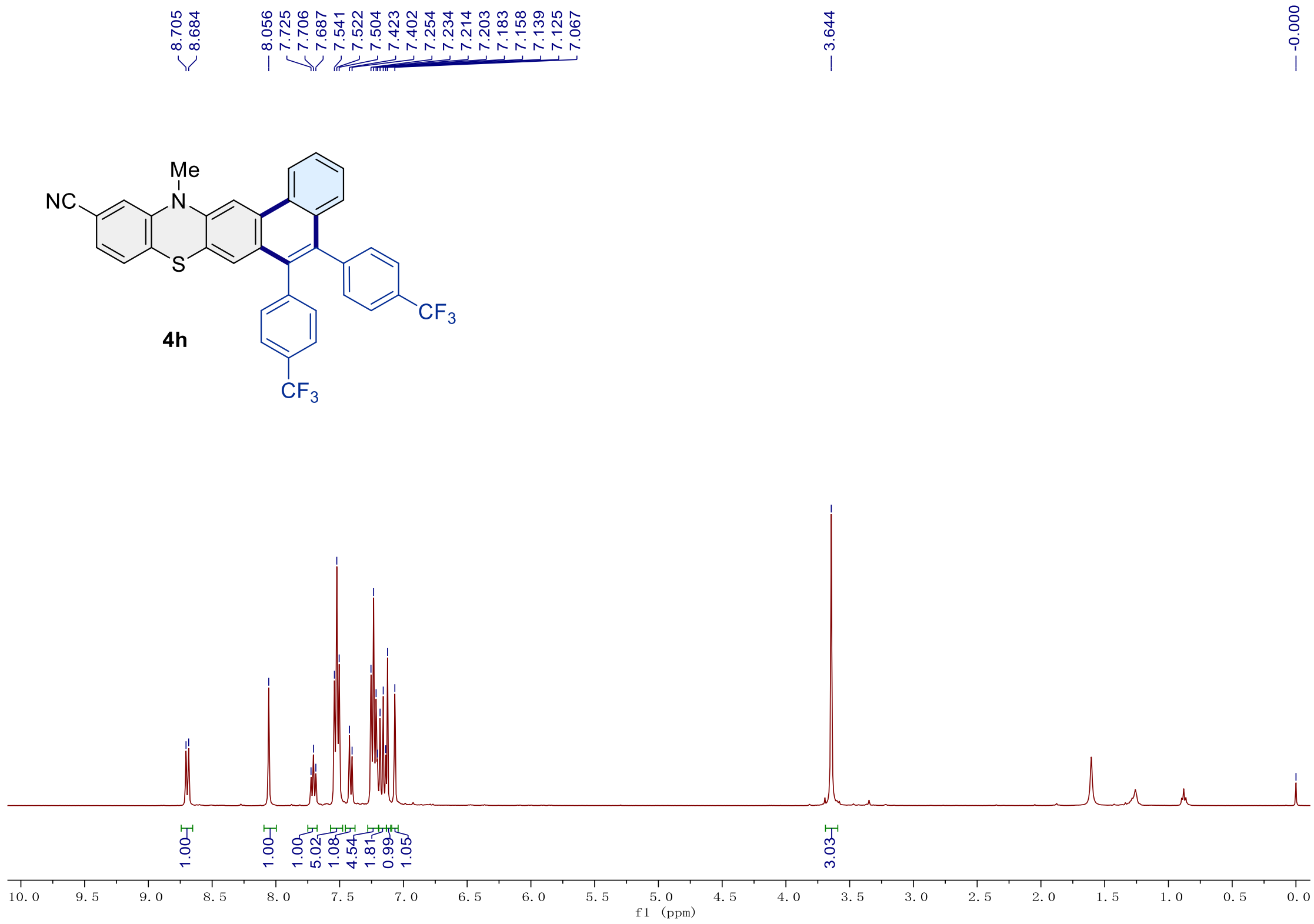
8.705
8.684
8.056
7.725
7.706
7.687
7.541
7.522
7.504
7.423
7.402
7.254
7.234
7.214
7.203
7.183
7.158
7.139
7.125
7.067
3.644
-0.000
NC
Me
N
S
CF_3
CF_3
4h
1.00
1.00
1.00
5.02
1.08
4.54
1.81
0.99
1.05
3.03
f1 (ppm)

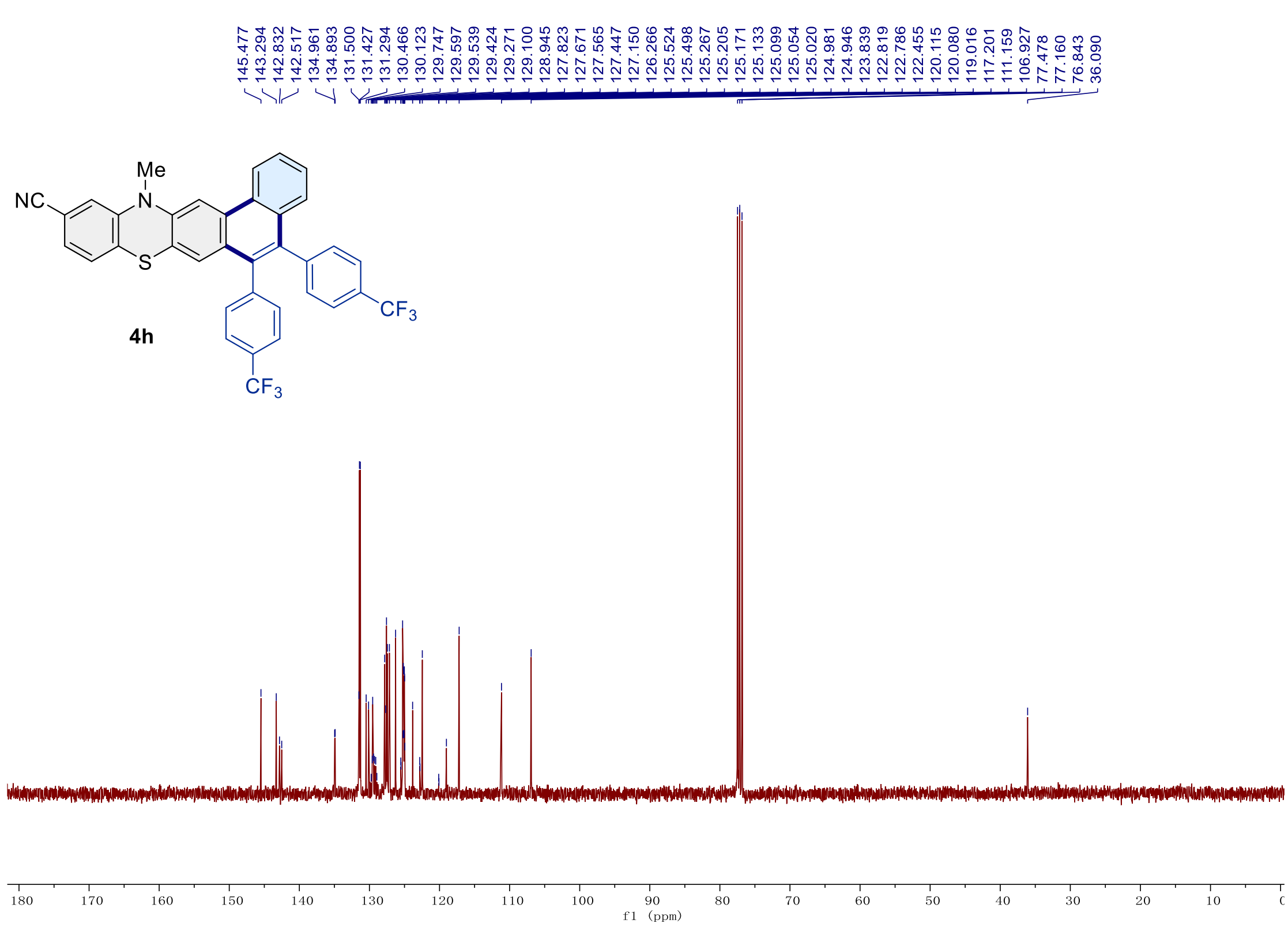
145.477
143.294
142.832
142.517
134.961
134.893
131.500
131.427
131.294
130.466
130.123
129.747
129.597
129.539
129.424
129.271
129.100
128.945
127.823
127.671
127.565
127.447
127.150
126.266
125.524
125.498
125.267
125.205
125.171
125.133
125.099
125.054
125.020
124.981
124.946
123.839
122.819
122.786
122.455
120.115
120.080
119.016
117.201
111.159
106.927
77.478
77.160
76.843
36.090
NC
Me
N
S
CF_3
CF_3
4h
f1 (ppm)

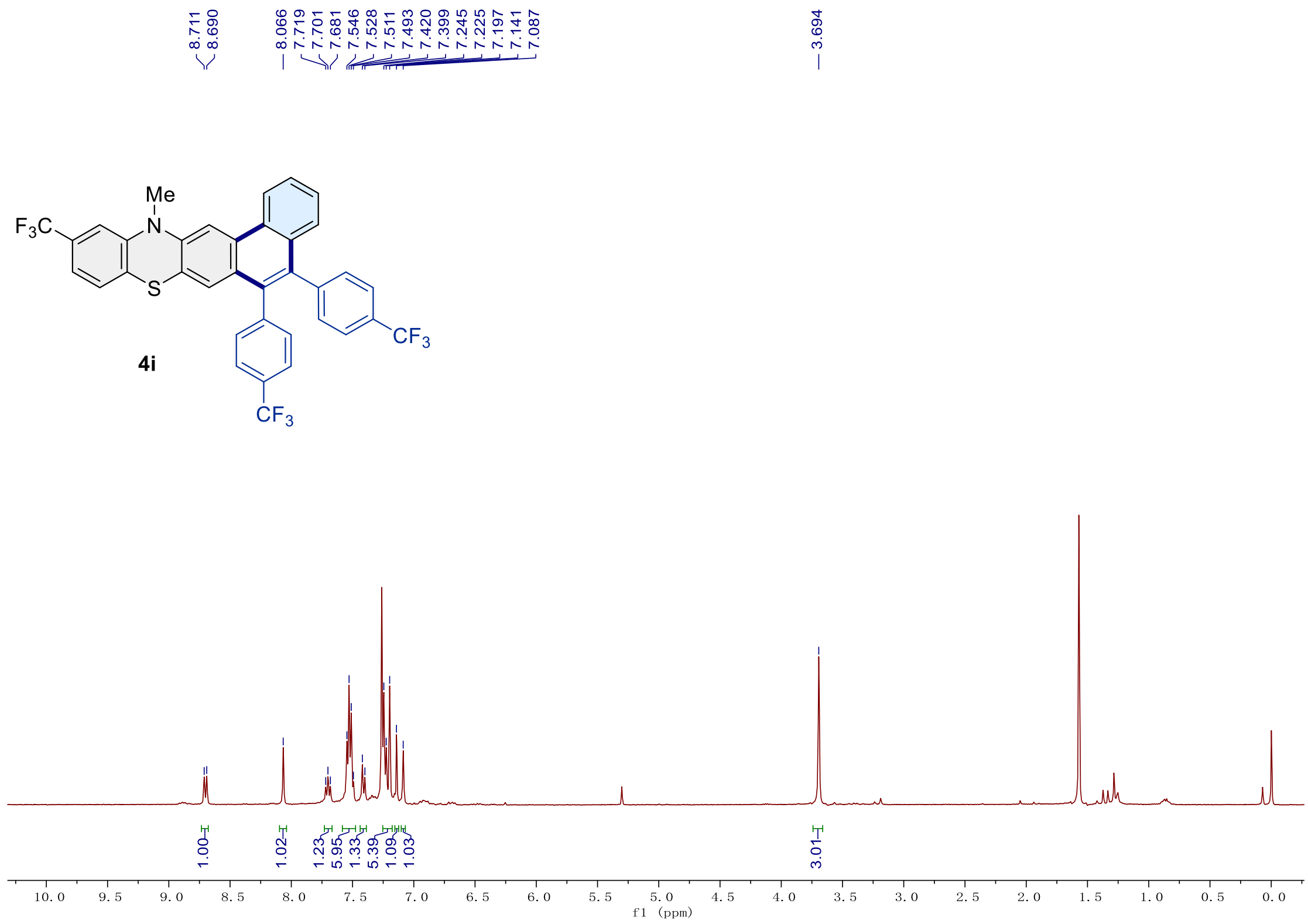

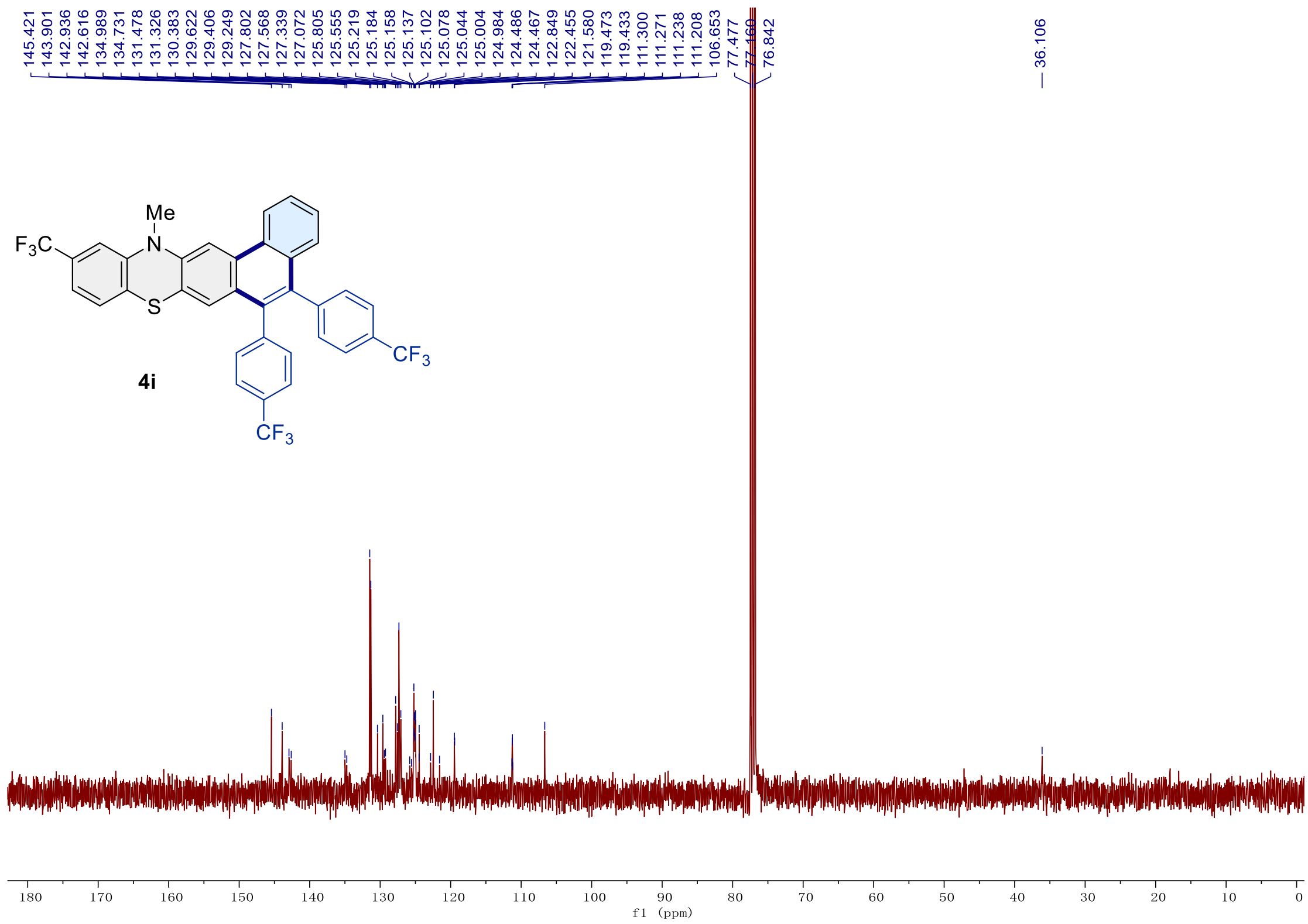

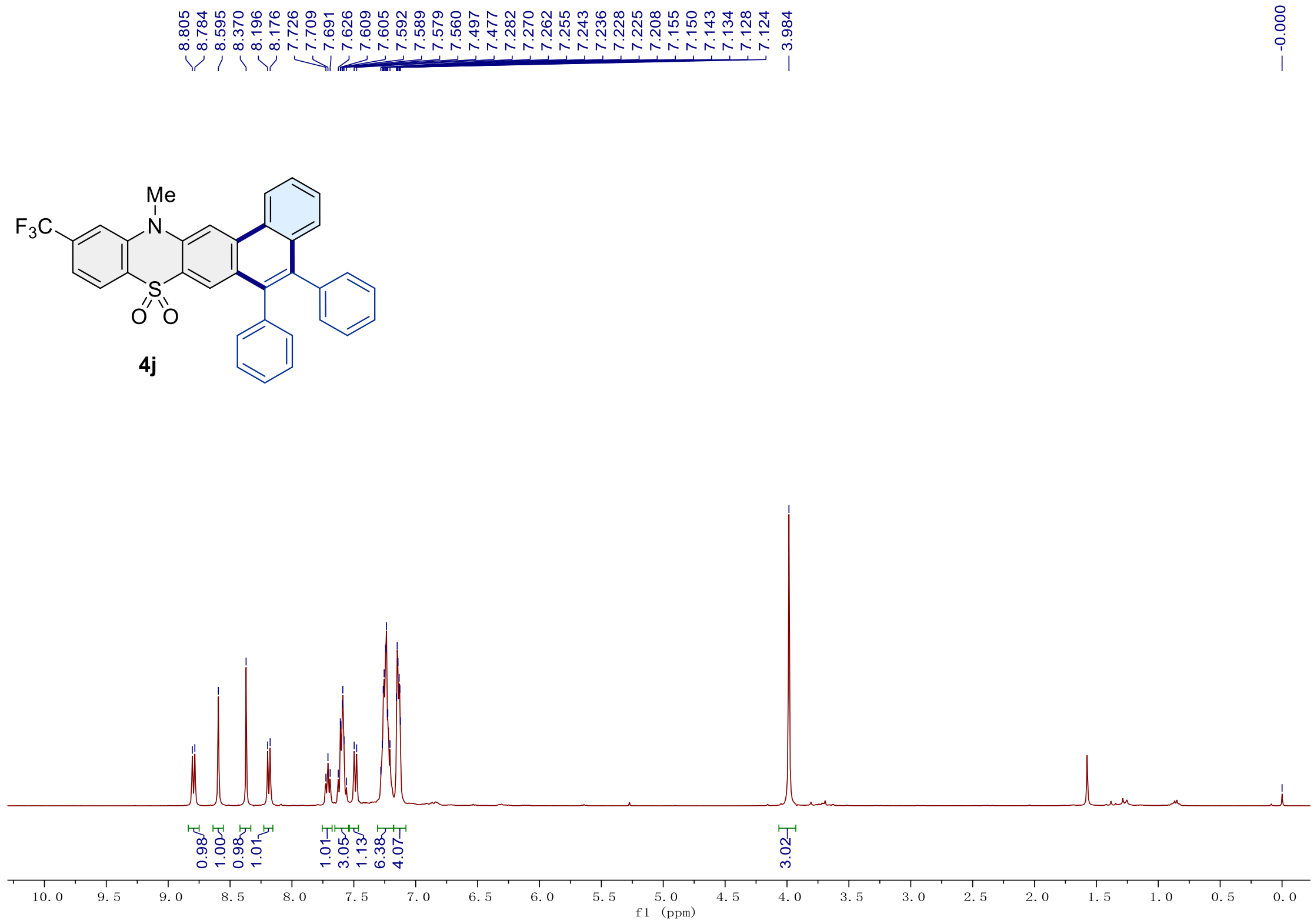
8.805
8.784
8.595
8.370
8.196
8.176
7.726
7.709
7.691
7.626
7.609
7.605
7.592
7.589
7.579
7.560
7.497
7.477
7.282
7.270
7.262
7.255
7.243
7.236
7.228
7.225
7.208
7.155
7.150
7.143
7.134
7.128
7.124
3.984
-0.000
Me
N
F3C
S
O O
4j
0.98
1.00
0.98
1.01
1.01
3.05
1.13
6.38
4.07
3.02
f1 (ppm)

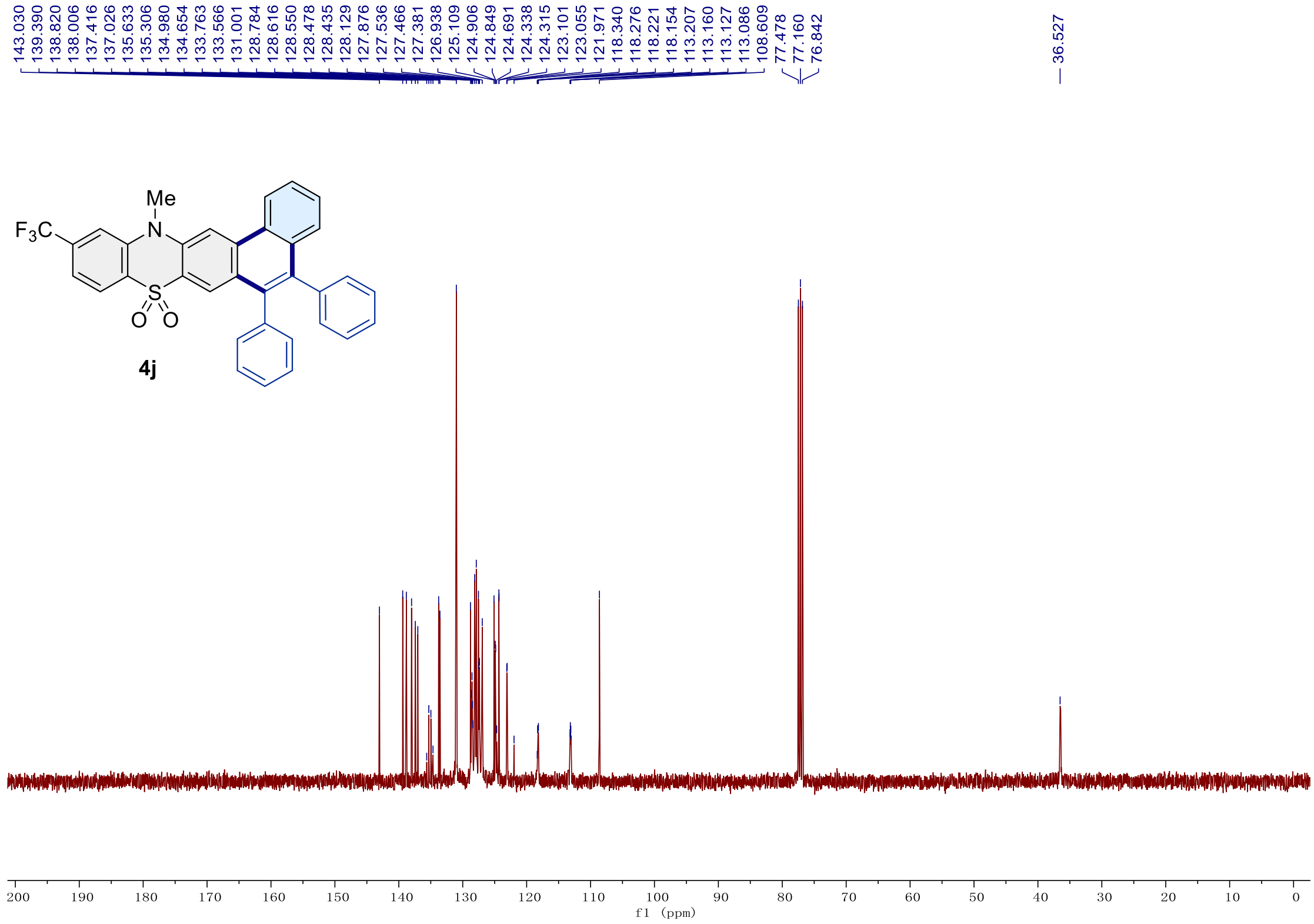
143.030
139.390
138.820
138.006
137.416
137.026
135.633
135.306
134.980
134.654
133.763
133.566
131.001
128.784
128.616
128.550
128.478
128.435
128.129
127.876
127.536
127.466
127.381
126.938
125.109
124.906
124.849
124.691
124.338
124.315
123.101
123.055
121.971
118.340
118.276
118.221
118.154
113.207
113.160
113.127
113.086
108.609
77.478
77.160
76.842
36.527
Me
N
F3C
S
O O
4j
f1 (ppm)

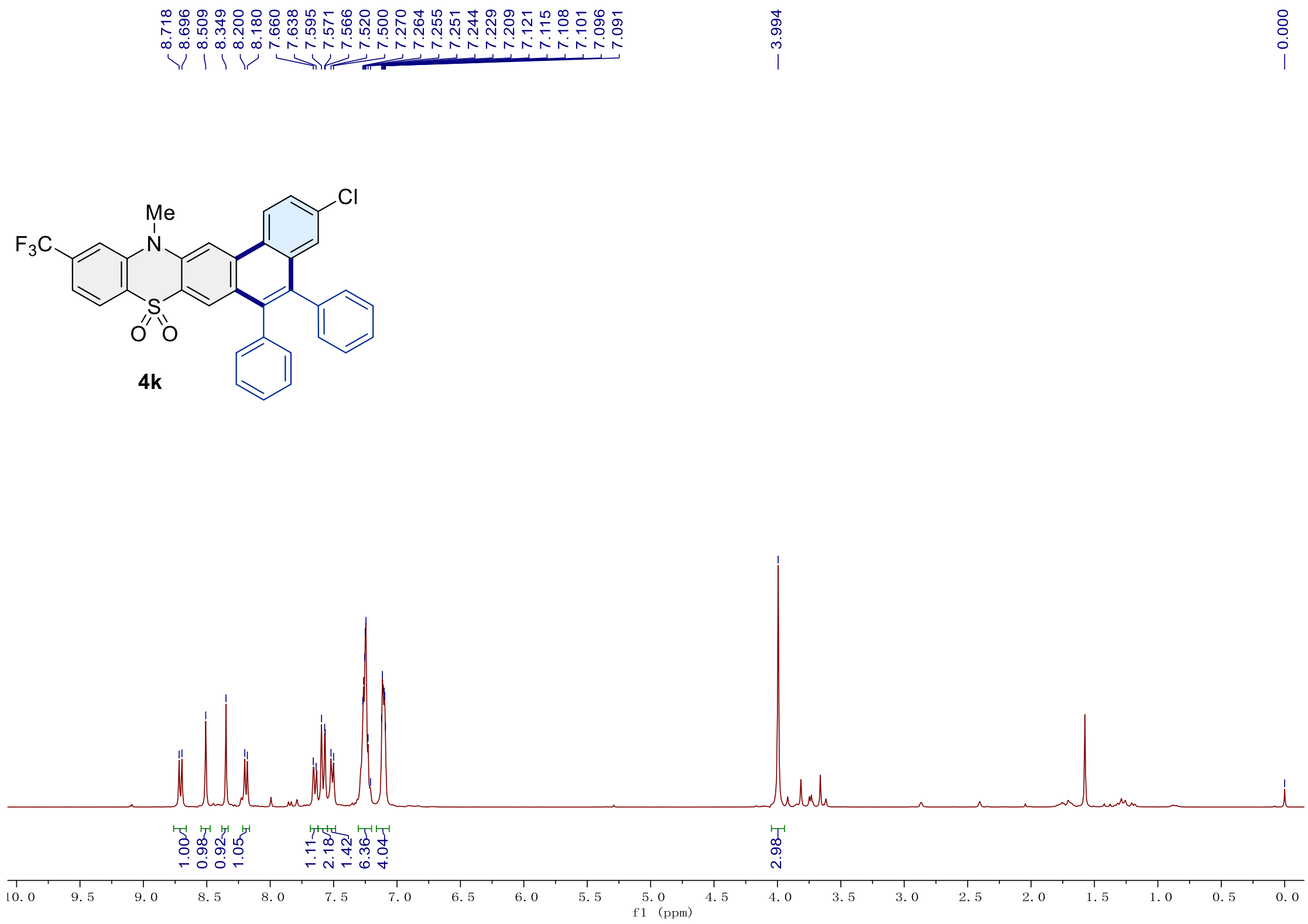
8.718
8.696
8.509
8.349
8.200
8.180
7.660
7.638
7.595
7.571
7.566
7.520
7.500
7.270
7.264
7.255
7.251
7.244
7.229
7.209
7.121
7.115
7.108
7.101
7.096
7.091
3.994
0.000
Me
N
F_3C
S
O O
Cl
4k
1.00
0.98
0.92
1.05
1.11
2.18
1.42
6.36
4.04
2.98
f1 (ppm)

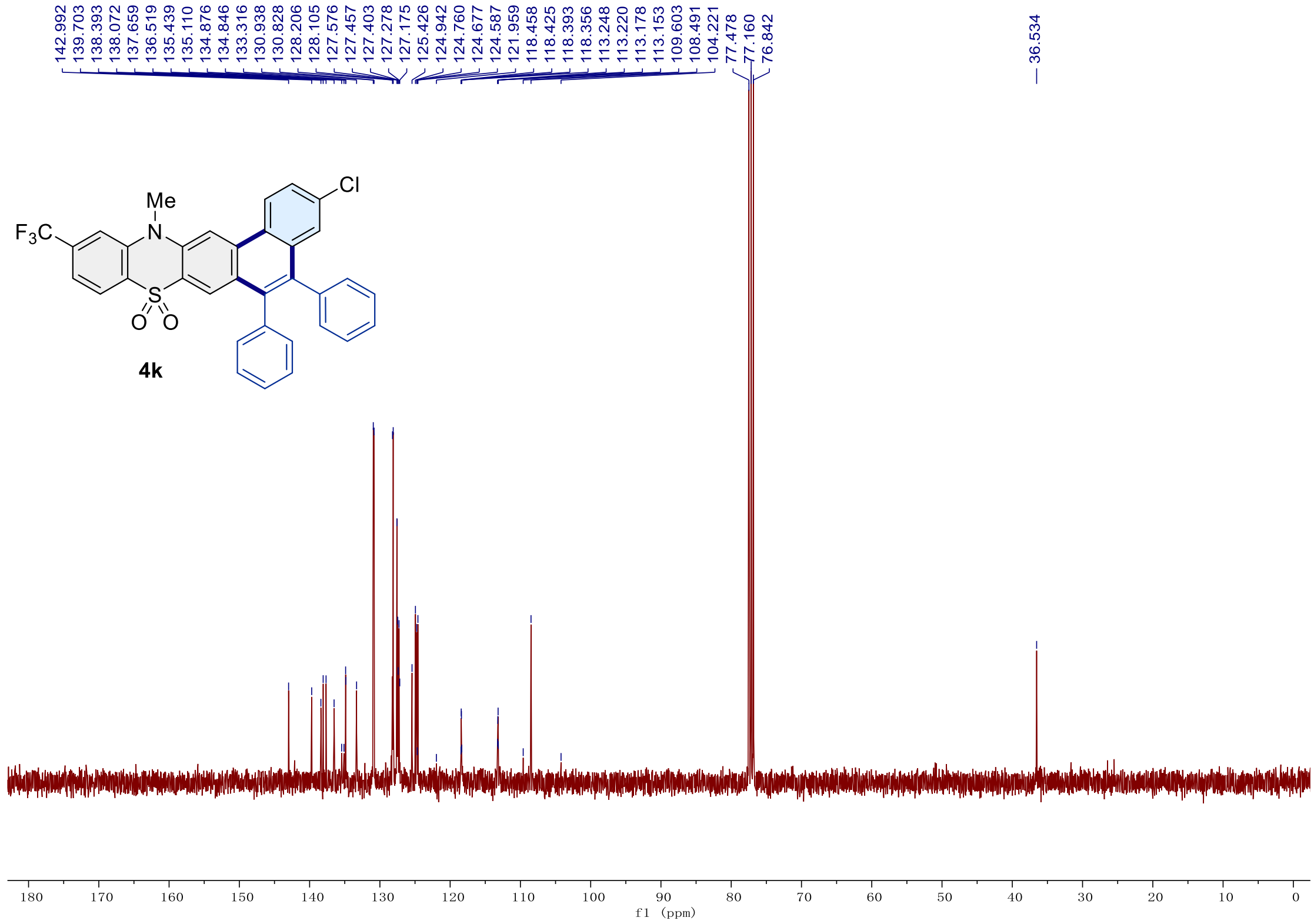
142.992
139.703
138.393
138.072
137.659
136.519
135.439
135.110
134.876
134.846
133.316
130.938
130.828
128.206
128.105
127.576
127.457
127.403
127.278
127.175
125.426
124.942
124.760
124.677
124.587
121.959
118.458
118.425
118.393
118.356
113.248
113.220
113.178
113.153
109.603
108.491
104.221
77.478
77.160
76.842
36.534
Me
N
F_3C
S
O O
Cl
4k
f1 (ppm)

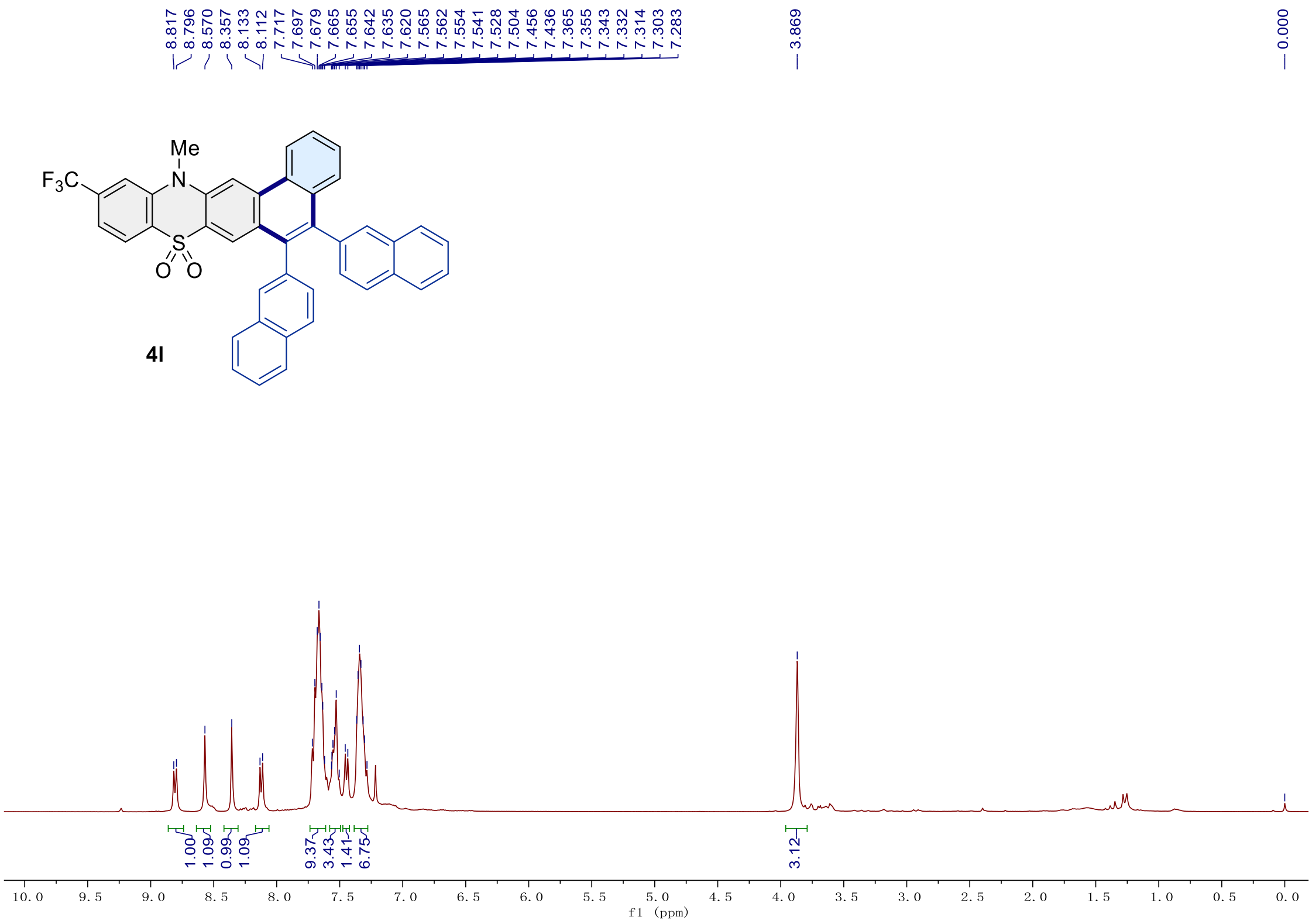
8.817 8.796 8.570 8.357 8.133 8.112 7.717 7.697 7.679 7.665 7.655 7.642 7.635 7.620 7.565 7.562 7.554 7.541 7.528 7.504 7.456 7.436 7.365 7.355 7.343 7.332 7.314 7.303 7.283
3.869
0.000
Me
N
F3C
S
O O
4l
1.00 1.09 0.99 1.09 9.37 3.43 1.41 6.75 3.12
f1 (ppm)

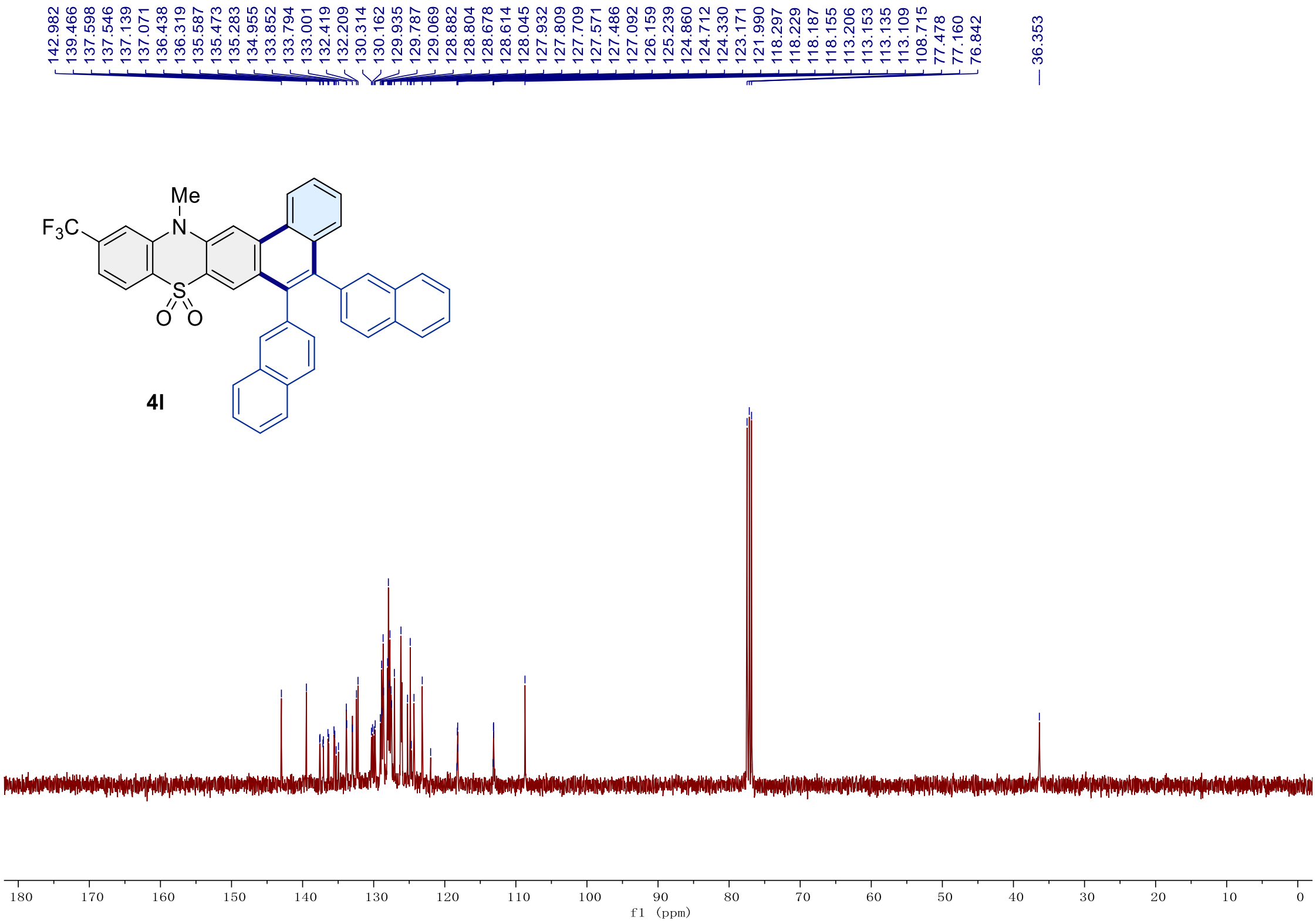
142.982 139.466 137.598 137.546 137.139 137.071 136.438 136.319 135.587 135.473 135.283 134.955 133.852 133.794 133.001 132.419 132.209 130.314 130.162 129.935 129.787 129.069 128.882 128.804 128.678 128.614 128.045 127.932 127.809 127.709 127.571 127.486 127.092 126.159 125.239 124.860 124.712 124.330 123.171 121.990 118.297 118.229 118.187 118.155 113.206 113.153 113.135 113.109 108.715 77.478 77.160 76.842
36.353
Me
N
F3C
S
O O
4l
f1 (ppm)

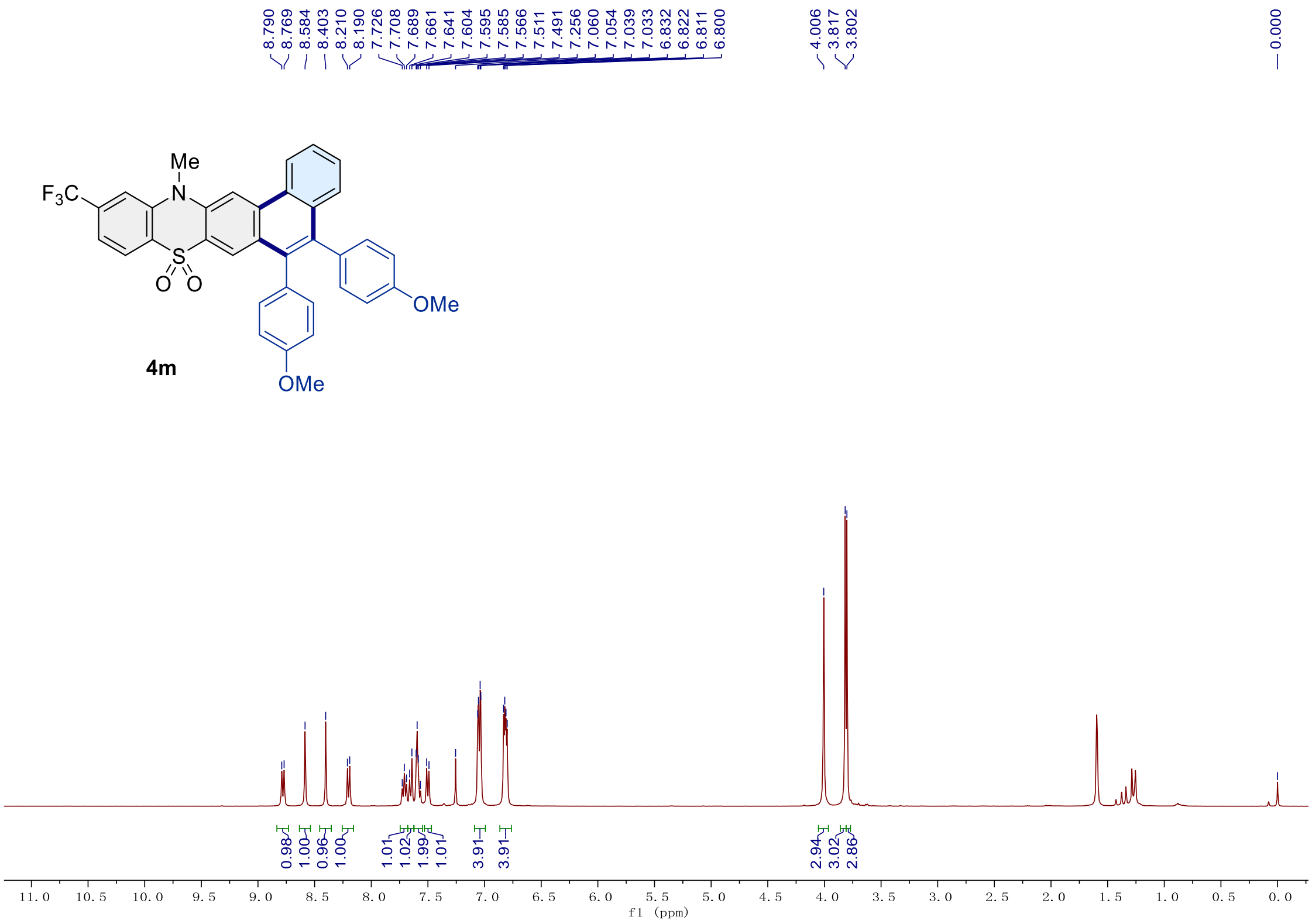
8.790
8.769
8.584
8.403
8.210
8.190
7.726
7.708
7.689
7.661
7.641
7.604
7.595
7.585
7.566
7.511
7.491
7.256
7.060
7.054
7.039
7.033
6.832
6.822
6.811
6.800
4.006
3.817
3.802
0.000
Me
N
F3C
S
O O
OMe
OMe
4m
0.98
1.00
0.96
1.00
1.01
1.02
1.99
1.01
3.91
3.91
2.94
3.02
2.86
11.0 10.5 10.0 9.5 9.0 8.5 8.0 7.5 7.0 6.5 6.0 5.5 5.0 4.5 4.0 3.5 3.0 2.5 2.0 1.5 1.0 0.5 0.0
f1 (ppm)

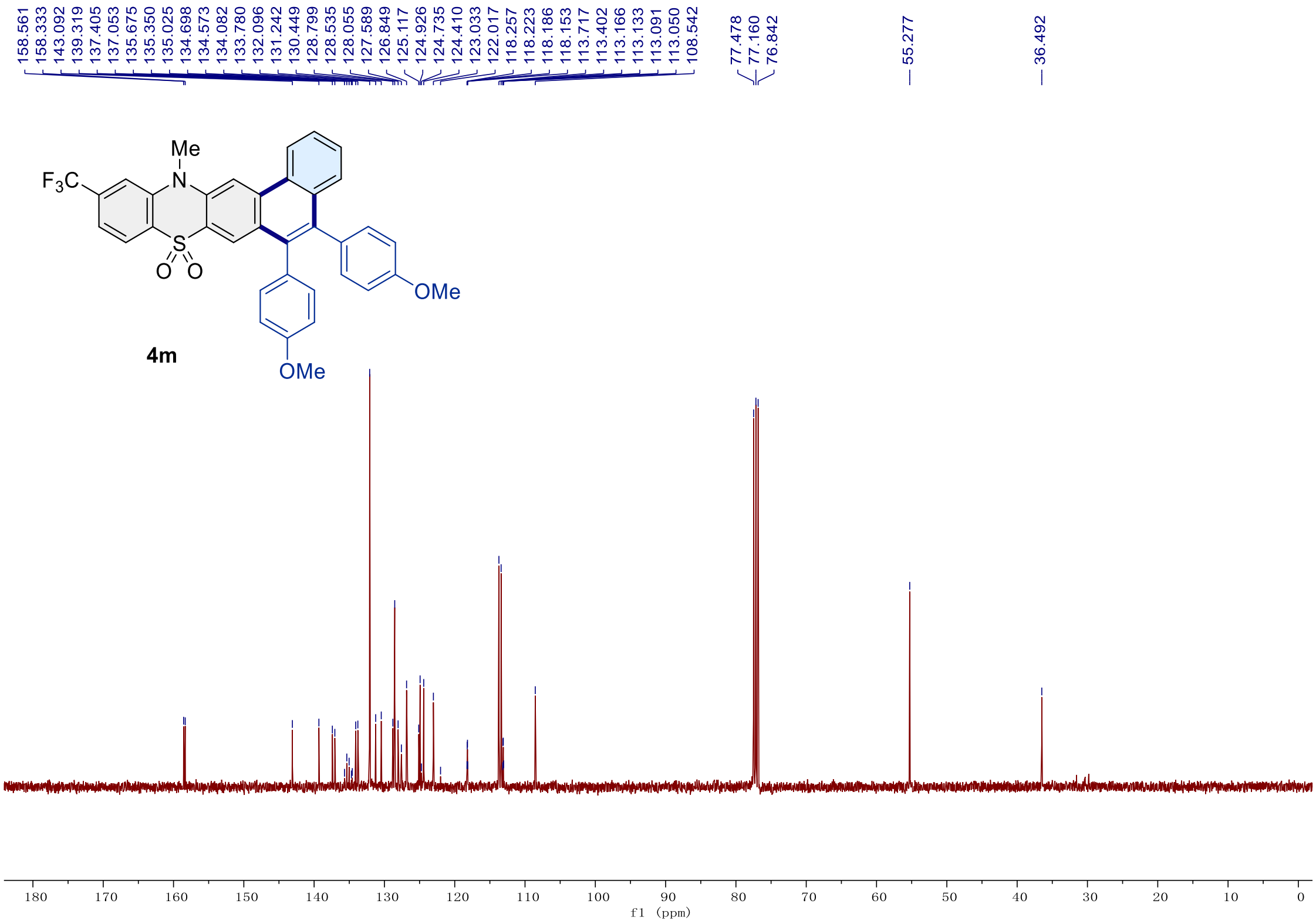
158.561
158.333
143.092
139.319
137.405
137.053
135.675
135.350
135.025
134.698
134.573
134.082
133.780
132.096
131.242
130.449
128.799
128.535
128.055
127.589
126.849
125.117
124.926
124.735
124.410
123.033
122.017
118.257
118.223
118.186
118.153
113.717
113.402
113.166
113.133
113.091
113.050
108.542
77.478
77.160
76.842
55.277
36.492
Me
N
F3C
S
O O
OMe
OMe
4m
180 170 160 150 140 130 120 110 100 90 80 70 60 50 40 30 20 10 0
f1 (ppm)

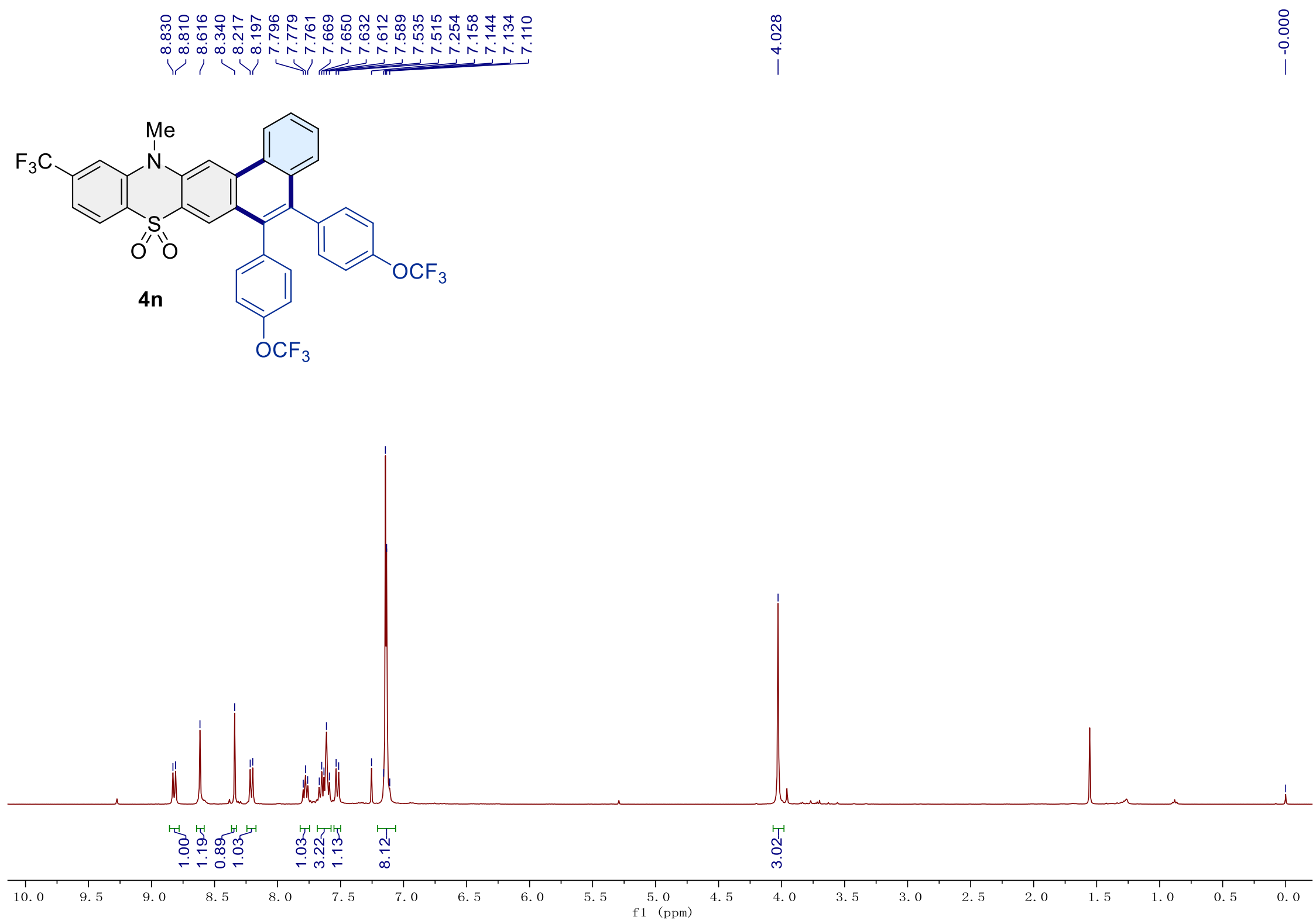
8.830
8.810
8.616
8.340
8.217
8.197
7.796
7.779
7.761
7.669
7.650
7.632
7.612
7.589
7.535
7.515
7.254
7.158
7.144
7.134
7.110
4.028
-0.000
Me
N
F3C
S
O O
4n
OCF3
OCF3
1.00
1.19
0.89
1.03
1.03
3.22
1.13
8.12
3.02
f1 (ppm)

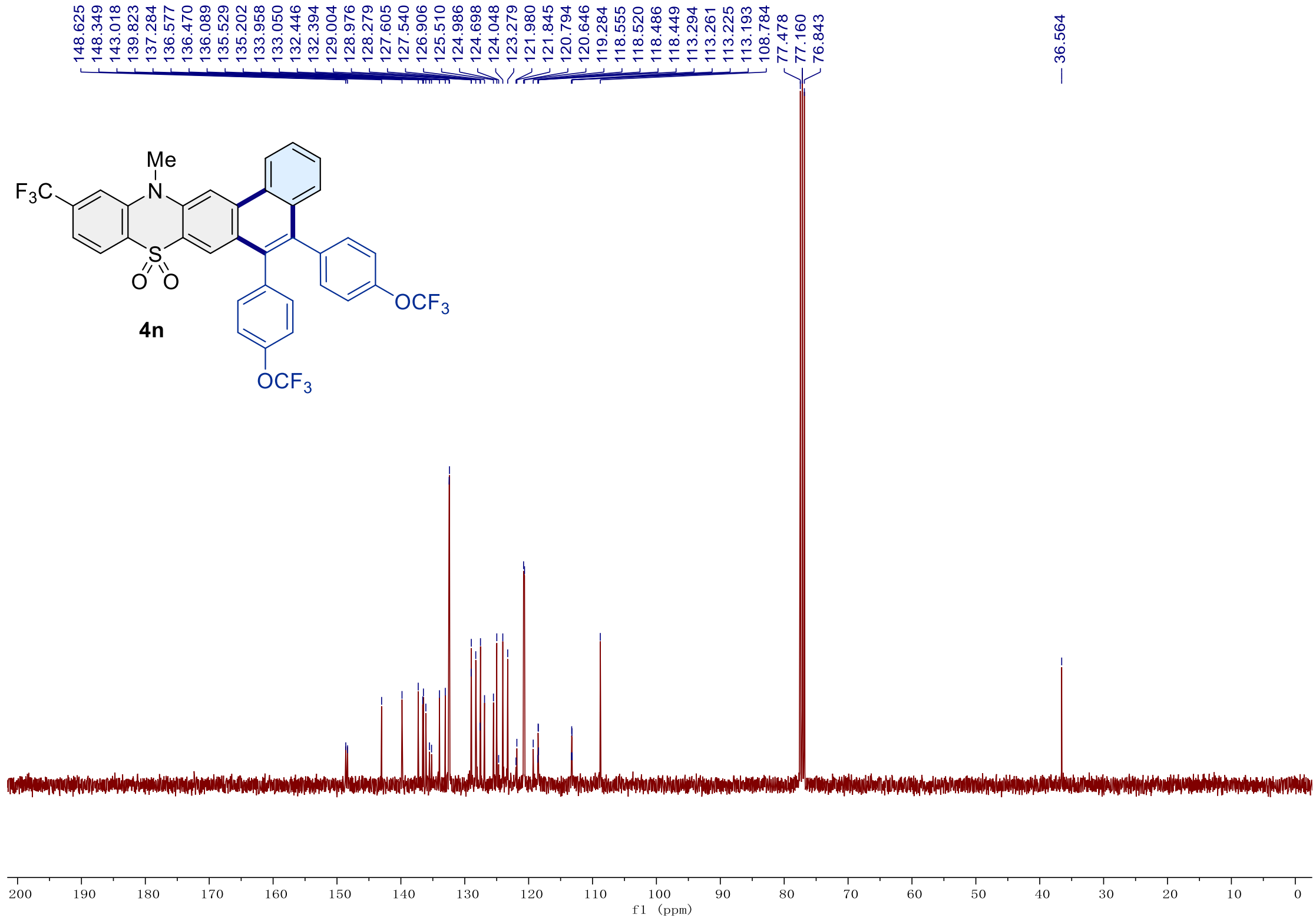
148.625
148.349
143.018
139.823
137.284
136.577
136.470
136.089
135.529
135.202
133.958
133.050
132.446
132.394
129.004
128.976
128.279
127.605
127.540
126.906
125.510
124.986
124.698
124.048
123.279
121.980
121.845
120.794
120.646
119.284
118.555
118.520
118.486
118.449
113.294
113.261
113.225
113.193
108.784
77.478
77.160
76.843
36.564
Me
N
F3C
S
O O
4n
OCF3
OCF3
f1 (ppm)

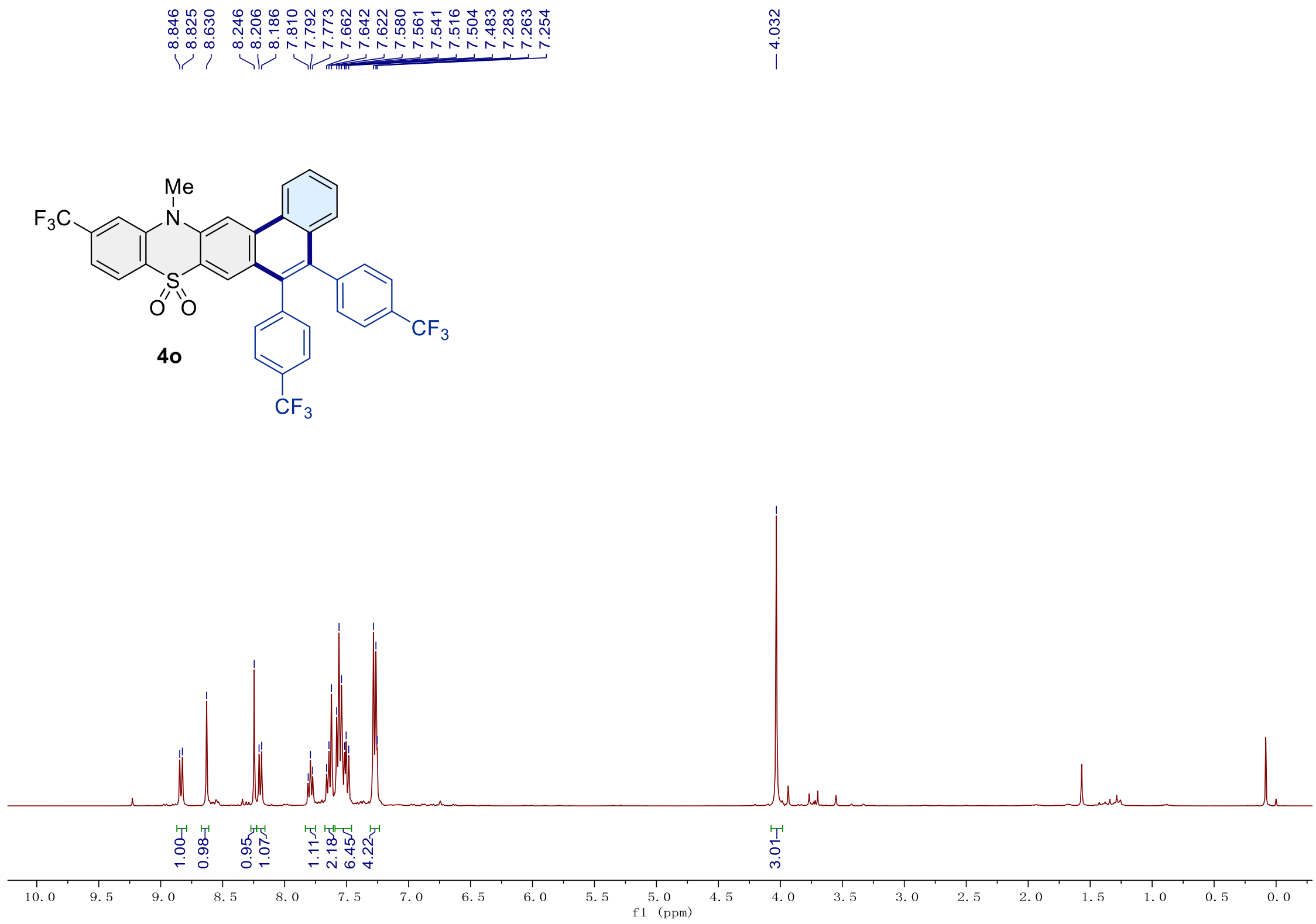

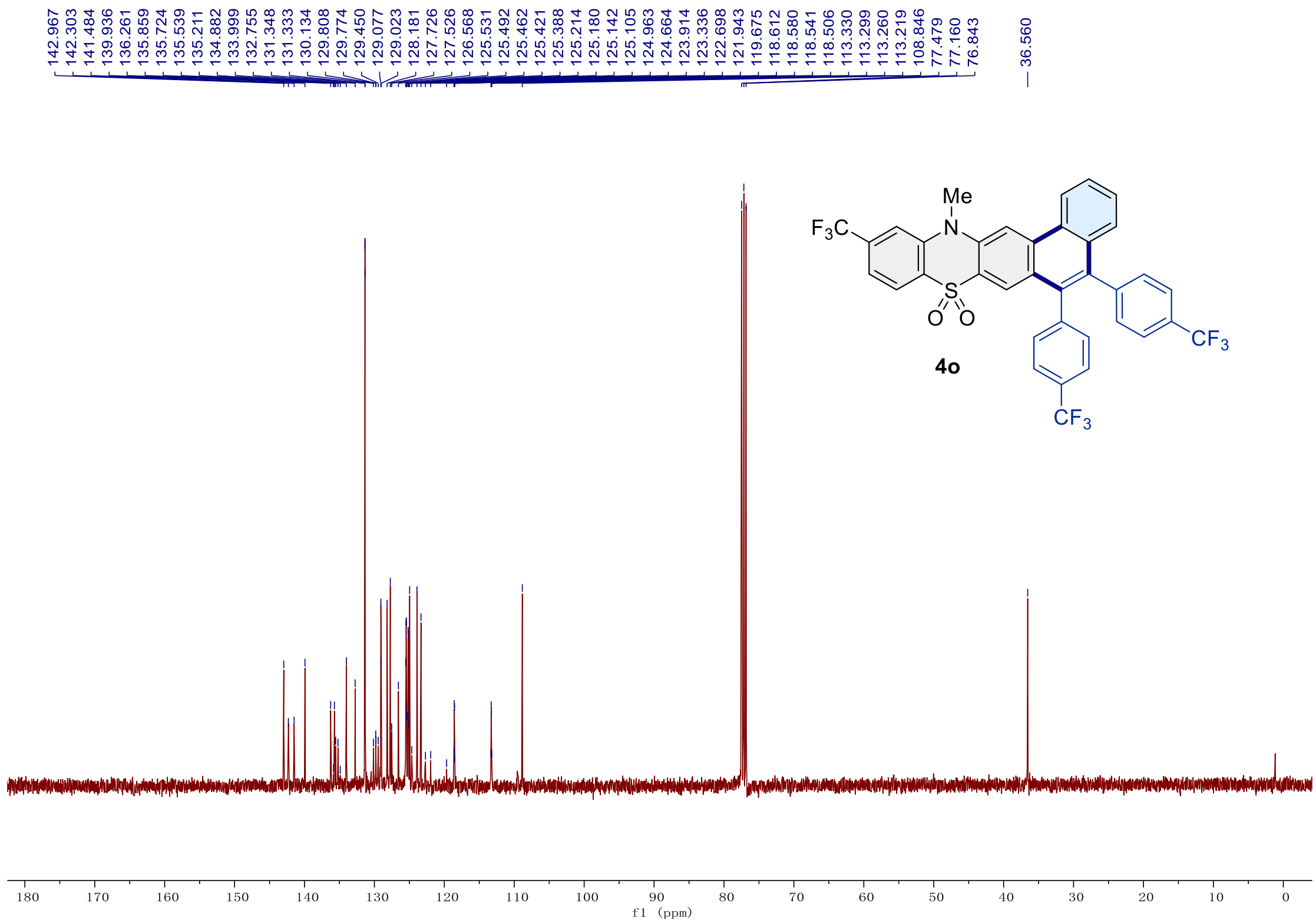

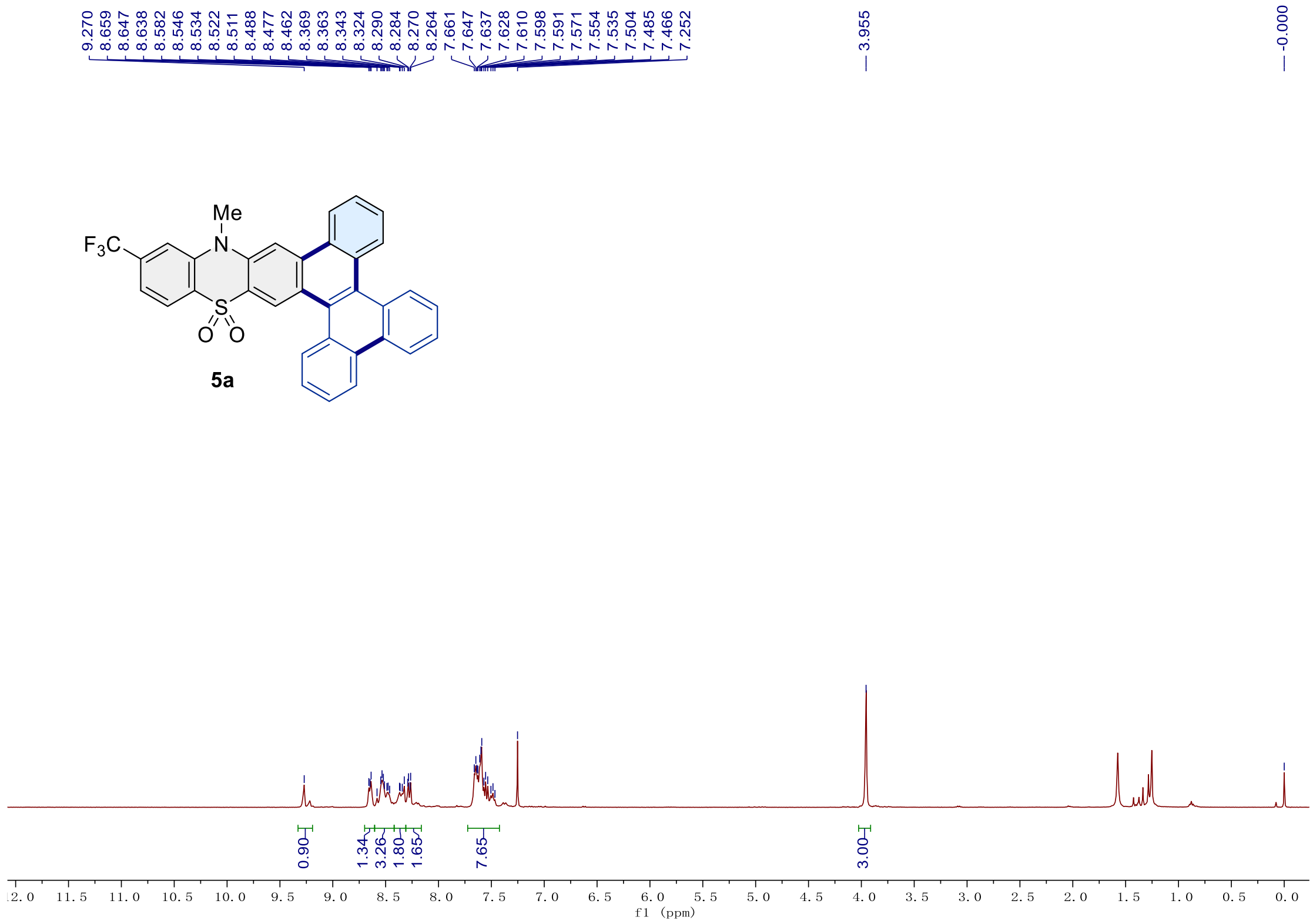
9.270
8.659
8.647
8.638
8.582
8.546
8.534
8.522
8.511
8.488
8.477
8.462
8.369
8.363
8.343
8.324
8.290
8.284
8.270
8.264
7.661
7.647
7.637
7.628
7.610
7.598
7.591
7.571
7.554
7.535
7.504
7.485
7.466
7.252
3.955
-0.000
Me
N
F3C
S
O O
5a
0.90
1.34
3.26
1.80
1.65
7.65
3.00
f1 (ppm)

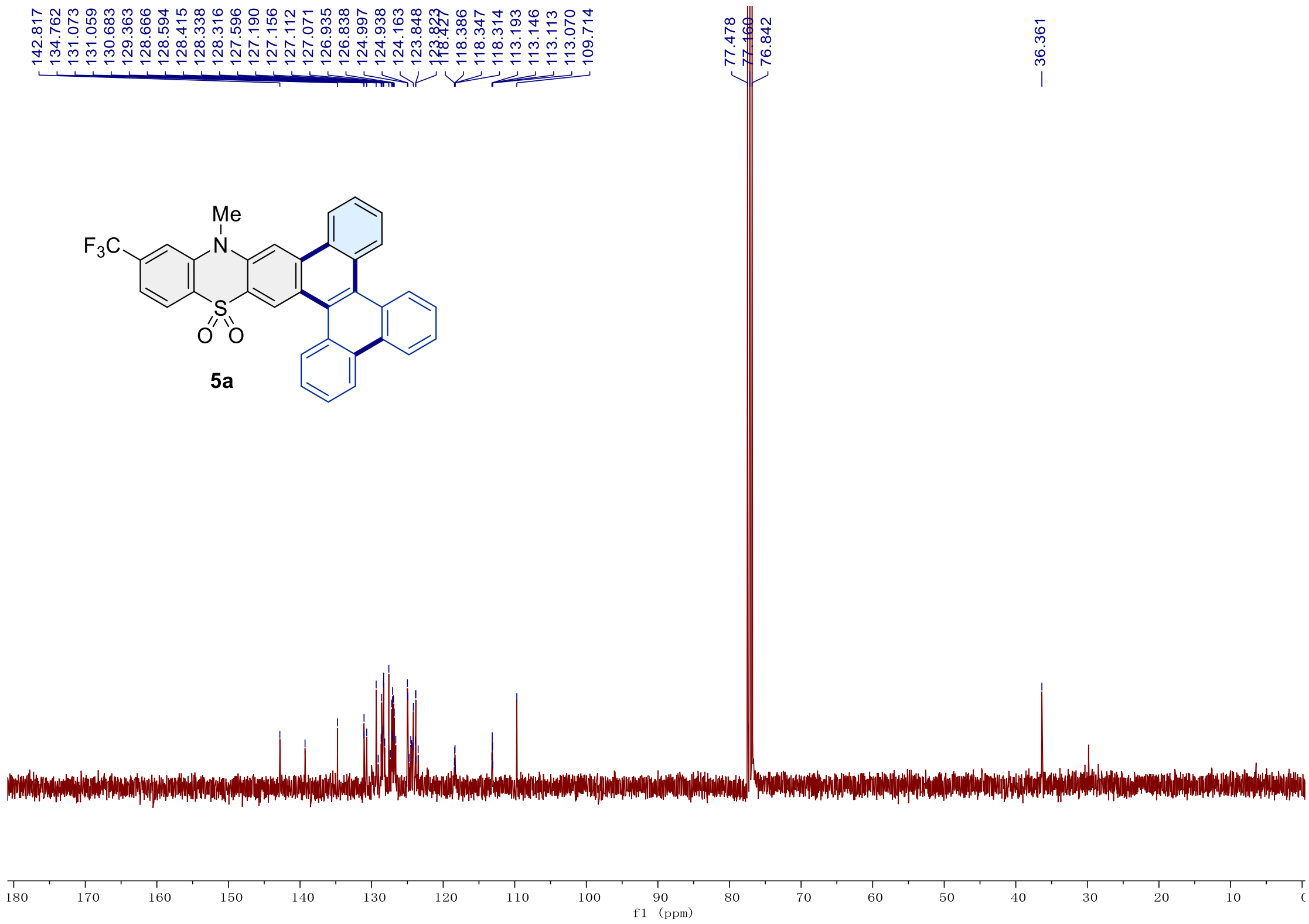
142.817
134.762
131.073
131.059
130.683
129.363
128.666
128.594
128.415
128.338
128.316
127.596
127.190
127.156
127.112
127.071
126.935
126.838
124.997
124.938
124.163
123.848
118.386
118.347
118.314
113.193
113.146
113.113
113.070
109.714
77.478
77.160
76.842
36.361
Me
N
F3C
S
O O
5a
f1 (ppm)

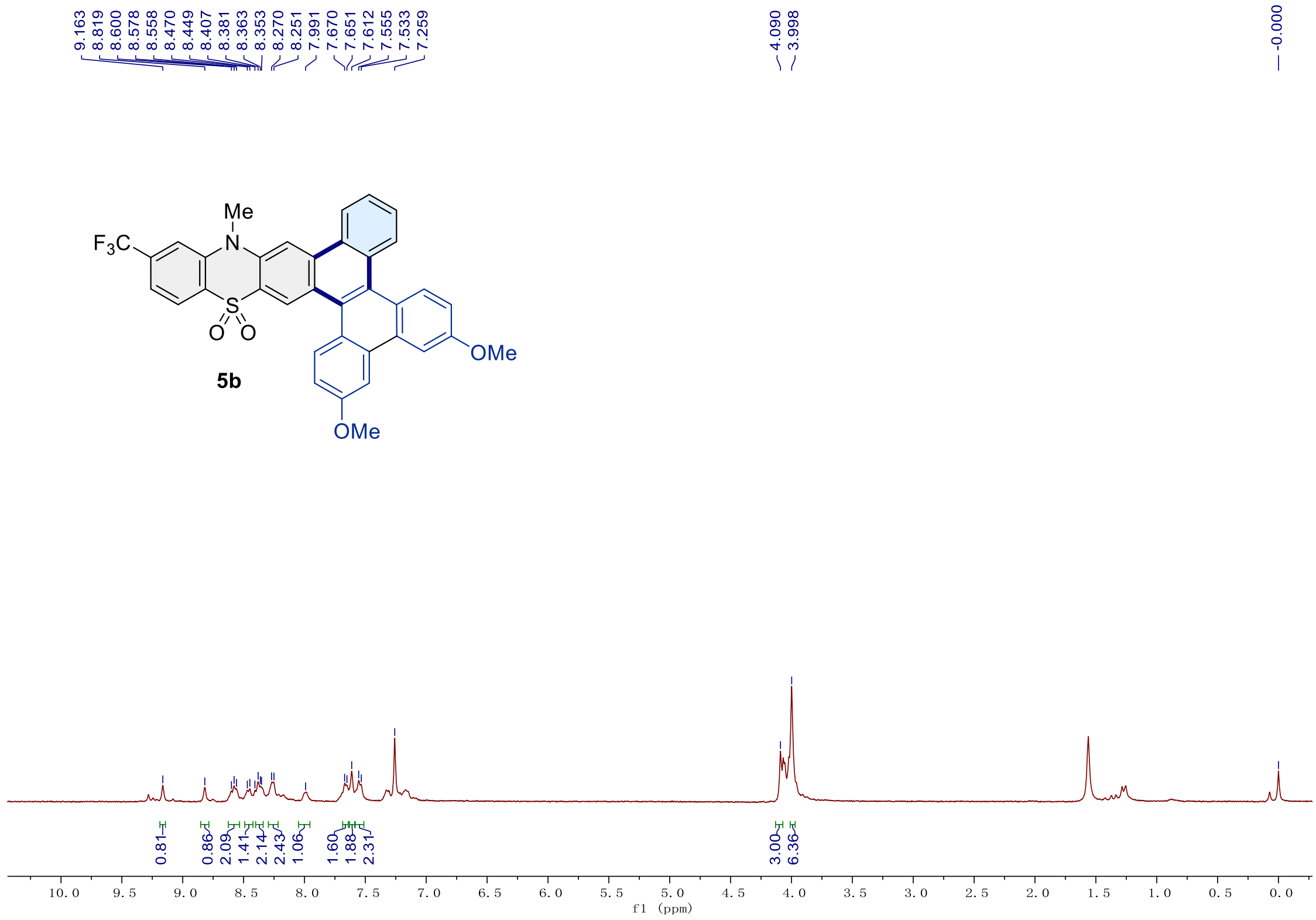
9.163
8.819
8.600
8.578
8.558
8.470
8.449
8.407
8.381
8.363
8.353
8.270
8.251
7.991
7.670
7.651
7.612
7.555
7.533
7.259
4.090
3.998
-0.000
Me
N
F3C
S
O
O
OMe
OMe
0.81
0.86
2.09
1.41
2.14
2.43
1.06
1.60
1.88
2.31
3.00
6.36
10.0
9.5
9.0
8.5
8.0
7.5
7.0
6.5
6.0
5.5
5.0
4.5
4.0
3.5
3.0
2.5
2.0
1.5
1.0
0.5
0.0
f1 (ppm)

5b

NMR spectrum of substrates 1c and 1d:

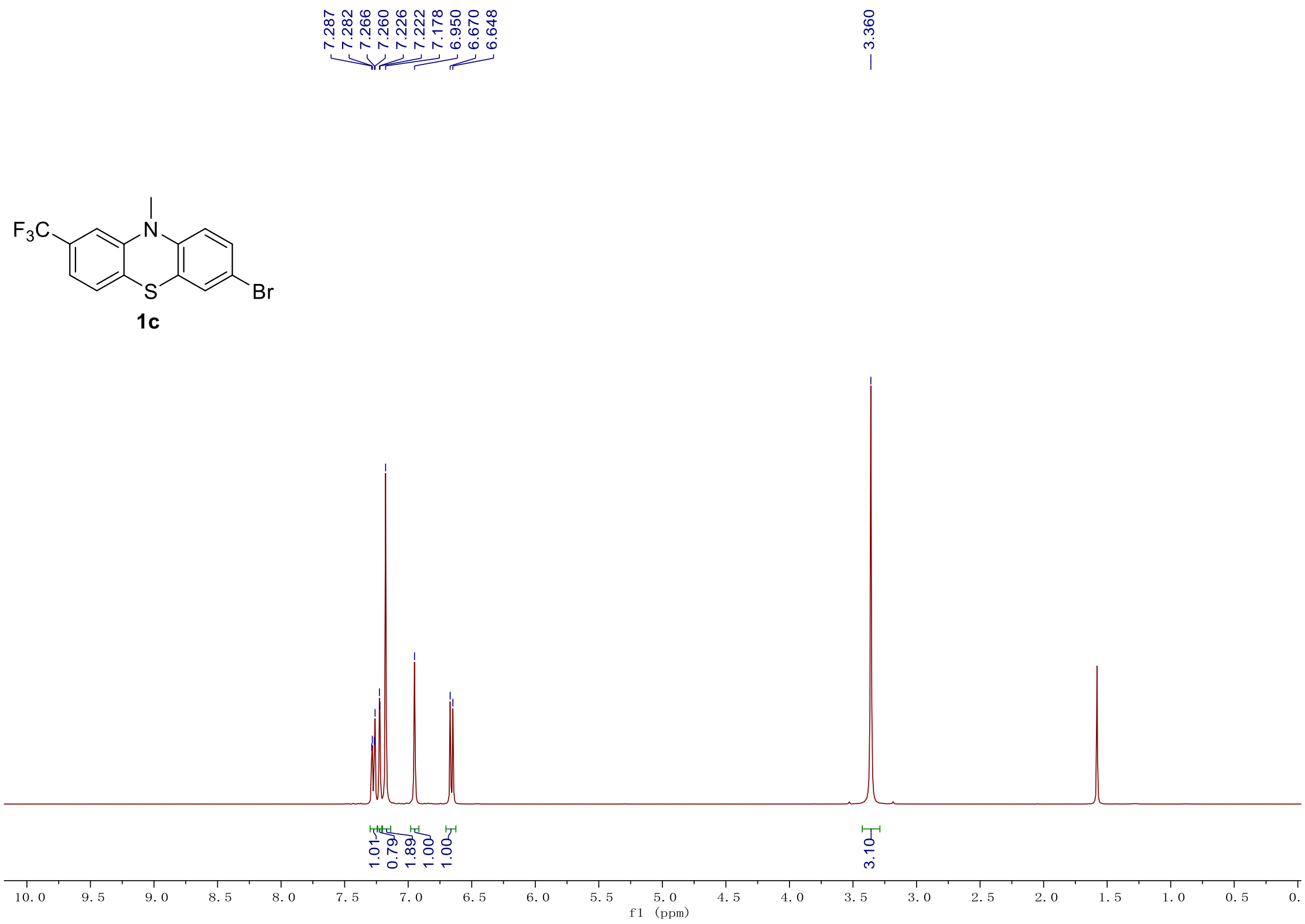

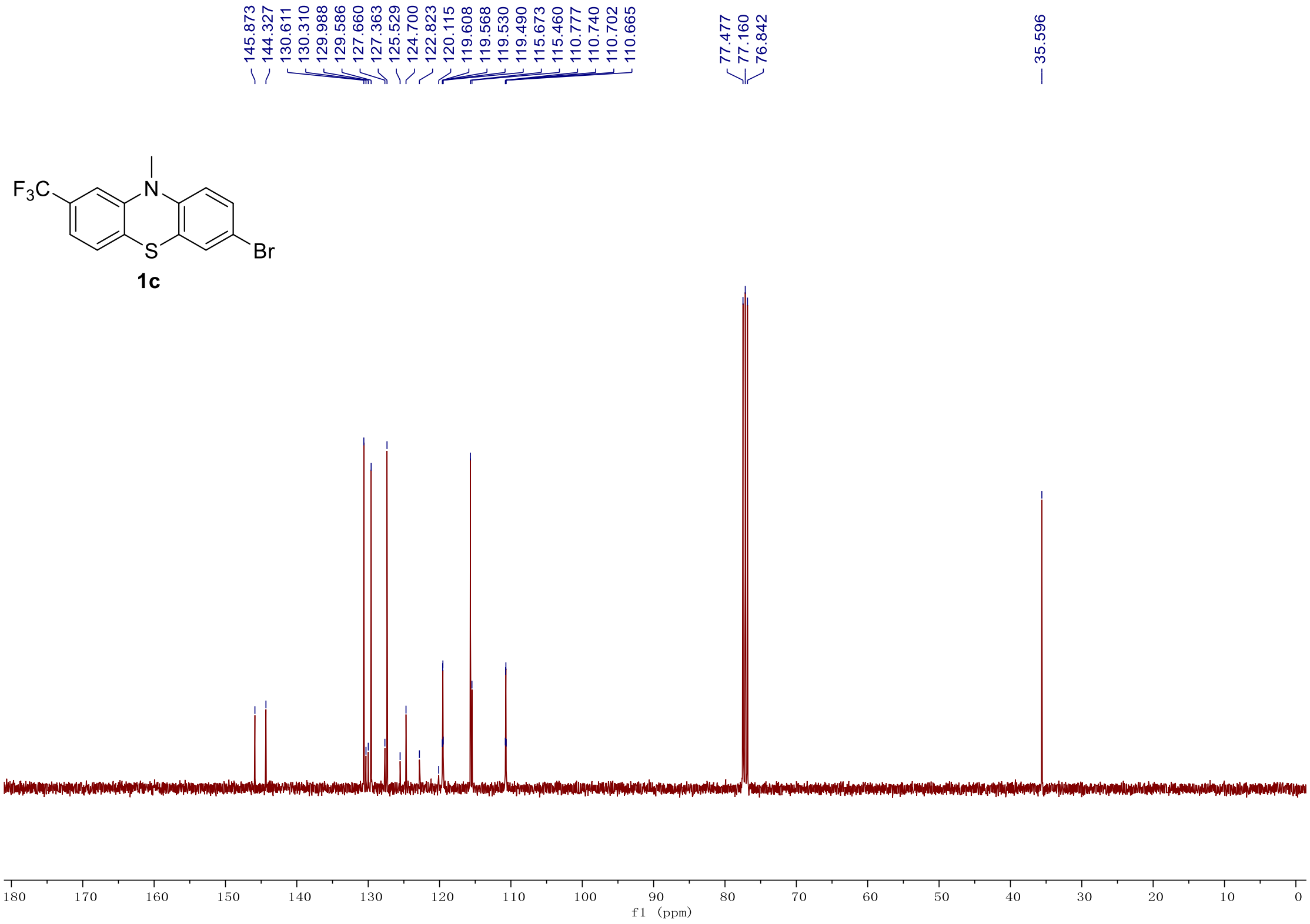

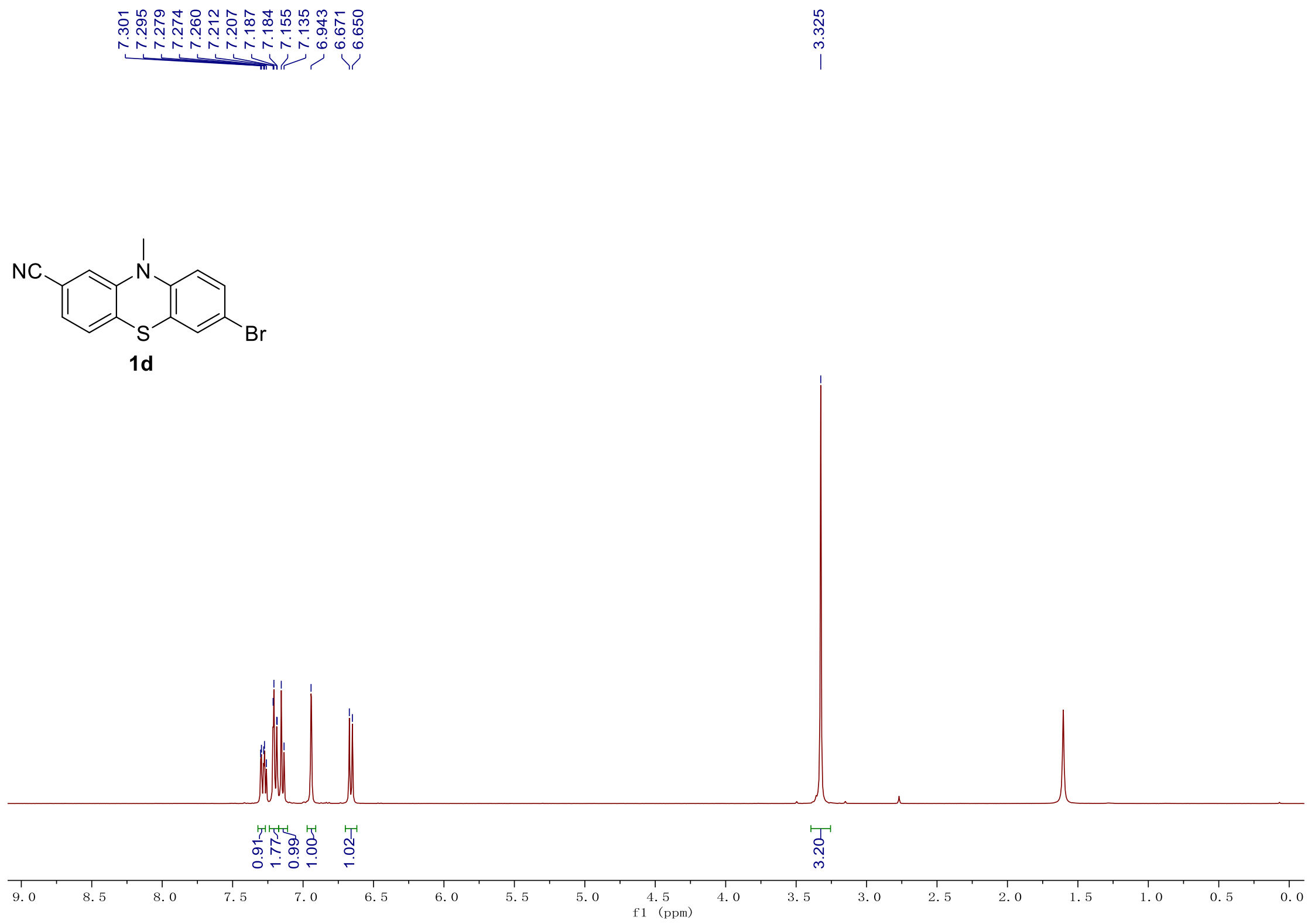
7.301
7.295
7.279
7.274
7.260
7.212
7.207
7.187
7.184
7.155
7.135
6.943
6.671
6.650
3.325
NC
N
S
Br
1d
0.91
1.77
0.99
1.00
1.02
3.20
9.0 8.5 8.0 7.5 7.0 6.5 6.0 5.5 5.0 4.5 4.0 3.5 3.0 2.5 2.0 1.5 1.0 0.5 0.0
f1 (ppm)

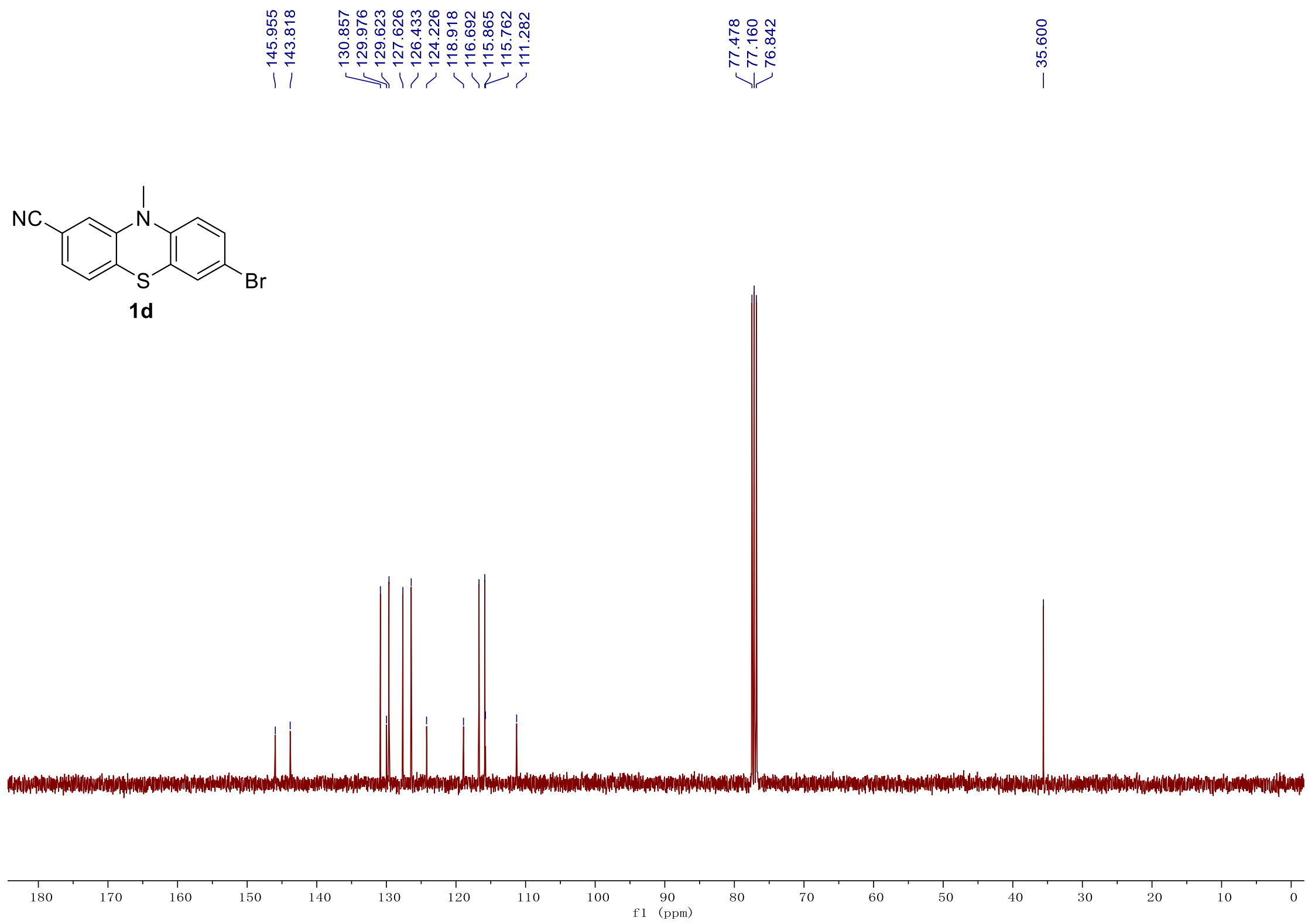
145.955
143.818
130.857
129.976
129.623
127.626
126.433
124.226
118.918
116.692
115.865
115.762
111.282
77.478
77.160
76.842
35.600
NC
N
S
Br
1d
180 170 160 150 140 130 120 110 100 90 80 70 60 50 40 30 20 10 0
f1 (ppm)

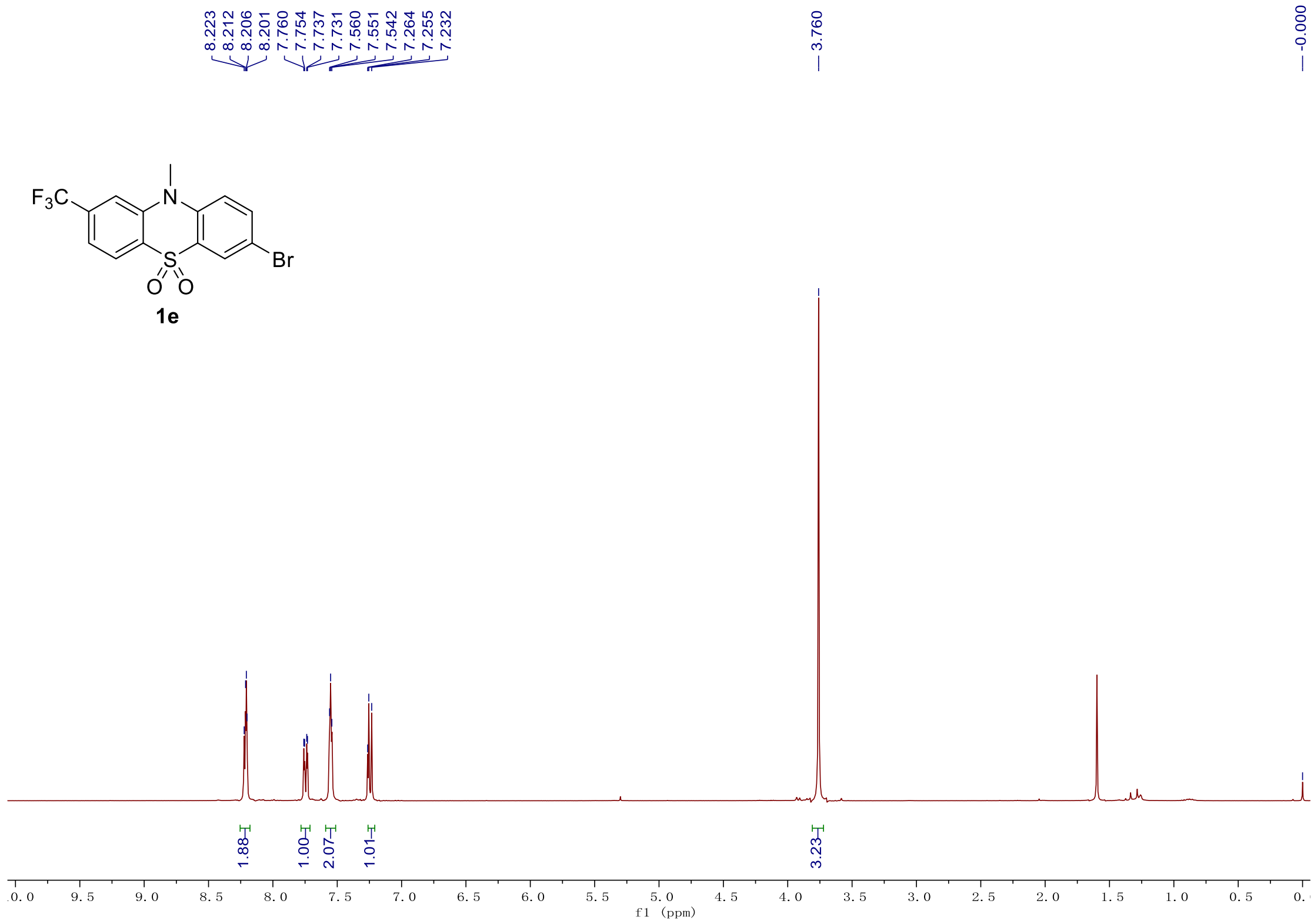
8.223
8.212
8.206
8.201
7.760
7.754
7.737
7.731
7.560
7.551
7.542
7.264
7.255
7.232
3.760
-0.000
F_3C
N
S
O O
Br
1e
1.88
1.00
2.07
1.01
3.23
f1 (ppm)

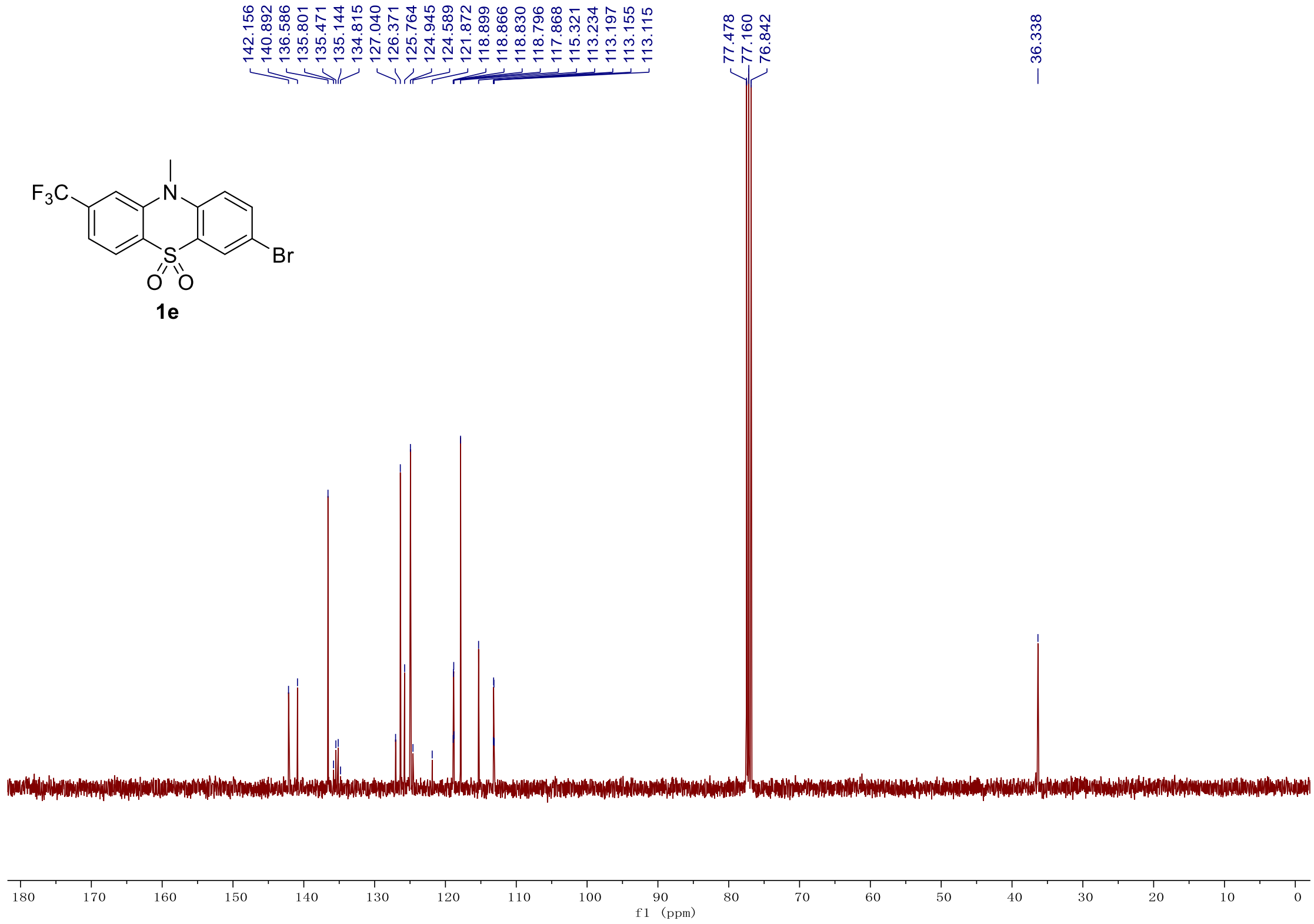
142.156
140.892
136.586
135.801
135.471
135.144
134.815
127.040
126.371
125.764
124.945
124.589
121.872
118.899
118.866
118.830
118.796
117.868
115.321
113.234
113.197
113.155
113.115
77.478
77.160
76.842
36.338
F_3C
N
S
O O
Br
1e
f1 (ppm)
